# Visible-light photoredox-catalyzed umpolung carboxylation of carbonyl compounds with CO_2_

**DOI:** 10.1038/s41467-021-23447-8

**Published:** 2021-06-03

**Authors:** Guang-Mei Cao, Xin-Long Hu, Li-Li Liao, Si-Shun Yan, Lei Song, Jason J. Chruma, Li Gong, Da-Gang Yu

**Affiliations:** 1 Key Laboratory of Green Chemistry and Technology of Ministry of Education, College of Chemistry, Sichuan University, Chengdu, China; 2 Department of Chemistry, University of Virginia, Charlottesville, VA USA; 3 Shanghai Key Laboratory of Green Chemistry and Chemical Processes, East China Normal University, School of Chemistry and Molecular Engineering, Shanghai, China

**Keywords:** Sustainability, Synthetic chemistry methodology, Photocatalysis

## Abstract

Photoredox-mediated umpolung strategy provides an alternative pattern for functionalization of carbonyl compounds. However, general approaches towards carboxylation of carbonyl compounds with CO_2_ remain scarce. Herein, we report a strategy for visible-light photoredox-catalyzed umpolung carboxylation of diverse carbonyl compounds with CO_2_ by using Lewis acidic chlorosilanes as activating/protecting groups. This strategy is general and practical to generate valuable α-hydroxycarboxylic acids. It works well for challenging alkyl aryl ketones and aryl aldehydes, as well as for α-ketoamides and α-ketoesters, the latter two of which have never been successfully applied in umpolung carboxylations with CO_2_ (to the best of our knowledge). This reaction features high selectivity, broad substrate scope, good functional group tolerance, mild reaction conditions and facile derivations of products to bioactive compounds, including oxypheonium, mepenzolate bromide, benactyzine, and tiotropium. Moreover, the formation of carbon radicals and carbanions as well as the key role of chlorosilanes are supported by control experiments.

## Abstract (web summary)

Compounds bearing a carbonyl group, such as aldehydes and ketones, are important industrial chemicals and widespread in pharmaceuticals and natural products. Here, the authors report a strategy for visible-light photoredox-catalyzed umpolung carboxylation of diverse carbonyl compounds with CO_2_, to generate valuable α-hydroxycarboxylic acids.

## Introduction

**Fig. 1:**
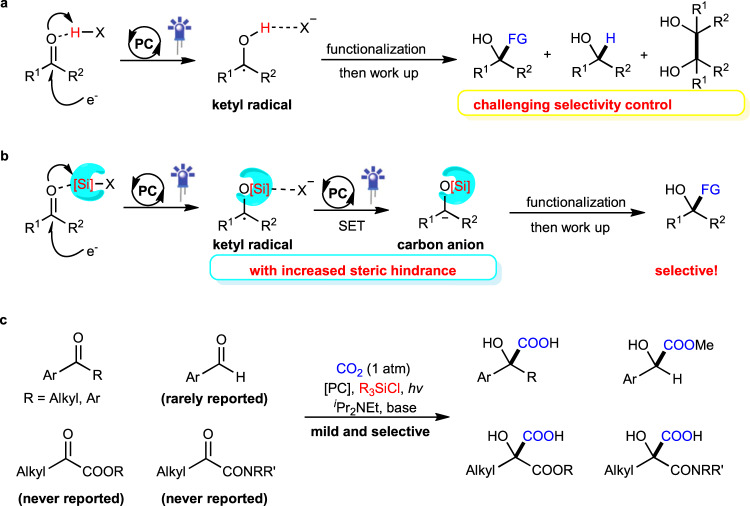
Strategies for umpolung functionalization of carbonyl compounds via photocatalysis. **a** Previous strategy: proton-coupled electron transfer via photocatalysis. **b** Our design: chlorosilane-coupled electron transfer via photocatalysis. **c** This work: a general umpolung carboxylation of carbonyls via photocatalysis. FG functional groups, PC photocatalyst.

Carbonyl compounds, especially ketones and aldehydes, are important bulk chemicals in industry and they exist widely in natural products, pharmaceuticals, and materials. The electronic structure of the carbonyl group renders it sensitive to attack from nucleophiles at the carbon center. Different from the inherent reactivity, the umpolung strategy has been investigated extensively to achieve C–C and C–X bond formations with non-nucleophilic reaction partners^1–3^. Notably, significant attention has been paid to recent progress in visible-light-driven umpolung reactions of carbonyl compounds owing to their mild reaction conditions and green processes^2,3^. In addition to direct single electron transfer (SET) reduction of carbonyls^4^, the photocatalytic proton-coupled electron transfer (PCET)^5^ approach represents a powerful tool for SET reduction of unactivated carbonyls to generate ketyl radicals (Fig. 1a), which then could undergo diverse transformations, including ketyl-olefin/alkyne coupling^6–10^, ketyl-ketyl homocoupling (pinacol coupling)^11^, and other couplings^12–16^. Such electron-rich ketyl radical intermediates, however, rarely undergo a second photocatalytic SET reduction process to produce a carbanionic species^17–20^. Meanwhile, the introduction of protons can accelerate pinacol coupling side reactions^20^. We envisioned the photoredox-catalyzed umpolung functionalization of carbonyl groups by using the Lewis acidic chlorosilane as an activating/protecting groups (Fig. 1b).

**Fig. 2:**
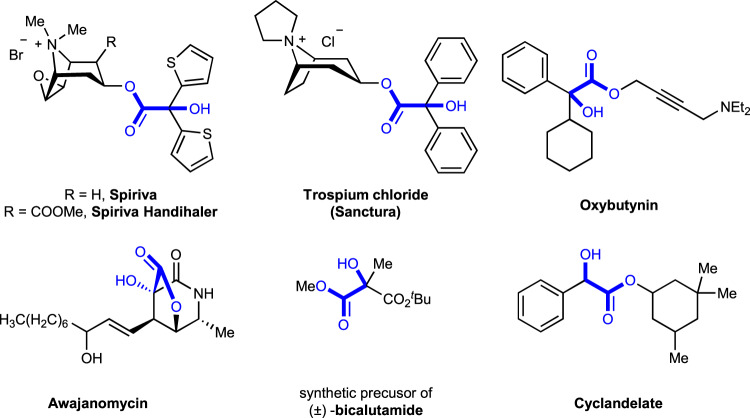
The significance of α-Hydroxycarboxylic acids. Selected examples of bioactive molecules and natural products that containing α-Hydroxycarboxylic acids moiety.

α-Hydroxycarboxylic acids are highly important motifs in pharmaceutical and polymer industries (Fig. 2)^21–29^. As traditional methods through cyanation and hydrolysis suffer from the use of toxic cyanide and harsh conditions, chemists have long searched for more sustainable synthetic routes^30^. Compared to cyanide and carbon monoxide, CO_2_ represents a better choice as a C1 source in carboxylation due to its abundance, nontoxicity, and recyclability^31–39^. Although significant progress has been achieved in recent decades to generate α-hydroxycarboxylic acids with CO_2_^18–20,40–43^, most of these methods suffer from the utilization of stoichiometric amounts of metal reductants, low functional group tolerance, limited substrate scope, and/or competitive side reactions, especially the pinacol coupling and reduction, thus hampering industrial application. Therefore, a general and practical method is desirable. Inspired by recent visible-light-driven reductive carboxylations of different electrophiles with CO_2_^44–59^, we wondered whether we could develop a general and practical strategy to achieve efficient and selective carboxylation of carbonyls with CO_2_ via visible-light photoredox catalysis. Achieving such a goal requires addressing several challenges. First, it is highly challenging to avoid common side reactions, namely pinacol coupling^11^ and hydrogen atom transfer (HAT) of ketyl radicals^60^. Second, as the direct radical addition of ketyl radicals to CO_2_ is less favored^61,62^, a second SET reduction to generate a carbanion, which could attack electrophilic CO_2_, would be promising. However, the direct SET reduction of electron-rich ketyl radicals and/or ketyl radical anions is challenging. Third, it is very difficult to avoid other processes, including direct α-carboxylation^63^ and homo-aldol reaction^64^ of alkyl ketones, as well as the disproportionation^65^ of aldehydes (Cannizzaro reaction) under basic conditions. Here, we show a general and practical reductive carboxylation of carbonyl compounds, such as alkyl aryl ketones, diaryl ketones, α-ketoamides, α-ketoesters, and aryl aldehydes, with CO_2_ to give valuable α-hydroxycarboxylic acids (Fig. 1c).

## Results

**Fig. 3:**
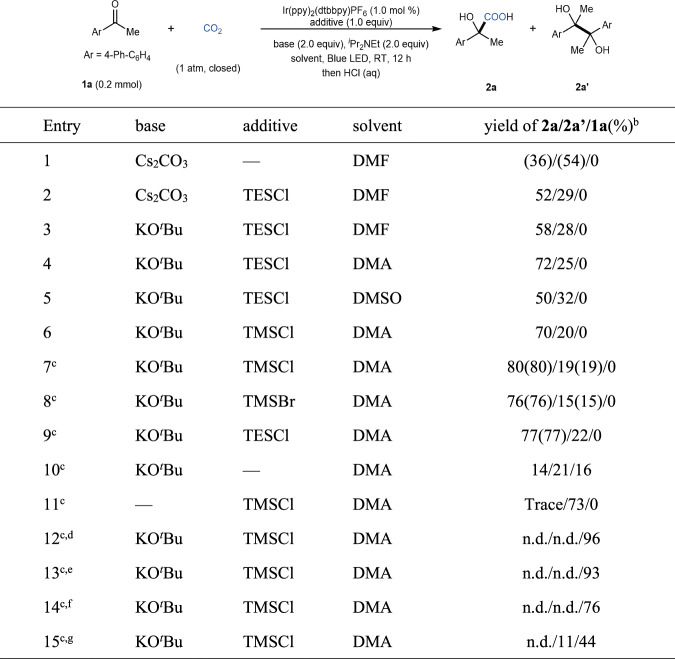
Reaction optimization. ^a^Reaction conditions: **1a** (0.2 mmol), Ir(ppy)_2_(dtbbpy)PF_6_ (1.0 mol %), base (2.0 equiv), ^*i*^Pr_2_NEt (2.0 equiv), additive (1.0 equiv) in solvent (2.0 mL) at room temperature (RT) in CO_2_ atmosphere under irradiation with 30 W blue LED for 12 h. The corresponding reductive product alcohol, 1-([1,1′-biphenyl]-4-yl)ethan-1-ol **2a**″, was observed as a minor byproduct in generally lower than 10% yield. ^b^Yields were determined by UPLC analysis with 1,1′-biphenyl as internal standard. The isolated yields are given in parentheses. ^c^Chlorosilane (1.3 equiv). ^d^Without light. ^e^Without Ir(ppy)_2_(dtbbpy)PF_6_. ^f^Without ^*i*^Pr_2_NEt. ^g^Under N_2_ atmosphere. n.d. not detected, LED light-emitting diode, DMSO dimethyl sulfoxide, dtbbpy 4,4′-di-*tert*-butyl-2,2′-bipyridine, ppy 2-phenylpyridine, TMS trimethylsilyl.

With such challenges in mind, we were inspired by PCET^5^ and hypothesized whether we could use a Lewis acidic chlorosilane instead of a proton as an activating group to promote the visible light-driven SET reduction of a carbonyl group, which has never been reported yet. Moreover, the activating chlorosilane might act as a temporary protecting group to generate α-silyloxy carbon radicals, which would increase the steric hindrance of the radical intermediates and thus retard undesired pinacol coupling. Based on this hypothesis, we started the investigation of reductive carboxylation with 4-acetylbiphenyl **1a** as a model substrate (Fig. 3). When we used Cs_2_CO_3_ as base, ^*i*^Pr_2_NEt as the electron donor and *N*,*N*-dimethylformamide (DMF) as solvent (entry 1), the desired product **2a** was obtained in 36% yield along with pinacol **2a′** in 54% yield. We were pleased to find that the use of triethylsilylchloride (TESCl) as an additive significantly improved the selectivity to afford **2a** in 52% yield and **2a′** in 29% yield (entry 2). After systematic screening of different bases, solvents, chlorosilanes, and other Lewis acids (entries 3–9. Please see Supplementary Information (SI) for more details), we found that the combination of trimethylsilylchloride (TMSCl) as additive, KO^*t*^Bu as base, and *N*,*N*-dimethylacetamide (DMA) as solvent was the best choice to give **2a** in 80% isolated yield (entry 7). Control experiments indicated that all of the components (PC, base, TMSCl, ^*i*^Pr_2_NEt, light, and CO_2_) played crucial roles in this successful transformation (entries 10–15).

**Fig. 4:**
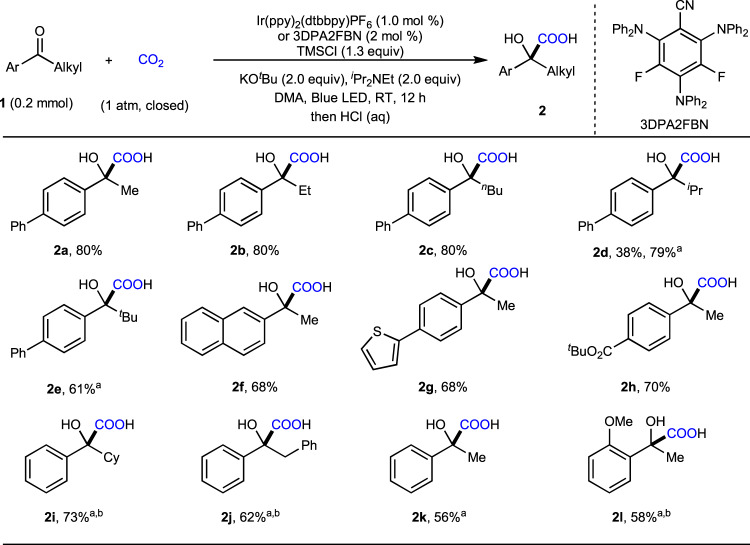
Substrate scope of alkyl aryl ketones. The standard conditions were used, as shown in Fig. 3, entry 7. Isolated yields of corresponding carboxylic acids **2** are shown. ^a^Using 3DPA2FBN (2.0 mol %) instead of Ir(ppy)_2_(dtbbpy)PF_6_. ^b^KO^*t*^Bu (2.5 equiv), DMA (4 mL).

With appropriate reaction conditions in hand, we began to explore the substrate scope of alkyl aryl ketones, which are prone to undergo α-carboxylation^63^ and homo-aldol reaction^64^ in the presence of base. To our delight, as shown in Fig. 4, ketones bearing different alkyl groups, such as methyl (**1a**, **1f**, **1g**, **1h**, **1k**, **1l**), ethyl (**1b**), *n*-butyl (**1c**), isopropyl (**1d**), *tert*-butyl (**1e**), cyclohexyl (**1i**), and benzyl (**1j**), all underwent the umpolung carboxylation selectively. Although increased steric hindrance on the alkyl groups induced lower reactivity, the desired products (**2d**, **2e**, **2i**) were obtained in good yields when 2,4,6-tris(diphenylamino)-3,5-difluorobenzonitrile (3DPA2FBN) was used as the photocatalyst. Notably, the umpolung carboxylation of the challenging substrate **1i** with CO_2_ to give **2i**, which is a key motif in oxybutynin (Fig. 2), had not been realized previously^21^. In addition to substrate **1h**, which is activated with an electron-withdrawing group (EWG), the effective conversion of substrates **1k** and **1l** bearing higher reduction potential (e.g., **1k** (*E*_1/2_^red^ = −2.1 V vs SCE)) could be achieved by using stronger reductive photosensitizer (3DPA2FBN, *E*_1/2_^red^ = −1.92 V vs SCE)^66^, especially in the presence of TMSCl as carbonyl activating reagent. Moreover, esters (**1h**), methoxy groups (**1l**), naphthalene (**1f**), and thiophene (**1g**) moieties were tolerated under the reaction conditions. However, the dialkyl ketones and aldehydes, such as 4-phenylbutan-2-one and 3-phenylpropanal, were performed in standard condition, no desirable products were detected so far. It is presumably due to the higher reduction potential of the dialkyl ketones and alkyl aldehydes.

**Fig. 5:**
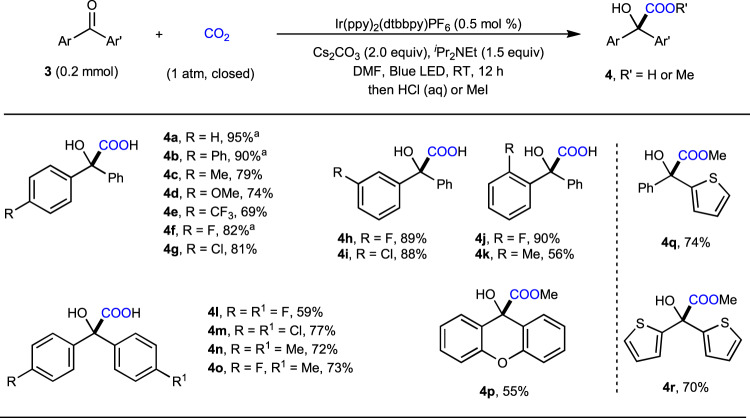
Substrate scope of diaryl ketones. Reaction condition: ketones (0.2 mmol), Ir-catalyst (0.001 mmol), Cs_2_CO_3_ (0.4 mmol), ^*i*^Pr_2_NEt (0.3 mmol), DMF (2 mL), isolated yields of corresponding carboxylic acids, or methyl esters **4** are shown. ^a*i*^Pr_2_NEt (1.0 equiv).

Considering the importance of α-hydroxycarboxylic acids bearing two aryl groups, we further applied our catalytic system to the carboxylation of diaryl ketones (Fig. 5). Due to the higher reactivity and fewer side reactions, these substrates could undergo reductive carboxylation smoothly under milder reaction conditions. A variety of functional groups, such as trifluoromethyl (**3e**), fluoro (**3f**, **3l**, **3o**), chloro (**3g**, **3m**), and methoxyl (**3d**), were tolerated at the *para* position of non-symmetric or symmetric diaryl ketones. Moreover, benzophenones bearing *meta*- (**3h**, **3i**) or *ortho*-substitutions (**3j**, **3k**) could afford the corresponding products in moderate to high yields (56–90%). Notably, the carboxylation of the benzylic C–H bond in **3k** was not observed through HAT process by ketyl radical^67^. In addition to benzophenones, this method could be expanded to xanthone **3p**, 2-benzoylthiophene **3q** and di(thiophen-2-yl)methanone **3r**.

**Fig. 6:**
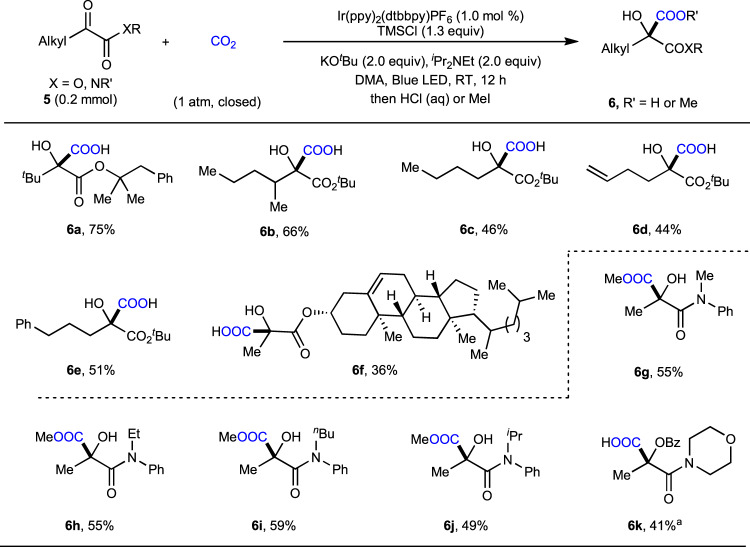
Substrate scope of α-ketoesters and α-ketoamides. The standard conditions were used, as shown in Fig. 3, entry 7. Isolated yields of corresponding carboxylic acids or methyl esters **6** are shown. ^a^Isolated yield of corresponding benzoate ester upon work up with BzCl. Bz benzoyl.

As α-hydroxymalonic acid (tartronic acid) derivatives are important motifs in a range of natural products, the development of methods to synthesize them is imperative. The construction of such motifs via the umpolung carboxylation of α-ketoesters with CO_2_ has not been reported. Therefore, we further tried to apply this strategy in generation of α-hydroxymalonic acid derivatives. To our delight, this catalytic system worked well for selective umpolung carboxylation of α-ketoesters (Fig. 6). The challenging sterically hindered *tert*-butyl-substituted α-ketoester **5a** showed good reactivity under standard reaction conditions to give the desired product **6a** in 75% yield. Moreover, α-ketoesters bearing secondary (**5b**) and primary alkyl groups (**5c**–**5e**) also were suitable substrates to afford the desired products in moderate to good yields. Notably, an α-hydroxymalonic acid derivative containing a cholesterol moiety (**6f**) was selectively obtained albeit in a diminished yield, demonstrating potential application of this strategy for late-stage diversification under mild conditions.

Based on the success of α-ketoesters, we further tested the umpolung carboxylation of α-ketoamides with CO_2_, a process which has never been reported. Indeed, *N*-alkyl-*N*-aryl amides (**5g**–**5j**) and *N*,*N*-dialkyl amide (**5k**) also reacted under the standard reaction conditions to afford the corresponding α-hydroxyamides in moderate yields. The steric hindrance of the alkyl group did not significantly affect the reaction, as demonstrated by the comparable yields for the carboxylations of *N*-alkyl-*N*-aryl amides (**5g**–**5j**). However, amides containing an N*–*H bond were not suitable substrates at this stage, which might because of the increased electron density of the carbonyl and/or the competitive quenching of deprotonated form of secondary amide with respect to ^*i*^Pr_2_NEt.

**Fig. 7:**
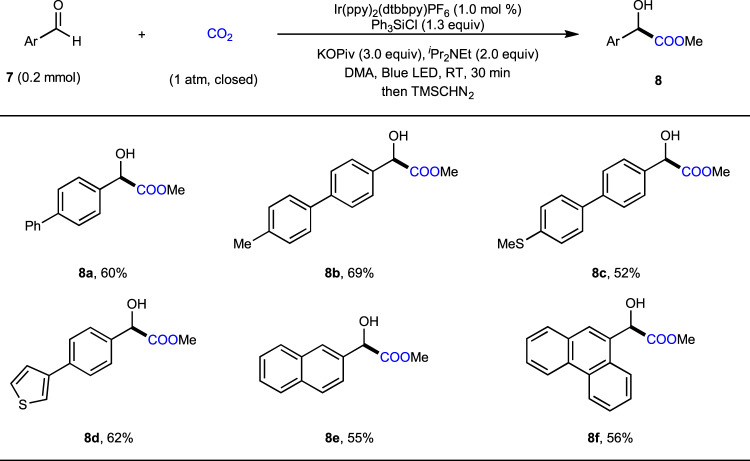
Substrate scope of aryl aldehydes. Isolated yields of corresponding methyl esters are shown.

After demonstrating the viability of different kinds of ketones, we turned our attention to more challenging aryl aldehydes. Although umpolung carboxylations of aryl aldehydes with CO_2_ are rare^43,68,69^, α-hydroxycarboxylic acids synthesis typically suffer from low yields, low selectivity, and limited scope. When aryl aldehydes were applied as substrates to the standard reaction conditions, we observed a complicated mixture of products due to intense side reactions, including pinacol coupling and disproportionation, which might arise from the lower steric hindrance of aldehydes and the strong base. In order to prevent such side reactions, we further tested several bulky chlorosilanes and weak bases. After systematic screening, we were pleased to find a suitable option using potassium pivalate (KOPiv) as base and Ph_3_SiCl as additive (Fig. 7). Under these modified reaction conditions, umpolung carboxylation of **7a** took place smoothly to afford, after esterification, the desired product **8a** in 60% yield along with 33% of pinacol **8a′**. In contrast, **8a** could only be obtained in 8% yield along with 63% of **8a′** in the absence of Ph_3_SiCl, which indicates the significance of Ph_3_SiCl to the carboxylation of aryl aldehydes. Several aryl aldehydes bearing different substituents, including thiol ether (**7c**) and thiophene (**7d**), reacted to give the desired α-hydroxycarboxylic esters in 30 min with moderate to good yields. Moreover, fused aryl aldehydes, such as 2-naphthaldehyde (**7e**) and phenanthrene-9-carboxaldehyde (**7f**), also reacted well.

**Fig. 8:**
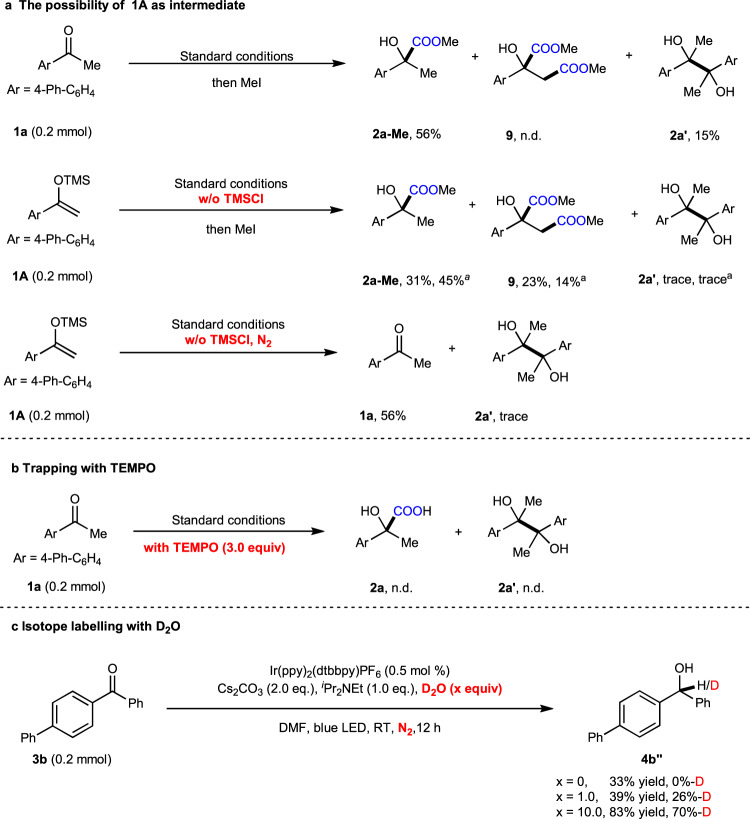
Control experiments. Isolated yields are shown. **a** Investigation of the possibility with sily enol ether **1A** as intermediate. **b** Investigation of the effect of radical scavenger in the reaction. **c** Investigation of the effect of D_2_O in the reaction. ^a^KO^*t*^Bu (1.0 equiv) was used.

To gain more mechanistic insight into this transformation, we conducted several control experiments. As silyl enol ether **1A** can be prepared easily from ketone **1a** and TMSCl in the presence of base, we wondered whether it might act as the reaction intermediate. However, we failed to detect **1A** at different stages of the standard reaction of **1a**. To further confirm the assumption, we synthesized **1A** and then applied it as the starting material for the carboxylation in the absence of TMSCl (Fig. 8a). We found that a lower yield of the desired product **2a-Me** was generated along with the dimethyl 2-hydroxy-2-phenylsuccinate **9**, which was never detected in the standard reaction of **1a**, as a major byproduct. We hypothesized that the formation of **9** might involve the successive single electron transfer (SSET) reduction and carboxylation processes of the silyl enol ether **1A** with CO_2_^70^. Moreover, we observed the full decomposition of **1A** under a N_2_ atmosphere to give **1a** in 53% yield, indicating that the formation of **2a** from **1A** and CO_2_ might proceed with **1a** as the real reactant. Meanwhile, we observed that the reduction potential of **1A** was slightly higher than **1a** (see below). Therefore, we excluded the possibility that silyl enol ether **1A** is the key intermediate in the observed umpolung carboxylation. Moreover, alcohol **2a**″ and TMS-protected **2a**″ were treated with standard conditions, **2a** was not observed, **2a**″ and some dehydroxylative byproducts were detected, which rule out the direct C–H carboxylation pathway (please see the SI for details).

To further explore other intermediates in the reaction, we added a radical scavenger, 2,2,6,6-tetramethylpiperidinooxy (TEMPO), to the reaction of **1a** under the standard conditions (Fig. 8b). In the presence of TEMPO, the reaction was completely suppressed, indicating that radicals might be involved in this reaction. Furthermore, we tested the effect of D_2_O on the carboxylation reaction. The generation of **2a**″ from **1a** was inhibited by the addition of D_2_O, thus precluding the determination of deuterium incorporation (please see the SI for details). The reaction of **3b** in the presence of different amounts of D_2_O as additive, however, did provide alcohol **4b**″ in up to 83% yield and 70% deuterium incorporation (Fig. 8c), suggesting that the benzylic carbanion is a possible intermediate.

**Fig. 9:**
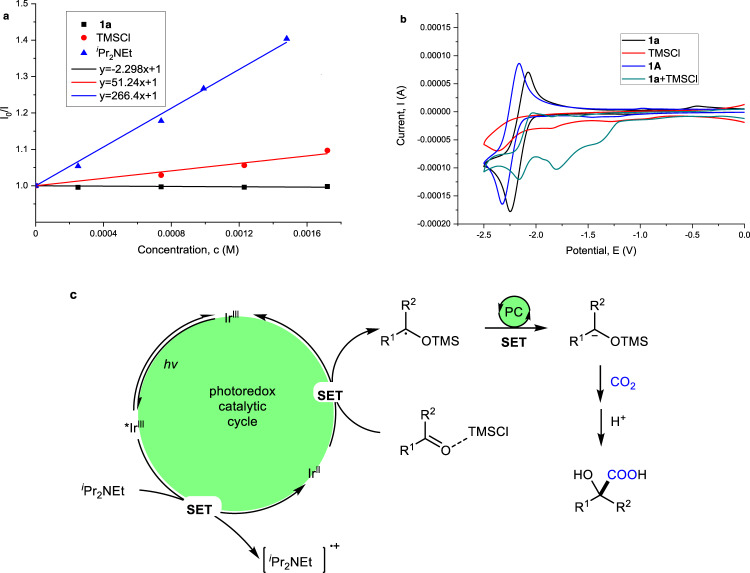
Mechanistic experiments and proposed mechanism. **a** Stern-Volmer experiment. **b** Cyclic voltammetry (CV) tests. **c** Proposed mechanism.

To demonstrate that the activation of carbonyl groups by a Lewis acidic chlorosilane is a critical part of the reaction, we performed Stern–Volmer luminescence studies and electrochemistry investigations (please see the SI for details). The luminescence of Ir(ppy)_2_(dtbbpy)PF_6_ at *λ*_max_ = 570 nm was readily quenched by ^*i*^Pr_2_NEt with a slope of 266.4 (Fig. 9a), which is much more significant than ketone **1a** (−2.3) and TMSCl (51.2). These results suggest that the reaction proceeds with reductive quenching to give reduced Ir^II^-catalyst (*E*_1/2_^Red^[Ir^III^/Ir^II^] = −1.51 V vs SCE)^71^. Moreover, the cyclic voltammetry (CV) test (Fig. 9b) indicated that the presence of TMSCl promoted reduction of the carbonyl by lowering the reductive potential. Thus, we believe that the SET reduction of the TMSCl-activated ketone **1a** is favored to give the corresponding α-silyloxyl benzyl radical^72,73^, which might be further reduced by the Ir^II^-catalyst to generate a benzylic carbon anion. The following nucleophilic attack to CO_2_ would give the corresponding products (Fig. 9c).

In order to test other alternative pathways, such as coupling between benzyl radicals and a CO_2_ radical anion, we further detected the formation of formate from CO_2_. Although a turnover number (TON) of 43 for the generation of formate was observed in the absence of ketones, the reduction of CO_2_ was significantly depressed under the standard reaction of **1a** (please see the SI for details). Meanwhile, the standard reduction potential of CO_2_ in dry DMF is −2.21 V vs SCE^74^. The reduction potentials of **1a**, **1k**, and **7a** in DMF are −1.81 V vs SCE, −2.1 V vs SCE, and −1.76 V vs SCE (please see the SI for details). Moreover, the reduction potential of α-ketoesters (for methyl 3-methyl-2-oxobutanoate, *E*_1/2_^red^ = −1.75 V vs SCE^75^) is also lower than CO_2_. These results indicated that the selective reduction of ketones over CO_2_.

**Fig. 10:**
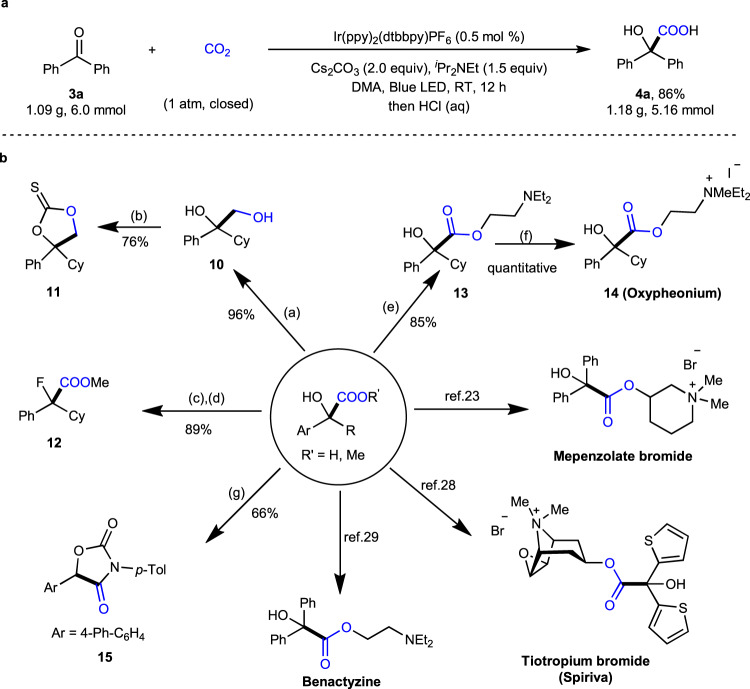
Synthetic applications. **a** Gram-scale reaction. **b** Diverse functionalizations of products. (a) LiAlH_4_, THF, reflux. (b) TCDI, DCM, RT. (c) TMSCHN_2_, MeOH/Et_2_O; (d) Deoxofluor, DCM, RT, N_2_. (e) diethylamine, Na_2_CO_3_, DCE. (f) MeI, CH_3_CN, 65 °C. (g) *p*-Me-C_6_H_4_NCO, DMAP, THF, RT − 50 °C. TCDI = 1,1′-Thiocarbonyldiimidazole. DCM dichloromethane, Deoxofluor Bis(2-methoxyethyl)aminosulfur trifluoride, DMAP *N*,*N*-dimethylpyridin-4-amine, THF tetrahydrofuran.

With this methodology in hand, we further demonstrated its utility by testing the gram-scale reaction and the synthesis of valuable intermediates and bioactive compounds (Fig. 10). First of all, we were delighted to find that the gram-scale reaction of **3a** (6.0 mmol) proceeded well with comparable yield and efficiency (Fig. 10a). Second, the product **2i** could be reduced by LiAlH_4_ to give diol **10**, which underwent cyclization to generate 1,3-dioxolane-2-thione **11** in a good yield (Fig. 10b)^76^. The esterification of **2i** and the following fluorination of the C–O bond generated α-fluorocarboxylic ester **12**^77^. Importantly, **2i** could be transformed easily to bioactive oxypheonium **14**. In addition, the cyclization of **8a** with isocyanate could give oxazolidine-2,4-dione **15**^78^. Moreover, as shown in Fig. 1, the product **4a** could be readily converted into valuable anticholinergic drugs trospium chloride (Sanctura)^24,27^, mepenzolate bromide (Cantil)^23^, and benactyzine^29^. The α-hydroxycarboxylic acid **4r** is also a synthetic precursor for the drugs tiotropium bromide (Spiriva) and aclidinium bromide (Tudorza)^28^.

## Discussion

In this work, we developed a strategy for the visible-light photoredox-catalyzed umpolung carboxylation of carbonyl groups with CO_2_ by using Lewis acidic chlorosilanes as activating/protecting groups. This strategy is general and practical for the carboxylation of diverse carbonyl compounds, including alkyl aryl ketones, diaryl ketones, α-ketoamides, α-ketoesters, and aryl aldehydes, to give valuable α-hydroxycarboxylic acids. The utility of this method is highlighted by facile generation of key intermediates to produce many drugs and natural products, including oxypheonium, mepenzolate bromide, benactyzine, and tiotropium. Moreover, this transformation features low catalyst loading, high selectivity, broad substrate scope, good functional group tolerance, mild reaction conditions (room temperature, 1 atm), and facile gram-scale reaction. The control experiments demonstrated the important role of chlorosilanes to promote the reaction and excluded the possibility of silyl enol ethers serving as key intermediates. Finally, carbon radicals and carbanions might be involved in this transformation.

## Methods

### Synthesis of **2a**–**2l** and **6a**–**6k**

The oven-dried Schlenk tube (10 mL) containing a stirring bar was charged with ketone (0.2 mmol), Ir(ppy)_2_(dtbbpy)PF_6_ (1.9 mg, 1 mol %), or 3DPA2FBN (2.6 mg, 0.004 mmol), then added ^*t*^BuOK (44.9 or 60.9 mg, 0.4 or 0.5 mmol) in glovebox. The tube was taken out, evacuated, and back-filled with CO_2_ for three times. Subsequently, ^*i*^Pr_2_NEt (66 μL, 0.4 mmol), TMSCl (34 μL, 0.26 mmol), and DMA (2 or 4 mL) were added via syringe under CO_2_ atmosphere. Once added, the Schlenk tube was sealed at atmospheric pressure of CO_2_ (1 atm). The reaction was stirred in water bath and irradiated with a 30 W blue LED lamp (3 cm away, with cooling fan to keep the reaction temperature at 25–30 °C) for 12 h. After completion, 0.5 mL ^*n*^Bu_4_NF (1.0 N in THF) was carefully added to quench the reaction, the mixture was allowed to stir for 30 min at room temperature. Then, Work-up 1: the mixture was quenched with HCl (2 N), extracted with EtOAc, the combined organic phases were concentrated in vacuo. The residue was purified by silica gel flash chromatography (0.1% AcOH in petroleum ether/EtOAc) to give the corresponding α-hydroxycarboxylic acids as products. Or Work-up 2: MeI (37 μL, 3.0 equiv) was added via syringe. The resulting mixture was further stirred for 3 h at 60 °C. After cooling to room temperature, the mixture was quenched with HCl (2 N), extracted with EtOAc, the combined organic phases were concentrated in vacuo. The residue was purified by silica gel flash column chromatography (petroleum ether/EtOAc 100/1-50/1) to give the pure desired methyl α-hydroxycarboxylates.

### Synthesis of **4a**–**4r**

The oven-dried Schlenk tube (10 mL) containing a stirring bar was charged with ketone (0.2 mmol), Ir(ppy)_2_(dtbbpy)PF_6_ (1.0 mg, 0.001 mmol, 0.5 mol %), then added Cs_2_CO_3_ (130.0 mg, 0.4 mmol) in glovebox. The tube was taken out, evacuated, and back-filled with CO_2_ for three times. Subsequently, ^*i*^Pr_2_NEt (33 or 50 μL, 0.2 or 0.3 mmol), DMF (2 mL) was added via syringe under CO_2_ atmosphere. Once added, the Schlenk tube was sealed at atmospheric pressure of CO_2_ (1 atm.). Then the work-up procedures were the same as above mentioned.

### Synthesis of **8a**–**8f**

The oven-dried Schlenk tube (25 mL) containing a stirring bar was charged with aldehyde (0.2 mmol), Ir(ppy)_2_(dtbbpy)PF_6_ (1.9 mg, 0.002 mmol, 1 mol %), Ph_3_SiCl (76.7 mg, 0.26 mmol), and PivOK (84.1 mg, 0.6 mmol) in glovebox. The tube was taken out, evacuated, and back-filled with CO_2_ for three times. Subsequently, ^*i*^Pr_2_NEt (66 μL, 0.4 mmol) and DMA (6 mL) were added via syringe under CO_2_ atmosphere. Once added, the Schlenk tube was sealed at atmospheric pressure of CO_2_ (1 atm.). After completion, 0.5 mL ^*n*^Bu_4_NF (1.0 N in THF) was carefully added to quench the reaction, the mixture was quenched with HCl (2 N), extracted with EtOAc, the combined organic phases were concentrated in vacuo. The crude residue was dissolved in 4 mL MeOH/Et_2_O (1/3), TMSCHN_2_ (0.3 mL, 0.6 mmol, 2 M in hexanes) was added drop wisely at 0 °C. Upon completion. After removal of the solvent, the crude reaction mixture was purified on silica gel (petroleum ether and ethyl acetate) to afford the desired methyl α-hydroxycarboxylates.

## Supplementary information

Supplementary Information

## Data Availability

The authors declare that the data supporting the findings of this study are available within the article and its Supplementary Information files. Extra data are available from the author upon request.

## References

[1] Seebach D (1979). Methods of reactivity umpolung. Angew. Chem. Int. Ed. Engl..

[2] Lee KN, Ngai M-Y (2017). Recent developments in transition-metal photoredox-catalysed reactions of carbonyl derivatives. Chem. Commun..

[3] Xia Q, Dong J, Song H, Wang Q (2019). Visible-light photocatalysis of the ketyl radical coupling reaction. Chem. Eur. J..

[4] Szostak M, Fazakerley NJ, Parmar D, Procter DJ (2014). Cross-coupling reactions using samarium(II) iodide. Chem. Rev..

[5] Gentry EC, Knowles RR (2016). Synthetic applications of proton-coupled electron transfer. Acc. Chem. Res..

[6] Tarantino KT, Liu P, Knowles RR (2013). Catalytic ketyl-olefin cyclizations enabled by proton-coupled electron transfer. J. Am. Chem. Soc..

[7] Qi L, Chen Y (2016). Polarity-reversed allylations of aldehydes, ketones, and imines enabled by Hantzsch ester in photoredox catalysis. Angew. Chem. Int. Ed..

[8] Fava E, Nakajima M, Nguyen ALP, Rueping M (2016). Photoredox-catalyzed ketyl–olefin coupling for the synthesis of substituted chromanols. J. Org. Chem..

[9] Lee KN, Lei Z, Ngai M-Y (2017). β-Selective reductive coupling of alkenylpyridines with aldehydes and imines via synergistic lewis acid/photoredox catalysis. J. Am. Chem. Soc..

[10] Wang Z-S (2020). Ynamide smiles rearrangement triggered by visible-light-mediated regioselective ketyl–ynamide coupling: rapid access to functionalized indoles and isoquinolines. J. Am. Chem. Soc..

[11] Nakajima M, Fava E, Loescher S, Jiang Z, Rueping M (2015). Photoredox-catalyzed reductive coupling of aldehydes. Ketones, Imines Visible Light. Angew. Chem. Int. Ed..

[12] Rono LJ, Yayla HG, Wang DY, Armstrong MF, Knowles RR (2013). Enantioselective photoredox catalysis enabled by proton-coupled electron transfer: development of an asymmetric aza-pinacol cyclization. J. Am. Chem. Soc..

[13] Fava E, Millet A, Nakajima M, Loescher S, Rueping M (2016). Reductive umpolung of carbonyl derivatives with visible-light photoredox catalysis: direct access to vicinal diamines and amino alcohols via α-amino radicals and ketyl radicals. Angew. Chem. Int. Ed..

[14] Li W, Duan Y, Zhang M, Cheng J, Zhu C (2016). A photoredox catalyzed radical–radical coupling reaction: facile access to multi-substituted nitrogen heterocycles. Chem. Commun..

[15] Chen M, Zhao X, Yang C, Xia W (2017). Visible-light-triggered directly reductive arylation of carbonyl/iminyl derivatives through photocatalytic PCET. Org. Lett..

[16] Xia Q (2018). N-Arylamines coupled with aldehydes, ketones, and imines by means of photocatalytic proton-coupled electron transfer. Chem. Eur. J..

[17] Lin L (2017). Organocatalytic enantioselective protonation for photoreduction of activated ketones and ketimines induced by visible light. Angew. Chem. Int. Ed..

[18] Masashi K, Hirofumi A, Yuji W, Shozo Y (1992). Visible-light induced photofixation of CO_2_ into benzophenone catalyzed by colloidal CdS microcrystallites. Chem. Lett..

[19] Tomoyuki O, Kunizo H, Shinjiro M, Yuji W, Shozo Y (1993). Visible-light induced photocatalytic fixation of CO_2_ into benzophenone using poly(p-phenylene) as a photocatalyst. Chem. Lett..

[20] Scialdone O (2007). Electrocarboxylation of aromatic ketones: influence of operative parameters on the competition between ketyl and ring carboxylation. J. Electroanal. Chem..

[21] Thompson IM, Lauvetz R (1976). Oxybutynin in bladder spasm, neurogenic bladder, and enuresis. Urology.

[22] Middleton B, Cacciaguerra F, White D (1987). Cyclandelate: an inhibitor of cholesterol esterification. Drugs.

[23] Yamashita Y (2014). Synthesis and biological comparison of enantiomers of mepenzolate bromide, a muscarinic receptor antagonist with bronchodilatory and anti-inflammatory activities. Bioorg. Med. Chem..

[24] Rovner ES (2004). Trospium chloride in the management of overactive bladder. Drugs.

[25] Jang J-H, Kanoh K, Adachi K, Shizuri Y (2006). Awajanomycin, a cytotoxic γ-lactone-δ-lactam metabolite from marine-derived *Acremonium* sp. AWA16-1. J. Nat. Prod..

[26] Reddy DS, Shibata N, Nagai J, Nakamura S, Toru T (2009). A dynamic kinetic asymmetric transformation in the α-hydroxylation of racemic malonates and its application to biologically active. Molecules. Angew. Chem. Int. Ed..

[27] Krebs, A., Schäfer, B. & Kochner, A. Method for producing azoniaspironortropine esters and nortropan-3-one compounds. US2010/48903, A1 (2010).

[28] Xiang Z, Liu J, Sun H, Wen X (2017). Discovery of novel potent muscarinic M3 receptor antagonists with proper plasma stability by structural recombination of marketed M3 antagonists. ChemMedChem.

[29] da Silva AF, Afonso MAS, Cormanich RA, Jurberg ID (2020). Room temperature coupling of aryldiazoacetates with boronic acids enhanced by blue light irradiation. Chem. Eur. J..

[30] Coppola, G. M. & Schuster, H. F. *a-Hydroxy Acids in Enantioselective Syntheses* (Wiley-VCH, 1997).

[31] Liu Q, Wu L, Jackstell R, Beller M (2015). Using carbon dioxide as a building block in organic synthesis. Nat. Commun..

[32] Luo J, Larrosa I (2017). C−H carboxylation of aromatic compounds through CO_2_ fixation. ChemSusChem.

[33] Tortajada A, Juliá-Hernández F, Börjesson M, Moragas T, Martin R (2018). Transition-metal-catalyzed carboxylation reactions with carbon dioxide. Angew. Chem. Int. Ed..

[34] Grignard B, Gennen S, Jérôme C, Kleij AW, Detrembleur C (2019). Advances in the use of CO_2_ as a renewable feedstock for the synthesis of polymers. Chem. Soc. Rev..

[35] Zhang L, Li Z, Takimoto M, Hou Z (2020). Carboxylation reactions with carbon dioxide using N-heterocyclic carbene-copper catalysts. Chem. Rec..

[36] Song L (2020). CO_2_ = CO + [O]: recent advances in carbonylation of C–H bonds with CO_2_. Chem. Commun..

[37] Cao Y, He X, Wang N, Li H-R, He L-N (2018). Photochemical and electrochemical carbon dioxide utilization with organic compounds. Chin. J. Chem..

[38] Huang K, Sun C-L, Shi Z-J (2011). Transition-metal-catalyzed C–C bond formation through the fixation of carbon dioxide. Chem. Soc. Rev..

[39] Hong J, Li M, Zhang J, Sun B, Mo F (2019). C−H bond carboxylation with carbon dioxide. ChemSusChem.

[40] Liu R (2012). Silicon nanowires as photoelectrodes for carbon dioxide fixation. Angew. Chem. Int. Ed..

[41] Wang H, Xu X-M, Lan Y-C, Wang H-M, Lu J-X (2014). Electrocarboxylation of haloacetophenones at silver electrode. Tetrahedron.

[42] Senboku H, Sakai K, Fukui A, Sato Y, Yamauchi Y (2019). Efficient synthesis of mandel acetates by electrochemical carboxylation of benzal diacetates. ChemElectroChem.

[43] Masada K, Kusumoto S, Nozaki K (2020). Reductive coupling of carbon dioxide and an aldehyde mediated by a copper(I) complex toward the synthesis of α-hydroxycarboxylic acids. Org. Lett..

[44] Yeung CS (2019). Photoredox catalysis as a strategy for CO_2_ incorporation: direct access to carboxylic acids from a renewable feedstock. Angew. Chem. Int. Ed..

[45] Ye J-H, Ju T, Huang H, Liao L-L, Yu D-G (2021). Radical carboxylative cyclizations and carboxylations with CO_2_. Acc. Chem. Res..

[46] He X, Qiu L-Q, Wang W-J, Chen K-H, He L-N (2020). Photocarboxylation with CO_2_: an appealing and sustainable strategy for CO_2_ fixation. Green. Chem..

[47] Pradhan S, Roy S, Sahoo B, Chatterjee I (2021). Utilization of CO_2_ feedstock for organic synthesis by visible‐light photoredox catalysis. I. Chem. Eur. J..

[48] Cai, B., Cheo, H. W., Liu, T. & Wu, J. Light-promoted organic transformations utilizing carbon-based gas molecules as feedstocks. *Angew. Chem. Int. Ed.*10.1002/anie.202010710 (2021). 10.1002/anie.202010710 33002315

[49] Murata K, Numasawa N, Shimomaki K, Takaya J, Iwasawa N (2017). Construction of a visible light-driven hydrocarboxylation cycle of alkenes by the combined use of Rh(I) and photoredox catalysts. Chem. Commun..

[50] Shimomaki K, Murata K, Martin R, Iwasawa N (2017). Visible-light-driven carboxylation of aryl halides by the combined use of palladium and photoredox catalysts. J. Am. Chem. Soc..

[51] Meng Q-Y, Wang S, König B (2017). Carboxylation of aromatic and aliphatic bromides and triflates with CO_2_ by dual visible-light–nickel catalysis. Angew. Chem. Int. Ed..

[52] Meng Q-Y, Wang S, Huff GS, König B (2018). Ligand-controlled regioselective hydrocarboxylation of styrenes with CO_2_ by combining visible light and nickel catalysis. J. Am. Chem. Soc..

[53] Hou J (2018). Visible-light-driven alkyne hydro-/carbocarboxylation using CO_2_ via iridium/cobalt dual catalysis for divergent heterocycle synthesis. J. Am. Chem. Soc..

[54] Ju T (2018). Selective and catalytic hydrocarboxylation of enamides and imines with CO_2_ to generate α,α-disubstituted α-amino acids. Angew. Chem. Int. Ed..

[55] Fan X, Gong X, Ma M, Wang R, Walsh PJ (2018). Visible light-promoted CO_2_ fixation with imines to synthesize diaryl α-amino acids. Nat. Commun..

[56] Liao L-L (2018). Visible-light-driven external-reductant-free cross-electrophile couplings of tetraalkyl ammonium salts. J. Am. Chem. Soc..

[57] Wang H, Gao Y, Zhou C, Li G (2020). Visible-light-driven reductive carboarylation of styrenes with CO_2_ and aryl halides. J. Am. Chem. Soc..

[58] Zhou W-J (2020). Reductive dearomative arylcarboxylation of indoles with CO_2_ via visible-light photoredox catalysis. Nat. Commun..

[59] Huang H (2020). Visible-light-driven anti-markovnikov hydrocarboxylation of acrylates and styrenes with CO_2_. CCS Chem..

[60] Ishitani O, Yanagida S, Takamuku S, Pac C (1987). Redox-photosensitized reactions. 13. Ru(bpy)32+-photosensitized reactions of an NADH model, 1-benzyl-1,4-dihydronicotinamide, with aromatic carbonyl compounds and comparison with thermal reactions. J. Org. Chem..

[61] Isse AA, Gennaro A (2003). Mechanism of the electrochemical carboxylation of aromatic ketones in dimethylformamide. Collect. Czech. Chem. Commun..

[62] Lu Y (2020). Nanostructured electrocatalysts for electrochemical carboxylation with CO_2_. Nano Sel..

[63] Zhang W-Z (2014). Sequential carboxylation/intramolecular cyclization reaction of o-alkynyl acetophenone with CO_2_. Org. Chem. Front..

[64] Mahrwald, R. (ed). *Modern Aldol Reactions* (Wiley-VCH, 2004).

[65] Geissman TA (1944). Cannizzaro reaction. Org. React..

[66] Speckmeier E, Fischer TG, Zeitler K (2018). A toolbox approach to construct broadly applicable metal-free catalysts for photoredox chemistry: deliberate tuning of redox potentials and importance of halogens in donor-acceptor cyanoarenes. J. Am. Chem. Soc..

[67] Masuda Y, Ishida N, Murakami M (2015). Light-driven carboxylation of o-alkylphenyl ketones with CO_2_. J. Am. Chem. Soc..

[68] Juhl M, Lee J-W (2018). Umpolung reactivity of aldehydes toward carbon dioxide. Angew. Chem. Int. Ed..

[69] Juhla M, Kimb MJ, Lee H-Y, Baik M-H, Lee J-W (2019). Aldehyde carboxylation: a concise DFT mechanistic study and a hypothetical role of CO_2_ in the origin of life. Synlett.

[70] Ju T (2021). Dicarboxylation of alkenes, allenes and (hetero)arenes with CO_2_ via visible-light photoredox catalysis. Nat. Catal.

[71] Lowry MS (2005). Single-layer electroluminescent devices and photoinduced hydrogen production from an ionic iridium(III) complex. Chem. Mater..

[72] Kise N, Agui S, Morimoto S, Ueda N (2005). Electroreductive acylation of aromatic ketones with acylimidazoles. J. Org. Chem..

[73] Yan M, Kawamata Y, Baran PS (2017). Synthetic organic electrochemical methods since 2000: on the verge of a renaissance. Chem. Rev..

[74] Lamy E, Nadjo L, Saveant JM (1977). Standard potential and kinetic parameters of the electrochemical reduction of carbon dioxide in dimethylformamide. J. Electroanal. Chem..

[75] Zhu J, Yuan Y, Wang S, Yao Z-J (2017). Synthesis of 2,3-dialkylated tartaric acid esters via visible light photoredox-catalyzed reductive dimerization of α-ketoesters. ACS Omega.

[76] Song L (2020). Visible‐light photoredox‐catalyzed remote difunctionalizing carboxylation of unactivated alkenes with CO_2_. Angew. Chem. Int. Ed..

[77] Koyanagi J, Kamei T, Ishizaki M, Nakamura H, Takahashi T (2014). Improved synthetic route to methyl 1-fluoroindan-1-carboxylate (FICA Me ester) and 4-methyl derivatives. Chem. Pharm. Bull..

[78] Ichiyanagi T, Yamasaki R (2005). Anomeric O-acylation of Kdo using alkyl and aryl isocyanates. Carbohydr. Res..

