# Supplementary material for: Visible-light photoredox-catalyzed umpolung carboxylation of carbonyl compounds with CO2
Source: Nat Commun. 2021 Jun 3;12:3306. doi: 10.1038/s41467-021-23447-8 (PMC8175691; doi:10.1038/s41467-021-23447-8)
Supplement: Supplementary file 1 — Supplementary Information [file 41467_2021_23447_MOESM1_ESM.pdf]

Supplementary Information
for

Visible-light photoredox-catalyzed Umpolung Carboxylation of Carbonyl Compounds with CO_2

G.M.Cao et al.

Supplementary Methods

1. General Information

All reactions were set up using standard Schlenk techniques and carried out under a carbon dioxide atmosphere with anhydrous solvents, unless otherwise noted. Commercially available chemicals were obtained from Adamas-beta, Acros Organics, Aldrich Chemical Co., Alfa Aesar, ABCR, TCI and used as received unless otherwise stated. Reactions were monitored by thin-layer chromatography (TLC) carried out on 0.2±0.03 mm using UV light as a visualizing agent and bromocresol green in EtOH as developing agents. ^{1}H, ^{13}C, and ^{19}F NMR spectra were recorded on a Brüker Advance 400 spectrometer (^{1}H: 400 MHz, ^{13}C: 101 MHz and ^{19}F NMR :376 MHz). Chemical shifts (δ) for ^{1}H, ^{13}C, and ^{19}F NMR spectra are given in ppm relative to TMS. The residual solvent signals were used as references for ^{1}H and ^{13}C NMR spectra and the chemical shifts converted to the TMS scale ($CDCl_3$): δH = 7.26 ppm, δC = 77.16 ppm; $(CD_3)_2SO$: δH = 2.50 ppm, δC = 39.52 ppm). The following abbreviations were used to explain the multiplicities: s = singlet, d = doublet, t = triplet, q = quartet, m = multiplet, b = broad. High-resolution mass spectra (HRMS) were recorded on a Bruker Daltonics MicroTOF-Q. GC-MS was obtained using electron ionization (Agilent Technologies 7890B/GCSystem and 5977A/MSD). LRMS was obtained using Thermo-Fisher LTQ-ESI-MS. Fluorescence quenching experiments were measured on a RF-5301PC Spectrofluorophotometer. Electrochemical studies were carried out with a CHI600E electrochemical workstation. UPLC yields were recorded on Waters ACQUITY UPLC M-Class. TLC was performed using commercially prepared 100-400 mesh silica gel plates (GF254), and visualization was effected at 254 nm. Visible light irradiation was performed with a 30 W LED Light at λ_{ir} = 450 ± 10 nm for photocatalytic reactions. Detected side products in this reaction were pinacols and alcohol. Pinacols **2a”**, **2f’**, **2h’**, **6c’**, **8a’**, **8b’**, **8d’**, **8e’**, **8f’** for substrates **1a**, **1f**, **1h**, **5c**, **7a**, **7b**, **7d**, **7e**, **7f** was isolated in 20%, 25%, 13, 12%, 33%, 27%, 32%, 20%, 12% yield. **2d** was obtained in 38% along with 61% isolated yield of **1d**. alcohol **6a” for** substrate **5a** was isolated in 18% yield. As for ketoamides substrates **5g**, **5i** and **5j** and sterically hindered carbonyl compounds (eg. **1b**, **1d**, **1e**, **1i**), corresponding alcohols were detected by GC-MS. As for compound containing chloride **3m**, trace amount of dechloroprotonative product was detected by GC-MS. As for the product **4p**, **4q** and **4r**, corresponding acids were unstable.

2. The synthesis of substrates

The synthesis of α-ketoesters and α-ketoamides used in this work were prepared according to the methods reported in literature[1,2,3]. C-silyl compound **1B** used in this work was prepared according to the methods reported in literature[4,5]. O-silyl compound **1C** used in this work was prepared according to the methods reported in literature[6].

3. The synthesis of α-hydroxycarboxylic acids.

General procedure for the carboxylation of alkyl aryl ketones, α-ketoamides and α-ketoesters (1a-1l, 5a-5k)

The oven-dried Schlenk tube (10 mL) containing a stirring bar was charged with ketone (0.2 mmol, 1.0 equiv), $Ir(ppy)_2(dtbbpy)PF_6$ (1.9 mg, 0.002 mmol, 1 mol %) or 3DPA2FBN (2.6 mg, 0.004 mmol), then added ^{t}BuOK (44.9 mg or 60.9 mg, 0.4 mmol or 0.5 mmol, 2.0 equiv or 2.5 equiv) in glovebox. The tube was taken out, evacuated and back-filled with CO_2 for 3 times. Subsequently, $^i Pr_2NEt$ (66 μL, 0.4 mmol, 2.0 equiv), TMSCl (34 μL, 0.26 mmol, 1.3 equiv), DMA (2 mL or 4 mL) was added via syringe under CO_2 atmosphere. Once added, the Schlenk tube was sealed at atmospheric pressure of CO_2 (1 atm). The reaction was stirred in water bath and irradiated with a 30 W blue LED lamp (3 cm away, with cooling fan to keep the reaction temperature at 25~30 °C) for 12 h. After completion, 0.5 mL $^n Bu_4NF$ (1.0 N in THF) was carefully added to quench the reaction, the mixture was allowed to stir for 30 min at room temperature.

Work-up1: The reaction was quenched by 2 or 2.5 mL HCl (2 N), stirred for 10 min, and diluted with 2.5 mL EtOAc. The reaction mixture was extracted by EtOAc and the combined organic phases were concentrated *in vacuo*. The residue was purified by silica gel flash column chromatography (petroleum ether/EtOAc/AcOH 10/1/0.1%-10/1/0.5%) to give the pure desired product.

Work-up 2: MeI (37 μL, 3.0 equiv) was added via syringe and the reaction tube was sealed. The resulting mixture was further stirred for 3 h at 60 °C. After cooling to room temperature, the resulting mixture was quenched by 2 mL HCl (2 N), stirred for 10 min, and diluted with 2.5 mL EtOAc. The reaction mixture was extracted by EtOAc and the combined organic phases were concentrated *in vacuo*. The residue was purified by silica gel flash column chromatography (petroleum ether/ EtOAc 100/1-50/1) to give the pure desired product.

General procedure for the carboxylation of diaryl ketones (3a-3r)

The oven-dried Schlenk tube (10 mL) containing a stirring bar was charged with ketone (0.2 mmol, 1.0 equiv), $Ir(ppy)_2(dtbbpy)PF_6$ (1.0 mg, 0.001 mmol, 0.5 mol %), then added Cs_2CO_3 (130.0 mg, 0.4 mmol, 2.0 equiv) in glovebox. The tube was taken out, evacuated and back-filled with CO_2 for 3 times. Subsequently, $^iPr_2NEt$ (33 μL or 50 μL, 0.2 mmol or 0.3 mmol, 1.0 equiv or 1.5 equiv), DMF (2 mL) was added via syringe under CO_2 atmosphere. Once added, the Schlenk tube was sealed at atmospheric pressure of CO_2 (1 atm.). The reaction was stirred in water bath and irradiated with a 30 W blue LED lamp (3 cm away, with cooling fan to keep the reaction temperature at 25~30 °C) for 12 h. The work-up procedures were same as above mentioned.

General procedure for the carboxylation of aldehydes (7a-7f)

The oven-dried Schlenk tube (25 mL) containing a stirring bar was charged with aldehyde (0.2 mmol, 1.0 equiv), $Ir(ppy)_2(dtbbpy)PF_6$ (1.9 mg, 0.002 mmol, 1 mol %), Ph_3SiCl (76.7 mg, 0.26 mmol, 1.3 eq.), PivOK (84.1 mg, 0.6 mmol, 3.0 equiv) in glovebox. The tube was taken out, evacuated and back-filled with CO_2 for 3 times. Subsequently, $^iPr_2NEt$ (66 μL, 0.4 mmol, 2.0 equiv), DMA (6 mL) was added via syringe under CO_2 atmosphere. Once added, the Schlenk tube was sealed at atmospheric pressure of CO_2 (1 atm). The reaction was stirred in water bath and irradiated with a 30 W blue LED lamp (3 cm away, with cooling fan to keep the reaction temperature at 25~30 °C) for 30 min. The work-up procedures were same as above mentioned. After completion, 0.5 mL $^nBu_4NF$ (1.0 N in THF) was carefully added to quench the reaction, the mixture was allowed to stir for 30 min at room temperature, quenched by 3 mL HCl (2N), stirred for 10 min, and diluted with 10 mL EtOAc. The reaction mixture was extracted by EtOAc (10 mL x 2) and the combined organic phases were concentrated *in vacuo*. 30 mL EtOAc was added to combined organic phases, then washed by water (5 mL x 2), the combined organic phases were concentrated *in vacuo*. The crude residue was dissolved in 4 mL $MeOH/Et_2O$ (1/3), $TMSCHN_2$ (0.3 mL, 0.6 mmol, 2 M in hexanes) was added drop wisely at 0 °C. The mixture was stirred at ambient temperature until the completion of the methylation reaction. All the volatile materials were removed using a rotary evaporator under reduced pressure, and the product was purified by flash chromatography on silica gel using ethyl acetate and petroleum ether as eluents.

2-([1,1'-biphenyl]-4-yl)-2-hydroxypropanoic acid (2a)

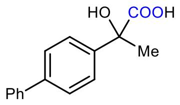

38.8 mg, 80% yield; White solid;

R_f = 0.33 (DCM/MeOH 10/1);

Mp: 115-117 °C;

^{1}H NMR (400 MHz, DMSO-d_6) δ 7.81 – 7.50 (m, 6H), 7.46 – 7.38 (m, 2H), 7.37 – 7.27 (m, 1H), 1.63 (s, 3H). **^{13}C NMR** (101 MHz, DMSO-d_6) δ 176.46, 143.99, 140.30, 139.37, 129.34, 127.81, 127.06, 126.65, 126.26, 75.20, 27.74. **HRMS (ESI$^-$)**: calcd for $C_{15}H_{13}O_3^-$ [M-H]$^-$: 241.0870, found 241.0874.

2-([1,1'-biphenyl]-4-yl)-2-hydroxybutanoic acid (2b)

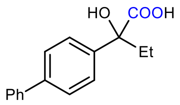

41.0 mg, 80% yield; White solid;

R_f = 0.47 (DCM/MeOH 10/1);

Mp: 138-140 °C;

^{1}H NMR (400 MHz, DMSO-d_6) δ 7.70 – 7.58 (m, 6H), 7.48 – 7.44 (m, 2H), 7.41 – 7.31 (m, 1H), 2.21-2.11 (m, 1H), 1.97-0.88 (m, 1H), 0.84 (t, J = 7.2 Hz, 3H). **^{13}C NMR** (101 MHz, DMSO-d_6) δ 176.14, 142.73, 140.27, 139.27, 129.33, 127.80, 127.04, 126.62, 126.57, 78.15, 32.73, 8.62. **HRMS (ESI$^-$)** calcd for $C_{16}H_{15}O_3^-$ [M-H]$^-$: 255.1027, found 255.1030.

2-([1,1'-biphenyl]-4-yl)-2-hydroxyhexanoic acid (2c)

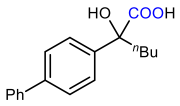

45.5 mg, 80% yield; White solid;

R_f = 0.54 (DCM/MeOH 10/1);

Mp: 128-130 °C;

^{1}H NMR (400 MHz, DMSO-d_6) δ 7.64 – 7.54 (m, 6H), 7.42 (t, J = 7.6 Hz, 2H), 7.31 (t, J = 7.3 Hz, 1H), 2.10 – 1.95 (m, 1H), 1.91 – 1.74 (m, 1H), 1.29 – 1.15 (m, 4H), 0.81 (t, 3H). **^{13}C NMR** (101 MHz, DMSO-d_6) δ 176.53, 143.71, 140.43, 139.00, 129.37, 127.77, 127.06, 126.66, 126.45, 77.91, 26.21, 22.90, 14.49. **HRMS (ESI$^-$)** calcd for $C_{18}H_{19}O_3^-$ [M-H]$^-$: 283.1340, found 283.1336.

2-([1,1'-biphenyl]-4-yl)-2-hydroxy-3-methylbutanoic acid (2d)

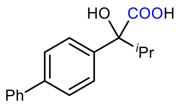

20.7 mg, 38%; 42.6 mg, 79% yield; White solid;

R$_f$ = 0.64 (DCM/MeOH 10/1);

Mp: 117-119 °C;

^{1}H NMR (400 MHz, DMSO-d_6) δ 7.69-7.62 (m, 6H), 7.48-7.44 (t, J = 7.5 Hz, 2H), 7.35 (m, 1H), 2.59 (q, J = 6.7 Hz, 1H), 0.97 (d, J = 6.6 Hz, 3H), 0.62 (d, J = 6.7 Hz, 3H). **^{13}C NMR** (101 MHz, DMSO-d_6) δ 176.41, 142.25, 139.09, 129.33, 127.78, 127.02, 126.91, 126.45, 80.55, 35.60, 17.82, 16.43. **HRMS** (**ESI**$^-$) calcd for $C_{17}H_{17}O_3^-$ [M-H]$^-$: 269.1183, found 269.1188.

2-([1,1'-biphenyl]-4-yl)-2-hydroxy-3,3-dimethylbutanoic acid (2e)

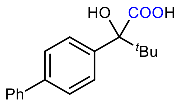

37.2 mg, 65% yield; White solid;

R$_f$ = 0.69 (DCM/MeOH 10/1);

Mp: 116-118 °C;

^{1}H NMR (400 MHz, DMSO-d_6) δ 7.80 – 7.74 (m, 2H), 7.70 – 7.59 (m, 4H), 7.48-7.44 (m, 2H), 7.39 – 7.33 (m, 1H), 0.98 (s, 9H). **^{13}C NMR** (101 MHz, DMSO-d_6) δ 175.90, 140.19, 139.97, 139.02, 129.33, 128.43, 127.79, 126.98, 125.54, 82.05, 38.75, 26.25. **HRMS** (**ESI**$^-$) calcd for $C_{18}H_{19}O_3^-$ [M-H]$^-$: 283.1340, found 283.1343.

2-hydroxy-2-(naphthalen-2-yl)propanoic acid (2f)

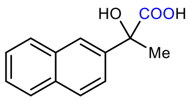

20.6 mg, 68% yield; Pale yellow solid;

R$_f$ = 0.35 (DCM/MeOH 10/1);

Mp: 138-140 °C;

^{1}H NMR (400 MHz, DMSO-d_6) δ 8.04 (br s, 1H), 7.93 – 7.84 (m, 4H), 7.68 (dd, J = 8.6, 1.9 Hz, 1H), 7.53 – 7.46 (m, 2H), 1.72 (s, 3H). **^{13}C NMR** (101 MHz, DMSO-d_6) δ 176.62, 142.83, 132.99, 132.50, 128.48, 127.77, 127.69, 126.51, 126.28, 124.64, 123.98, 75.52, 27.82. **HRMS** (**ESI**$^-$) calcd for $C_{13}H_{11}O_3^-$ [M-H]$^-$:215.0714, found 215.0718.

2-hydroxy-2-(4-(thiophen-2-yl)phenyl)propanoic acid (2g)

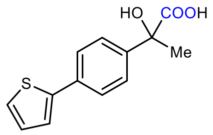

34.5 mg, 68% yield; White solid;

R_f = 0.49 (DCM/MeOH 10/1);

Mp: 152-154 °C;

^{1}H NMR (400 MHz, DMSO-d_6) δ 7.65 – 7.59 (m, 2H), 7.57 – 7.51 (m, 3H), 7.49 (d, J = 3.6 Hz, 1H), 7.48 – 7.50 (m, 1H), 1.63 (s, 3H). **^{13}C NMR** (101 MHz, DMSO-d_6) δ 176.32, 144.13, 143.46, 132.98, 128.90, 126.42, 126.04, 125.40, 124.08, 75.16, 27.65. **HRMS** (**ESI**–) calcd for $C_{13}H_{11}O_3S^-$ $[M-H]^-$: 247.0434, found 247.0433.

2-(4-(tert-butoxycarbonyl)phenyl)-2-hydroxypropanoic acid (2h)

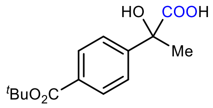

37.1 mg, 70% yield; White solid;

R_f = 0.34 (DCM/MeOH 10/1);

Mp: 106-108 °C;

^{1}H NMR (400 MHz, DMSO-d_6) δ 7.87 (d, J = 8.2 Hz, 2H), 7.64 (d, J = 8.2 Hz, 2H), 1.63 (s, 3H), 1.54 (s, 9H). **^{13}C NMR** (101 MHz, DMSO-d_6) δ 176.00, 165.26, 149.86, 130.54, 129.11, 125.89, 81.02, 75.44, 28.25, 27.82. **HRMS** (**ESI**–) calcd for $C_{14}H_{17}O_5^-$ $[M-H]^-$: 265.1081, found 265.1080.

2-cyclohexyl-2-hydroxy-2-phenylacetic acid (2i)

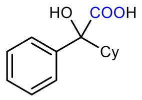

34.1 mg, 73% yield; white solid;

R_f = 0.48 (DCM/MeOH 10/1);

Mp: 122-124 °C;

^{1}H NMR (400 MHz, DMSO-d_6) δ 7.58 (d, J = 7.7 Hz, 2H), 7.32 (t, J = 7.5 Hz, 2H), 7.23 (t, J = 7.2 Hz, 1H), 2.23 – 2.07 (m, 1H), 1.80 – 1.68 (m, 1H), 1.65 – 1.45 (m, 3H), 1.42 – 1.21 (m, 2H), 1.15 – 0.89 (m, 4H). **^{13}C NMR** (101 MHz, DMSO-d_6) δ 176.42, 142.53, 128.12, 127.23, 126.28, 80.60, 45.63, 27.57, 26.39, 26.27, 25.82. **HRMS** (**ESI**–) calcd for $C_{14}H_{17}O_3^-$ $[M-H]^-$: 233.1183, found 233.1182. The spectroscopic data correspond to those previously reported in the literature[7].

2-hydroxy-2,3-diphenylpropanoic acid (2j)

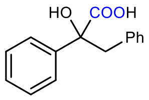

29.9 mg, 62% yield; white solid;

R$_f$ = 0.40 (DCM/MeOH 10/1);

Mp: 101-103 °C;

^{1}H NMR (400 MHz, DMSO-*d*$_6$) δ 7.60 – 7.54 (m, 2H), 7.36 – 7.28 (m, 2H), 7.28 – 7.22 (m, 1H), 7.20 – 7.09 (m, 5H), 3.42 (d, *J* = 13.7 Hz, 1H), 3.15 (d, *J* = 13.7 Hz, 1H). **^{13}C NMR** (101 MHz, DMSO-*d*$_6$) δ 175.67, 143.34, 137.31, 131.07, 128.15, 127.79, 127.52, 126.45, 126.13, 78.45, 45.40. **HRMS** (**ESI**$^-$) calcd for $C_{15}H_{13}O_3^-$ [M-H]$^-$: 241.0870, found 241.0872. The spectroscopic data correspond to those previously reported in the literature[8].

2-hydroxy-2-phenylpropanoic acid (2k)

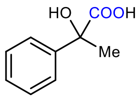

18.6 mg, 56% yield; white solid;

R$_f$ = 0.13 (DCM/MeOH 10/1);

Mp: 106-108 °C;

^{1}H NMR (400 MHz, DMSO-*d*$_6$) δ 7.55 – 7.49 (m, 2H), 7.35 – 7.29 (m, 2H), 7.27 – 7.21 (m, 1H), 1.60 (s, 3H). **^{13}C NMR** (101 MHz, DMSO-*d*$_6$) δ 176.56, 145.03, 128.21, 127.33, 125.60, 75.25, 27.81. **HRMS** (**ESI**+) calcd for $C_9H_{10}NaO_3^+$ [M+Na]$^+$: 189.0522, found 189.0517.

2-hydroxy-2-(2-methoxyphenyl)propanoic acid (2l)

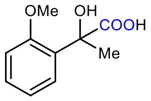

22.6 mg, 58%; white solid;

R$_f$ = 0.23 (DCM/MeOH 10/1);

Mp: 114-116 °C;

^{1}H NMR (400 MHz, DMSO-*d*$_6$) δ 7.56 – 7.42 (m, 1H), 7.31 – 7.11 (m, 1H), 6.99 – 6.79 (m, 2H), 3.66 (s, 3H), 1.46 (s, 3H). **^{13}C NMR** (101 MHz, DMSO-*d*$_6$) δ 175.73, 156.29, 133.99, 128.60, 125.86, 120.45, 111.63, 73.65, 55.81, 25.88. **HRMS** (**ESI+**): calcd for $C_{10}H_{12}NaO_4^+$ [M+Na]$^+$: 219.0628, found 219.0627.

2-hydroxy-2,2-diphenylacetic acid (4a)

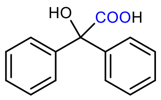

43.2 mg, 95% yield; White solid;

Mp: 114-116 °C;

^{1}H NMR (400 MHz, DMSO-*d*$_6$) δ 7.41 – 7.33 (m, 4H), 7.33 – 7.27 (m, 4H), 7.27 – 7.22 (m, 2H). **^{13}C NMR** (101 MHz, DMSO-*d*$_6$) δ 175.15, 144.14, 128.10, 127.65, 127.52, 80.69. **HRMS** (ESI$^-$):calcd for $C_{14}H_{11}O_3^-$ [M-H]$^-$: 227.07, found 227.00. The spectroscopic data correspond to those previously reported in the literature[8].

2-([1,1'-biphenyl]-4-yl)-2-hydroxy-2-phenylacetic acid (4b)

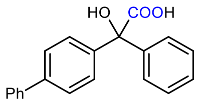

55.7 mg, 90% yield; White solid;

R$_f$ = 0.19 (DCM/MeOH 10/1);

Mp: 163-165 °C;

^{1}H NMR (400 MHz, DMSO-*d*$_6$) δ 7.64 (7.73 – 7.57, m, 4H), 7.58 – 7.16 (m, 10H).**^{13}C NMR** (101 MHz, DMSO-*d*$_6$) δ 175.08, 144.06, 143.35, 140.20, 139.47, 129.36, 128.18, 128.14, 127.89, 127.73, 127.49, 127.09, 126.44, 80.55. **HRMS** (**ESI$^-$**) calcd for $C_{20}H_{15}O_3^-$ [M-H]$^-$: 303.1027, found 303.1025.

2-hydroxy-2-phenyl-2-(p-tolyl)acetic acid (4c)

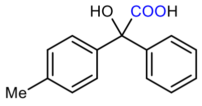

38.5 mg, 79% yield; White solid;

R$_f$ = 0.46 (DCM/MeOH 10/1);

Mp: 115-117 °C;

^{1}H NMR (400 MHz, DMSO-*d*$_6$) δ 7.40 – 7.19 (m, 7H), 7.12 (d, J = 8.1 Hz, 2H), 2.28 (s, 3H). **^{13}C NMR** (101 MHz, DMSO-*d*$_6$) δ 175.25, 144.24,141.19, 136.77, 128.63, 128.02, 127.57, 127.51, 127.44, 80.53, 21.03. **HRMS** (**ESI$^-$**) calcd for $C_{15}H_{13}O_3^-$ [M-H]$^-$: 241.0870, found 241.0867.

2-hydroxy-2-(4-methoxyphenyl)-2-phenylacetic acid (4d)

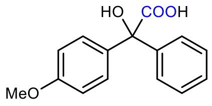

38.4 mg, 74% yield; White solid;

R$_f$ = 0.40 (DCM/MeOH 10/1);

^{1}H NMR (400 MHz, DMSO-*d*$_6$) δ 7.39 – 7.18 (m, 7H), 6.90 – 6.81 (m, 2H), 3.71 (s, 3H). **^{13}C NMR** (101 MHz, DMSO-*d*$_6$) δ 175.42, 158.76, 144.40, 136.15, 128.78, 128.06, 127.59, 127.52, 113.41,

80.34, 55.47. **HRMS** (**ESI**⁻) calcd for $C_{15}H_{13}O_4^-$ $[M-H]^-$: 257.0819, found 257.0816. The spectroscopic data correspond to those previously reported in the literature[9].

2-hydroxy-2-phenyl-2-(4-(trifluoromethyl)phenyl)acetic acid (4e)

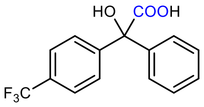

40.9 mg, 69% yield; Pale yellow oil;

R$_f$ = 0.28 (DCM/MeOH 10/1);

^{1}H NMR (400 MHz, DMSO-*d*$_6$) δ 7.71 (d, J = 8.3 Hz, 2H), 7.61 (d, J = 8.2 Hz, 2H), 7.40 – 7.27 (m, 5H). **^{13}C NMR** (101 MHz, DMSO-*d*$_6$) δ 174.50, 148.89, 143.84, 128.43, 128.35, 127.90, 128.23 (q, *J* = 31.8 Hz). 127.39, 124.75 (q, *J* = 272.0 Hz), 125.00 (q, *J* = 3.8 Hz), 80.54. **^{19}F NMR** (376 MHz, DMSO-*d*$_6$) δ -60.94. **HRMS** (**ESI**⁻) calcd for $C_{15}H_{10}F_3O_3^-$ $[M-H]^-$: 295.0588, found 295.0951.

2-(4-fluorophenyl)-2-hydroxy-2-phenylacetic (4f)

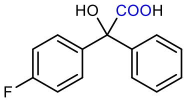

40.1 mg, 82% yield; White solid;

R$_f$ = 0.15 (DCM/MeOH 10/1);

Mp: 102-104 °C;

^{1}H NMR (400 MHz, DMSO-*d*$_6$) δ 7.40 – 7.23 (m, 7H), 7.17 – 7.09 (m, 2H). **^{13}C NMR** (101 MHz, DMSO-*d*$_6$) δ 174.97, 161.70 (d, *J* = 243.6 Hz), 144.03, 140.31 (d, *J* = 3.1 Hz), 129.60 (d, *J* = 8.3 Hz), 128.21, 127.78, 127.37, 114.79 (d, *J* = 21.3 Hz), 80.26. **^{19}F NMR** (376 MHz, DMSO-*d*$_6$) δ -115.66. **HRMS** (**ESI**⁻) calcd for $C_{14}H_{10}FO_3^-$ $[M-H]^-$: 245.0619, found 245.0610.

2-(4-chlorophenyl)-2-hydroxy-2-phenylacetic acid (4g)

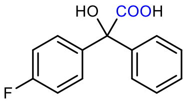

2.6 mg, 81% yield; White solid;

R$_f$ = 0.15 (DCM/MeOH 10/1);

Mp: 82-84 °C;

^{1}H NMR (400 MHz, DMSO-*d*$_6$) δ 7.48 – 7.20 (m, 9H). **^{13}C NMR** (101 MHz, DMSO-*d*$_6$) δ 174.73, 143.85, 143.14, 132.34, 129.45, 128.25, 128.04, 127.83, 127.34, 80.28. **HRMS** (**ESI**⁻) calcd for $C_{14}H_{10}ClO_3^-$ $[M-H]^-$: 261.0324, found 261.0321.

2-(3-fluorophenyl)-2-hydroxy-2-phenylacetic acid (4h)

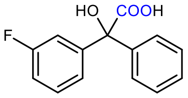

43.8 mg, 89% yield; Colorless oil;

R$_f$ = 0.14 (DCM/MeOH 10/1);

^{1}H NMR (400 MHz, DMSO-d_6) δ 7.39 – 7.23 (m, 6H), 7.22 – 7.18 (m, 1H), 7.16 – 7.06 (m, 2H). **^{13}C NMR** (101 MHz, DMSO-d_6) δ 174.59, 162.08 (d, J = 242.6 Hz), 147.08 (d, J = 6.8 Hz), 143.82, 130.01 (d, J = 8.1 Hz), 128.27, 127.84, 127.33, 123.72 (d, J = 2.7 Hz), 114.42 (d, J = 20.9 Hz), 114.25 (d, J = 22.9 Hz), 80.29. **^{19}F NMR** (376 MHz, DMSO-d_6) δ -113.64. **HRMS** (**ESI**$^-$) calcd for $C_{14}H_{10}FO_3^-$ [M-H]$^-$: 245.0619, found 245.0613.

2-(3-chlorophenyl)-2-hydroxy-2-phenylacetic acid (4i)

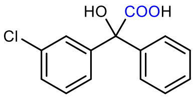

46.2 mg, 88% yield; Colorless oil;

R$_f$ = 0.19 (DCM/MeOH 10/1);

^{1}H NMR (400 MHz, DMSO-d_6) δ 7.77 – 7.00 (m, 9H). **^{13}C NMR** (101 MHz, DMSO-d_6) δ 174.55, 146.54, 143.59, 132.87, 130.04, 128.34, 127.95, 127.65, 127.31, 127.26, 126.34, 80.33. **HRMS** (**ESI**$^-$) calcd for $C_{14}H_{10}ClO_3^-$[M-H]$^-$: 261.0324, found 261.0325.

2-(2-fluorophenyl)-2-hydroxy-2-phenylacetic acid (4j)

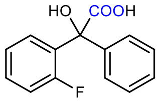

44.4 mg, 90% yield; White solid;

R$_f$ = 0.60 (DCM/MeOH 10/1);

Mp: 55-57 °C;

^{1}H NMR (400 MHz, DMSO-d_6) δ 7.54 (d, J = 7.3 Hz, 2H), 7.41 – 7.29 (m, 4H), 7.16 (dd, J = 11.4, 8.1 Hz, 1H), 7.08 (t, J = 7.5 Hz, 1H), 6.95 (dt, J = 7.9, 4.0 Hz, 1H). **^{13}C NMR** (101 MHz, DMSO-d_6) δ 174.57, 160.65 (d, J = 247.8 Hz), 141.65, 132.40 (d, J = 12.7 Hz), 130.19 (d, J = 8.5 Hz), 129.34 (d, J = 3.8 Hz), 128.17, 128.01, 127.20, 124.00 (d, J = 3.3 Hz), 116.08 (d, J = 22.2 Hz), 78.15. **^{19}F NMR** (376 MHz, DMSO-d_6) δ -110.44. **HRMS** (**ESI**$^-$) calcd for $C_{14}H_{10}CFO_3^-$ [M-H]$^-$: 245.0619, found 245.0615.

2-hydroxy-2-phenyl-2-(o-tolyl)acetic acid (4k)

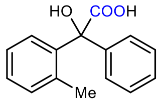

27.3 mg, 56%; Colorless oil;

R_f = 0.39 (DCM/MeOH 10/1);

^{1}H NMR (400 MHz, DMSO-d_6) δ 7.49 – 7.44 (m, 2H), 7.39 – 7.28 (m, 3H), 7.20 – 7.13 (m, 2H), 7.05 (td, *J* = 7.2, 6.8, 2.2 Hz, 1H), 6.84 (dd, *J* = 7.8, 1.2 Hz, 1H), 2.19 (s, 3H). **^{13}C NMR** (101 MHz, DMSO-d_6) δ 175.65, 142.99, 142.30, 138.04, 132.10, 128.16, 128.10, 127.95, 127.72, 127.36, 125.25, 81.52, 20.92. **HRMS** (**ESI**$^-$) calcd for $C_{15}H_{13}FO_3^-$ [M-H]$^-$: 241.0870, found 241.0872.

2,2-bis(4-fluorophenyl)-2-hydroxyacetic acid (4l)

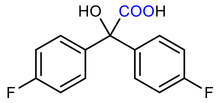

31.1 mg, 59% yield; White solid;

R_f = 0.16 (DCM/MeOH 10/1);

Mp: 117-119 °C;

^{1}H NMR (400 MHz, DMSO-d_6) δ 7.52 – 7.33 (m, 4H), 7.15 (t, *J* = 8.7 Hz, 4H). **^{13}C NMR** (101 MHz, DMSO-d_6) δ 174.84, 161.67 (d, *J* = 243.4 Hz), 140.69 (d, *J* = 3.0 Hz), 129.51 (d, *J* = 8.2 Hz), 114.81 (d, *J* = 21.3 Hz), 79.75. **^{19}F NMR** (376 MHz, DMSO-d_6) δ -115.63. **HRMS** (**ESI**$^-$) calcd for $C_{14}H_9F_2O_3^-$ [M-H]$^-$: 263.0525, found 263.0521.

2,2-bis(4-chlorophenyl)-2-hydroxyacetic acid (4m)

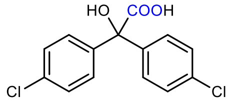

45.8 mg, 77% yield; White solid;

R_f = 0.15 (DCM/MeOH 10/1);

Mp: 104-106 °C;

^{1}H NMR (400 MHz, DMSO-d_6) δ 7.89 – 7.17 (m, 8H). **^{13}C NMR** (101 MHz, DMSO-d_6) δ 174.39, 142.91, 132.52, 129.32, 128.20, 79.87. **HRMS** (**ESI**$^-$) calcd for $C_{14}H_9Cl_2O_3^-$ [M-H]$^-$: 294.9934, found 294.9933.

2-hydroxy-2,2-di-p-tolylacetic acid (4n)

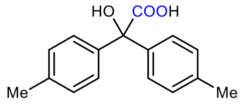

36.9 mg, 72% yield; White solid;

R_f = 0.20 (DCM/MeOH 10/1);

^{1}H NMR (400 MHz, DMSO-d_6) δ 7.25 – 7.17 (m, 4H), 7.09 (d, J = 7.9 Hz, 4H), 2.25 (s, 6H). **^{13}C NMR** (101 MHz, DMSO-d_6) δ 175.41, 141.34, 136.70, 128.59, 127.47, 80.38, 21.05. **HRMS** (**ESI**$^-$)

calcd for $C_{16}H_{15}O_3^-$ $[M\text{-}H]^-$: 255.1027, found 255.1031. The spectroscopic data correspond to those previously reported in the literature[9].

2-(4-fluorophenyl)-2-hydroxy-2-(p-tolyl)acetic acid (4o)

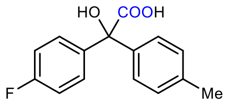

38.0 mg, 73% yield; White solid;

$\mathbf{R_f}$ = 0.20 (DCM/MeOH 10/1);

Mp: 83-85 °C;

^{1}H NMR (400 MHz, DMSO-d_6) δ 7.44 – 7.34 (m, 2H), 7.29 – 7.21 (m, 2H), 7.20 – 7.01 (m, 4H), 2.28 (s, 3H). **^{13}C NMR** (101 MHz, DMSO-d_6) δ 175.09, 161.66 (d, J = 243.4 Hz), 141.14, 140.48 (d, J = 3.2 Hz), 136.92, 129.60 (d, J = 8.3 Hz), 128.75, 127.31, 114.72 (d, J = 21.3 Hz), 80.10, 21.03. **^{19}F NMR** (376 MHz, DMSO-d_6) δ -115.81. **HRMS (ESI–)** calcd for $C_{15}H_{12}FO_3^-$ $[M\text{-}H]^-$: 259.0776, found 259.0774.

methyl 9-hydroxy-9H-xanthene-9-carboxylate (4p)

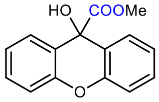

27.9 mg, 55% yield; Colorless oil;

$\mathbf{R_f}$ = 0.25 (PE/EA 50/1);

^{1}H NMR (400 MHz, $CDCl_3$) δ 7.48 (ddd, J = 7.8, 1.7, 0.5 Hz, 2H), 7.35 (ddd, J = 8.3, 7.2, 1.7 Hz, 2H), 7.20 – 7.11 (m, 4H), 4.92 (s, 1H), 3.60 (s, 3H). **^{13}C NMR** (101 MHz, $CDCl_3$) δ 174.89, 150.16, 129.91, 126.59, 123.41, 121.76, 116.78, 69.81, 53.95. **HRMS (ESI+)** calcd for $C_{15}H_{12}NaO_4^+$ $[M+Na]^+$: 279.0628, found 279.0631.

methyl 2-hydroxy-2-phenyl-2-(thiophen-2-yl)acetate (4q)

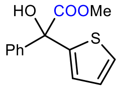

36.6 mg, 74% yield; Colourless oil;

$\mathbf{R_f}$ = 0.2 (PE/EA 50/1);

^{1}H NMR (400 MHz, $CDCl_3$) δ 7.49 (dt, J = 7.0, 2.8 Hz, 2H), 7.40 – 7.26 (m, 4H), 7.17 – 7.04 (m, 1H), 7.03 – 6.94 (m, 1H), 4.47 (s, 1H), 3.85 (s, 3H). **^{13}C NMR** (101 MHz, $CDCl_3$) δ 173.83, 128.43, 128.22, 126.69, 126.56, 126.36, 125.92, 53.87. **HRMS (ESI+)** calcd for $C_{13}H_{12}NaO_3S^+$ $[M+Na]^+$: 271.0399, found 271.0400.

methyl 2-hydroxy-2,2-di(thiophen-2-yl)acetate (4r)

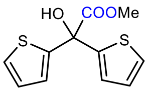

35.7 mg, 70% yield; Pale yellow solid;

R$_f$ = 0.2 (PE/EA 50/1);

^{1}H NMR (400 MHz, $CDCl_3$) δ 7.28 (dd, *J* = 5.1, 1.3 Hz, 2H), 7.16 (dd, *J* = 3.7, 1.3 Hz, 2H), 6.97 (dd, *J* = 5.1, 3.6 Hz, 2H), 4.71 (s, 1H), 3.88 (s, 3H). **^{13}C NMR** (101 MHz, $CDCl_3$) δ 172.81, 126.77, 126.01, 125.94, 76.52, 54.13. **HRMS (ESI**+) calcd for $C_{11}H_{10}NaO_3S_2^+$ [M+Na]$^+$: 276.9964, found 276.9969. The spectroscopic data correspond to those previously reported in the literature[10].

2-hydroxy-3,3-dimethyl-2-(((2-methyl-1-phenylpropan-2-yl)oxy)carbonyl)butanoic acid (6a)

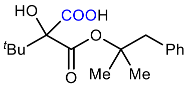

46.3 mg, 75% yield; White solid;

R$_f$ = 0.39 (DCM/MeOH 10/1);

Mp: 102-104 °C;

^{1}H NMR (400 MHz, $CDCl_3$) δ 7.32 – 7.20 (m, 5H), 3.16 – 3.04 (m, 2H), 1.53 (d, *J* = 3.6 Hz, 6H), 1.08 (s, 9H). **^{13}C NMR** (101 MHz, $CDCl_3$) δ 169.96, 169.39, 135.96, 130.70, 128.10, 126.82, 87.81, 83.46, 47.04, 39.12, 25.55, 25.46, 25.30. **HRMS (ESI$^-$)** calcd for $C_{17}H_{23}O_5^-$ [M-H]$^-$: 307.1551, found 307.1552.

2-(tert-butoxycarbonyl)-2-hydroxy-3-methylhexanoic acid (6b)

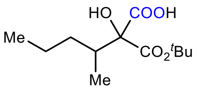

32.7 mg, 66% yield; Oil; d.r.: 2.5/1

R$_f$ = 0.39 (DCM/MeOH 10/1);

^{1}H NMR (400 MHz, DMSO-*d*$_6$) δ 2.27 – 2.12 (m, 1H), 1.40 (s, 9H), 1.29 – 1.05 (m, 4H), 0.96 – 0.65 (m, 6H). **^{13}C NMR** (101 MHz, DMSO-*d*$_6$) δ 172.32, 172.28, 170.16, 170.09, 82.96, 82.88, 81.68, 81.61, 37.56, 37.28, 33.57, 33.54, 27.91, 20.64, 20.59, 14.65, 14.50, 14.03. **HRMS (ESI+)** calcd for $C_{12}H_{22}NaO_5^+$ [M+Na]$^+$: 269.1359, found 269.1355. The *d.r.* value was determined by ^{13}C NMR analysis of isolated product.

2-(tert-butoxycarbonyl)-2-hydroxyhexanoic acid (6c)

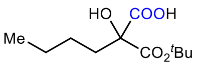

21.6 mg, 46% yield; Oil;

R$_f$ = 0.54 (DCM/MeOH 10/1);**^{1}H NMR** (400 MHz, DMSO-*d*$_6$) δ 1.76 – 1.64 (m, 2H), 1.35 (s, 9H), 1.26 – 1.12 (m, 4H), 0.81 (t, *J* = 7.0 Hz, 3H). **^{13}C NMR** (101 MHz, DMSO-

d_6) δ 172.52, 170.27, 81.48, 79.12, 35.19, 27.93, 25.48, 22.75, 14.35. **HRMS** (**ESI**+) calcd for $C_{11}H_{20}NaO_5^+$ [M+Na]$^+$:255.1203, found 255.1206.

2-(tert-butoxycarbonyl)-2-hydroxyhex-5-enoic acid (6d)

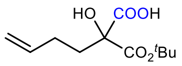

20.2 mg, 44% yield; Oil;

R$_f$ = 0.50 (DCM/MeOH 10/1);

^{1}H NMR (400 MHz, DMSO-d_6) δ 5.89 – 5.73 (m, 1H), 5.07 – 4.87 (m, 2H), 2.04 – 1.94 (m, 2H), 1.90 – 1.78 (m, 2H), 1.40 (s, 9H). **^{13}C NMR** (101 MHz, DMSO-d_6) δ 172.33, 170.13, 138.64, 115.26, 81.66, 78.80, 34.80, 27.93, 27.74. **HRMS** (**ESI**+) calcd for $C_{11}H_{18}NaO_5^+$ [M+Na]$^+$:253.1046, found 253.1044.

2-(tert-butoxycarbonyl)-2-hydroxy-5-phenylpentanoic acid (6e)

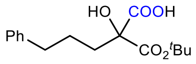

30.3 mg, 51% yield; oil;

R$_f$ = 0.68 (DCM/MeOH 10/1);

^{1}H NMR (400 MHz, DMSO-d_6) δ 7.29 – 7.25 (m, 2H), 7.19 – 7.14 (m, 3H), 2.66 – 2.51 (m, 2H), 1.84 – 1.70 (m, 2H), 1.64 – 1.43 (m, 2H), 1.36 (s, 9H). **^{13}C NMR** (101 MHz, DMSO-d_6) δ 172.43, 170.21, 142.36, 128.72, 128.70, 126.15, 81.50, 79.06, 35.53, 34.97, 27.89, 25.30. **HRMS** (**ESI**$^-$) calcd for $C_{16}H_{21}O_5^-$ [M-H]$^-$:293.1394, found 293.1390.

3-(((3*S*,8*S*,9*S*,10*R*,13*R*,14*S*,17*R*)-10,13-dimethyl-17-((*R*)-6-methylheptan-2-yl)-2,3,4,7,8,9,10,11,12,13,14,15,16,17-tetradecahydro-1*H*-cyclopenta[a]phenanthren-3-yl)oxy)-2-hydroxy-2-methyl-3-oxopropanoic acid (6f)

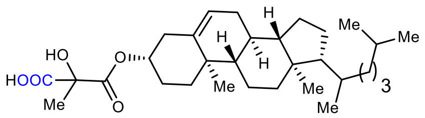

35.8 mg, 36% yield; White solid;

R$_f$ = 0.35 (DCM/MeOH 10/1);

^{1}H NMR (400 MHz, DMSO-d_6) δ 5.47 – 5.32 (m, 1H), 4.65 – 4.46 (m, 1H), 2.40 – 2.19 (m, 2H), 2.08 – 1.76 (m, 5H), 1.63 – 0.93 (m, 21H), 0.92 – 0.86 (m, 6H), 0.71 (s, 3H). **^{13}C NMR** (101 MHz, DMSO-d_6) δ 172.76, 170.90, 139.67, 122.71, 76.07, 74.63, 56.60, 56.13, 49.90, 42.32, 39.46, 37.78, 36.87, 36.53, 36.18, 35.75, 31.82, 28.28, 27.89, 27.45, 24.33, 23.80, 23.14, 23.12, 23.09, 22.84, 21.05, 19.40, 19.00, 12.10. **HRMS (ESI+)** calcd for $C_{31}H_{50}NaO_5^+$ [M+Na]$^+$: 525.3550, found 525.3553.

methyl 2-hydroxy-2-methyl-3-(methyl(phenyl)amino)-3-oxopropanoate (6g)

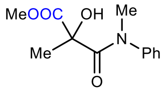

26.3 mg, 55% yield; Oil;

R_f = 0.31 (PE/EA 2/1);

^{1}H NMR (400 MHz, $CDCl_3$) δ 7.42 – 7.35 (m, 3H), 7.23 – 7.15 (m, 2H), 4.28 (s, 1H), 3.62 (s, 3H), 3.30 (s, 3H), 1.41 (s, 3H). **^{13}C NMR** (101 MHz, $CDCl_3$) δ 172.04, 170.04, 141.53, 129.44, 128.85, 75.74, 52.82, 40.30, 24.01. **HRMS** (**ESI**+) calcd for $C_{12}H_{15}NNaO_4^+$ $[M+Na]^+$: 260.0893, found 260.0891.

methyl 3-(ethyl(phenyl)amino)-2-hydroxy-2-methyl-3-oxopropanoate (6h)

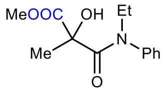

27.5 mg, 55% yield; Oil;

R_f = 0.40 (PE/EA 2/1);

^{1}H NMR (400 MHz, $CDCl_3$) δ 7.45 – 7.32 (m, 3H), 7.15 (dd, J = 6.6, 3.1 Hz, 2H), 4.38 (s, 1H), 3.84 (dt, J = 14.2, 7.0 Hz, 1H), 3.72 – 3.54 (m, 4H), 1.39 (s, 3H), 1.15 (t, J = 7.2 Hz, 3H). **^{13}C NMR** (101 MHz, $CDCl_3$) δ 171.98, 169.48, 139.73, 129.85, 129.25, 128.98, 75.74, 52.70, 47.19, 23.93, 12.48. **HRMS** (**ESI**+) calcd for $C_{13}H_{17}NNaO_4^+$ $[M+Na]^+$: 274.1050, found 274.1045.

methyl 3-(butyl(phenyl)amino)-2-hydroxy-2-methyl-3-oxopropanoate (6i)

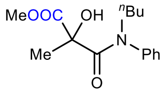

33.2 mg, 59% yield; Oil;

R_f = 0.49 (PE/EA 2/1);

^{1}H NMR (400 MHz, $CDCl_3$) δ 7.43 – 7.33 (m, 3H), 7.19 – 7.09 (m, 2H), 4.36 (s, 1H), 3.85 – 3.73 (m, 1H), 3.69 – 3.49 (m, 4H), 1.57 – 1.45 (m, 2H), 1.40 – 1.23 (m, 5H), 0.89 (t, J = 7.3 Hz, 3H). **^{13}C NMR** (101 MHz, $CDCl_3$) δ 172.04, 169.79, 140.08, 129.85, 129.26, 128.96, 75.83, 52.70, 52.13, 29.32, 23.98, 19.89, 13.82. **HRMS** (**ESI**+) calcd for $C_{15}H_{21}NNaO_4^+$ $[M+Na]^+$: 302.1363, found 302.1362.

methyl 2-hydroxy-3-(isopropyl(phenyl)amino)-2-methyl-3-oxopropanoate (6j)

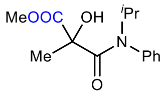

25.8 mg, 49% yield; Oil;

R_f = 0.47 (PE/EA 2/1)

^{1}H NMR (400 MHz, $CDCl_3$) δ 7.46 – 7.34 (m, 3H), 7.13 – 7.05 (m, 2H), 4.97 (hept, J = 6.8 Hz, 1H), 4.49 (s, 1H), 3.60 (s, 3H), 1.36 (s, 3H), 1.06 (dd, J = 10.0, 6.8 Hz, 6H). **^{13}C NMR** (101 MHz,

$CDCl_3$) δ 171.84, 169.62, 135.40, 132.02, 131.73, 129.26, 128.61, 128.57, 75.91, 52.60, 48.97, 24.03, 20.62, 20.36. **HRMS** (**ESI**+) calcd for $C_{14}H_{19}NNaO_4^+$ $[M+Na]^+$: 288.1206, found 288.1203.

2-(benzoyloxy)-2-methyl-3-oxo-3-(piperidin-1-yl)propanoic acid (6k)

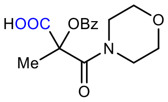

25.2 mg, 41%; White solid;

R_f = 0.22 (DCM/MeOH 3/1);

Mp: 85-87 °C;

^{1}H NMR (400 MHz, $CDCl_3$) δ 8.13 – 8.04 (m, 2H), 7.64 (t, J = 7.5 Hz, 1H), 7.49 (t, J = 7.7 Hz, 2H), 3.60 (d, J = 76.8 Hz, 9H), 1.95 (s, 3H). **^{13}C NMR** (101 MHz, DMSO-d_6) δ 169.92, 165.40, 164.09, 134.30, 129.80, 129.68, 129.44, 82.70, 66.52, 66.08, 46.58, 43.39, 21.51. **HRMS** (**ESI+**): calcd for $C_{16}H_{16}NaO_3^+$ $[M+Na]^+$:330.0948, found: 330.0941.

methyl 2-([1,1'-biphenyl]-4-yl)-2-hydroxyacetate (8a)

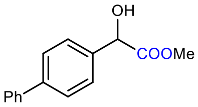

29.0 mg, 60% yield; White solid;

R_f = 0.36 (PE/EA 2/1);

Mp: 106-108 °C ;**^{1}H NMR** (400 MHz, $CDCl_3$) δ 7.66 – 7.53 (m, 4H), 7.52 – 7.39 (m, 4H), 7.38 – 7.30 (m, 1H), 5.22 (s, 1H), 3.77 (s, 3H), 3.46 (s, 1H). **^{13}C NMR** (101 MHz, $CDCl_3$) δ 174.09, 141.46, 140.52, 137.13, 128.78, 127.46, 127.38, 127.10, 127.00, 72.62, 53.15. **HRMS** (**ESI+**): calcd for $C_{15}H_{14}NaO_3^+$ $[M+Na]^+$: 265.0835, found 265.0829. The spectroscopic data correspond to those previously reported in the literature[11].

methyl 2-hydroxy-2-(4'-methyl-[1,1'-biphenyl]-4-yl)acetate (8b)

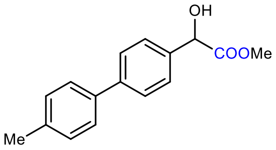

35.3mg, 69%; White solid;

R_f = 0.33 (PE/EA 2/1);

Mp: 116-118 °C;

^{1}H NMR (400 MHz, $CDCl_3$) δ 7.60 – 7.56 (m, 2H), 7.50 – 7.45 (m, 4H), 7.26 – 7.22 (m, 2H), 5.22 (d, J = 3.8 Hz, 1H), 3.79 (s, 3H), 3.44 (d, J = 5.4 Hz, 1H), 2.39 (s, 3H). **^{13}C NMR** (101 MHz, $CDCl_3$) δ 174.12, 141.39, 137.63, 137.29, 136.83, 129.49, 127.17, 126.97, 126.92, 72.65, 53.12, 21.10. **HRMS** (**ESI+**): calcd for $C_{16}H_{16}NaO_3^+$ $[M+Na]^+$: 279.0992, found 279. 0990.

methyl 2-hydroxy-2-(4'-(methylthio)-[1,1'-biphenyl]-4-yl)acetate (8c)

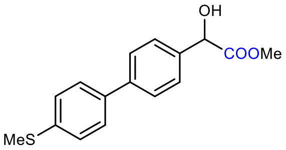

29.9mg, 52%; White solid;

R$_f$ = 0.26 (PE/EA 2/1).

Mp: 152-154 °C;

^{1}H NMR (400 MHz, $CDCl_3$) δ 7.58 – 7.52 (m, 2H), 7.52 – 7.43 (m, 4H), 7.33 – 7.28 (m, 2H), 5.21 (d, *J* = 3.5 Hz, 1H), 3.77 (s, 3H), 3.44 (d, *J* = 5.4 Hz, 1H), 2.51 (s, 3H). **^{13}C NMR** (101 MHz, $CDCl_3$) δ 174.07, 140.76, 137.91, 137.24, 137.07, 127.41, 127.06, 127.03, 126.80, 72.60, 53.16, 15.78. **HRMS** (**ESI+**): calcd for $C_{16}H_{16}NaO_3S^+$ $[M+Na]^+$: 311.0712, found 311. 0708.

methyl 2-hydroxy-2-(4-(thiophen-3-yl)phenyl)acetate (8d)

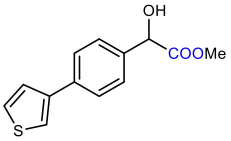

30.6 mg, 62%; White solid;

R$_f$ = 0.36 (PE/EA 2/1);

Mp: 127-129 °C;

^{1}H NMR (400 MHz, $CDCl_3$) δ 7.65 – 7.59 (m, 2H), 7.50 – 7.44 (m, 3H), 7.43 – 7.38 (m, 2H), 5.23 (d, *J* = 5.4 Hz, 1H), 3.81 (s, 3H), 3.46 (d, *J* = 5.6 Hz, 1H). **^{13}C NMR** (101 MHz, $CDCl_3$) δ 174.11, 141.74, 137.00, 136.15, 127.10, 126.68, 126.36, 126.28, 120.64, 72.66, 53.14. **HRMS** (**ESI+**): calcd for $C_{13}H_{12}NaO_3S^+$ $[M+Na]^+$: 271.0399, found 271.0395.

methyl 2-hydroxy-2-(naphthalen-2-yl)acetate (8e)

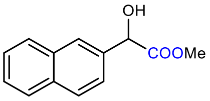

23.7mg, 55%; White solid;

R$_f$ = 0.37 (PE/EA 2/1);

Mp: 126-128 °C;

^{1}H NMR (400 MHz, $CDCl_3$) δ 7.92 – 7.87 (m, 1H), 7.87 – 7.79 (m, 3H), 7.54 – 7.42 (m, 3H), 5.33 (d, *J* = 5.4 Hz, 1H), 3.75 (s, 3H), 3.57 (d, *J* = 5.5 Hz, 1H). **^{13}C NMR** (101 MHz, $CDCl_3$) δ 174.18, 135.59, 133.34, 133.19, 128.53, 128.14, 127.74, 126.40, 126.01, 124.13, 73.07, 53.13. **HRMS** (**ESI+**): calcd for $C_{13}H_{12}NaO_3^+$ $[M+Na]^+$: 239.0679, found 239.0679. The spectroscopic data correspond to those previously reported in the literature[7].

methyl 2-hydroxy-2-(phenanthren-9-yl)acetate (8f)

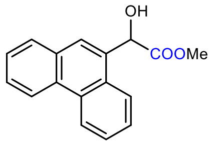

29.7 mg, 56% yield; White solid;

R$_f$ = 0.50 (PE/EA 2/1);

Mp: 60-62 °C;

^{1}H NMR (400 MHz, $CDCl_3$) δ 8.74 (dd, J = 7.9, 1.6 Hz, 1H), 8.66 (d, J = 8.3 Hz, 1H), 8.18 (dd, J = 7.9, 1.6 Hz, 1H), 7.88 (dd, J = 7.8, 1.4 Hz, 1H), 7.79 (s, 1H), 7.75 – 7.52 (m, 4H), 5.82 (d, J = 3.7 Hz, 1H), 3.73 (s, 3H), 3.55 (d, J = 4.4 Hz, 1H). **^{13}C NMR** (101 MHz, $CDCl_3$) δ 174.72, 132.14, 130.96, 130.62, 129.61, 128.93, 127.69, 127.37, 126.96, 126.89, 126.65, 124.48, 123.26, 122.52, 72.15, 53.22. **HRMS (ESI+)**: calcd for $C_{17}H_{14}NaO_3^+$ [M+Na]$^+$: 289.0835, found 289.0833.

4. Optimization details and optimization tables

Supplementary Table 1. Influence of photocatalyst and base.

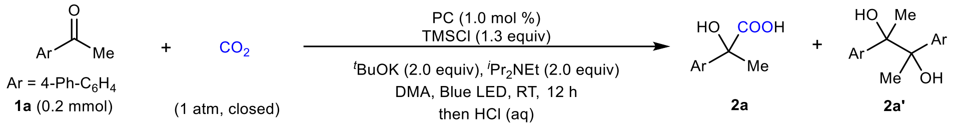

Entry	PC	**2a**^{a}	**2a'**^{a}	**1a**^{a}
1	Ir(ppy)$_2$(dtbbpy)PF$_6$	80% (80%)	19	0
2	Ir(dF(CF$_3$)ppy)$_2$(dtbbpy)PF$_6$	53%	15%	21%
3	Ir(dF(Me)ppy)$_2$(dtbbpy)PF$_6$	50%	15%	34%
4	4CzIPN	3%	0	69%
5	3DPA2FBN	24%	3%	0
6	w/o ^{t}BuOK, ^{i}Pr$_2$NEt (4.0 equiv)	0	76%	0
7	w/o ^{t}BuOK, ^{i}Pr$_2$NEt (6.0 equiv)	16%	61%	0

^{a}Yields were determined by UPLC analysis with 1,1'-biphenyl as internal standard.

Following general procedure for the carboxylation of alkyl aryl ketones, the screening of photocatalyst show that Ir(ppy)$_2$(dtbbpy)PF$_6$ was best (Entries 1-5). And the control experiments show that ^{t}BuOK was essential for chemical selectivity (entries 6-7).

Supplementary Table 2. Influence of lewis acid.

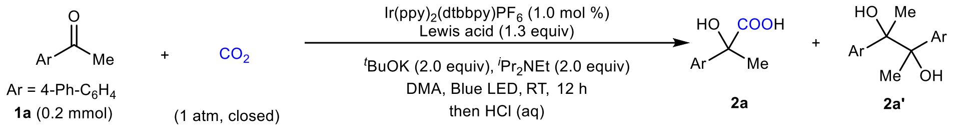

Entry	Lewis acid	**2a**[a]	**2a'**[a]	**1a**[a]
1	TMSCl	80% (80%),	19	0
2	TMSBr	76% (76%)	15%	0
3	TMSI	25%	13%	35%
4	Et_3SiCl	77% (77%)	22%	0
5	$^iPr_3SiCl$	24%	3%	32%
6	$^tBuMe_2SiCl$	9%	12%	25%
7	$PhMe_2SiCl$	76% (76%)	17%	0
8	$Ph_2MeSiCl$	60%	18%	11%
9	Ph_3SiCl	53%	19%	13%
10	TMSOTf	47%	19%	21%
11	$LiBF_4$	24%	31%	33%
12	$Sc(OTf)_3$	n.d.	94%	3%
13	B_2Pin_2	27%	0	14%
14	Cp_2TiCl	n.d.	6%	81%
15	TMSOMe	(39%)	(30%)	0
16	TMSCl (0.5 equiv)	35%	16%	35%

[a]Yields were determined by UPLC analysis with 1,1'-biphenyl as internal standard. The isolated yields are given in parentheses. n.d. = not detected.

Following general procedure for the carboxylation of alkyl aryl ketones, the screening of various lewis acids show that TMSCl was best (Entries 1-15). And the control experiments (entries 1,16) show that equivalent amount of TMSCl was essential for transformation efficiency.

Supplementary Table 3. Control experiments of [1,1'-biphenyl]-4-yl(phenyl)methanone **3b**.

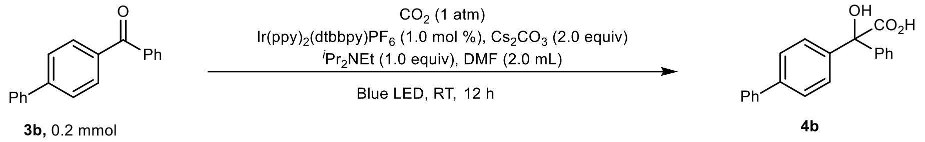

Entry	Derivation from standard conditions	**4b**[a], **3b**[b]
1	As shown	90%, n.d.
2	Without CO_2	n.d., 68%
3	Without light	n.d., 100%
4	Without $Ir(ppy)_2(dtbbpy)PF_6$	n.d., 98%
5	Without $^iPr_2NEt$	n.d., 95%

[a]Isolated yields. [b]LC yield

Following general procedure for the carboxylation of diaryl ketones, the control experiments show light, photocatalyst, $^iPr_2NEt$ and CO_2 were critical factors for this transformation.

Supplementary Table 4. The carboxylation of **7a** under carboxylation condition of alkyl aryl ketones.

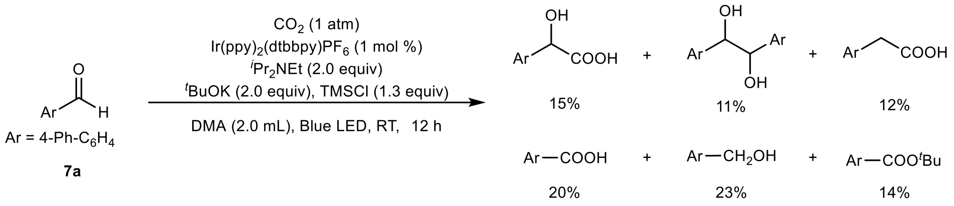

Following general procedure for the carboxylation of alkyl aryl ketones, we observed a complicated mixture of products due to intense side reactions, including pinacol coupling and disproportionation, which might arise from the lower steric hindrance of aldehydes and the strong base.

Supplementary Table 5. Control experiments for carboxylation of **7a**.

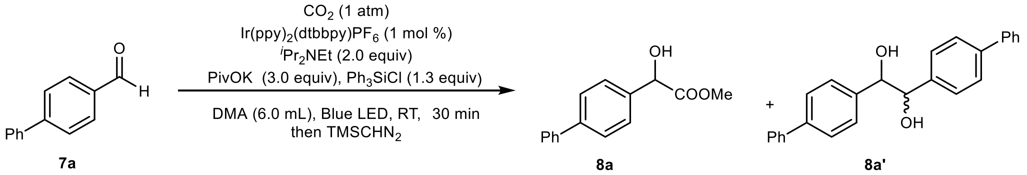

Entry	Derivation from standard conditions	**8a**[a]	**8a'**[a]	**7a**

1	As shown	60%	33%	n.d.
2	Without CO_2	n.d.	69%	n.d.
3	Without light	n.d.	n.d.	82%[b]
4	Without $Ir(ppy)_2(dtbbpy)PF_6$	n.d.	n.d.	94%[b]
5	Without $^iPr_2NEt$	7%	8%	65%[a]
6	Without Ph_3SiCl	8%	63%	22%[a]

[a]Isolated yields. [b]LC yield

Following general procedure for the carboxylation of aryl aldehydes, the control experiments show light, photocatalyst, $^iPr_2NEt$, Ph_3SiCl and CO_2 were critical factors for this transformation. Note that pinacols **8a'** was major byproduct in the absence of Ph_3SiCl.

Supplementary Table 6. Control experiments for carboxylation of **5g**.

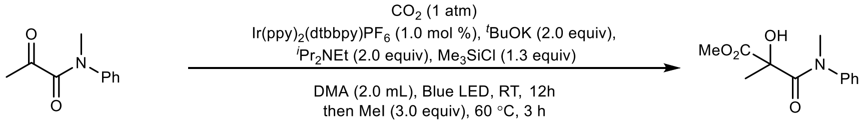

5g, 0.2 mmol

6g

Entry	Derivation from standard conditions	**The yield of 6g[a], 5g[b]**
1	As shown	55%, n.d.
2	Without CO_2	n.d., 0
3	Without light	n.d., 100%
4	Without $Ir(ppy)_2(dtbbpy)PF_6$	n.d., 100%
5	Without $^iPr_2NEt$	n.d., 54%
6	Without Me_3SiCl	43%, n.d.

[a]Isolated yields. [b]LC yield

Following general procedure for the carboxylation, the control experiments show light, photocatalyst, $^iPr_2NEt$ and CO_2 were critical factors for this transformation.

5. Mechanistic Studies

Control experiments with radical scavengers

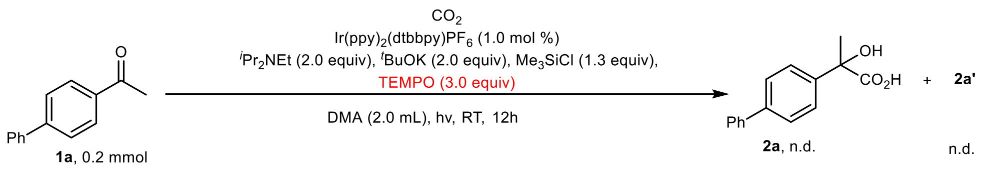

Figure S-1

Following the general procedures, the reaction of **1a** (0.2 mmol), Ir(ppy)$_2$(dtbbpy)PF$_6$ (1.0 mol %), ^{t}BuOK (2.0 equiv), ^{i}Pr$_2$NEt (2.0 equiv), TMSCl (1.3 equiv), TEMPO (0.4 mmol, 3.0 equiv) and DMA (2 mL) in the presence of CO_2 (closed, 1 atm) for 12 hours at room temperature afforded no desired product **2a or 2a'**, which indicates the existence of radical.

The effect of D_2O in the reaction

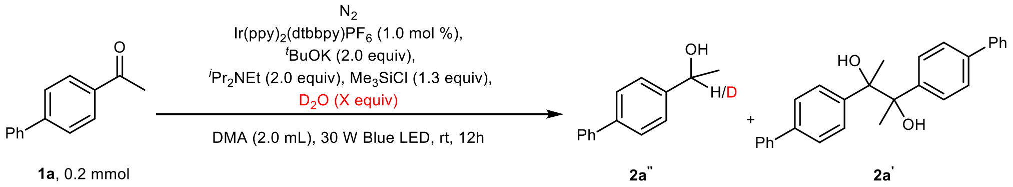

D_2O (X equiv)	**2a''**[a]	**1a**[a]	**2a'**[a]
X= 0	7%	22%	13%
X = 1.0	0	68%	1%
X = 10	0	72%	6%
X = 20	0	55%	25%

[a]LC yields.

Figure S-2

Following the general procedures, the reaction of **1a** (0.2 mmol), Ir(ppy)$_2$(dtbbpy)PF$_6$ (1.0 mol %), ^{t}BuOK (2.0 equiv), ^{i}Pr$_2$NEt$_2$ (2.0 equiv), TMSCl (1.3 equiv), D_2O (1.0 or 10 or 20 equiv) and DMA (2 mL) under N_2 atmosphere for 12 hours at room temperature, no **2a''** was detected. Increasing the amount of D_2O resulted in piancol **2a'** formation, which is presumably due to the moisture sensitivity of TMSCl.

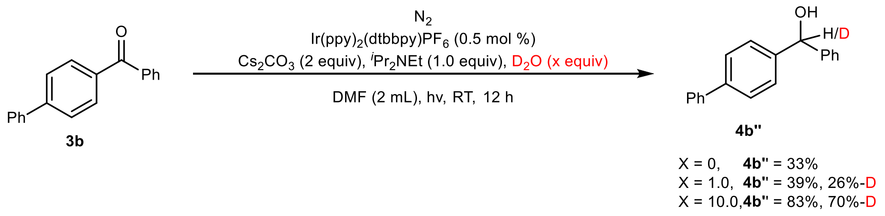

Figure S-3

Following the general procedures, the reaction of **3b** (0.2 mmol), $Ir(ppy)_2(dtbbpy)PF_6$ (0.5 mol %), Cs_2CO_3 (2.0 equiv), $^{i}Pr_2NEt$ (1.0 equiv), D_2O (1.0 equiv or 10.0 equiv) and DMF (2 mL) under N_2 atmosphere for 12 hours at room temperature afforded **4b”** as a colorless solid in 39% yield with 26% deuterium incorporation or 83% yield with 70% deuterium incorporation. This result indirectly confirmed the existence of benzylic carbanion. The ^{1}H-NMR spectrums were provided as below Supplementary Figure 1 and 2..

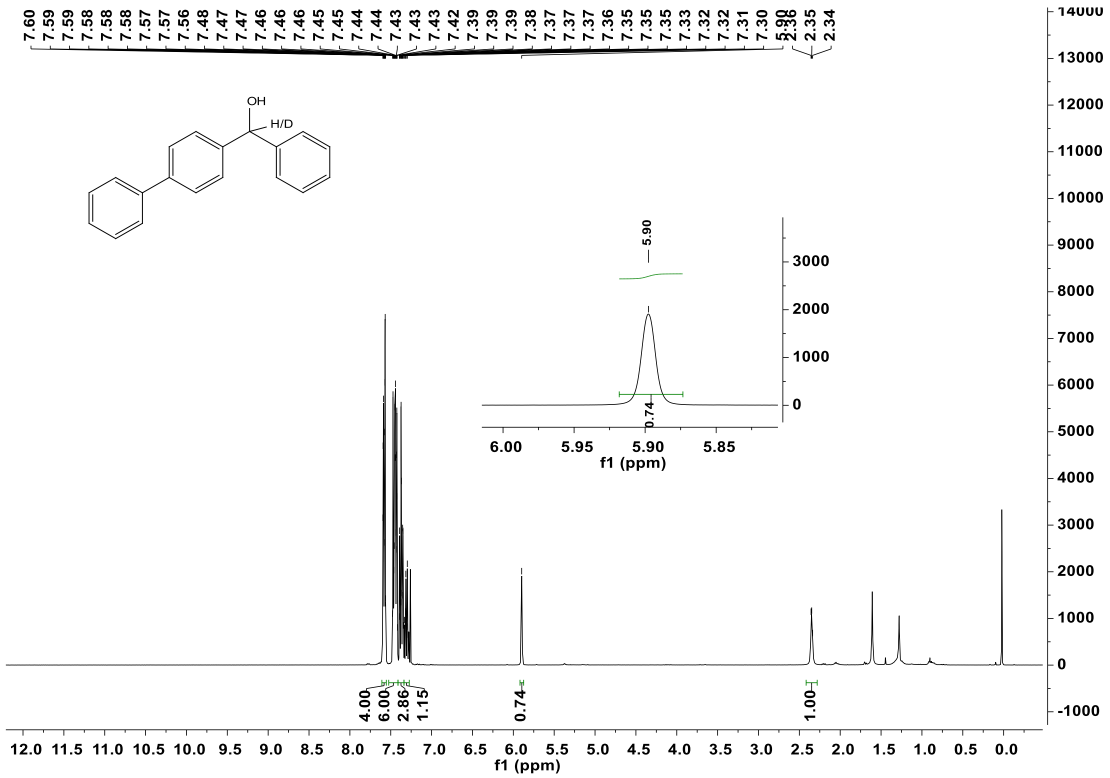

Supplementary Figure 1. ^{1}H NMR spectra of 26% deuterium compound **4b”**.

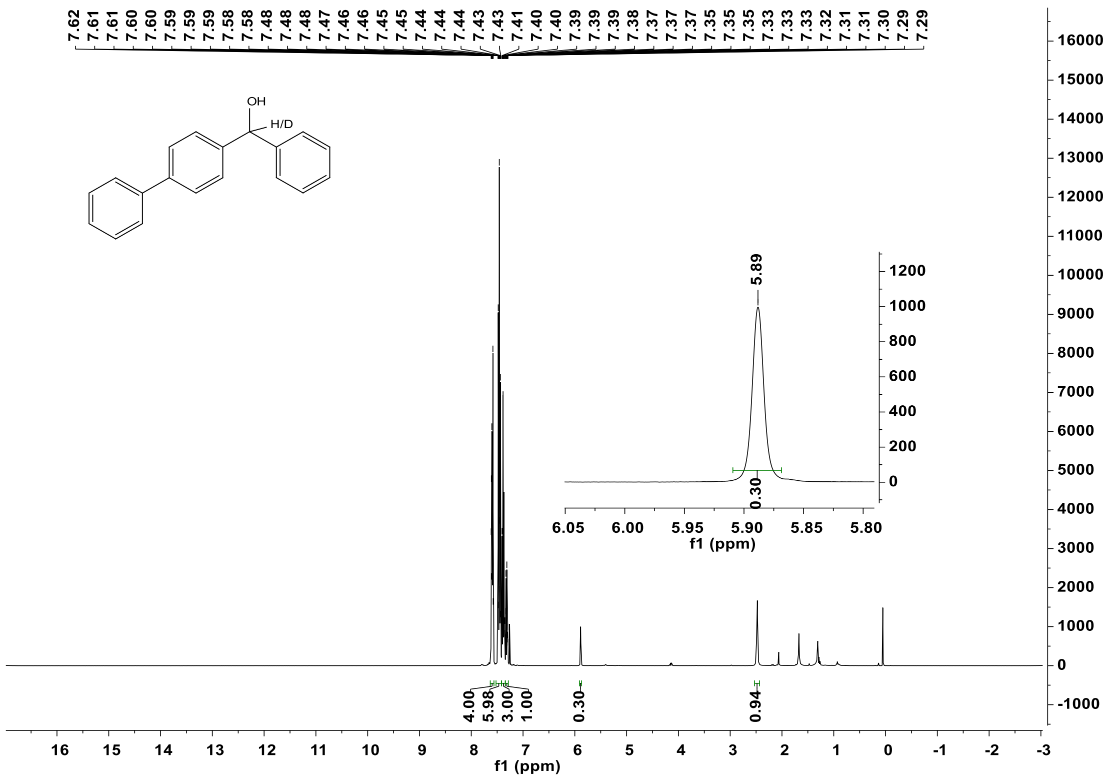

Supplementary Figure 2. ^{1}H NMR spectra of 70% deuterium compound **4b''**.

The possibility of sily enol ether 1A as intermediate.

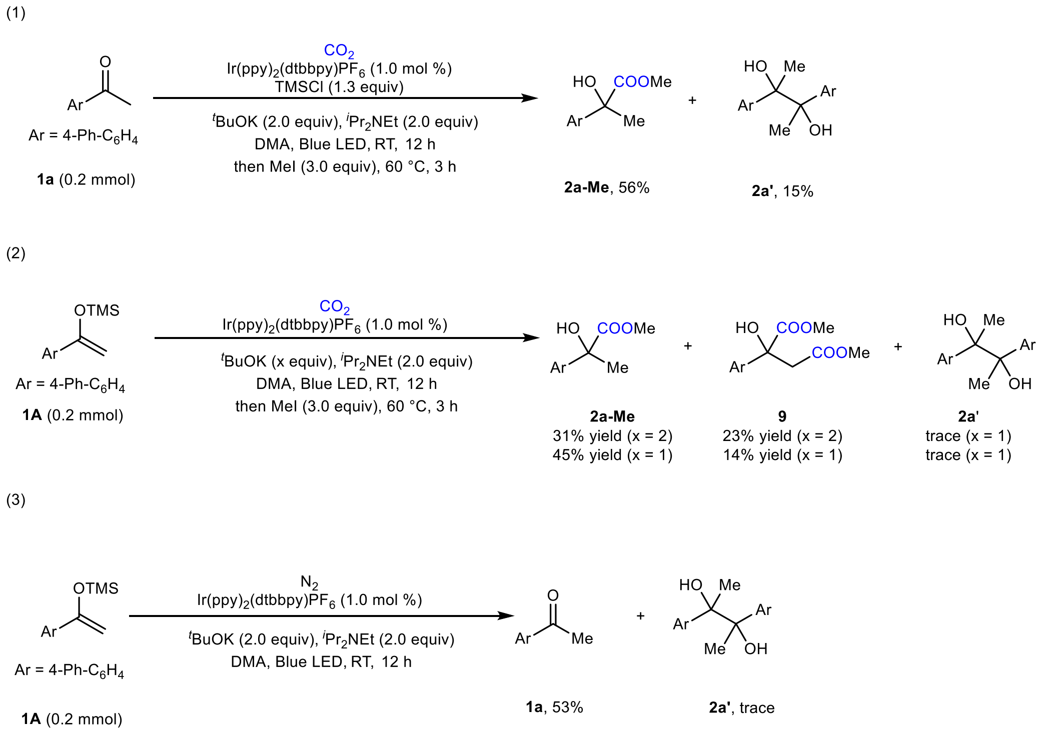

Figure S-4

When replacement of 4-acetylbiphenyl **1a** and TMSCl by the silyl enol ether 1**A** was conducted, **2a-**

Me was obtained in 31% yield, along with 23% yield of dicarboxylated product **9**, which was not detected under standard conditions (equiv 1). Considered the formation of **A** could consume one equivalent base, the reaction of **9** in presence of ^{t}BuOK (1.0 equiv) was tested to afford **2a-Me** in 14% yield. These results suggested the **A** was not intermediate in the catalytic cycle. In absence of TMSCl, we observed the full decomposition of **1A** under N_2 atmosphere to give **1a** in 53% yield, indicating that the formation of **2a** from **1A** and CO_2 might proceed with **1a** as the real reactant.

Characterization data of 2a-Me and 9

methyl 2-([1,1'-biphenyl]-4-yl)-2-hydroxypropanoate (2a-Me)

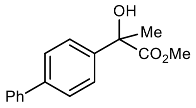

^{1}H NMR (400 MHz, $CDCl_3$) δ 7.69 – 7.54 (m, 6H), 7.47 – 7.39 (m, 2H), 7.38 – 7.29 (m, 1H), 3.81 (s, 1H), 3.80 (s, 3H), 1.82 (s, 3H). **^{13}C NMR** (101 MHz, $CDCl_3$) δ 176.11, 141.69, 140.78, 140.58, 128.79, 127.42, 127.11, 127.10, 125.68, 75.70, 53.36, 26.75. **HRMS (ESI$^+$)** calcd for $C_{16}H_{16}NaO_3^+$ $[M+Na]^+$: 279.0992, found 279.0995.

Dimethyl 2-([1,1'-biphenyl]-4-yl)-2-hydroxysuccinate (9)

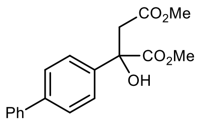

^{1}H NMR (400 MHz, $CDCl_3$) δ 7.67 – 7.55 (m, 6H), 7.47 – 7.41 (m, 2H), 7.38 – 7.32 (m, 1H), 4.41 (s, 1H), 3.83 (s, 3H), 3.74 (s, 3H), 3.50 (d, J = 16.6 Hz, 1H), 2.95 (d, J = 16.6 Hz, 1H). **^{13}C NMR** (101 MHz, $CDCl_3$) δ 174.20, 171.61, 141.19, 140.43, 139.09, 128.82, 127.53, 127.27, 127.13, 125.58, 76.20, 53.44, 52.09, 44.34. **HRMS (ESI$^+$)** calcd for $C_{18}H_{18}NaO_5^+$ $[M+Na]^+$: 337.1046, found 337.1043.

The possibility of pinacol 2a' as intermediate.

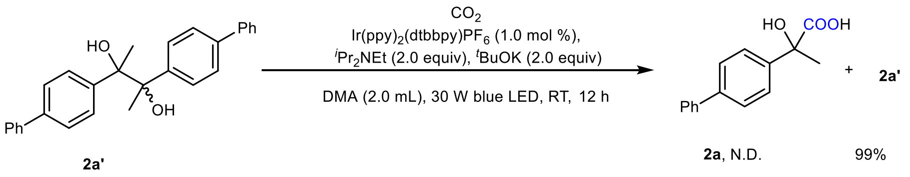

Figure S-5

When pinacol **2a'** was conducted in standard condition, **2a'** was recovered in 99% yield, indicating that the **2a'**was not intermediate.

The mechanistic possibility of 1B as brook rearrangement intermediate.

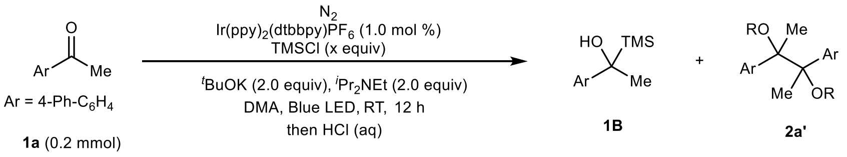

Entry	Condition	1B	1a[a]	2a'[a]
1	x = 2	n.d.	0	R = H, 81%
2	x = 3	n.d.	5%	R = H, 67%
3	x = 8	n.d.	23%[b]	R = H, 17%[b], R = TMS, 32%[b]

[a]LC yield. [b]Isolated yield

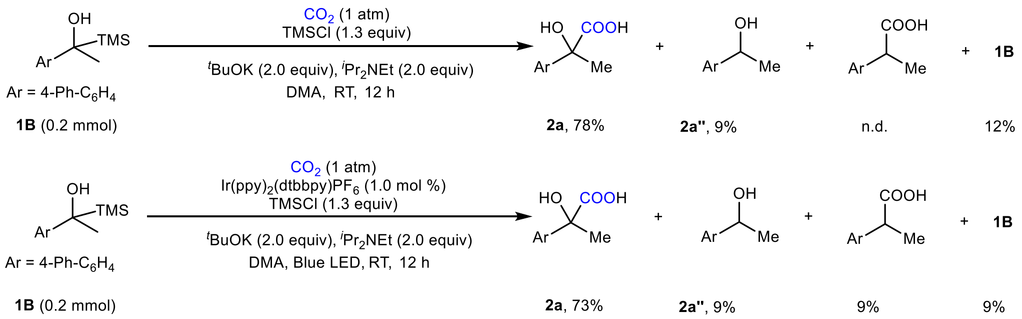

Figure S-6

Increasing the amount of TMSCl in standard condition in N_2 atmosphere, no C-silyl compound **1B** was detected by GC-MS. Meanwhile, reaction of pre-prepared **1B** was carried out in the absence of photocatalyst and blue light, **2a** was obtained in 78% isolated yield. When the carboxylation reaction of **1B** was performed in standard condition, the **2a** was obtained in 73% isolated yield, along with little amount of phenylacetic acid derivatives detected. Based on these results, the C-silylated compound might not be the reaction intermediate in this transformation. (Note: The order of reagent addition: dry 10 mL tube with Ir-phtocatalyst and ^{t}BuOK was evacuated and back-filled with CO_2 for 3 times, then solid **1B**, ${}^{i}Pr_2$NEt, TMSCl and DMA were added under CO_2 atmosphere.)

The possibility of alcohol and silylether of alcohol as intermediate.

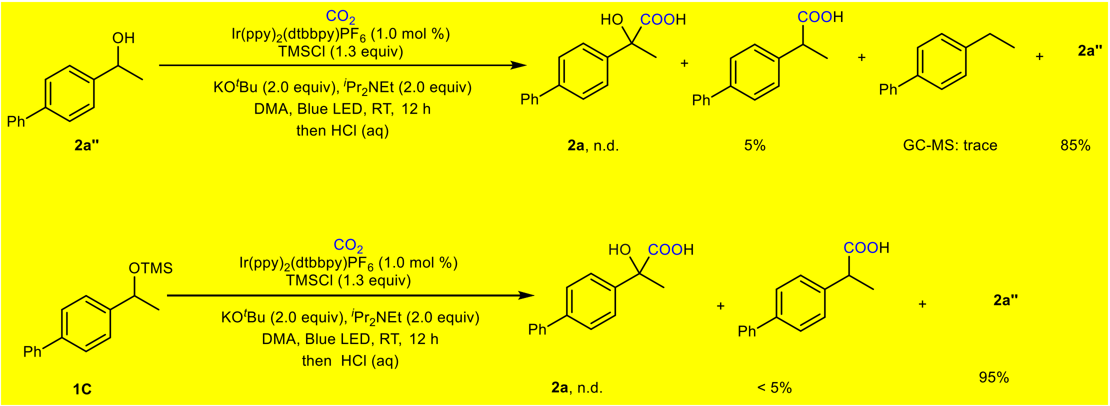

Figure S-7

Considering that the formation of carbanion could alternatively take a different mechanism: deprotonation of the reduced alcohol or silylether of alcohol. The **2a"** and **1C** were performed in standard conditions, no desired α-hydroxycarboxylic acids were detected, corresponding alcohols were remained along with little dehydroxycarboxylative products were detected. These results indicated this pathway was unlikely.

The detection of formate in standard condition

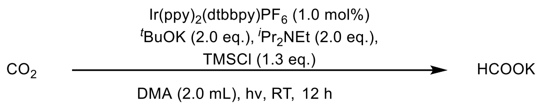

Entry	Derivation from standard condition	**TON**[a]
1	As shown	43
2	Without CO_2	4
3	Without TMSCl	43
4	Without $^{i}Pr_2NEt$	2
5	Without Light	2
6	Without $Ir(ppy)_2(dtbbpy)PF_6$	2
7	Without $^{t}BuOK$	n.d.
8	**1a** (0.2 mmol) as additive	2
9	DMSO instead of DMA	0

[a]TON = [HCOOK (mol)] / [$Ir(ppy)_2(dtbbpy)PF_6$ (mol)], the amount of HCOOK is determined by ^{1}H NMR with sodium *p*-toluenesulfonate as the internal standard. n.d.= not detected by ^{1}H NMR.

Figure S-8

The control experiments without CO_2, we found that the TON of formate was 4 using DMA as solvent. However, when using DMSO as solvent, no formate was detected by ^{1}H NMR. Moreover, no formate was detected in the absence of $^{t}BuOK$. These results indicate that the formation of trace formate might arise from the use of DMA in the presence of $^{t}BuOK$.

^{13}C-Labeling Experiments

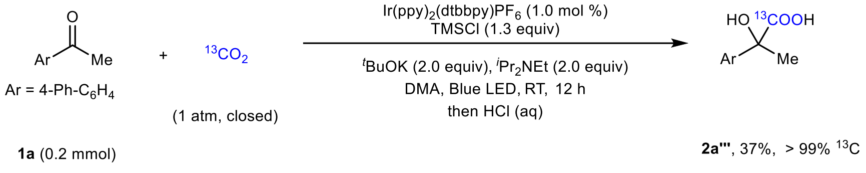

Figure S-9

The oven-dried Schlenk tube (10 mL) containing a stirring bar was charged with **1a** and $Ir(ppy)_2(dtbbpy)PF_6$, then added $^{t}BuOK$ in glovebox. The tube was taken out, evacuated and back-filled with N_2 for 3 times. Subsequently, $^{i}Pr_2NEt$, TMSCl and DMA was added via syringe under N_2 atmosphere. The resulting mixture was degassed by using a "freeze–pump–thaw" procedure and

then injected $^{13}CO_2$ through controlling cylinder. Then The reaction was stirred in water bath and irradiated with a 30 W blue LED lamp (3 cm away, with cooling fan to keep the reaction temperature at 25~30 °C) for 12 h. After completion, 0.5 mL $^{n}Bu_4NF$ (1.0 N in THF) was carefully added to quench the reaction, the mixture was allowed to stir for 30 min at room temperature. The reaction was quenched by 2 mL HCl (2 N), stirred for 10 min, and diluted with 2.5 mL EtOAc. The reaction mixture was extracted by EtOAc and the combined organic phases were concentrated *in vacuo*. The residue was purified by silica gel flash column chromatography to give the pure desired product in 37% yield. The 13C-content was determined to be 99% by HRMS (ESI-).

HRMS of ^{13}Carbonyl-labeled **2a**

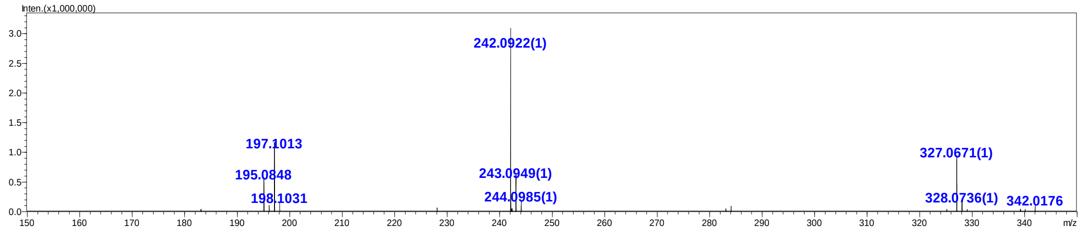

m/z	Intensity	Theo. Mass	Composition
n.d.	–	241.0870	$C_{15}H_{13}O_3^-$
242.0922	1351325	242.0904	$C_{14}{}^{13}CH_{13}O_3^-$

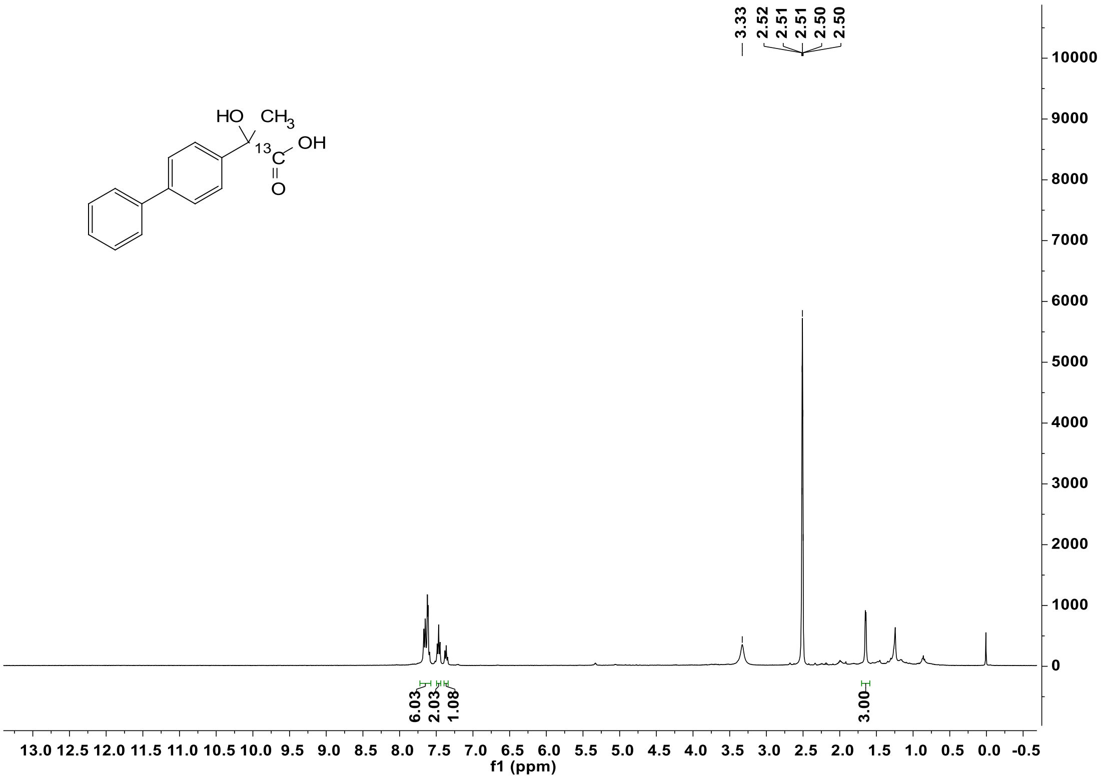

Supplementary Figure 3. ^{1}H NMR spectra of ^{13}Carbonyl-labeled **2a**.

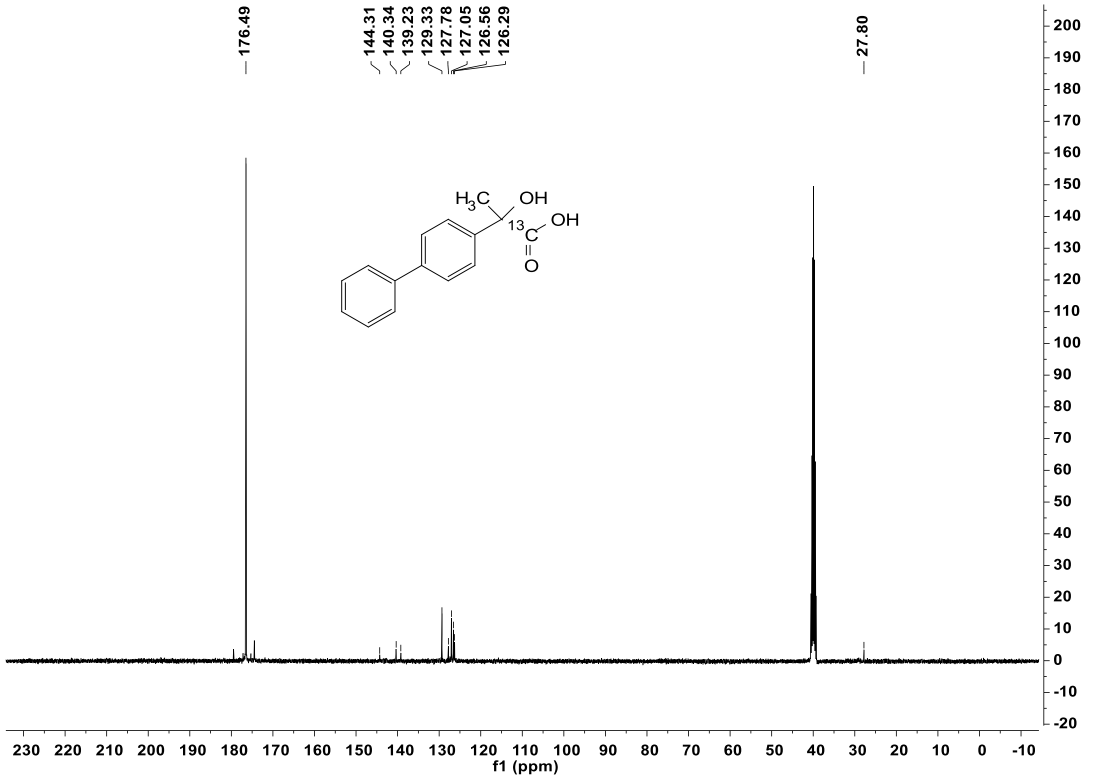

Supplementary Figure 4. ^{13}C NMR spectra of ^{13}Carbonyl-labeled **2a**.

Luminescence quenching experiments

Fluorescence quenching experiments were measured on a RF-5301PC Spectrofluorophotometer. Anhydrous DMA was degassed by N_2 bubbling for 30 minutes before using. The complex Ir(ppy)$_2$(dtbbpy)PF$_6$ was excited at 455 nm and the emission spectrum λ_{max} = 570 nm was recorded. In a typical experiment, in glovebox, increasing amount of **1a** (0.2 mmol of **1a** in 2 mL DMA), TMSCl (0.1 mmol of TMSCl in 1 mL DMA), and $^{i}Pr_2NEt$ (0.1 mmol of $^{i}Pr_2NEt$ in 1 mL DMA) were added to a 2.0 mL (5.0×10^{-5} M) solution of Ir(ppy)$_2$(dtbbpy)PF$_6$ in DMA in the 4.0 mL quartz cuvette (d = 1 cm) and covered with Teflon cap. Then the emission spectrum of the solution was collected at each addition.

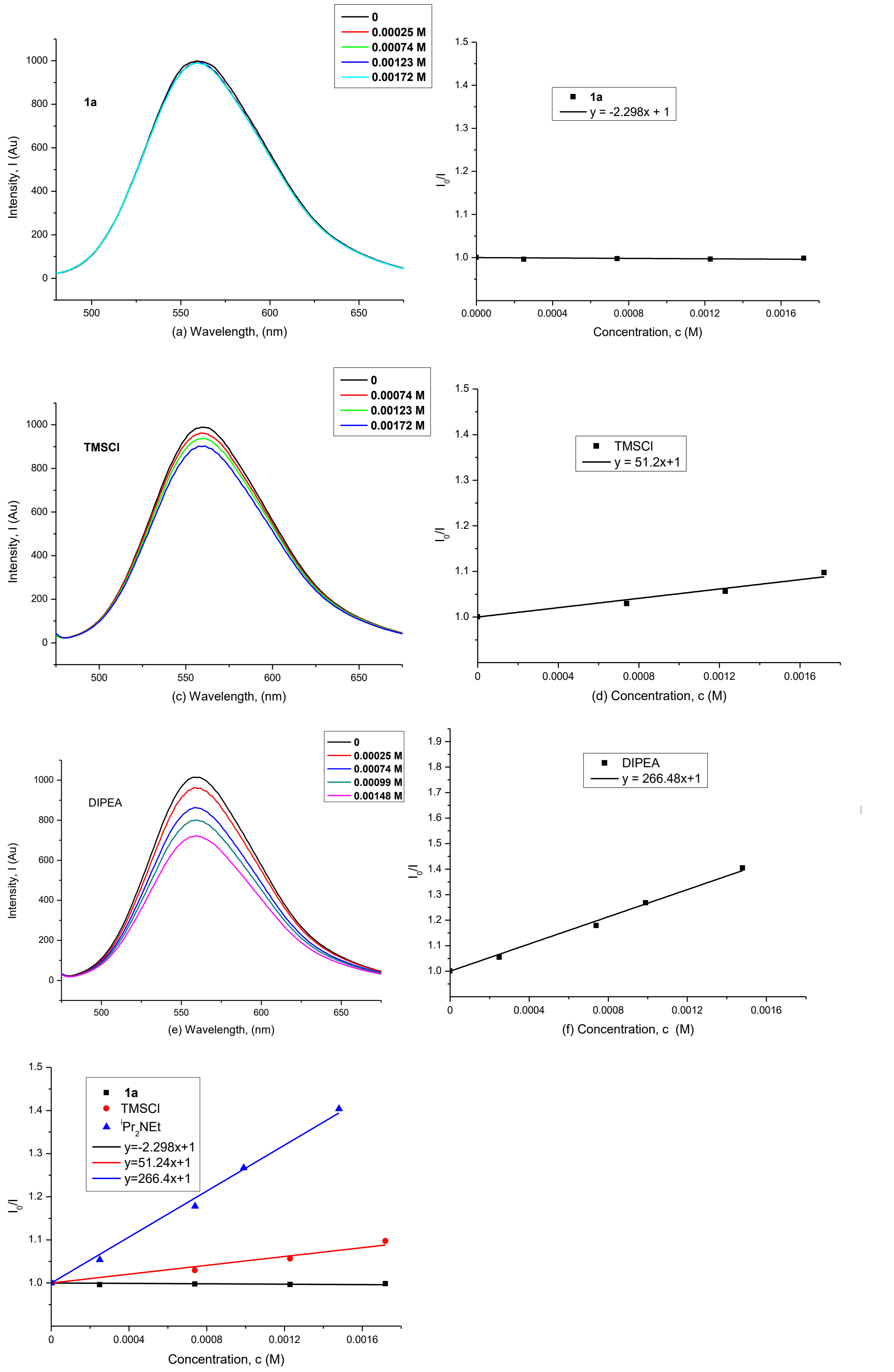

Figure S-10

The luminescence of Ir(ppy)$_2$(dtbbpy)PF_6 at λ_{max} = 570 nm was readily quenched with $^{i}Pr_2NEt$

with a slope of 266.4, while **1a** and TMSCl just with a quenching slope of -2.3 and 51.2. These results suggested that the reaction proceed with reductive quenching to give reduced Ir^{II}-catalyst.

Cyclic voltammetry test

Electrochemical studies were carried out with a CHI600E electrochemical workstation. All cyclic voltammograms were measured at room temperature using Ag/Ag^+ (10 mM $AgNO_3$ in MeCN) reference electrode, a platinum (Pt) wire counter and working electrode made of a glassy carbon disk. The conditions of the experiments were as follows: testing compounds are in solution of 100 mM tetrabutylammonium tetrafluoroborate ($^nBu_4NBF_4$) in DMF and a scan rate of 100 mV/s. Prior to each measurement, solutions were purged with N_2 for 10 min to ensure the oxygen-free conditions. According to redox couple ferrocene/ferrocenium is +0.48 V versus SCE, *E* (vs SCE) = *E* (vs $Ag/AgNO_3$) + 0.33 V.

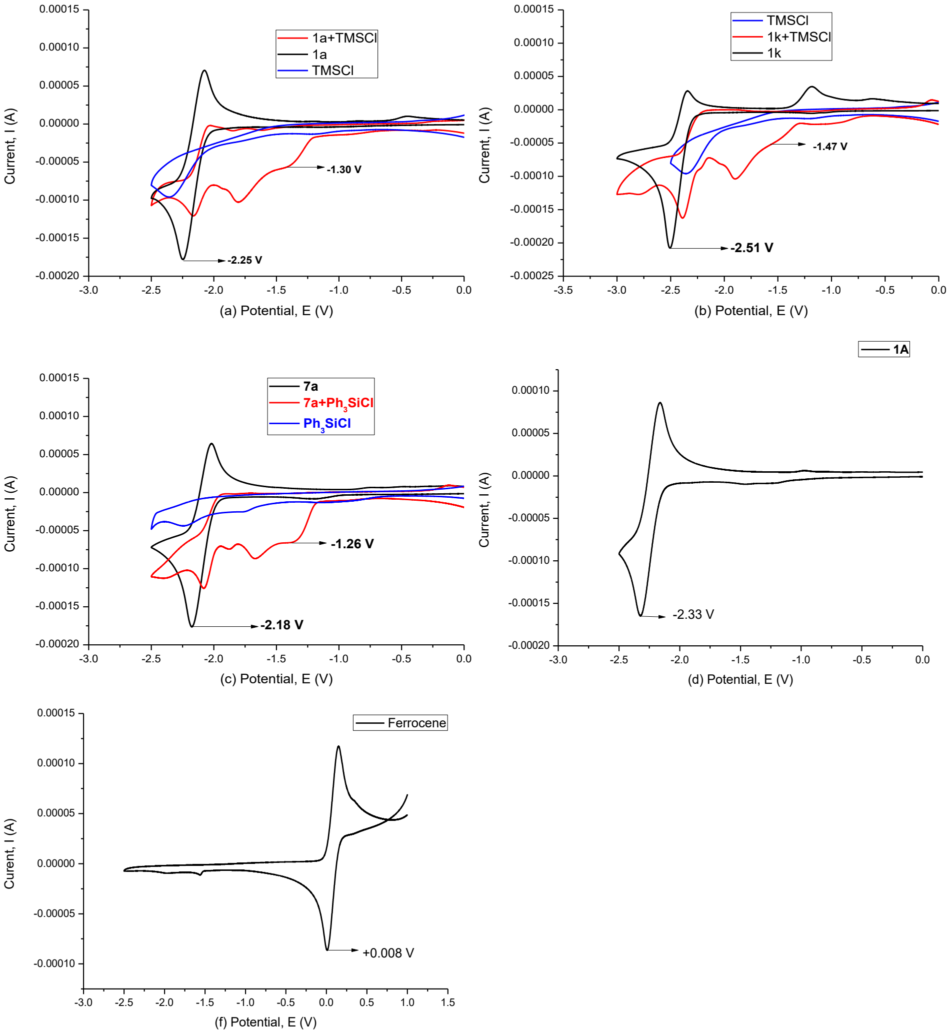

Figure S-11 Cyclic voltammograms: (a) **1a** (0.05 mmol of **1a** in 5 mL DMF), TMSCl (0.05 mmol

of TMSCl in 5 mL DMF) and **1a** in presence of TMSCl (0.05 mmol of **1a** by adding 0.065 mmol of TMSCl in 5 mL DMF under N_2; (b) **1k** (0.05 mmol of **1k** in 5 mL DMF), TMSCl (0.05 mmol of TMSCl in 5 mL DMF) and **1k** in presence of TMSCl (0.05 mmol of **1k** by adding 0.065 mmol of TMSCl in 5 mL DMF) under N_2; (c) **7a** (0.05 mmol of **7a** in 5 mL DMF), Ph_3SiCl (0.05 mmol of Ph_3SiCl in 5 mL DMF) and **7a** in presence of Ph_3SiCl (0.05 mmol of **7a** by adding 0.065 mmol of Ph_3SiCl in 5 mL DMF) under N_2. (d) **1A** (0.05 mmol of **1A** in 5 mL DMF); (e) Ferrocene (0.05 mmol of ferrocene in 5 mL DMF).

A variety of cyclic voltammetry (CV) studies were conducted in order to investigate the redox potentials of various chemicals. $E_{1/2}^{red}$ of **1a**, **1k**, **6a**, **1A** were -1.81 V vs SCE, -2.1 V vs SCE, -1.76 V vs SCE, -1.91 V vs SCE. As carbonyl compounds might be activated by Lewis-acidic TMSCl, we tested ketones **1a** and **1k**, aldehyde **7a** in presence of TMSCl. As pictures show, the decrease of reduction potentials of ketones in presence of TMSCl could be observed obviously, indicating that TMSCl-activated ketone might undergo easier SET with reduced $[Ir]^{II}$.

6. The gram-scale reaction and synthetic application

The gram-scale synthesis of 6a

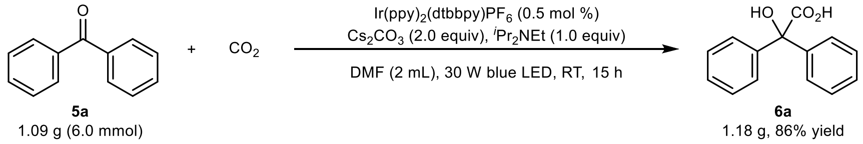

Figure S-12

Following the general procedure for the carboxylation of diaryl ketones, the oven-dried Schlenk tube (250 mL) containing a stirring bar was charged with **5a** (1.09 g, 6 mmol), $Ir(ppy)_2(dtbbpy)PF_6$ (30 mg, 0.03 mmol, 0.5 mol%), Cs_2CO_3 (3.9 g, 12 mmol, 2 equiv), $^iPr_2NEt$ (1.0 mL, 6 mmol, 1.0 equiv) and DMF (60 mL) under CO_2 for 15 h afforded **6a** as a white solid (1.18 g, 86% yield).

Synthetic applications of 2i or 8a

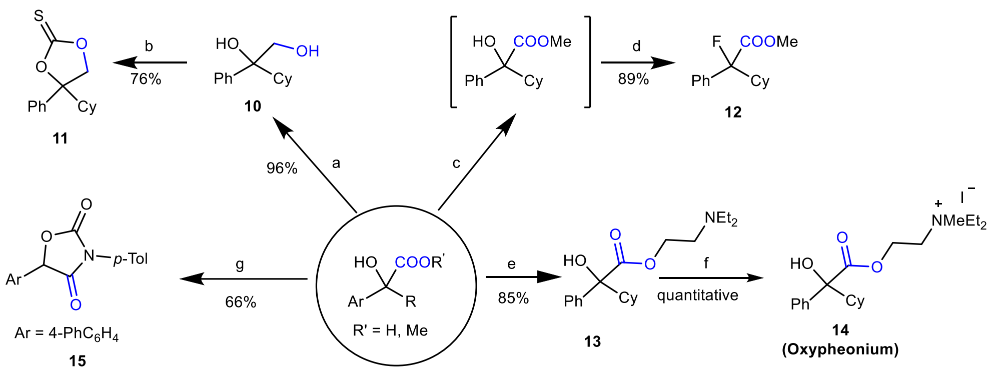

Figure S-13

Synthesis of 10

To a suspension of $LiAlH_4$ (74 mg, 1.95 mmol) in dry THF (3 mL) at 0 °C was slowly added a solution of 2-cyclohexyl-2-hydroxy-2-phenylethanoic acid **2i** (117 mg, 0.5 mmol) in dry THF (3 mL). The suspension was refluxed for 2 h, and then quenched with water at RT. To the mixture was added with stirring a saturated aqueous solution of potassium sodium tartrate until the mixture became clear. The aqueous layer was extracted with DCM, and the extracts were dried over anhydrous Na_2SO_4 and concentrated. The residue was purified on a short silica gel column, yielding 1-cyclohexyl-1-phenylethane-1,2-diol **10** (98.7 mg, 92%) as a white solid.

1-cyclohexyl-1-phenylethane-1,2-diol (10)

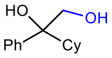

98.7 mg, 92% yield; white solid;

R_f = 0.5 (PE/EA 2/1);

Mp: 89-90 °C;

^{1}H NMR (400 MHz, $CDCl_3$) δ 7.47 – 7.34 (m, 4H), 7.33 – 7.28 (m, 1H), 4.04 (d, J = 11.2 Hz, 1H), 3.88 (d, J = 11.2 Hz, 1H), 2.63 (s, 1H), 1.92 – 1.58 (m, 5H), 1.54 – 1.40 (m, 2H), 1.31 – 1.11 (m, 2H), 1.09 – 0.99 (m, 2H). **^{13}C NMR** (101 MHz, $CDCl_3$) δ 143.07, 128.22, 126.97, 126.16, 79.22, 68.21, 45.56, 27.22, 26.85, 26.61, 26.48, 26.32. **HRMS (ESI$^+$)** calcd for $C_{14}H_{20}NaO_2^+$ [M+Na$^+$]: 243.1356; found 243.1352.

Synthesis of 11

To a stirred solution of 1-phenylethylene-1-cyclohexyl glycol **11** (44 mg, 0.2 mmol) in dry DCM (2 mL) was added 1,1'-thiocarbonyldiimidazole (53.4 mg, 0.3 mmol) in one portion. The

mixture was stirred for 6 hours at room temperature. The organic layer was washed with water, dried over $MgSO_4$ and finally concentrated under vacuum. The resulting crude material was purified by column chromatography to give the desired product 11 as a white solid (40.1 mg, 76% yield).

4-cyclohexyl-4-phenyl-1,3-dioxolane-2-thione (11)

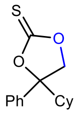

40.1 mg, 76% yield; White solid;

R_f = 0.35 (PE/EA 5/1);

Mp: 67-69 °C;

^{1}H NMR (400 MHz, $CDCl_3$) δ 7.44 – 7.33 (m, 3H), 7.27 – 7.22 (m, 2H), 4.86 (d, J = 8.7 Hz, 1H), 4.68 (d, J = 8.7 Hz, 1H), 1.94 – 1.71 (m, 4H), 1.69 – 1.62 (m, 1H), 1.57 – 1.48 (m, 1H), 1.36 – 0.86 (m, 5H). **^{13}C NMR** (101 MHz, $CDCl_3$) δ 191.21, 138.81, 128.71, 128.53, 124.84, 95.15, 76.92, 47.04, 26.83, 26.33, 25.97, 25.83, 25.77. **HRMS (ESI$^+$)** calcd for $C_{15}H_{18}NaO_2^+$ [M+Na$^+$]: 273.1261; found 273.1258.

Synthesis of 12

2i was dissolved in 2 mL MeOH/Et_2O (0.5/1.5) and then $TMSCHN_2$ (0.25 mL, 0.5 mmol, 2 M in hexanes) was added dropwisely at 0 °C. The mixture was stirred at ambient temperature until the completion of the methylation reaction. Concentrated to dryness to obtain an oil which was used in the next step without further purification. To a solution of the crude ester 1i' in dry CH_2Cl_2 (2 mL) was added slowly Deoxofluor (0.12 mL, 133 mg, 0.6 mmol) in dry CH_2Cl_2 (3 mL) at room temperature under nitrogen atmosphere. Saturated aqueous $NaHCO_3$ solution (3 mL) and H_2O (3 mL) were added to the reaction mixture. The solution was extracted with CH_2Cl_2. The organic layer was evaporated and the residue was purified by column chromatography to give the fluoroester 12 (44.6 mg, 0.178 mmol, 89%) as a colorless oil.

methyl 2-cyclohexyl-2-fluoro-2-phenylacetate (12)

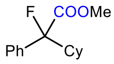

44.6 mg, 89% yield; Colorless oil;

R_f = 0.32 (PE/EA 50/1);

^{1}H NMR (400 MHz, $CDCl_3$) δ 7.54 – 7.48 (m, 2H), 7.39 – 7.34 (m, 2H), 7.33 – 7.28 (m, 1H), 3.75 (s, 3H), 2.38 – 2.21 (m, 1H), 1.85 – 1.77 (m, 1H), 1.71 – 1.59 (m, 3H), 1.52 – 1.39 (m, 1H), 1.36 – 1.23 (m, 2H), 1.19 – 1.03 (m, 3H). ^{13}C NMR (101 MHz, $CDCl_3$) δ 171.24 (d, J = 26.8 Hz), 137.31

(d, J = 22.6 Hz), 128.32 (d, J = 2.4 Hz), 128.11 , 124.84 (d, J = 10.3 Hz), 99.56 (d, J = 194.7 Hz), 52.65 , 45.25 (d, J = 21.1 Hz), 27.11 (d, J = 2.9 Hz), 26.24 , 26.13 , 26.01 , 25.28 (d, J = 2.6 Hz). ^{19}F NMR (376 MHz, $CDCl_3$) δ -179.27. GC-MS (EI) calcd for $C_{15}H_{19}FO_2$ [M]: 250.1 ; found 250.2.

Synthesis of 13

The oven-dried Schlenk tube (10 mL) containing a stirring bar was charged with 2-cyclohexyl-2-hydroxy-2-phenylethanoic acid **2i** (117 mg, 0.5 mmol), Na_2CO_3 (105 mg, 1 mmol), diethylamine (0.2 mL, 5 mmol) in DCE (2 mL) was heated at 80 °C overnight. The reaction mixture was diluted with water, basified with 2 M NaOH to pH 12 and extracted with DCM. The solvent was removed under vacuum and the residue was purified by column chromatography to give the desired product 13 as a thick oil (141.7 mg, 85%).

2-(diethylamino)ethyl (R)-2-cyclohexyl-2-hydroxy-2-phenylacetate (13)

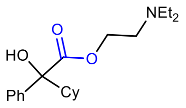

141.7 mg, 85% yield; Colorless oil;

R_f = 0.3 (DCM/MeOH 30/1);

^{1}H NMR (400 MHz, $CDCl_3$) δ 7.75 – 7.58 (m, 2H), 7.41 – 7.31 (m, 2H), 7.30 – 7.26 (m, 1H), 4.29 (dt, J = 11.2, 6.2 Hz, 1H), 4.19 (dt, J = 11.2, 6.0 Hz, 1H), 3.75 (s, 1H), 2.79 – 2.66 (m, 2H), 2.55 (q, J = 7.1 Hz, 4H), 2.31 – 2.19 (m, 1H), 1.86 – 1.78 (m, 1H), 1.71 – 1.61 (m, 2H), 1.58 – 1.50 (m, 1H), 1.50 – 1.40 (m, 1H), 1.37 – 1.27 (m, 1H), 1.25 – 1.08 (m, 4H), 1.02 (t, J = 7.1 Hz, 6H). ^{13}C NMR (101 MHz, $CDCl_3$) δ 175.61, 140.83, 127.99, 127.30, 126.06, 80.97, 64.65, 51.09, 47.37, 45.70, 27.37, 26.40, 26.38, 26.21, 25.53, 11.99. HRMS (ESI$^+$) calcd for $C_{21}H_{34}NO_3^+$[M+Na$^+$]: 348.2533; found 348.2524.

Synthesis of 14

13 (131 mg, 0.39 mmol) was dissolved in CH_3CN (5 mL), and MeI (93.4 μL, 1.5 mmol) was added to the mixture, then stirred for 3 h at 65 °C. The solvent was removed under vacuum and the residue was purified by column chromatography to give the desired product 14 as a pale yellow solid (183.8 mg, quantitative).

2-(2-cyclohexyl-2-hydroxy-2-phenylacetoxy)-*N*, *N*-diethyl-*N*-methylethan-1-aminium iodide (14)

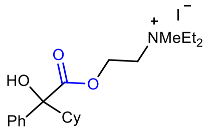

183.8 mg, quantitative yield; Pale yellow solid;

R_f = 0.14 (DCM/MeOH 10/1);

Mp: 51-53 °C;

^{1}H NMR (400 MHz, $CDCl_3$) δ 7.67 – 7.57 (m, 2H), 7.38 (t, *J* = 7.6 Hz, 2H), 7.29 (d, *J* = 7.8 Hz, 2H), 4.78 – 4.58 (m, 2H), 4.07 – 3.92 (m, 2H), 3.82 (s, 1H), 3.48 – 3.30 (m, 4H), 3.10 (s, 3H), 2.36 – 2.24 (m, 1H), 1.89 – 1.76 (m, 1H), 1.67 (d, *J* = 10.0 Hz, 2H), 1.51 – 1.31 (m, 3H), 1.28 – 1.20 (m, 8H), 1.19 – 1.05 (m, 3H). ^{13}C NMR (101 MHz, $CDCl_3$) δ 174.60, 139.88, 128.46, 127.87, 126.14, 81.49, 77.26, 59.50, 59.18, 57.75, 57.72, 48.27, 45.03, 27.66, 26.32, 26.20, 26.11, 25.52, 8.44. HRMS (ESI$^+$) calcd for $C_{21}H_{34}NO_3^+$[M+Na$^+$]: 348.2533; found 348.2524.

Synthesis of 15

To a stirred solution of methyl 2-([1,1'-biphenyl]-4-yl)-2-hydroxyacetate **8a** (48 mg, 0.2 mmol) and *N*,*N*-dimethylaminopyridine (48 mg, 0.4 mmol) in THF (4 mL) was added a solution of *p*-tolyl isocyanate (75 mg, 0.6 mmol) in THF (2 mL) at room temperature. After stirring for 6 h at 50 °C, the mixture was diluted with EtOAc and sat. $NaHCO_3$. The aq. layer was extracted with EtOAc. The combined organic extracts were concentrated under vacuum. The residue was purified by column chromatography to give a white solid 15 (45.2 mg, 66% yield).

5-([1,1'-biphenyl]-4-yl)-3-(p-tolyl)oxazolidine-2,4-dione (15)

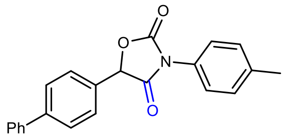

45.2 mg, 66% yield; White solid;

R_f = 0.25 (PE/EA 5/1);

Mp: 139-141 °C;

^{1}H NMR (400 MHz, $CDCl_3$) δ 7.76 – 7.68 (m, 2H), 7.66 – 7.58 (m, 4H), 7.54 – 7.47 (m, 2H), 7.46 – 7.39 (m, 1H), 7.40 – 7.30 (m, 4H), 5.95 (s, 1H), 2.43 (s, 3H). ^{13}C NMR (101 MHz, $CDCl_3$) δ 170.17, 154.12, 142.92, 140.10, 139.27, 130.45, 130.05, 128.95, 128.15, 127.92, 127.90, 127.22, 126.59, 125.53, 79.83, 21.24. HRMS (ESI$^+$) calcd for $C_{22}H_{17}NNaO_3^+$ [M+Na$^+$]: 366.1101 ; found 366.1100.

7. Supplementary References

1 Nakamura, A., Lectard, S., Hashizume, D., Hamashima Y. & Sodeoka, M. Diastereo- and Enantioselective Conjugate Addition of α-Ketoesters to Nitroalkenes Catalyzed by a Chiral $Ni(OAc)_2$ Complex under Mild Conditions. *J. Am. Chem. Soc.* **132**, 4036-4037 (2010).

2 Zhu, J., Yuan, Y., Wang S. & Yao, Z.-J. Synthesis of 2,3-Dialkylated Tartaric Acid Esters via Visible Light Photoredox-Catalyzed Reductive Dimerization of α-Ketoesters. *ACS Omega* **2**, 4665-4677 (2017).

3 Chiba, S., Zhang, L. & Lee, J.-Y. Copper-Catalyzed Synthesis of Azaspirocyclohexadienones from α-Azido-N-arylamides under an Oxygen Atmosphere. *J. Am. Chem. Soc.* **132**, 7266–7267 (2010).

4 Clayden, J., Watson D. W. & Chambers, M. Can relief of ring-strain in a cyclopropylmethyllithium drive the Brook rearrangement? *Tetrahedron*, **61**, 3195-3203 (2005).

5 Brook, A. G., Quigley, M. A., Peddle, G. J. D., Schwartz, N. V. & Warner, C. M. The Spectral and Chemical Properties of α-Silyl Ketones. *J. Am. Chem. Soc.* **82**, 5102-5106 (1960).

6 Mir, R. & Dudding, T. Phase-Transfer Catalyzed O-Silyl Ether Deprotection Mediated by a Cyclopropenium Cation. *J. Org. Chem.* **82**, 709-714 (2017).

7 Blay, G. et al. Nucleophilic benzoylation using lithiated methyl mandelate as a synthetic equivalent of the benzoyl carbanion. Oxidative decarboxylation of α-hydroxyacids. *Tetrahedron* **57**, 1075-1081 (2001).

8 Kim, S. M., Kim, D. W. & Yang, J. W. Transition-Metal-Free and Chemoselective $NaO^tBu–O_2$-Mediated Oxidative Cleavage Reactions of vic-1,2-Diols to Carboxylic Acids and Mechanistic Insight into the Reaction Pathways. *Org. Lett.* **16**, 2876-2879 (2014).

9 Bockman, T. M., Hubig, S. M. & Kochi, J. K. Direct Observation of Ultrafast Decarboxylation of Acyloxy Radicals via Photoinduced Electron Transfer in Carboxylate Ion Pairs. *J. Org. Chem.* **62**, 2210-2221(1997).

10 Prat, M. et al. Discovery of Novel Quaternary Ammonium Derivatives of (3R)-Quinuclidinol Esters as Potent and Long-Acting Muscarinic Antagonists with Potential for Minimal Systemic Exposure after Inhaled Administration: Identification of (3R)-3-{[Hydroxy(di-2-thienyl)acetyl]oxy}-1-(3-phenoxypropyl)-1-azoniabicyclo[2.2.2]octane Bromide (Aclidinium Bromide). *J. Med. Chem.* **52**, 5076-5092 (2009).

11 Sugayaa, M., Yamamotob, T. & Shinozaki, H. Palladium catalyzed synthesis of mandelate

derivatives from arylboronic acids and glyoxylate hemiacetals. *Tetrahedron Lett.* **58**, 2495–2497 (2017).

8. ^{1}H NMR, ^{13}C NMR and ^{19}F NMR Spectra

2-([1,1'-biphenyl]-4-yl)-2-hydroxypropanoic acid (2a)

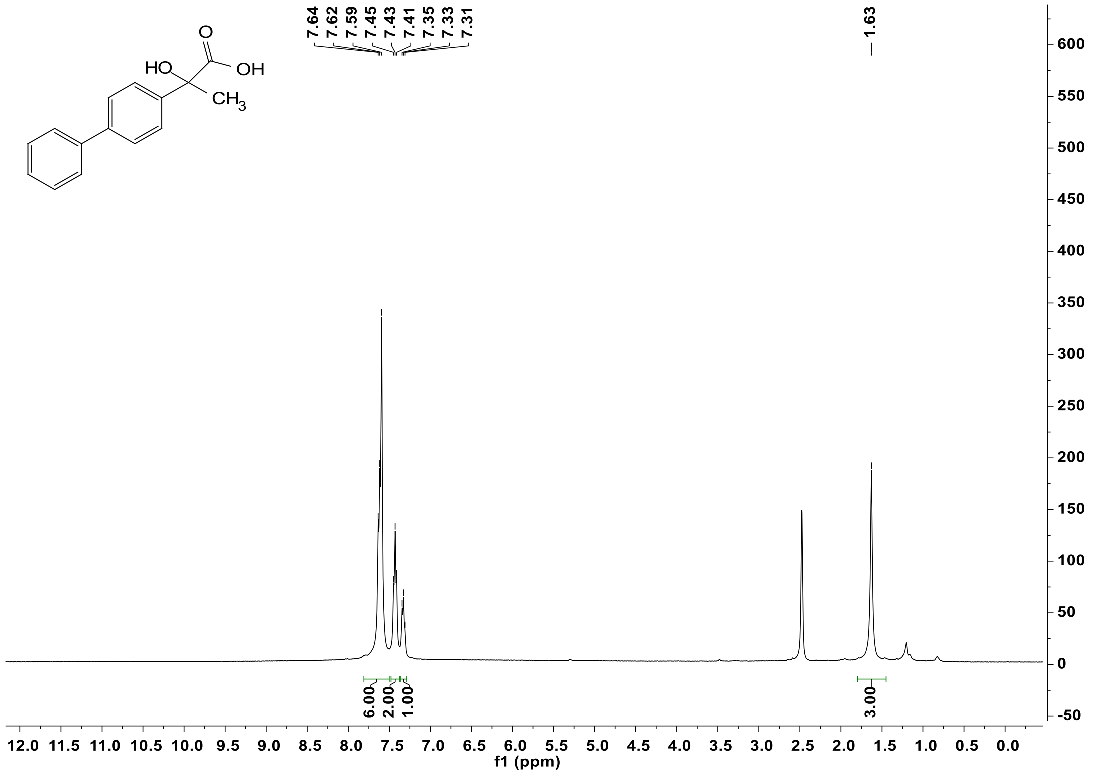

Supplementary Figure 5. ^{1}H NMR spectra of compound **2a**

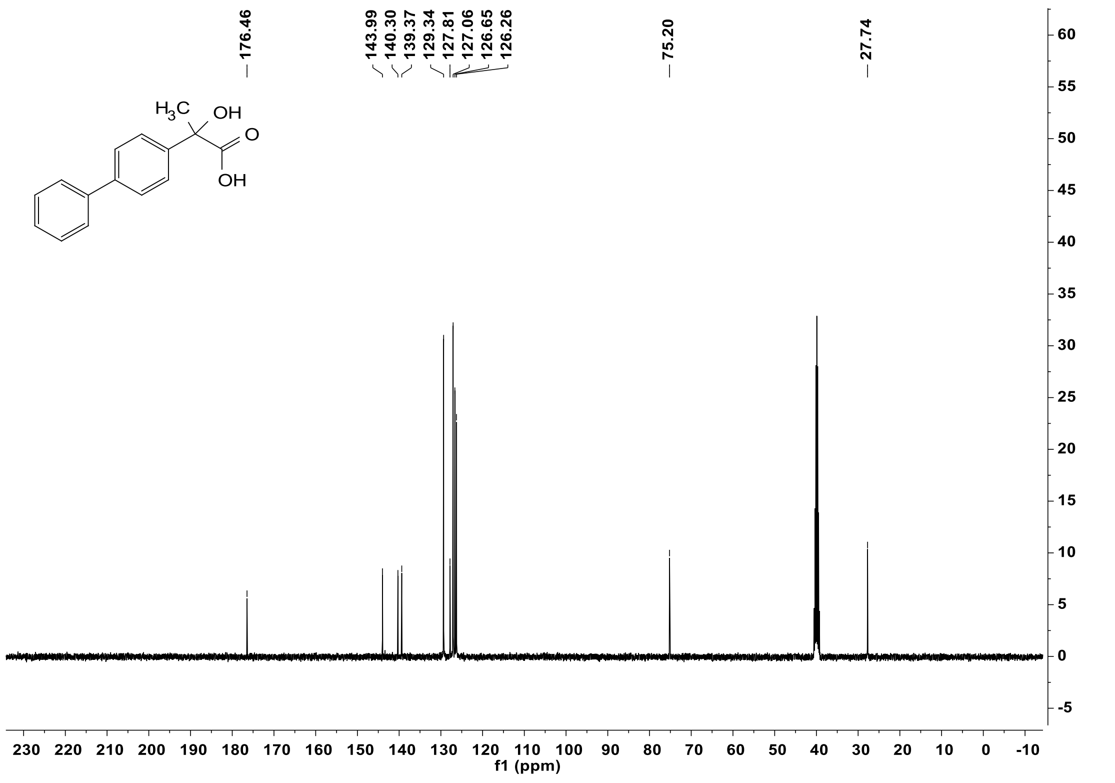

Supplementary Figure 6. ^{13}C NMR spectra of compound **2a**.

2-([1,1'-biphenyl]-4-yl)-2-hydroxybutanoic acid (2b)

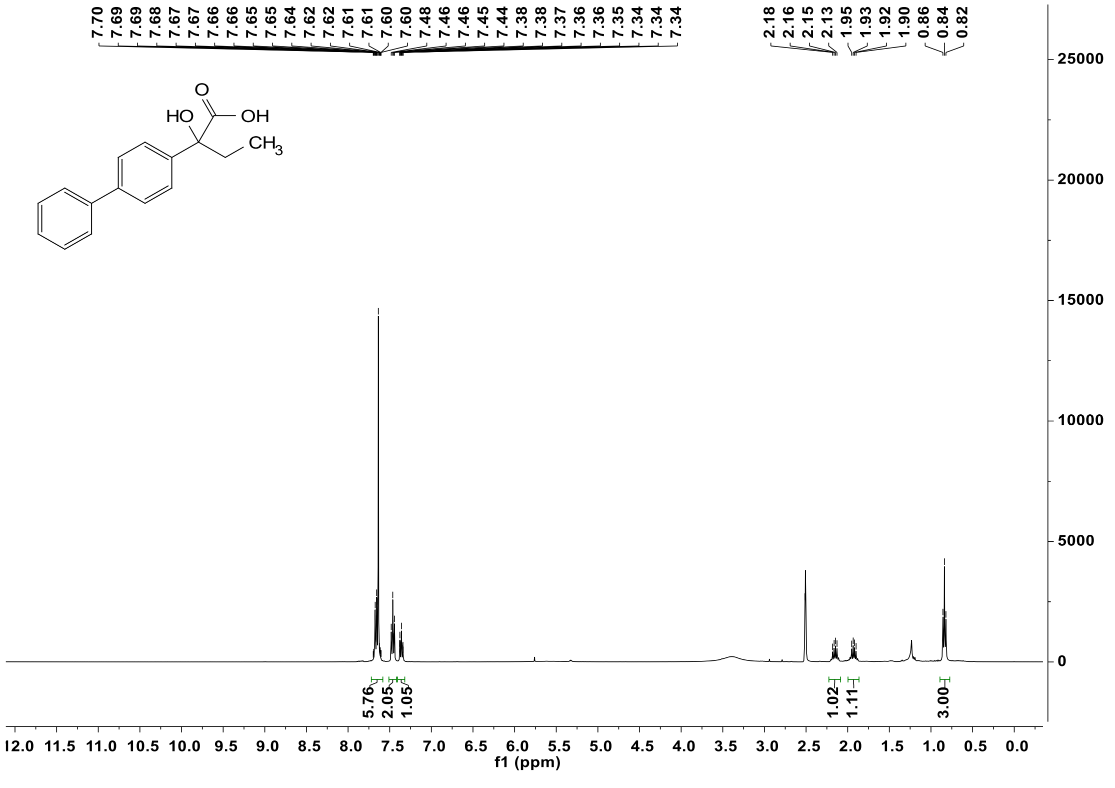

Supplementary Figure 7. ^{1}H NMR spectra of compound **2b**.

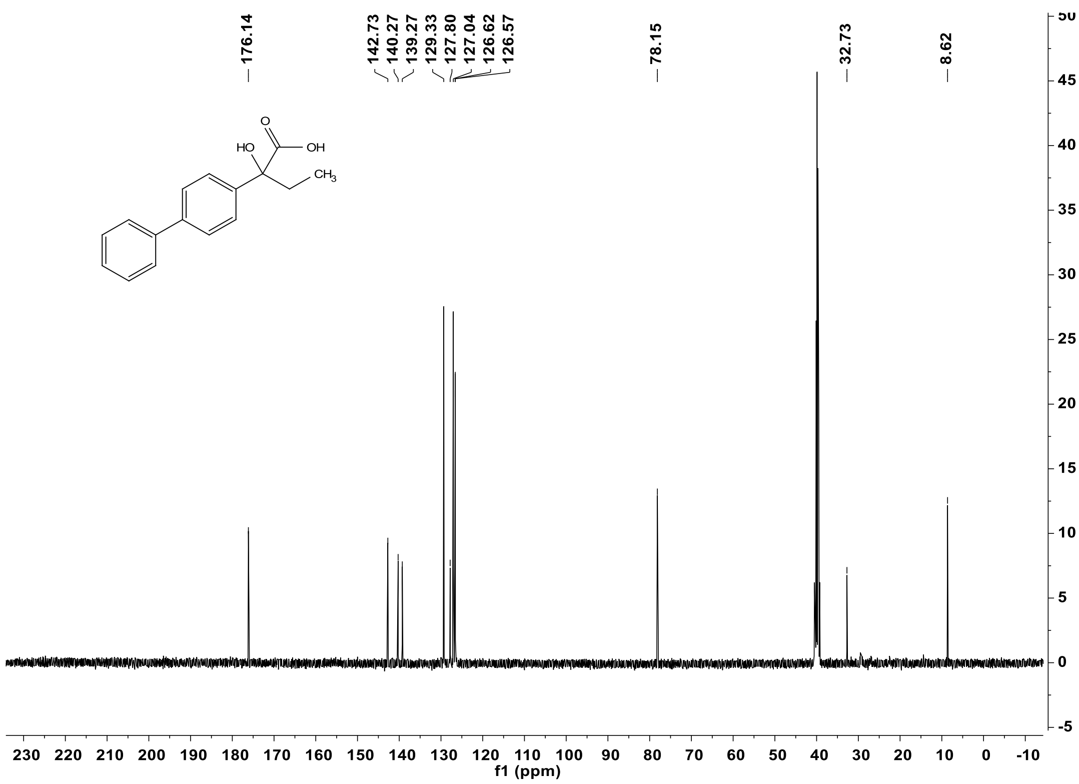

Supplementary Figure 8. ^{13}C NMR spectra of compound **2b**.

2-([1,1'-biphenyl]-4-yl)-2-hydroxyhexanoic acid (2c)

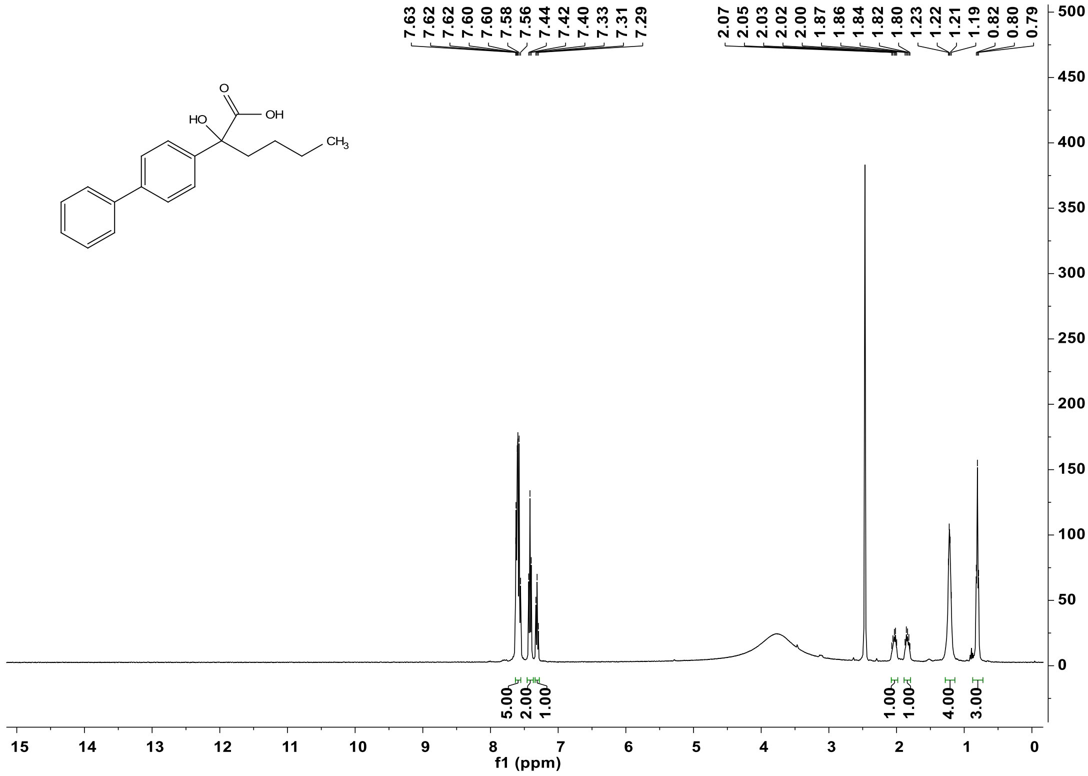

Supplementary Figure 9. ^{1}H NMR spectra of compound **2c**.

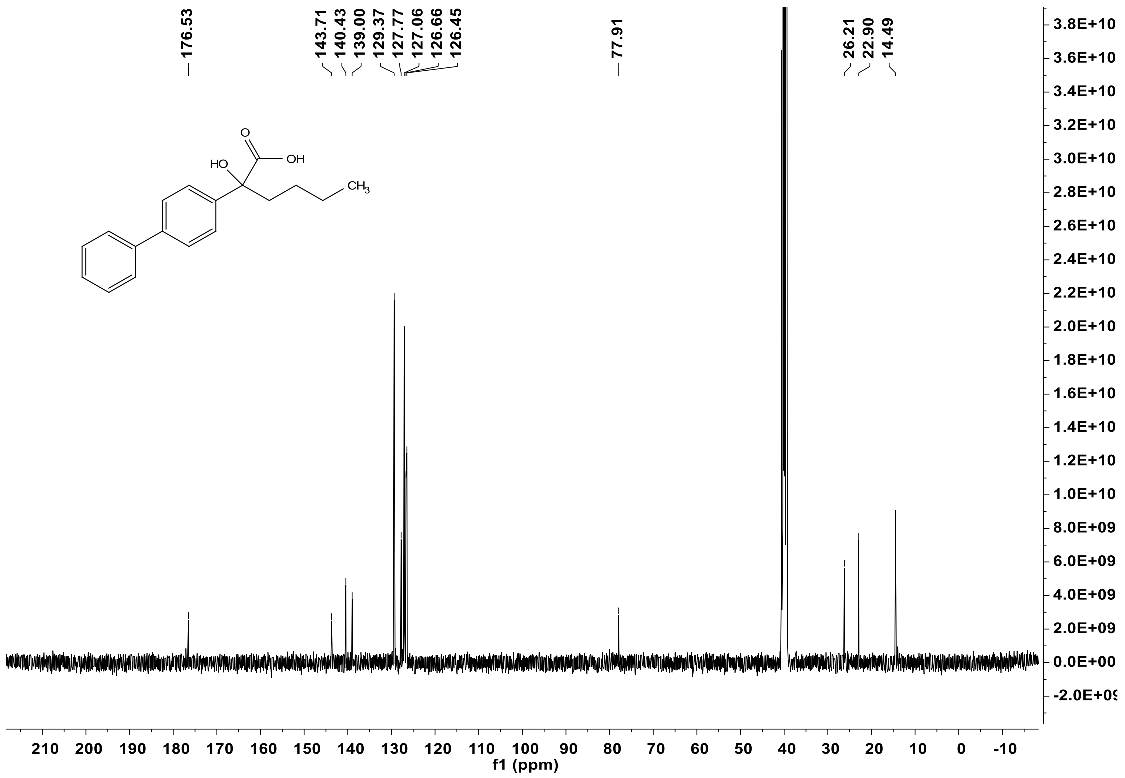

Supplementary Figure 10. ^{13}C NMR spectra of compound **2c**.

2-([1,1'-biphenyl]-4-yl)-2-hydroxy-3-methylbutanoic acid (2d)

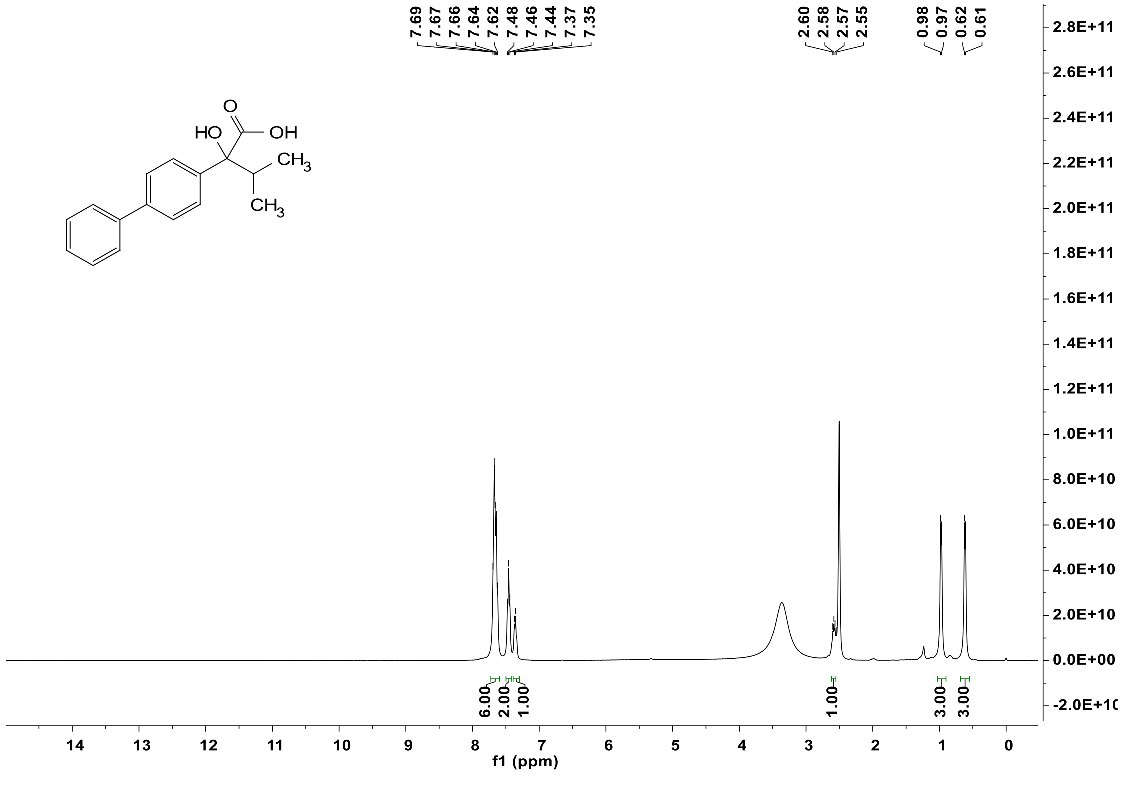

Supplementary Figure 11. ^{1}H NMR spectra of compound **2d**.

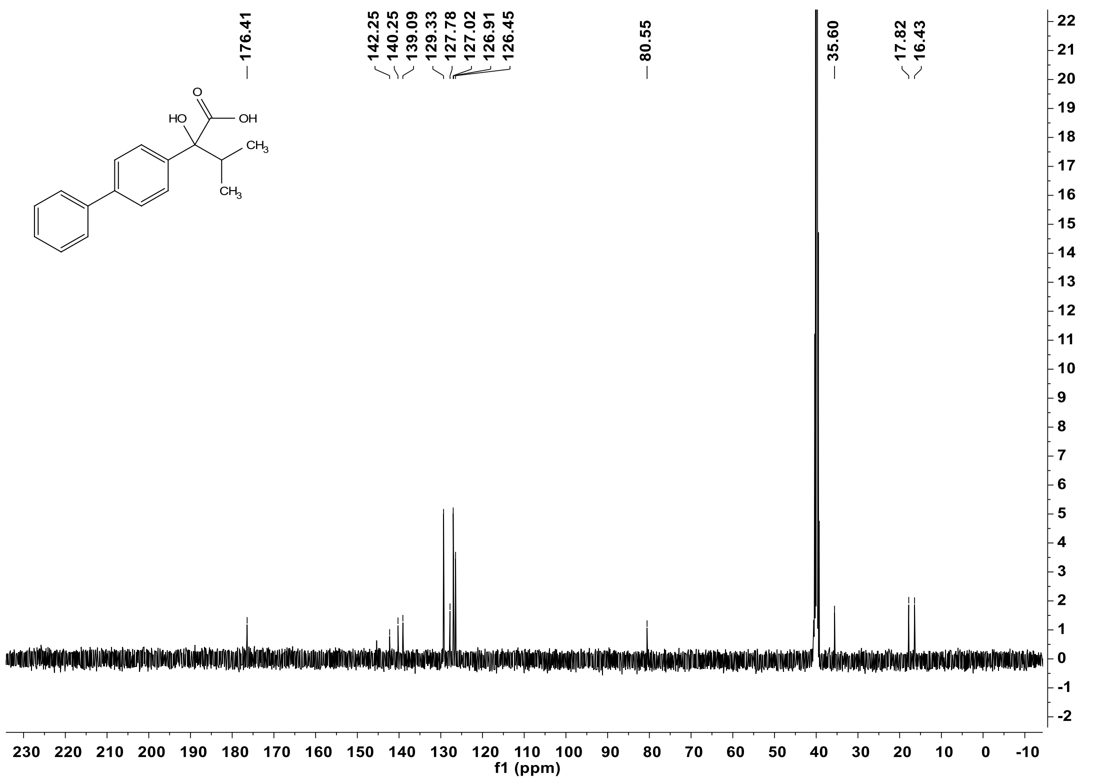

Supplementary Figure 12. ^{13}C NMR spectra of compound **2d**.

2-([1,1'-biphenyl]-4-yl)-2-hydroxy-3,3-dimethylbutanoic acid (2e)

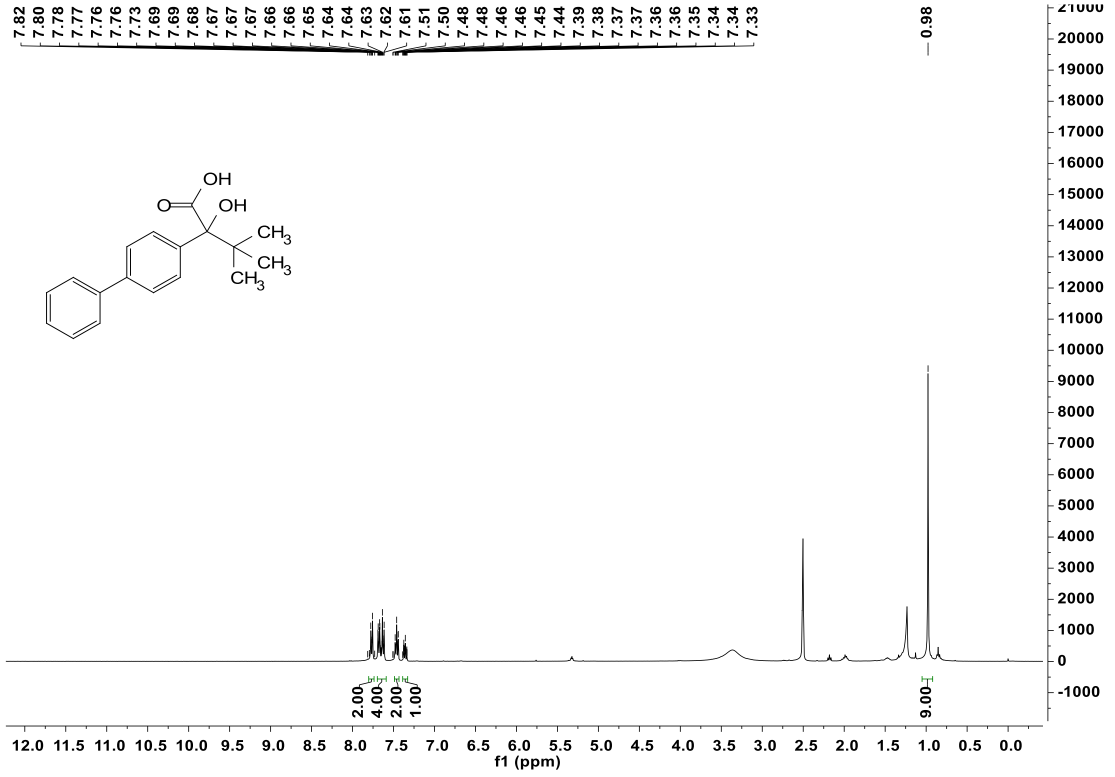

Supplementary Figure 13. ^{1}H NMR spectra of compound **2e**.

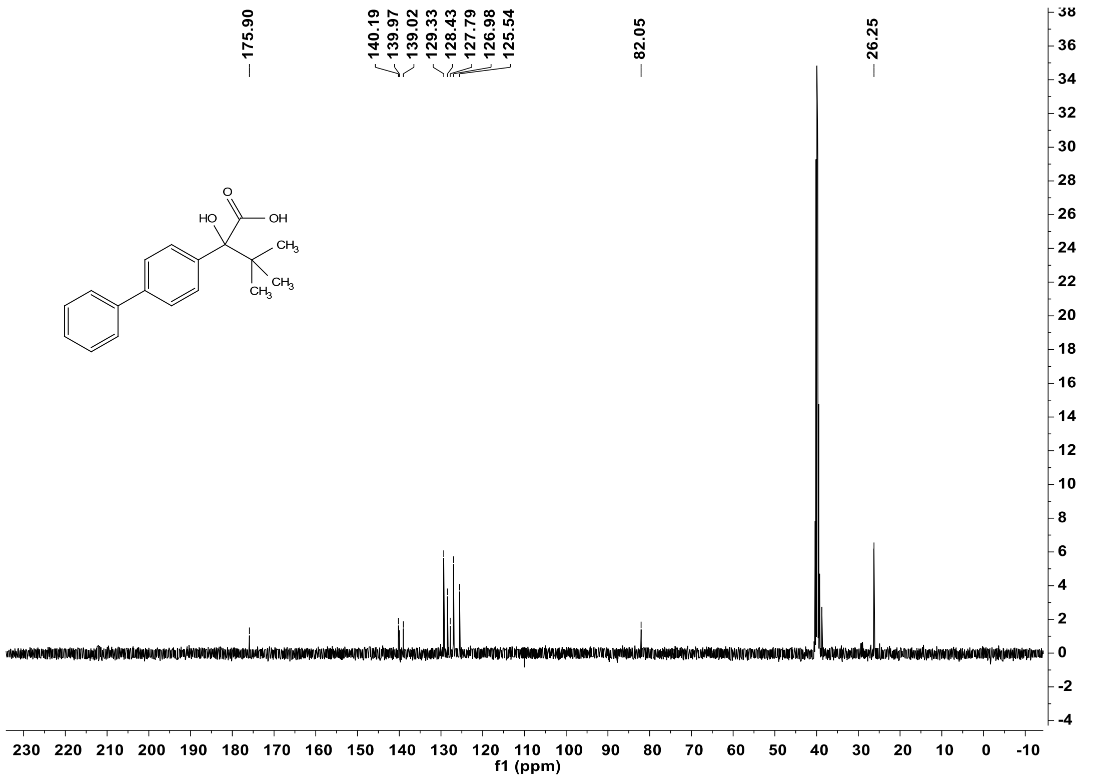

Supplementary Figure 14. ^{13}C NMR spectra of compound **2e**.

2-hydroxy-2-(naphthalen-2-yl)propanoic acid (2f)

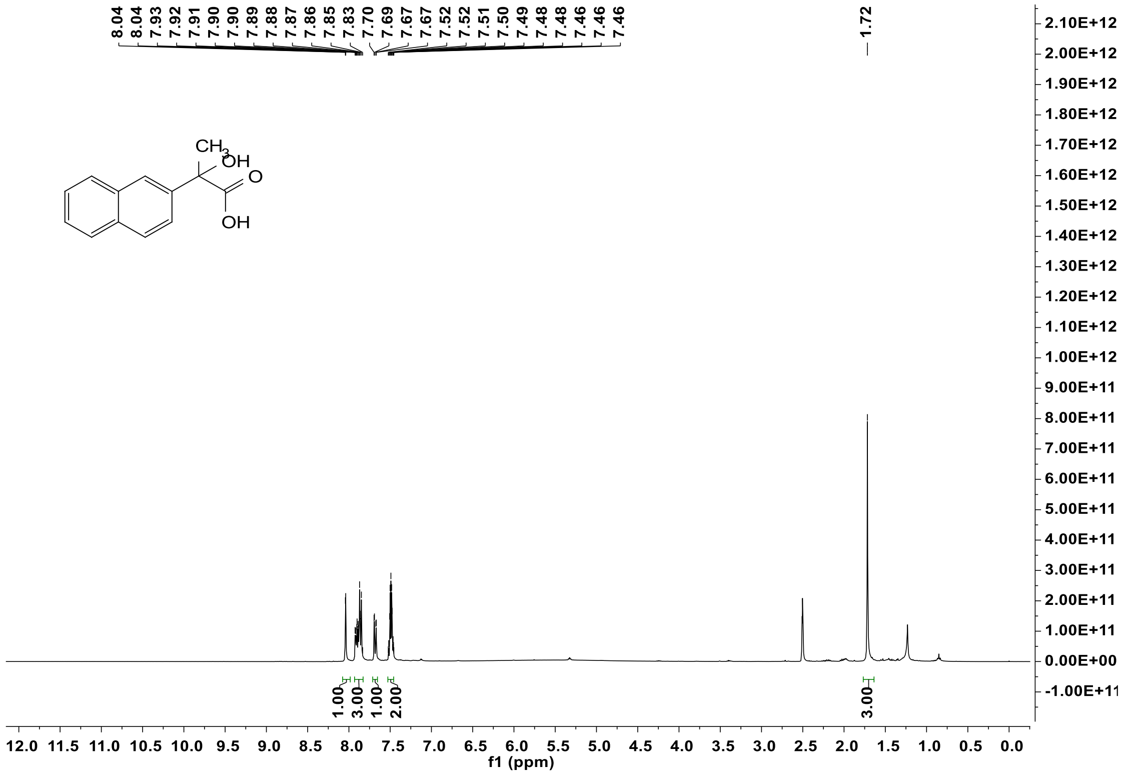

Supplementary Figure 15. ^{1}H NMR spectra of compound **2f**.

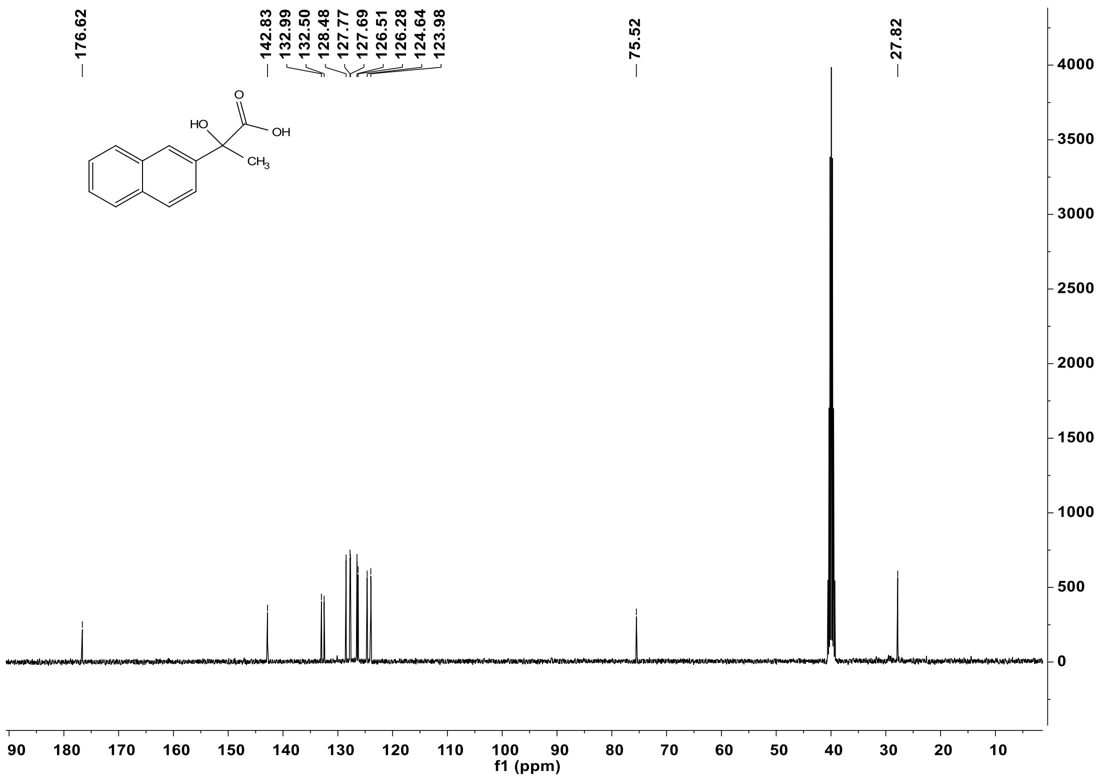

Supplementary Figure 16. ^{13}C NMR spectra of compound **2f**.

2-hydroxy-2-(4-(thiophen-2-yl)phenyl)propanoic acid (2g)

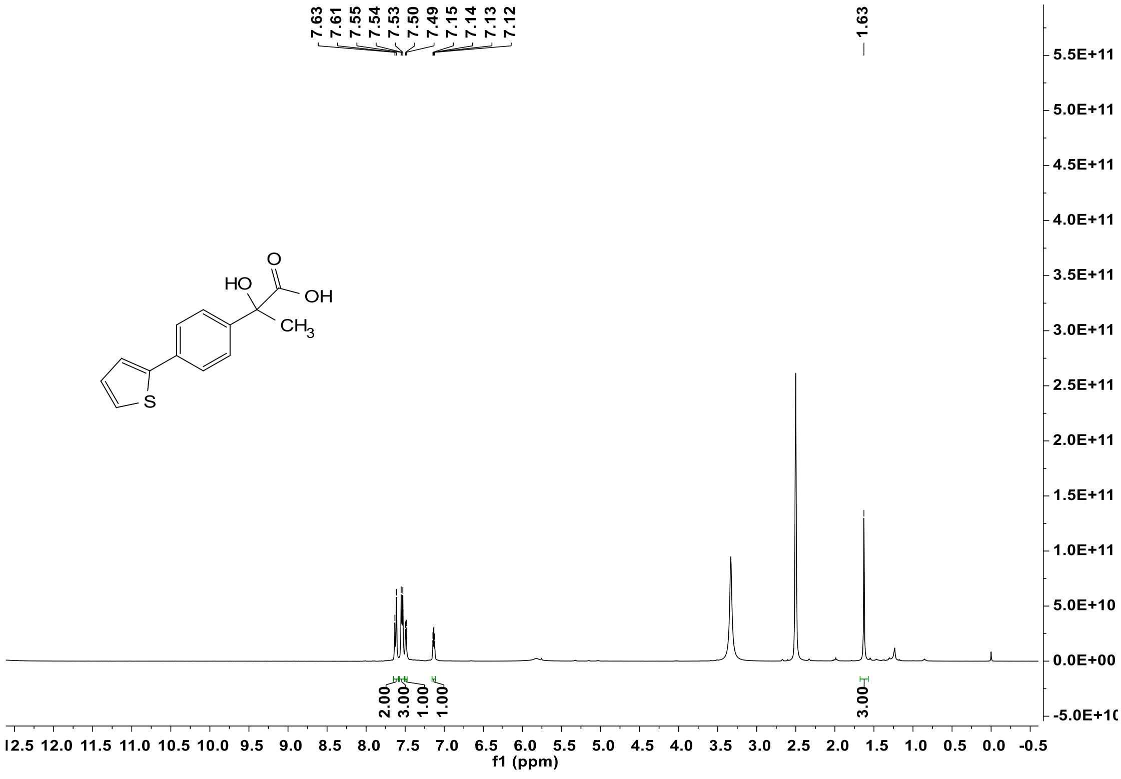

Supplementary Figure 17. ^{1}H NMR spectra of compound **2g**.

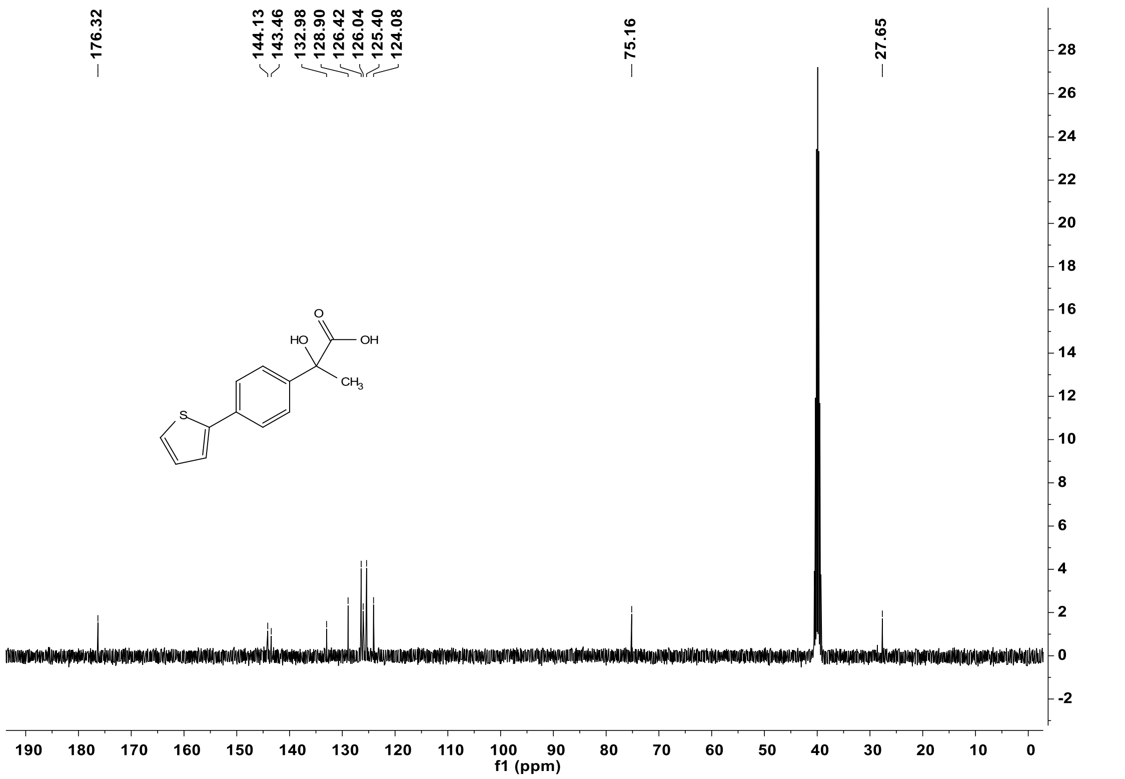

Supplementary Figure 18. ^{13}C NMR spectra of compound **2g**.

2-(4-(*tert*-butoxycarbonyl)phenyl)-2-hydroxypropanoic acid (2h)

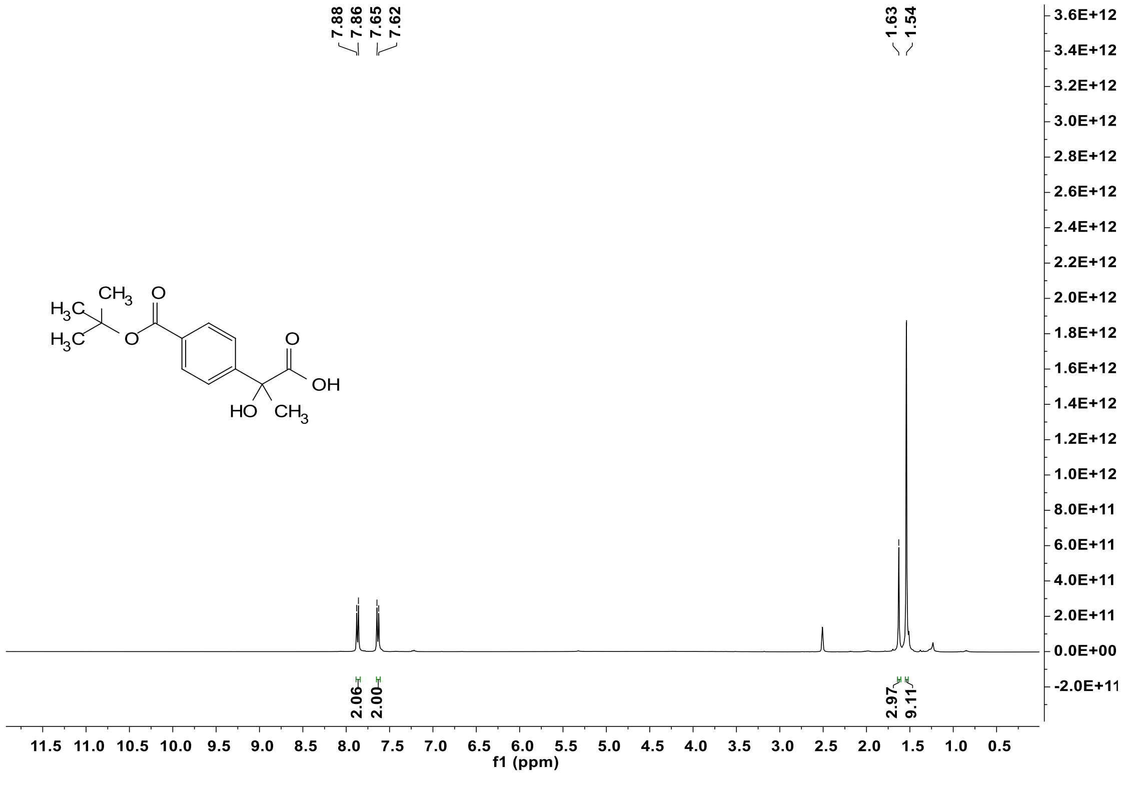

Supplementary Figure 19. ^{1}H NMR spectra of compound **2h**.

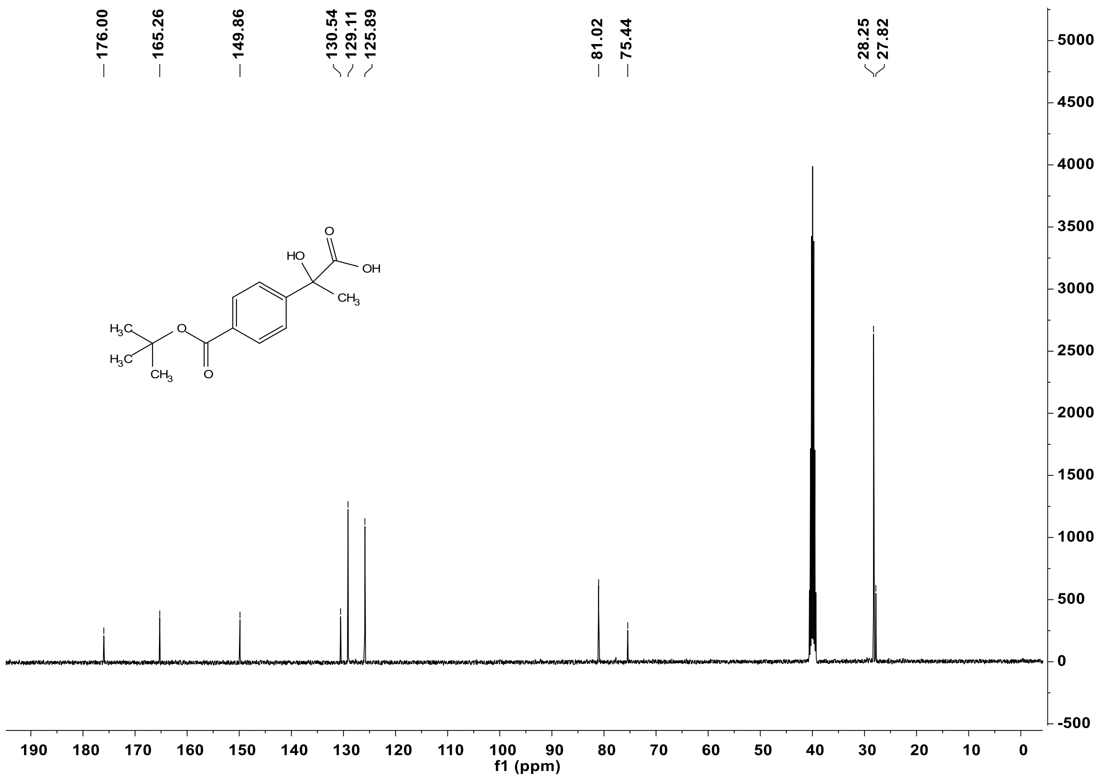

Supplementary Figure 20. ^{13}C NMR spectra of compound **2h**.

2-cyclohexyl-2-hydroxy-2-phenylacetic acid (2i)

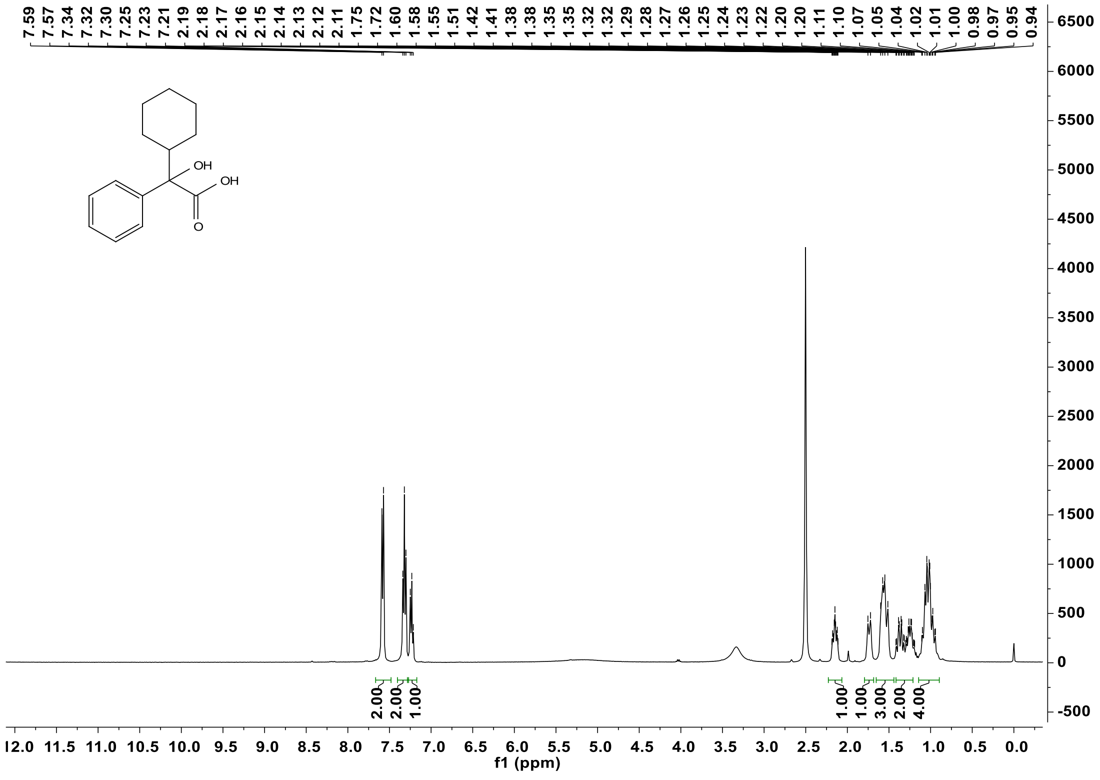

Supplementary Figure 21. ^{1}H NMR spectra of compound **2i**.

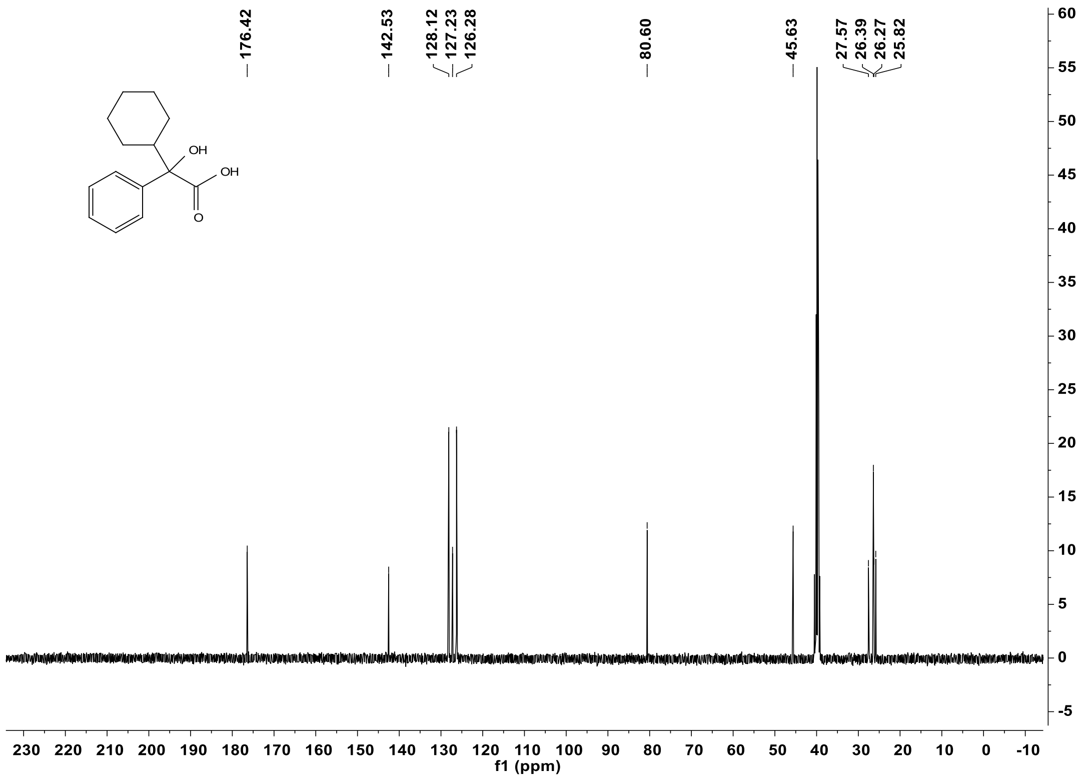

Supplementary Figure 22. ^{13}C NMR spectra of compound **2i**.

2-hydroxy-2,3-diphenylpropanoic acid (2j)

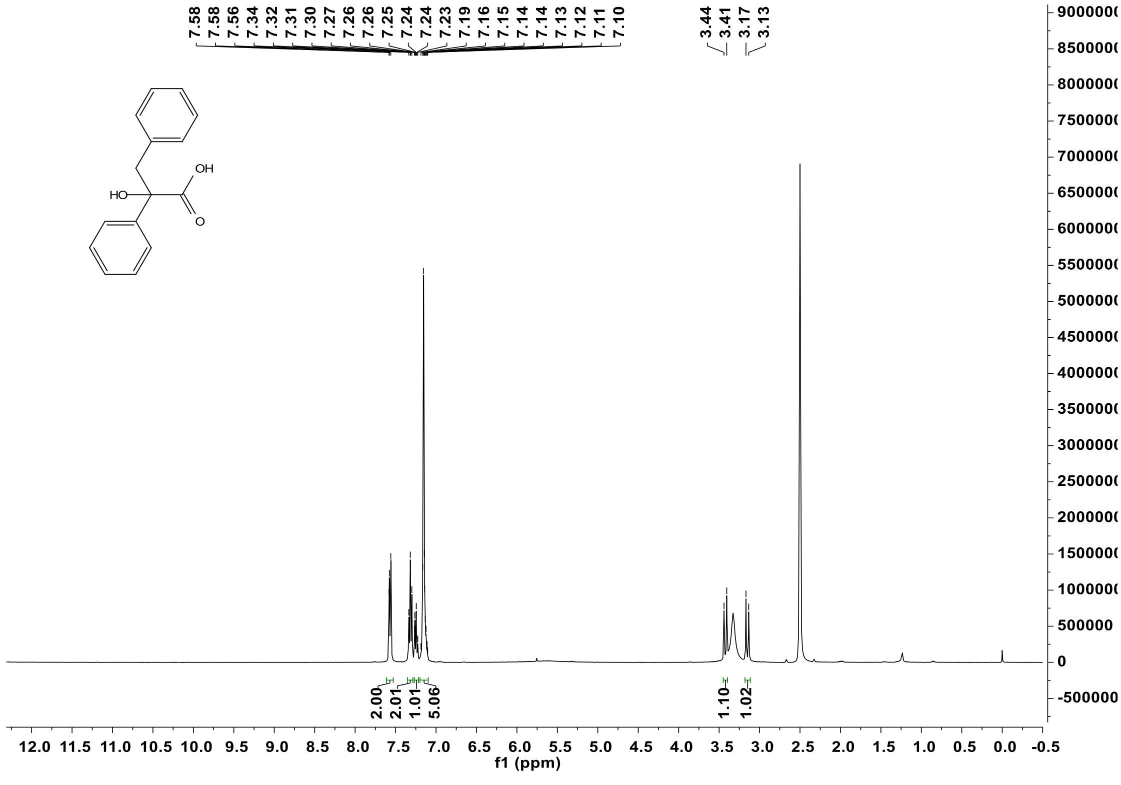

Supplementary Figure 23. ^{1}H NMR spectra of compound **2j**.

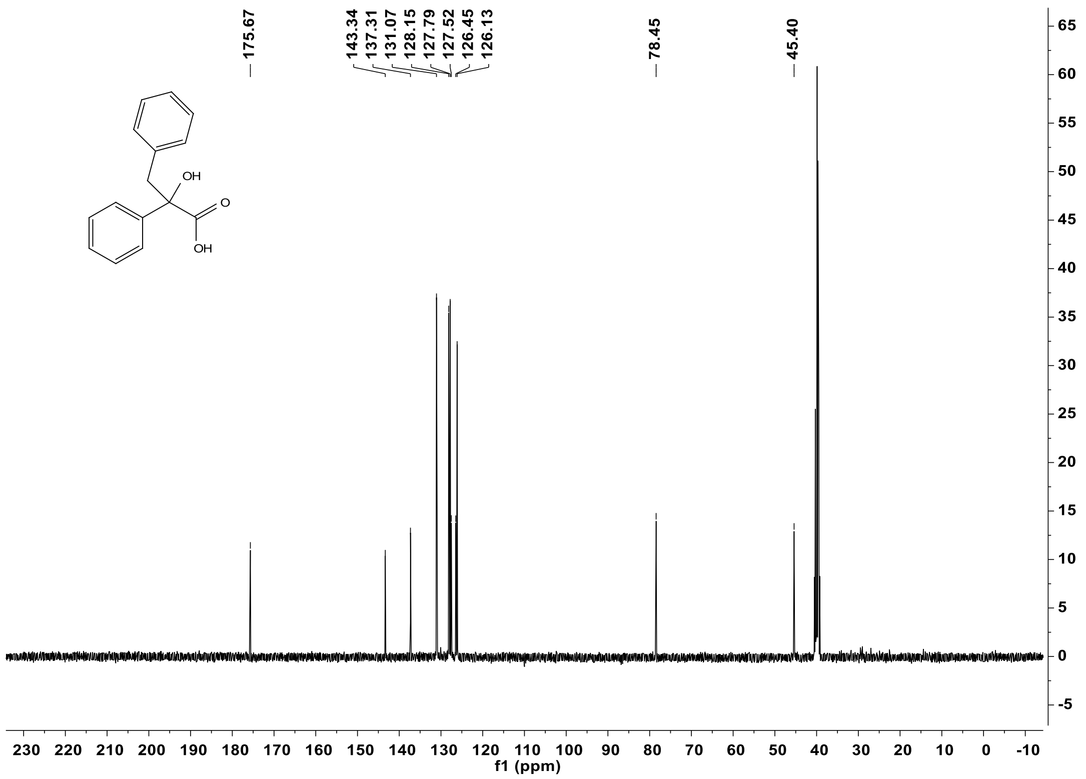

Supplementary Figure 24. ^{13}C NMR spectra of compound **2j**.

2-hydroxy-2-phenylpropanoic acid (2k)

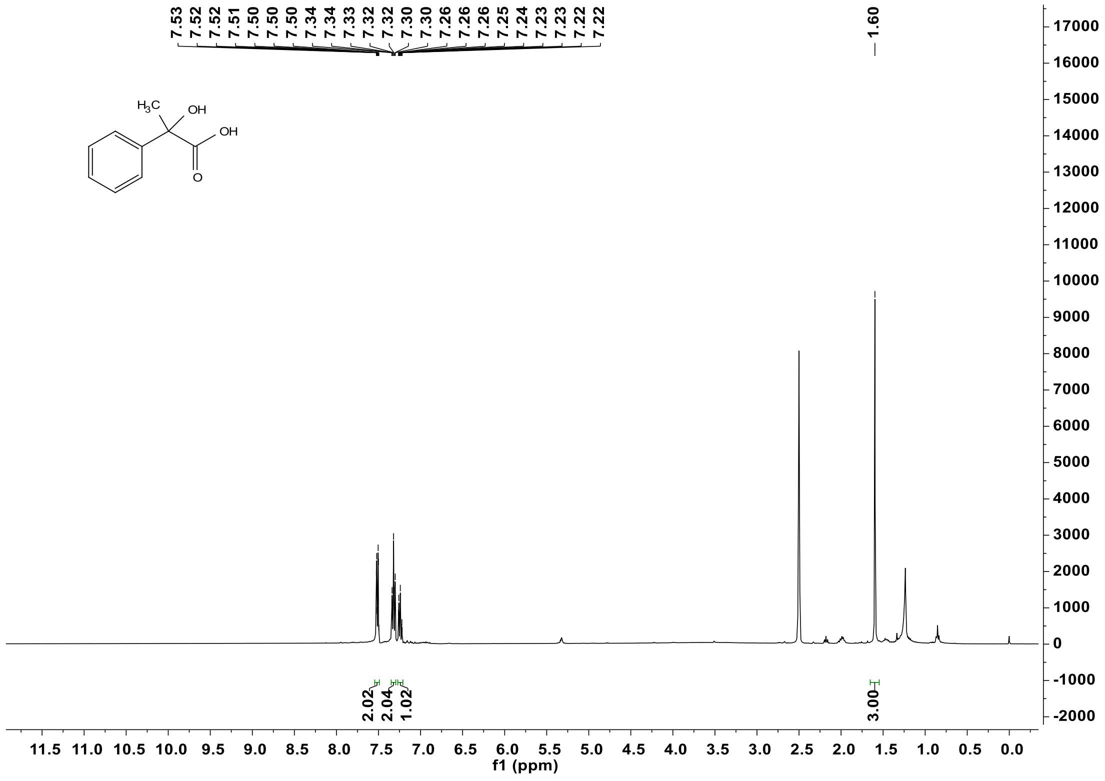

Supplementary Figure 25. ^{1}H NMR spectra of compound **2k**.

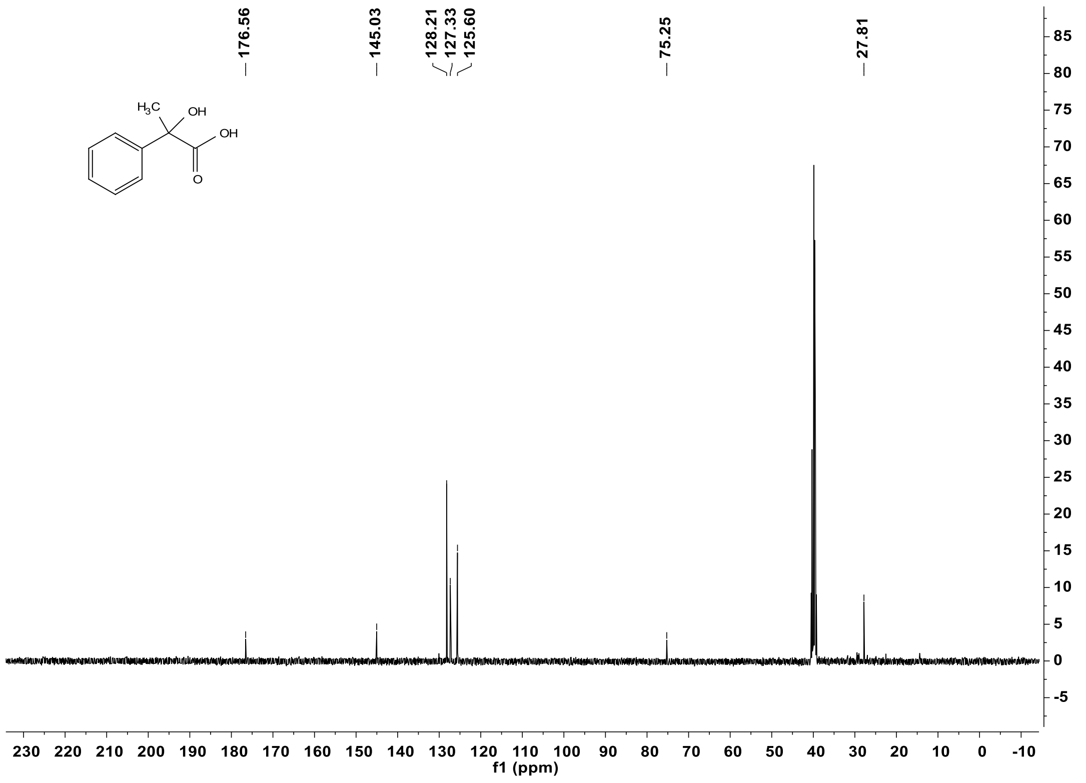

Supplementary Figure 26. ^{13}C NMR spectra of compound **2k**.

2-hydroxy-2-(2-methoxyphenyl)propanoic acid (2l)

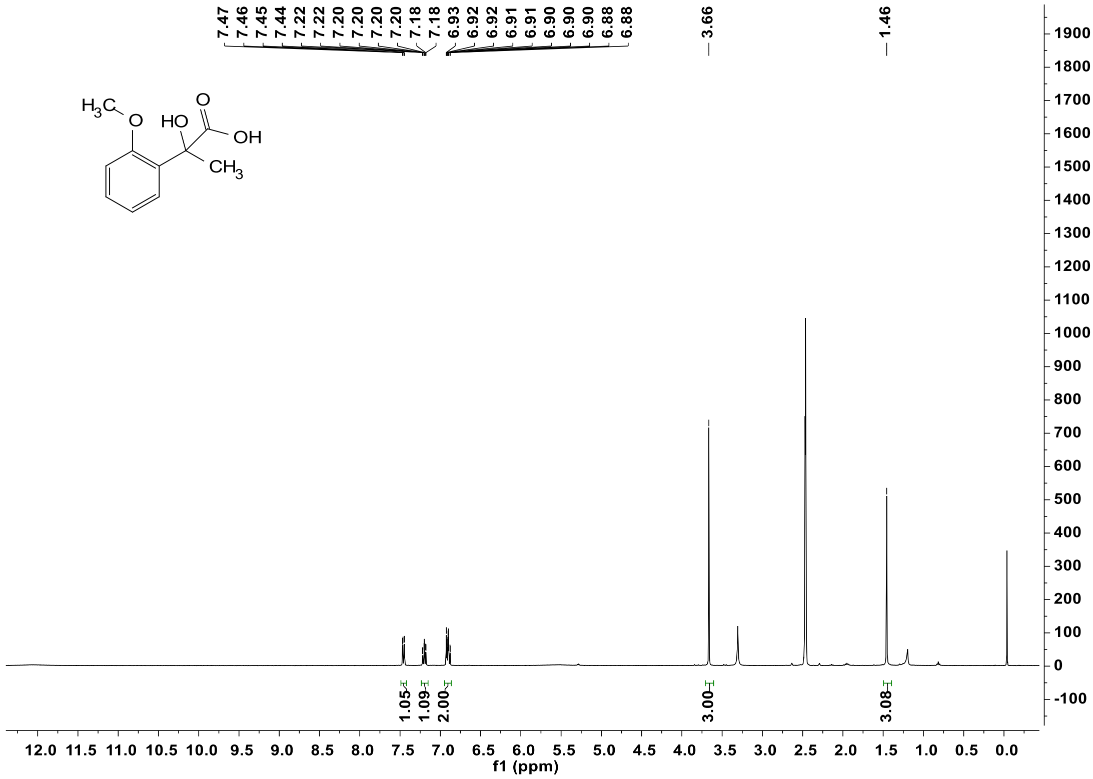

Supplementary Figure 27. ^{13}C NMR spectra of compound **2l**.

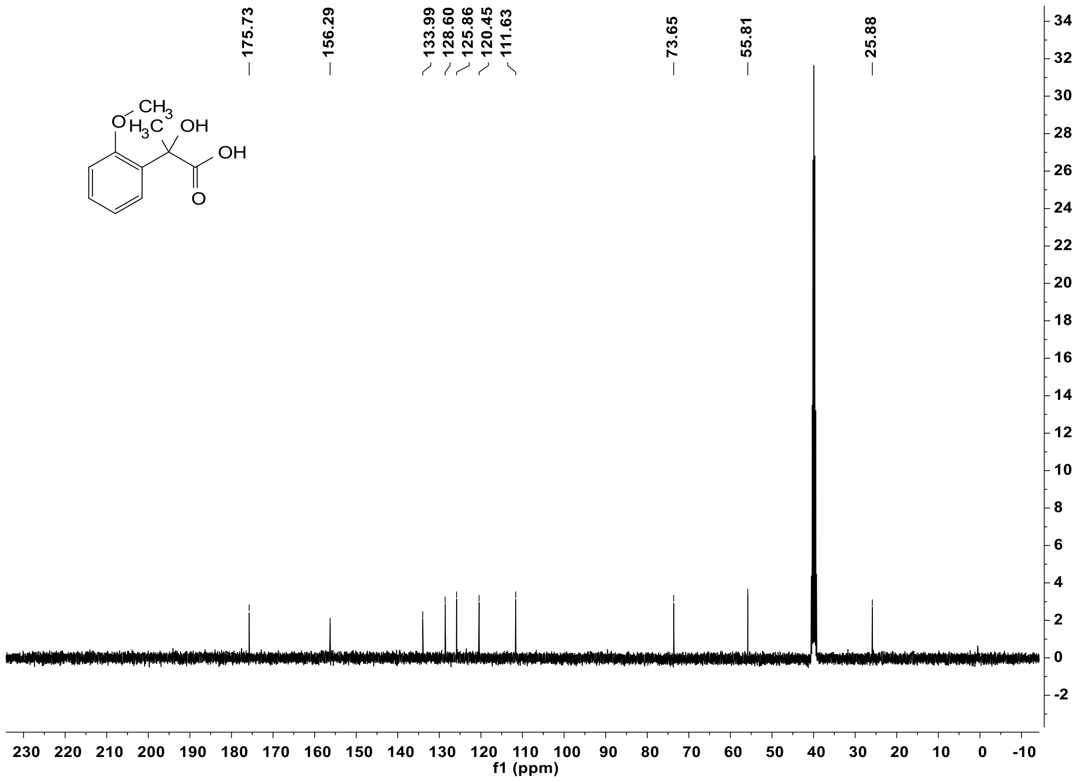

Supplementary Figure 28. ^{13}C NMR spectra of compound **2l**.

2-hydroxy-2,2-diphenylacetic acid (4a)

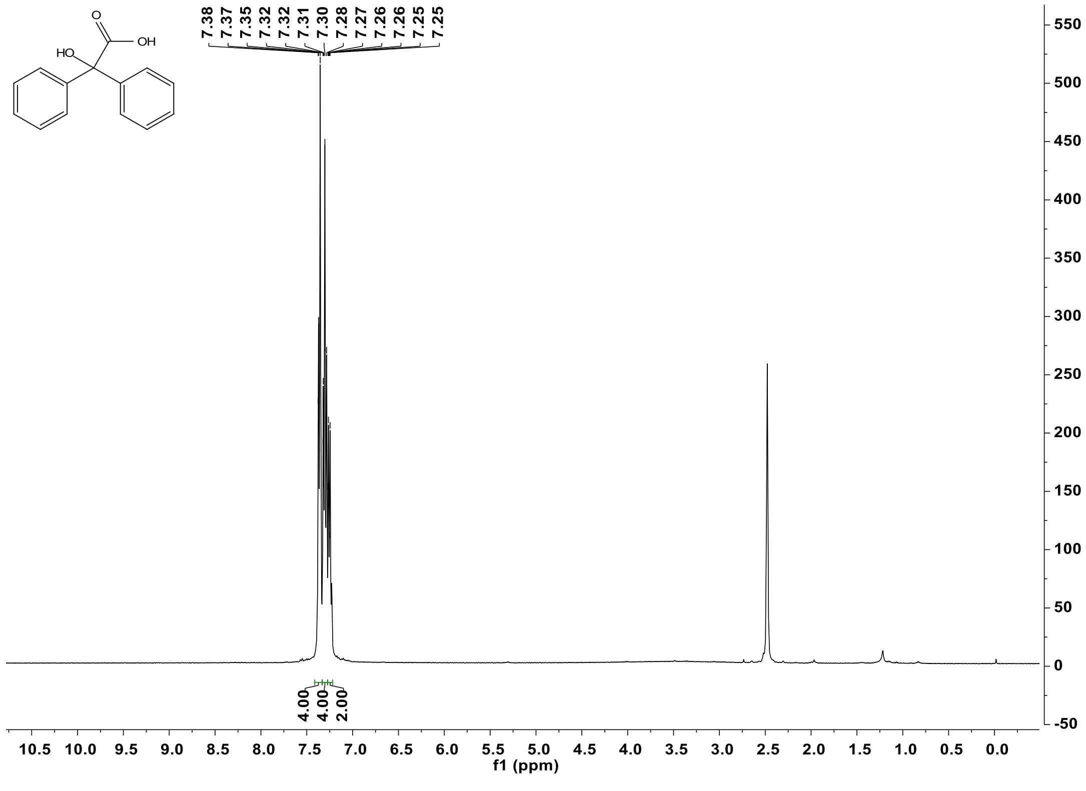

Supplementary Figure 29. ^{1}H NMR spectra of compound **4a**.

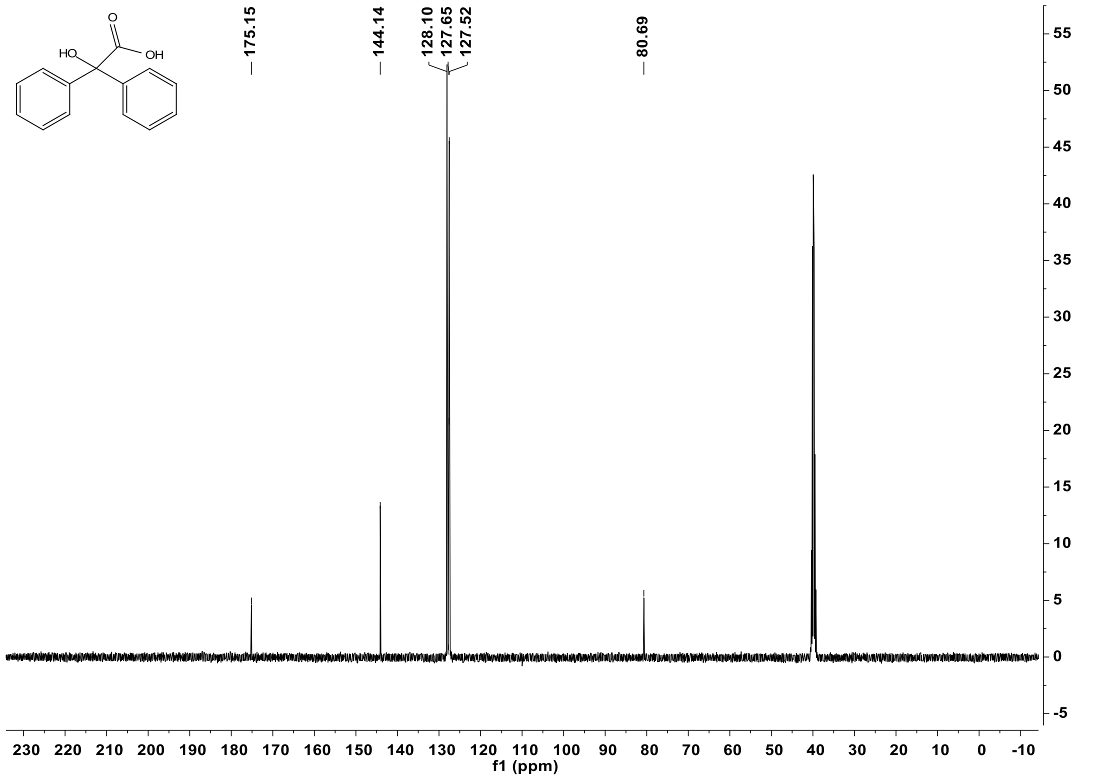

Supplementary Figure 30. ^{13}C NMR spectra of compound **4a**.

2-([1,1'-biphenyl]-4-yl)-2-hydroxy-2-phenylacetic acid (4b)

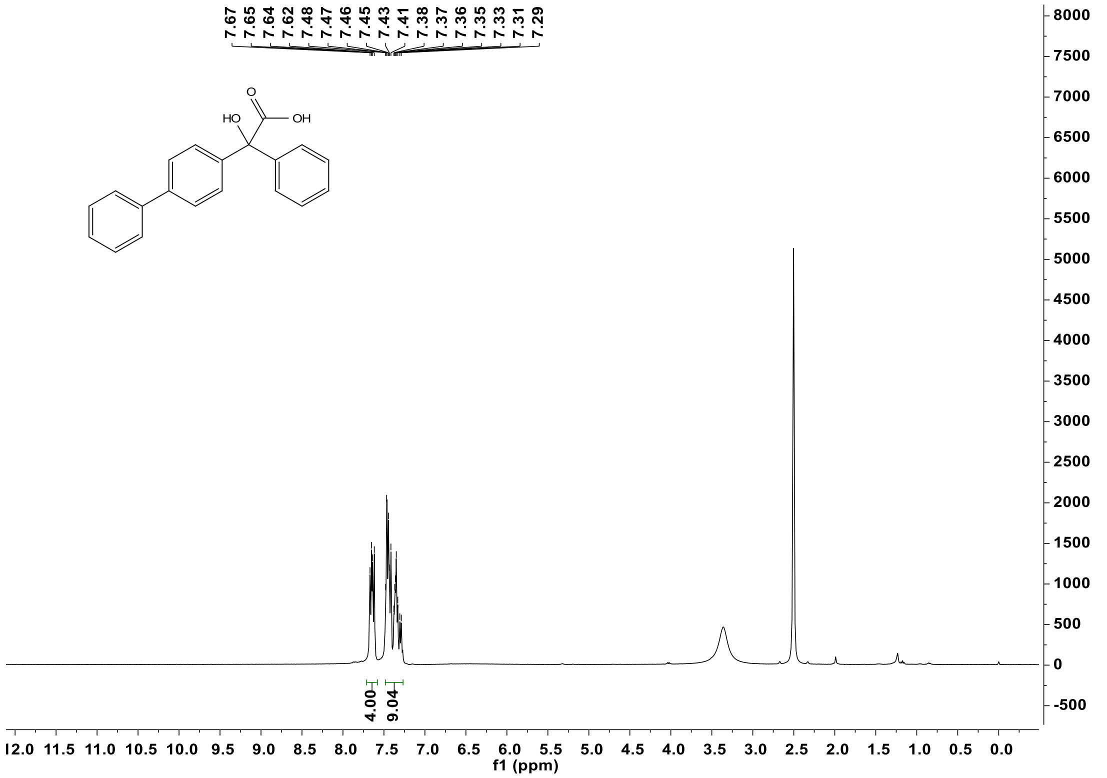

Supplementary Figure 31. ^{1}H NMR spectra of compound **4b**.

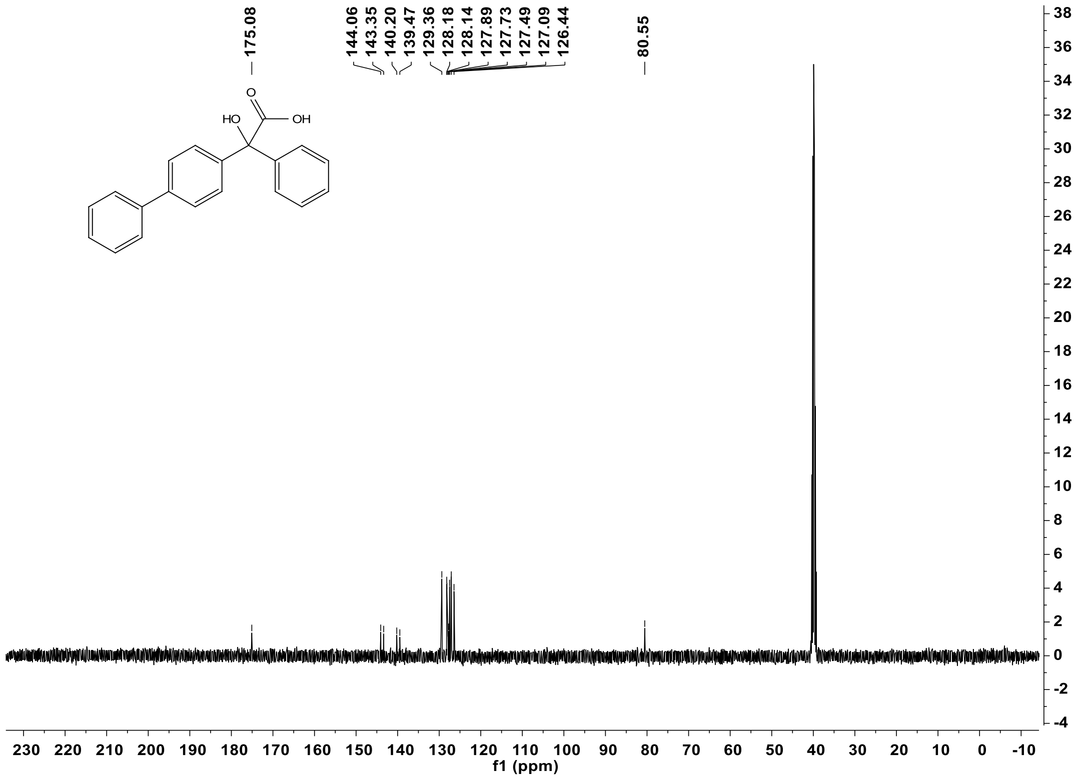

Supplementary Figure 32. ^{13}C NMR spectra of compound **4b**.

2-hydroxy-2-phenyl-2-(*p*-tolyl)acetic acid (4c)

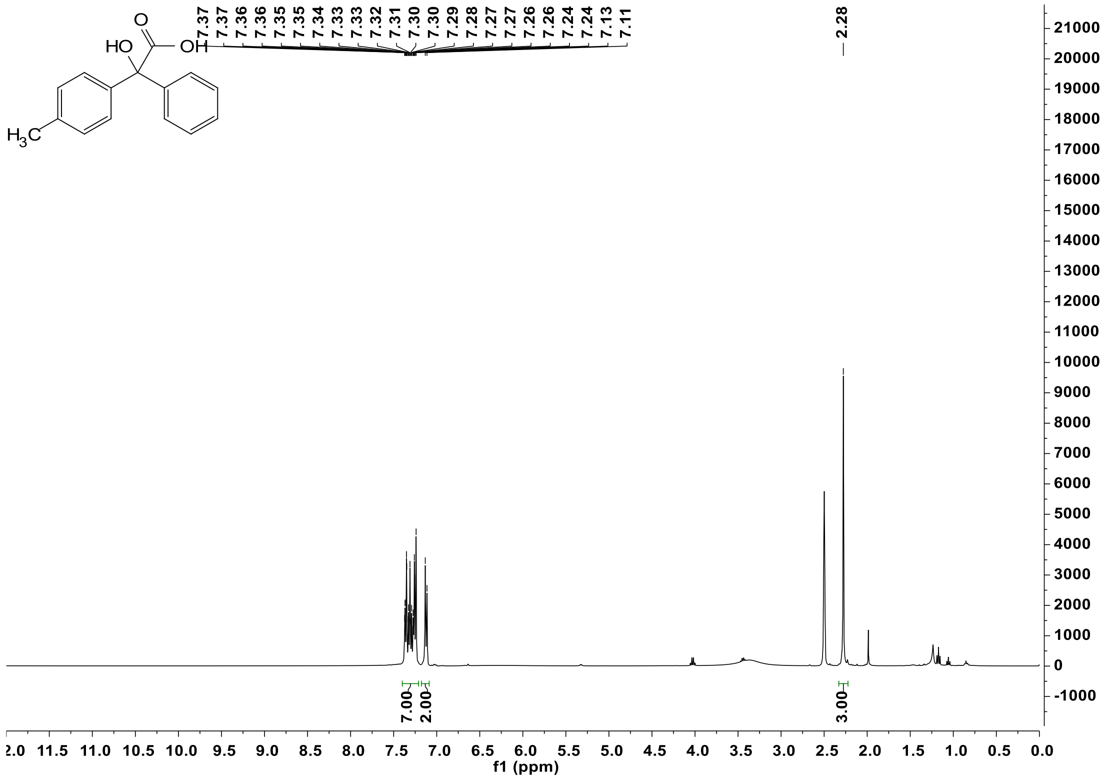

Supplementary Figure 33. ^{1}H NMR spectra of compound **4c**.

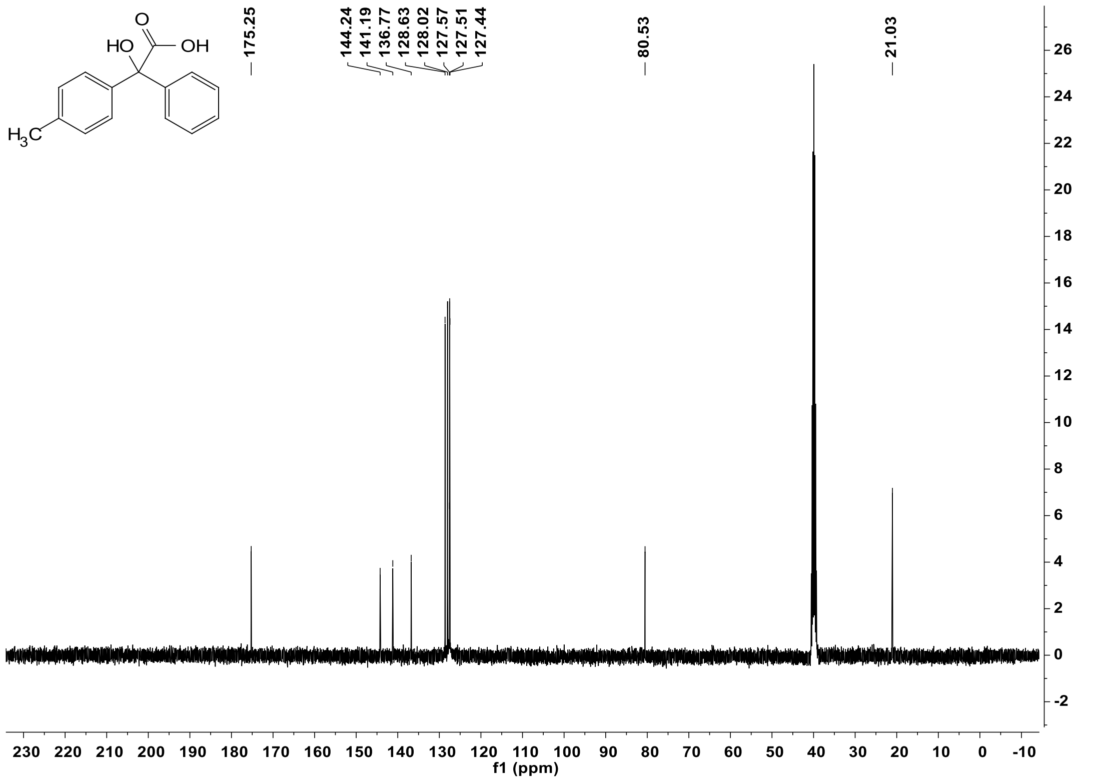

Supplementary Figure 34. ^{13}C NMR spectra of compound **4c**.

2-hydroxy-2-(4-methoxyphenyl)-2-phenylacetic acid (4d)

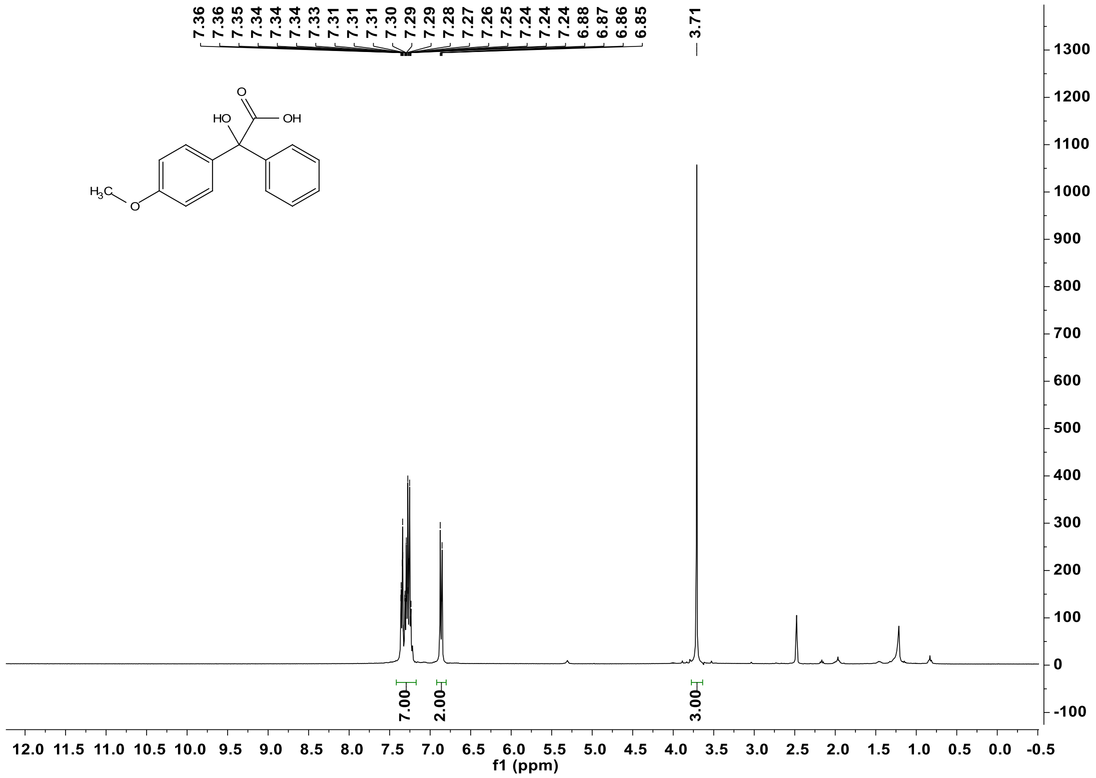

Supplementary Figure 35. ^{1}H NMR spectra of compound **4d**.

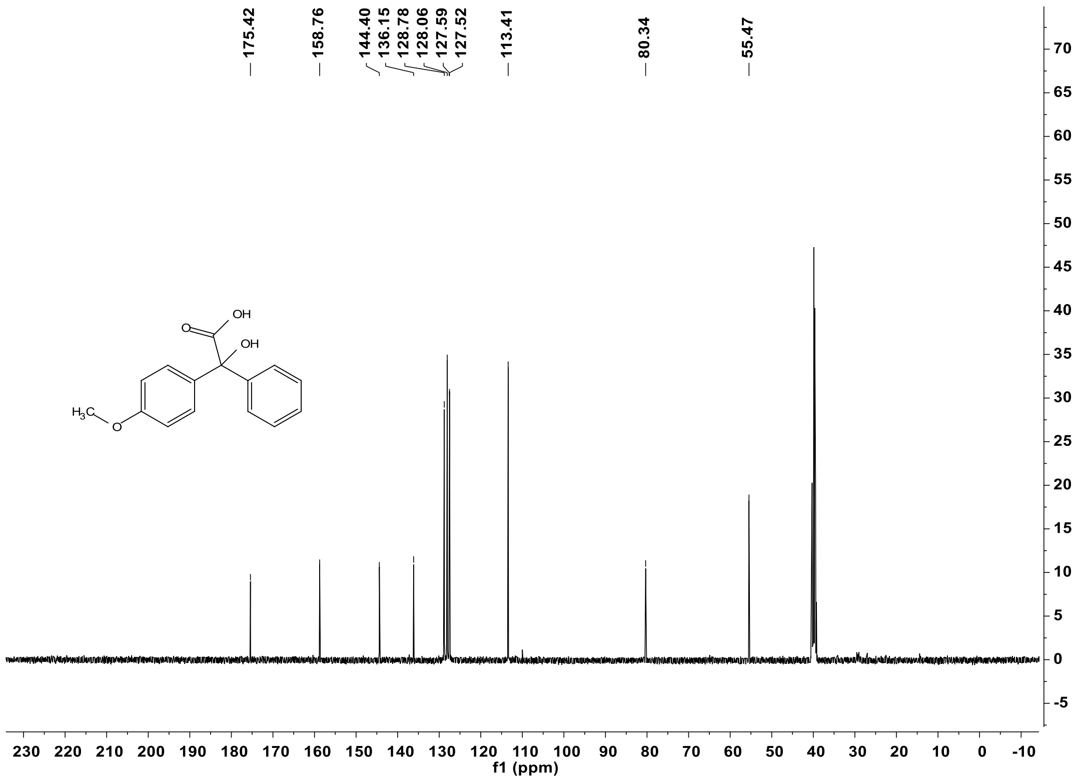

Supplementary Figure 36. ^{13}C NMR spectra of compound **4d**.

2-hydroxy-2-phenyl-2-(4-(trifluoromethyl)phenyl)acetic acid (4e)

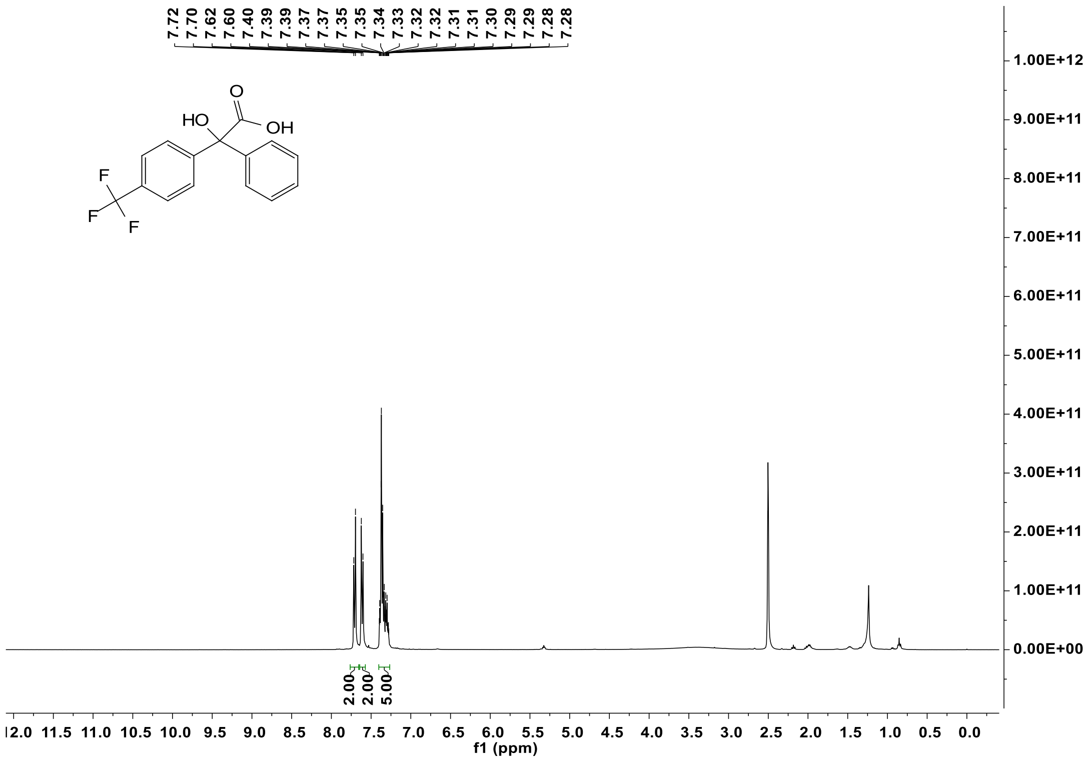

Supplementary Figure 37. ^{1}H NMR spectra of compound **4e**.

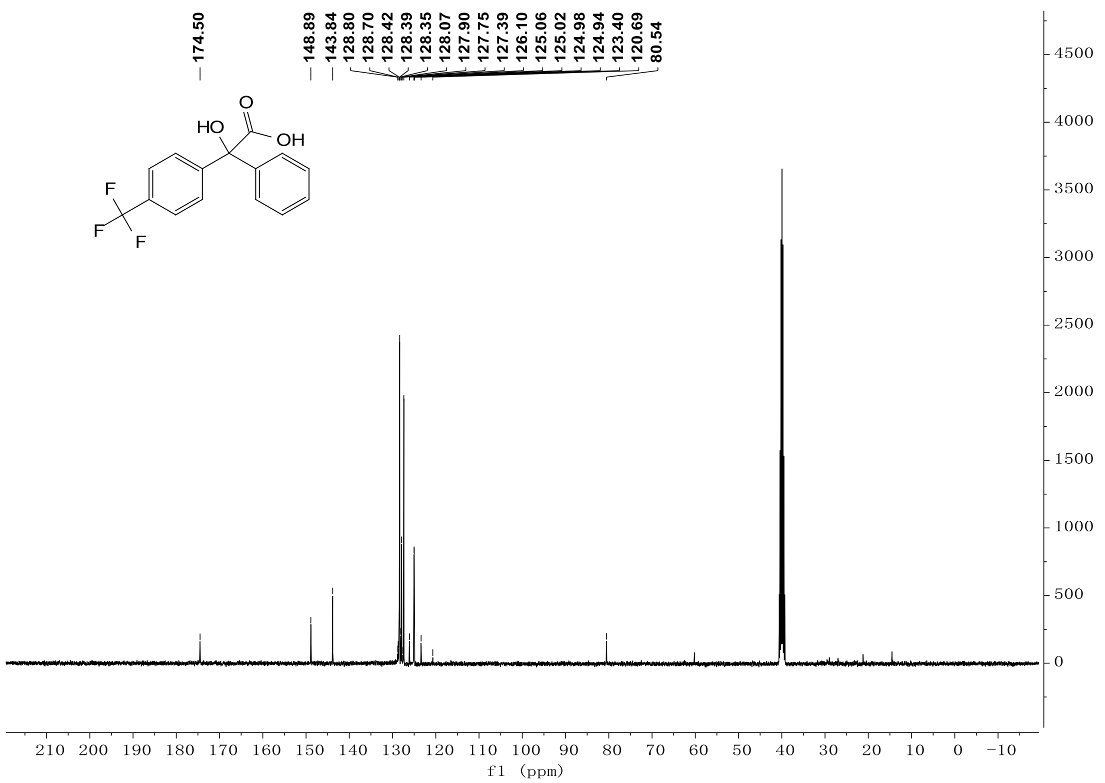

Supplementary Figure 38. ^{13}C NMR spectra of compound **4e**.

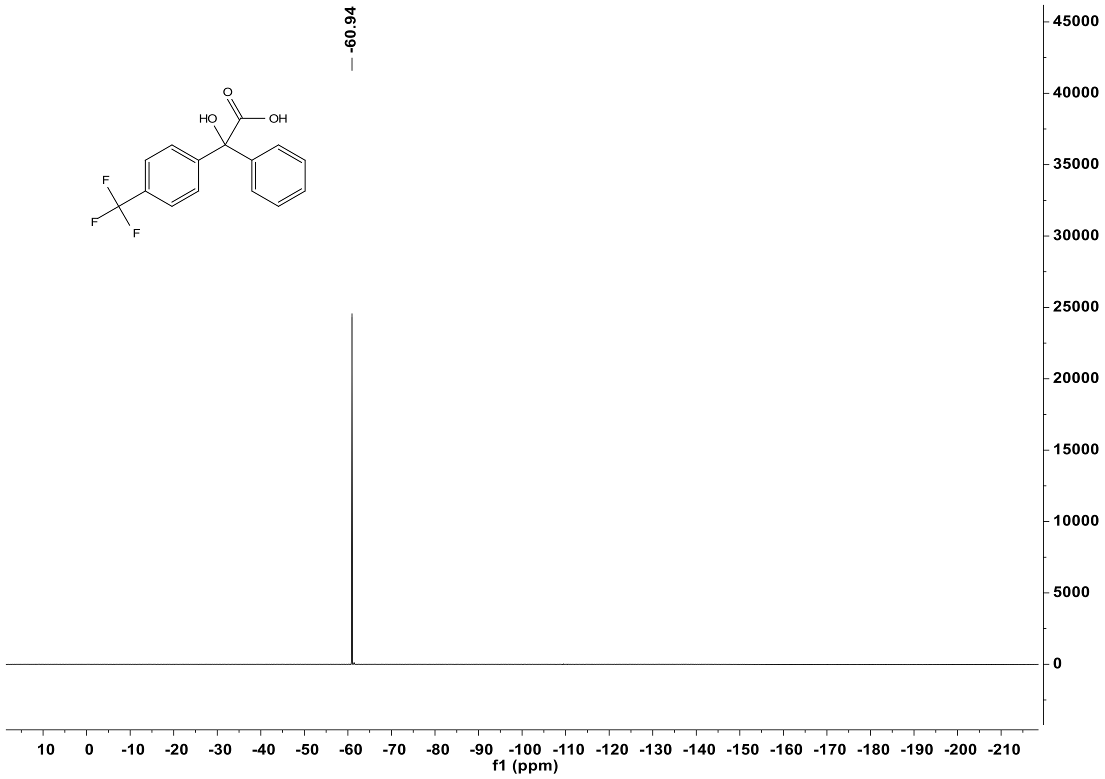

Supplementary Figure 39. ^{19}F NMR spectra of compound **4e**.

2-(4-fluorophenyl)-2-hydroxy-2-phenylacetic (4f)

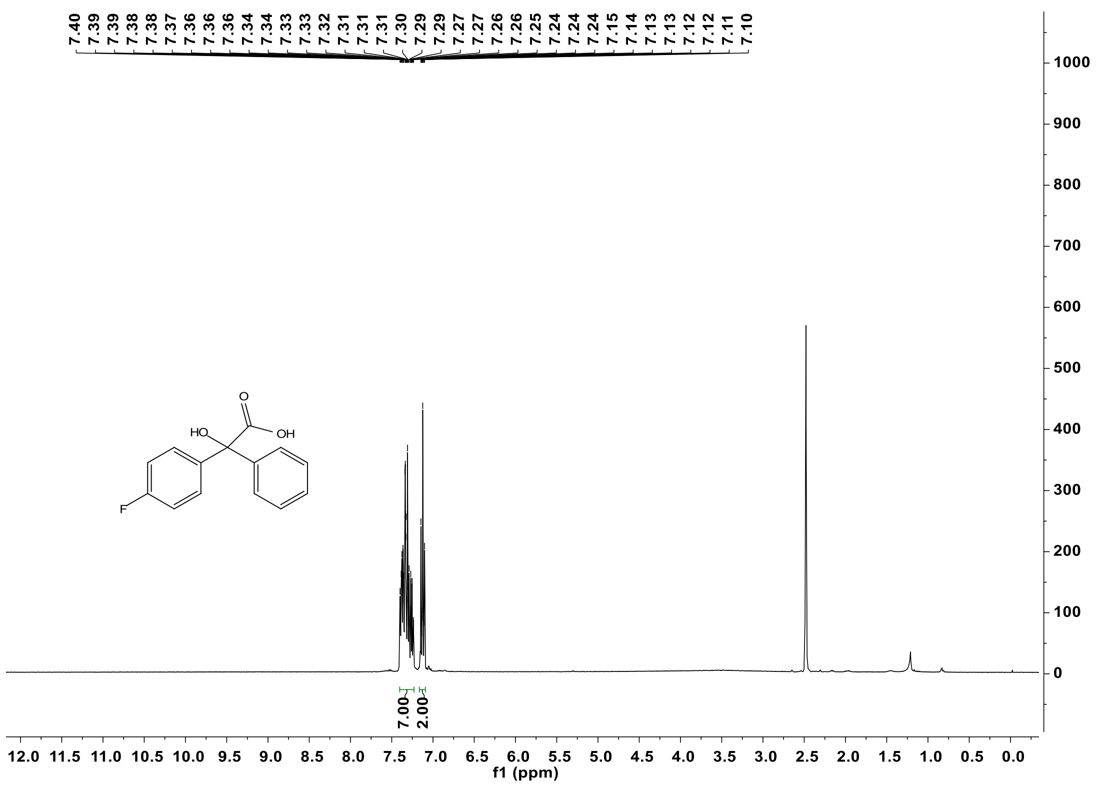

Supplementary Figure 40. ^{1}H NMR spectra of compound **4f**.

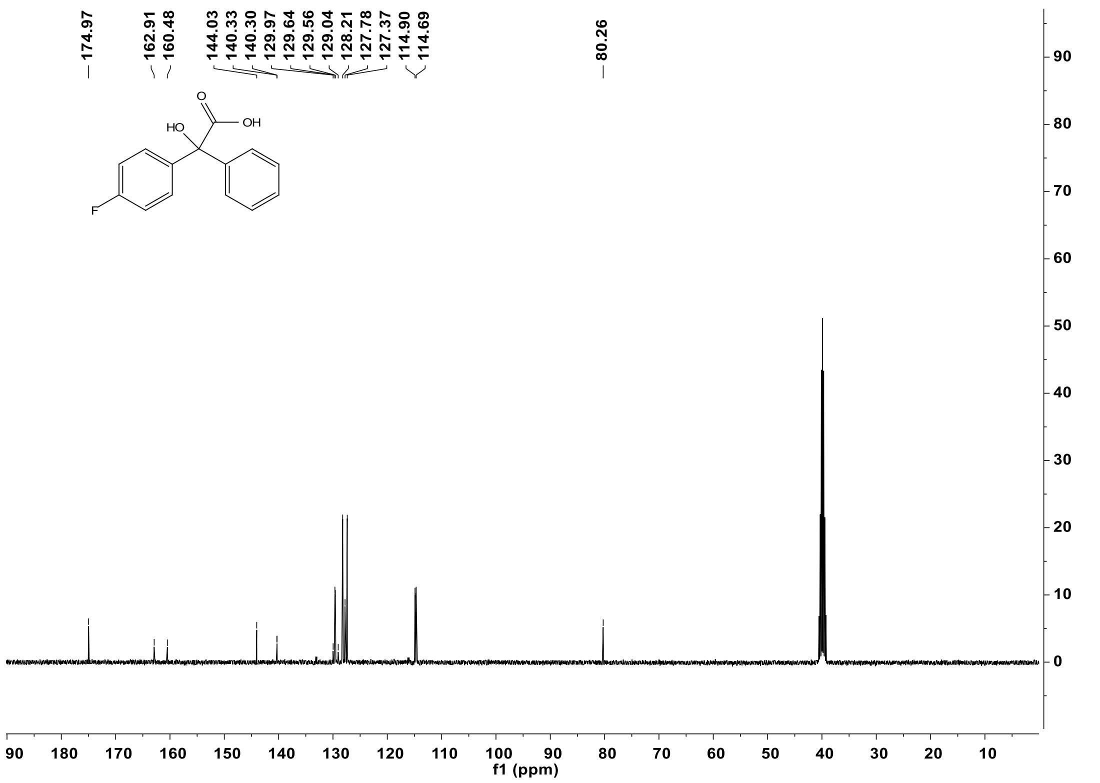

Supplementary Figure 41. ^{13}C NMR spectra of compound **4f**.

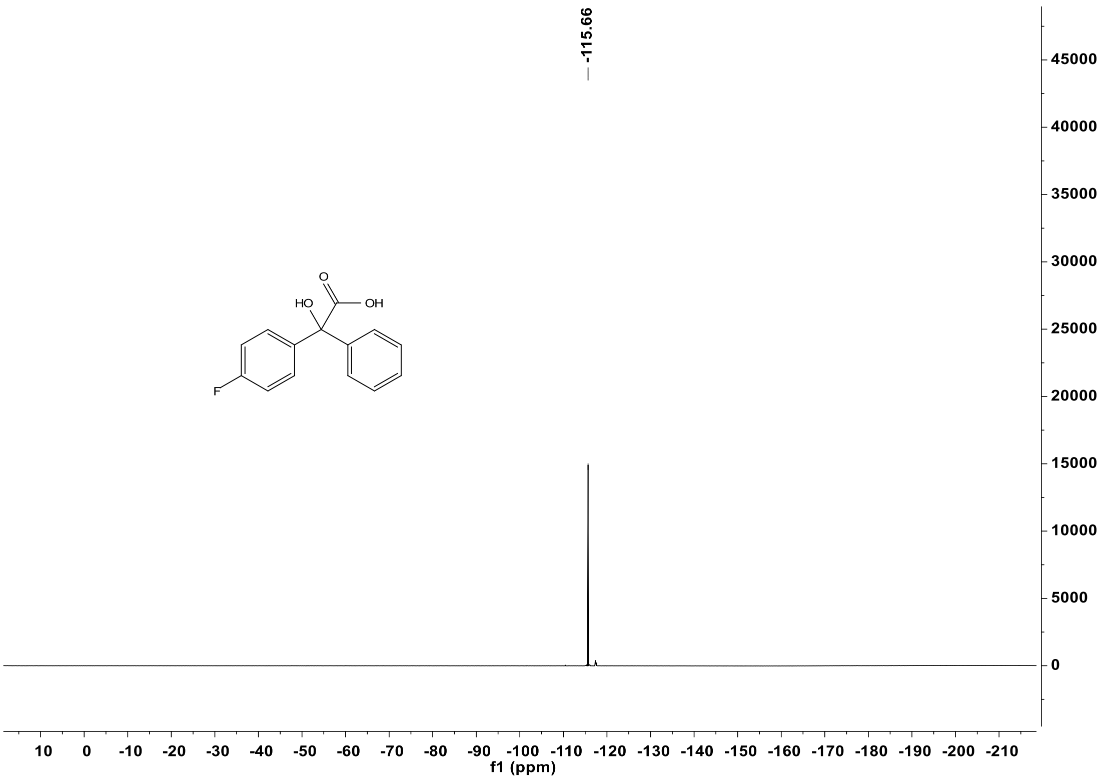

Supplementary Figure 42. ^{19}F NMR spectra of compound **4f**.

2-(4-chlorophenyl)-2-hydroxy-2-phenylacetic acid (4g)

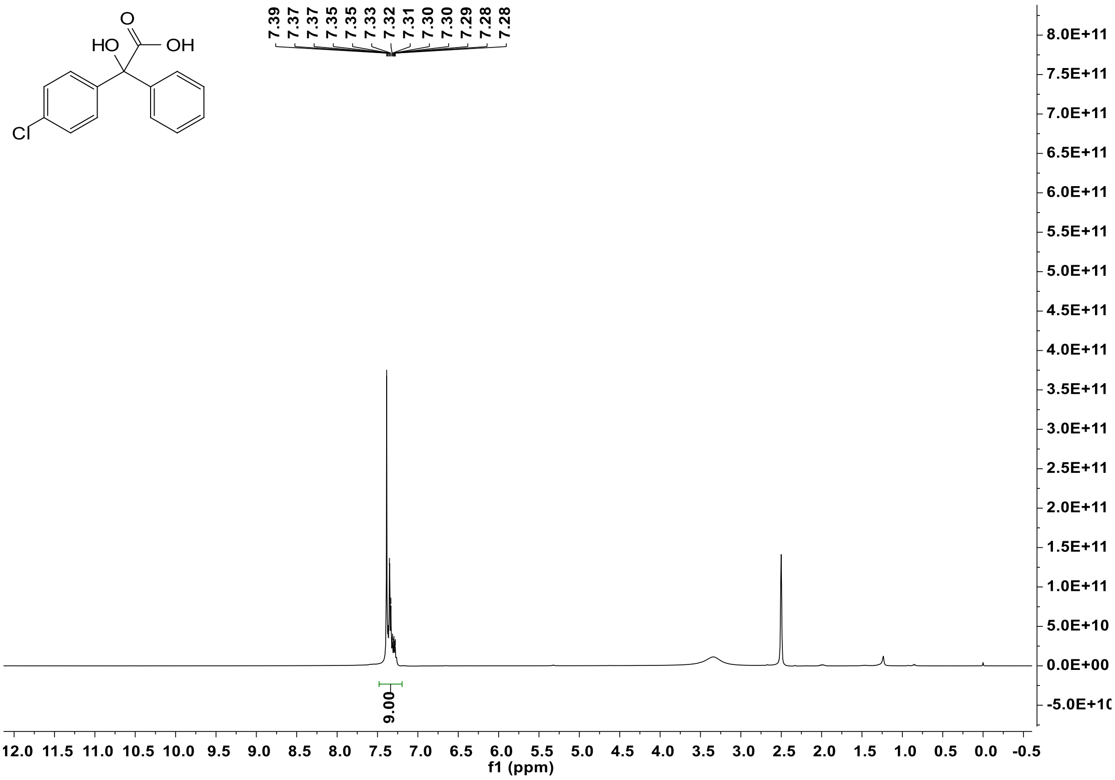

Supplementary Figure 43. ^{1}H NMR spectra of compound **4g**.

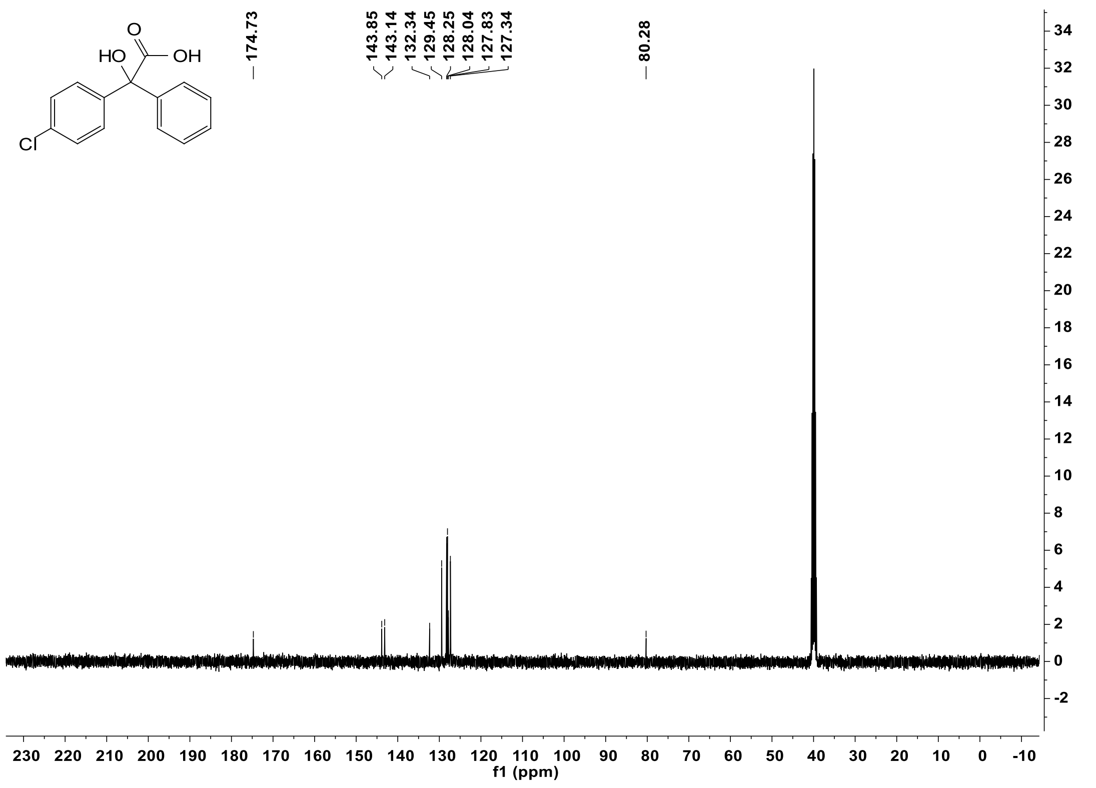

Supplementary Figure 44. ^{13}C NMR spectra of compound **4g**.

2-(3-fluorophenyl)-2-hydroxy-2-phenylacetic acid (4h)

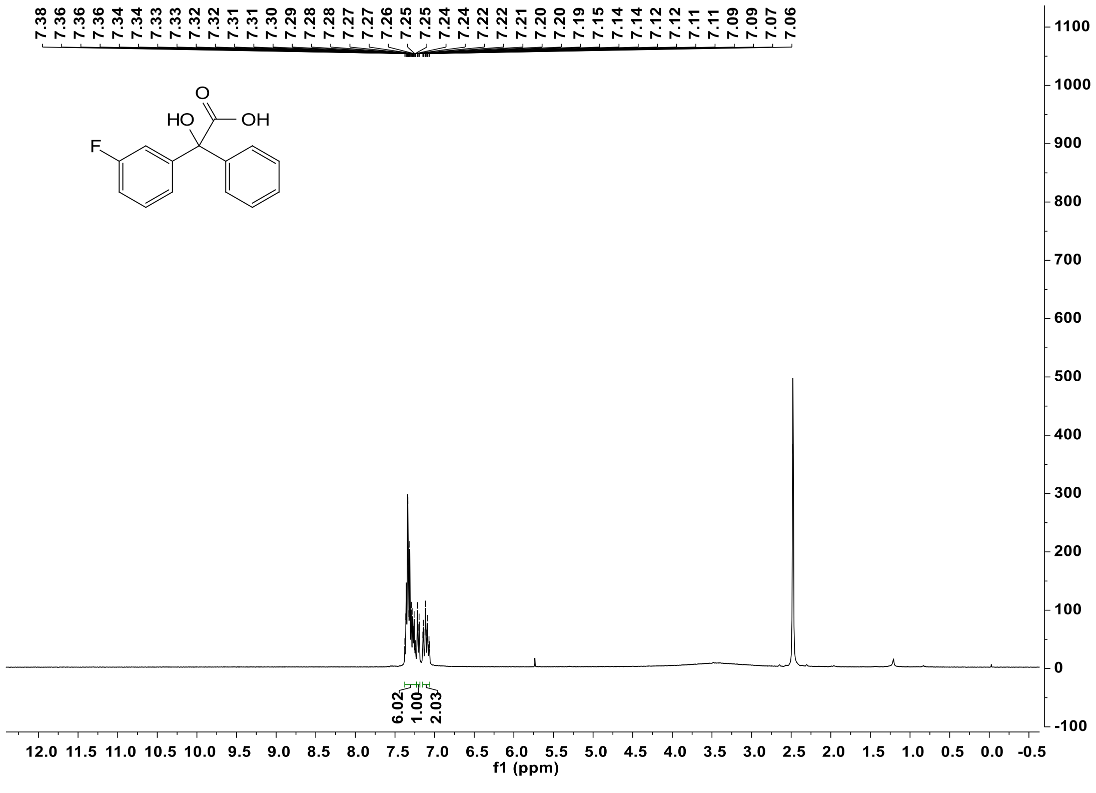

Supplementary Figure 45. ^{1}H NMR spectra of compound **4h**.

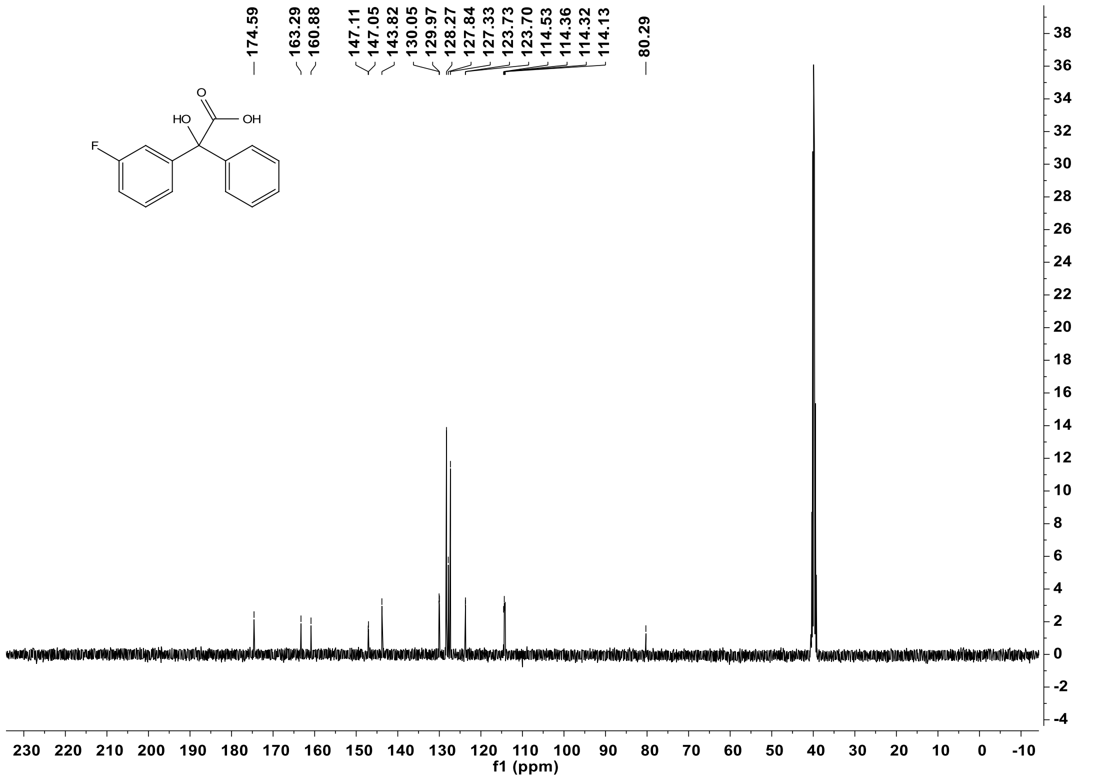

Supplementary Figure 46. ^{13}C NMR spectra of compound **4h**.

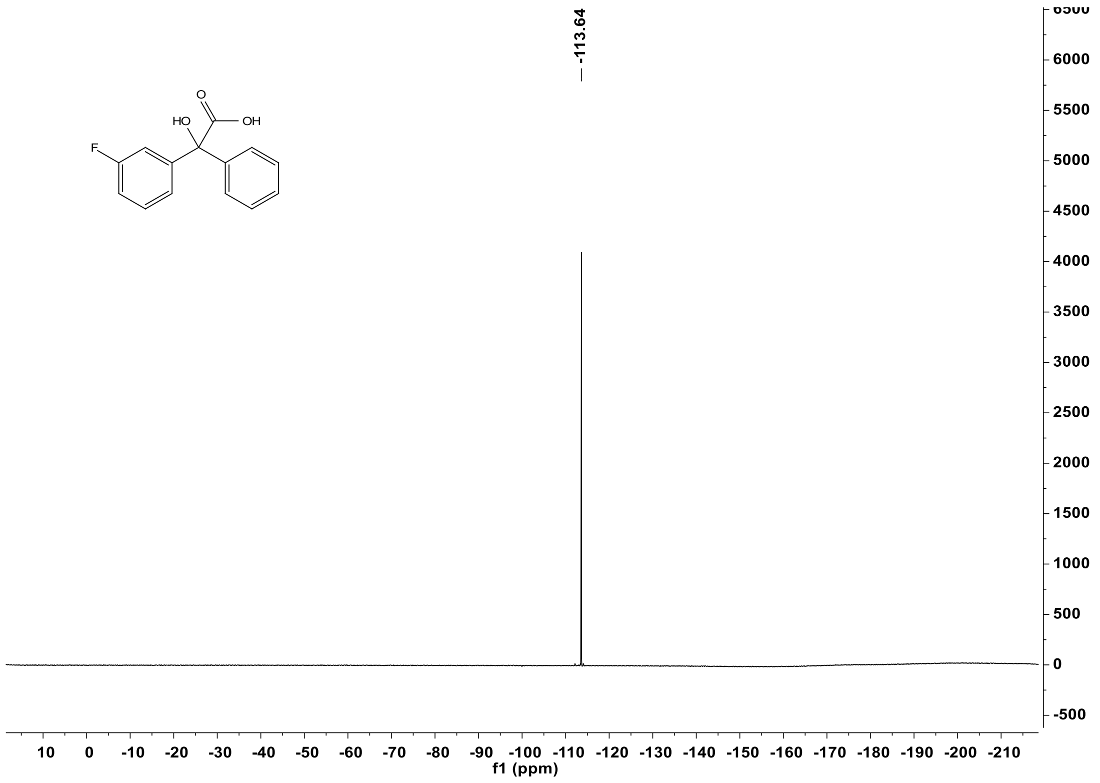

Supplementary Figure 47. ^{19}F NMR spectra of compound **4h**.

2-(3-chlorophenyl)-2-hydroxy-2-phenylacetic acid (4i)

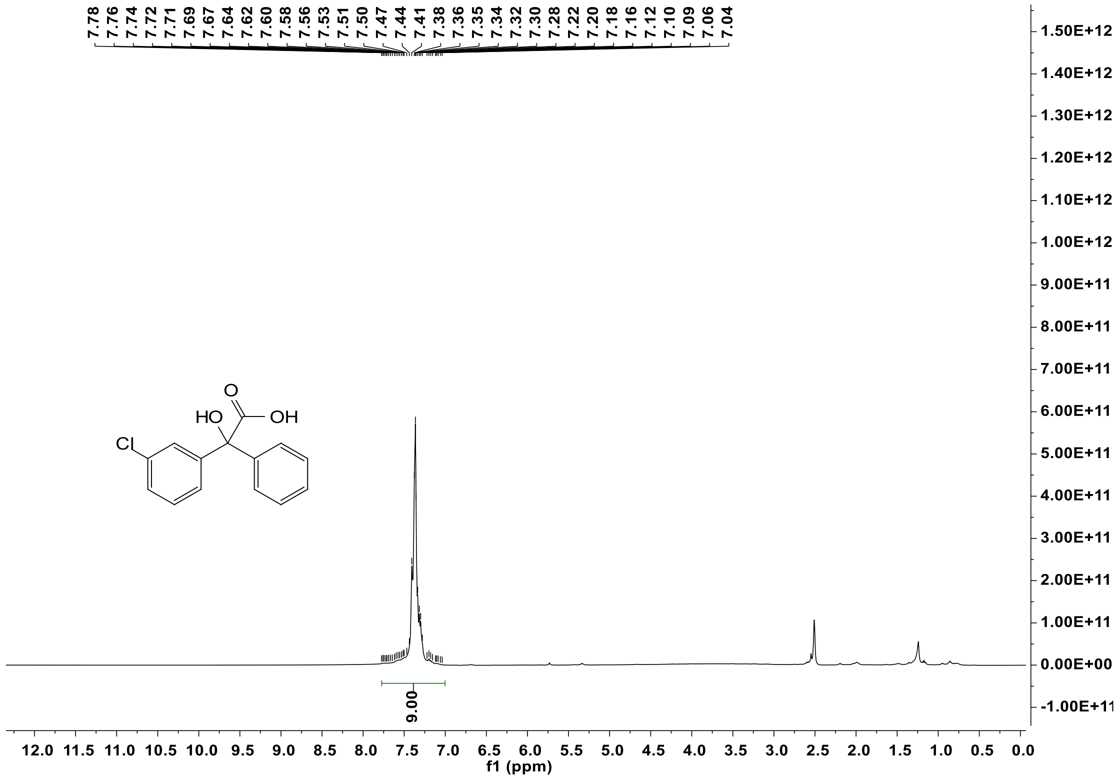

Supplementary Figure 48. ^{1}H NMR spectra of compound **4i**.

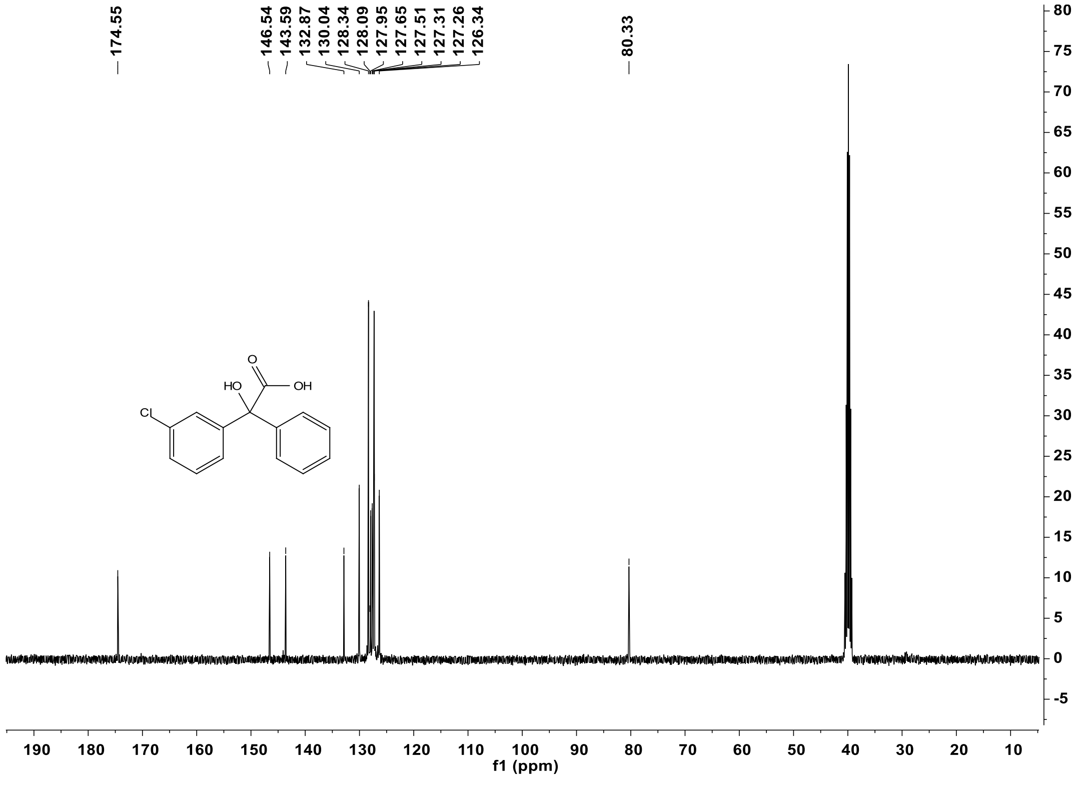

Supplementary Figure 49. ^{13}C NMR spectra of compound **4i**.

2-(2-fluorophenyl)-2-hydroxy-2-phenylacetic acid (4j)

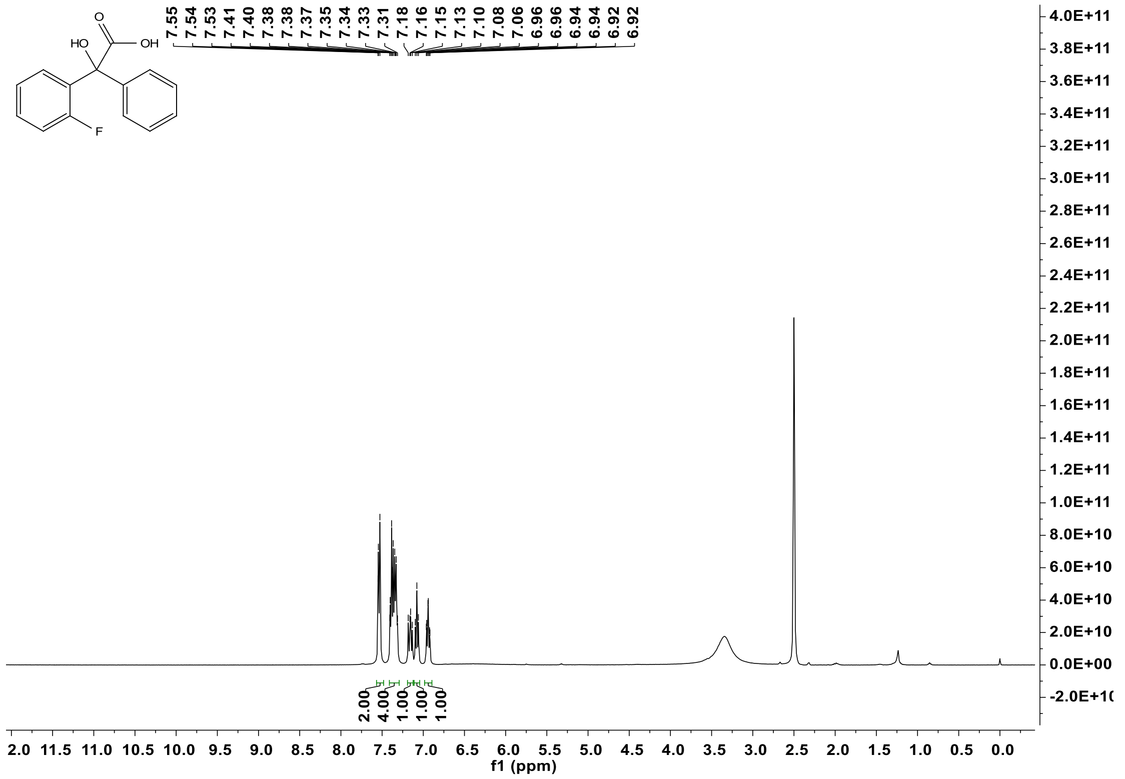

Supplementary Figure 50. ^{1}H NMR spectra of compound **4j**.

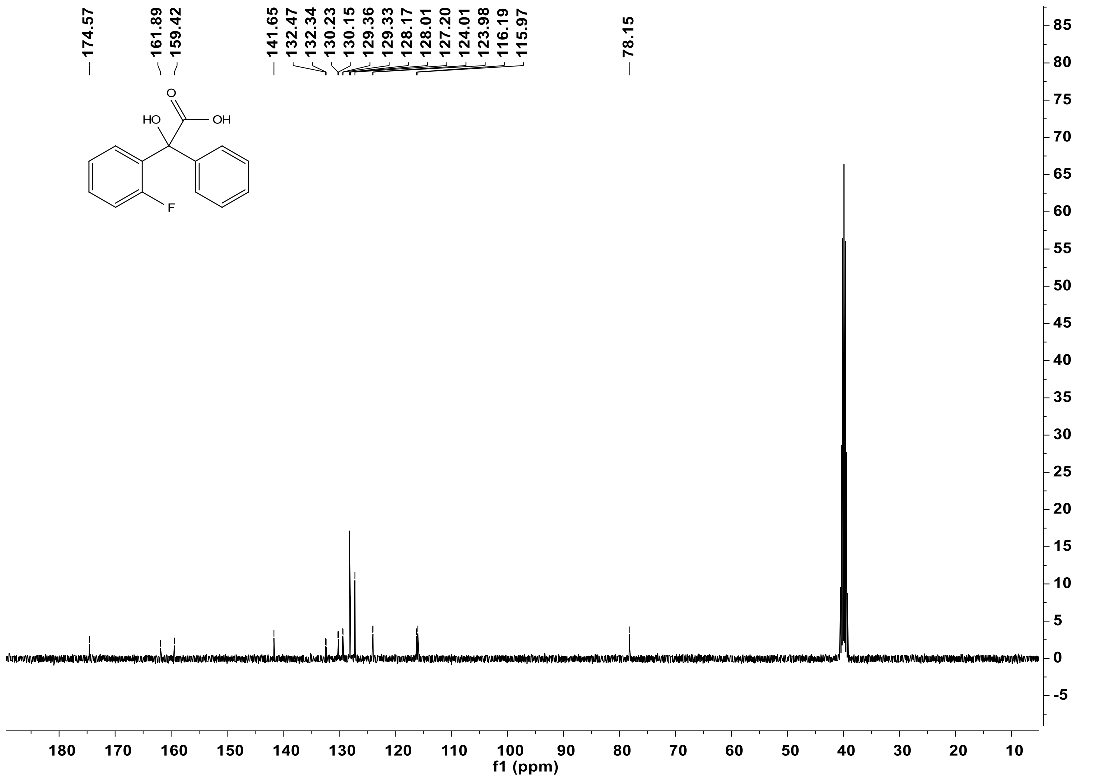

Supplementary Figure 51. ^{13}C NMR spectra of compound **4j**.

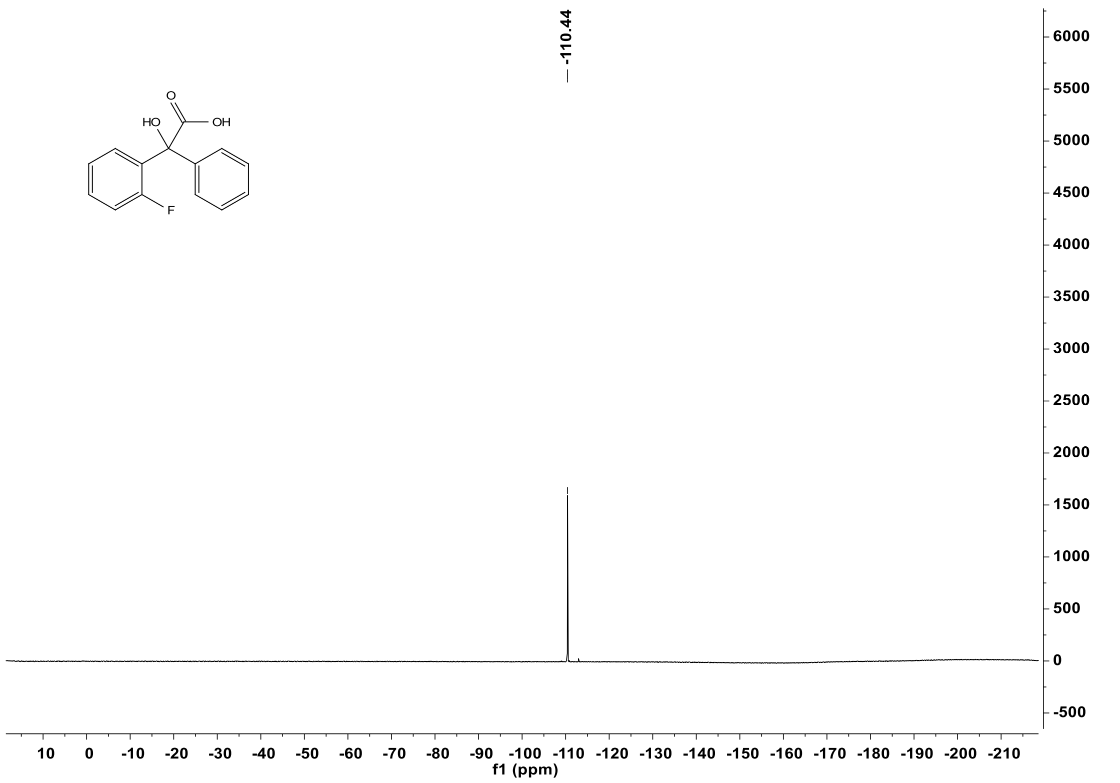

Supplementary Figure 52. ^{19}F NMR spectra of compound **4j**.

2-hydroxy-2-phenyl-2-(*o*-tolyl)acetic acid (4k)

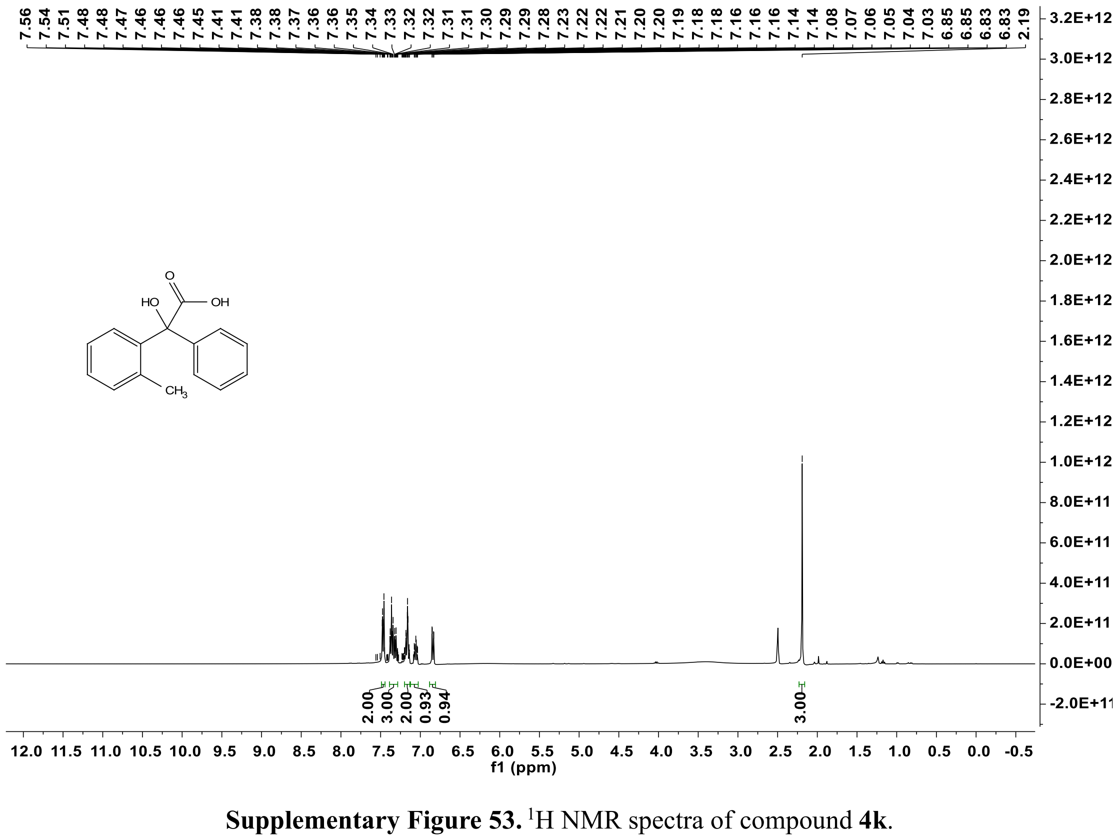

Supplementary Figure 53. ^{1}H NMR spectra of compound **4k**.

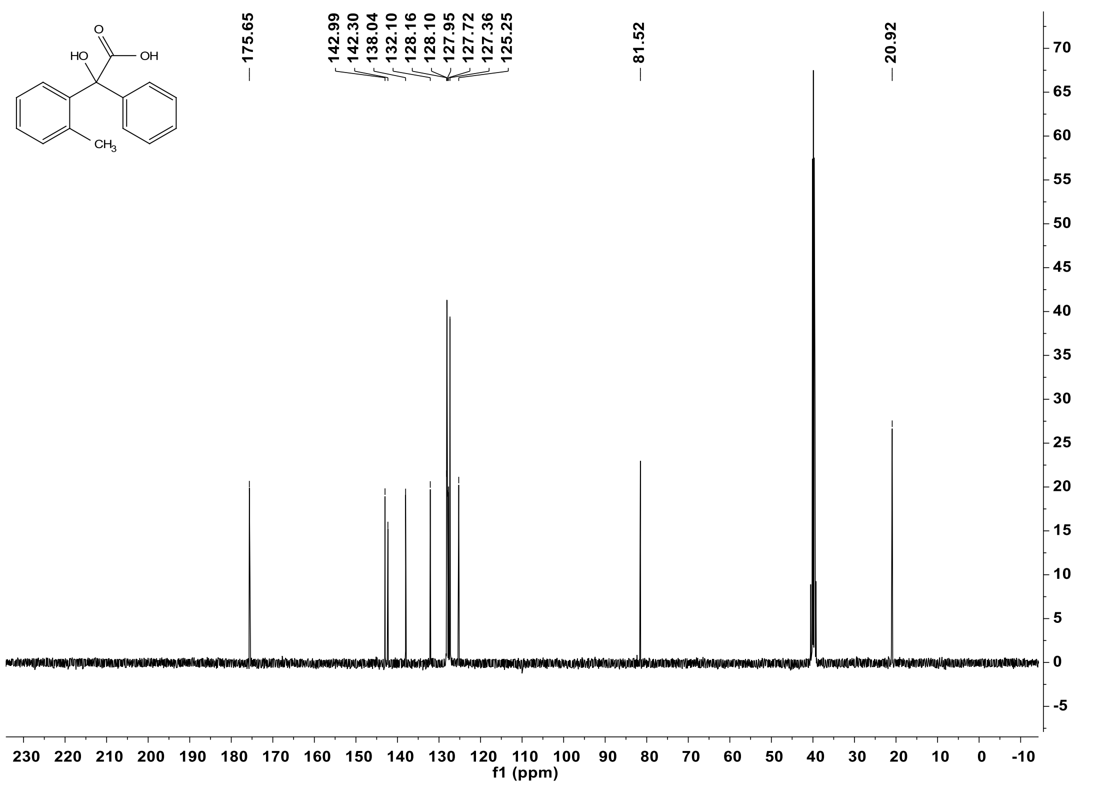

Supplementary Figure 54. ^{13}C NMR spectra of compound **4k**.

2,2-bis(4-fluorophenyl)-2-hydroxyacetic acid (4l)

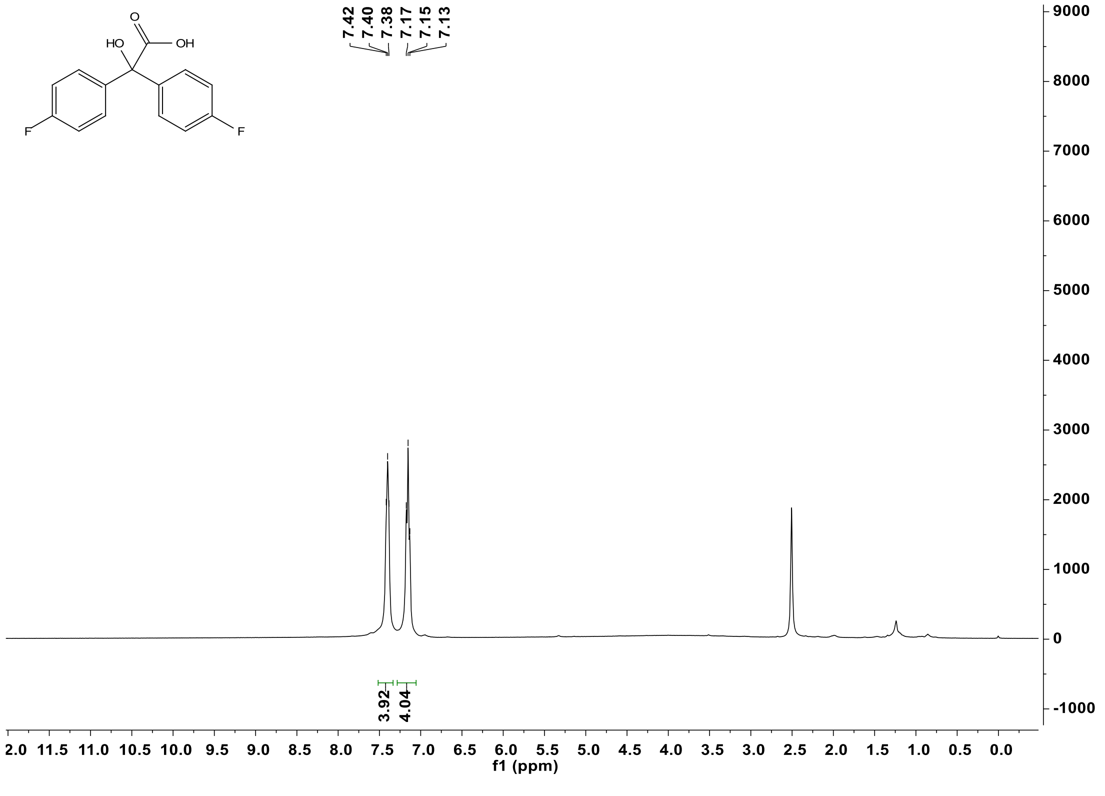

Supplementary Figure 55. ^{1}H NMR spectra of compound **4l**.

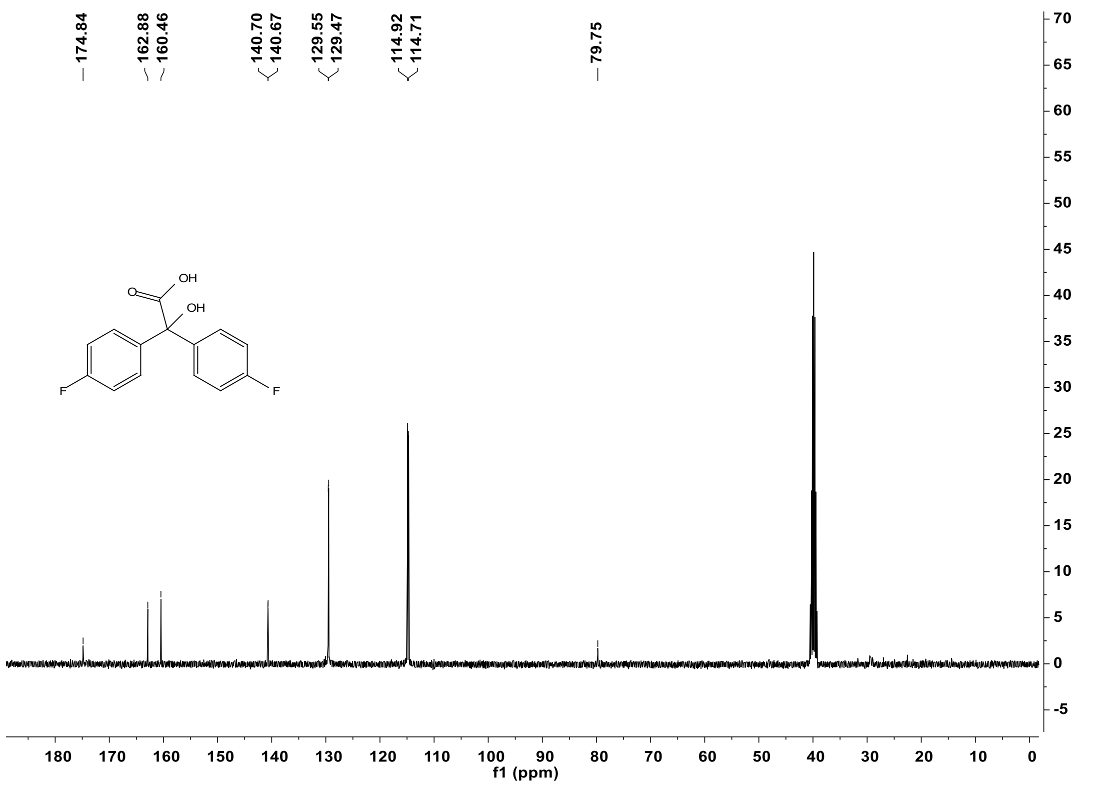

Supplementary Figure 56. ^{13}C NMR spectra of compound **4l**.

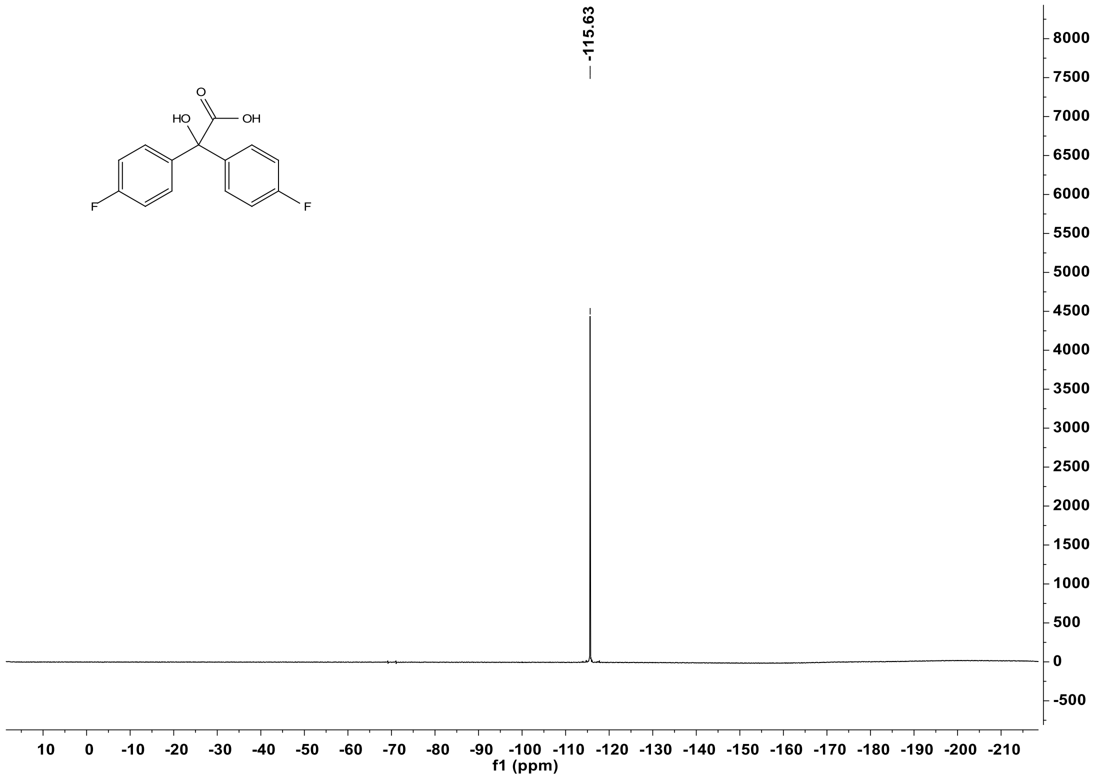

Supplementary Figure 57. ^{19}F NMR spectra of compound **4l**.

2,2-bis(4-chlorophenyl)-2-hydroxyacetic acid (4m)

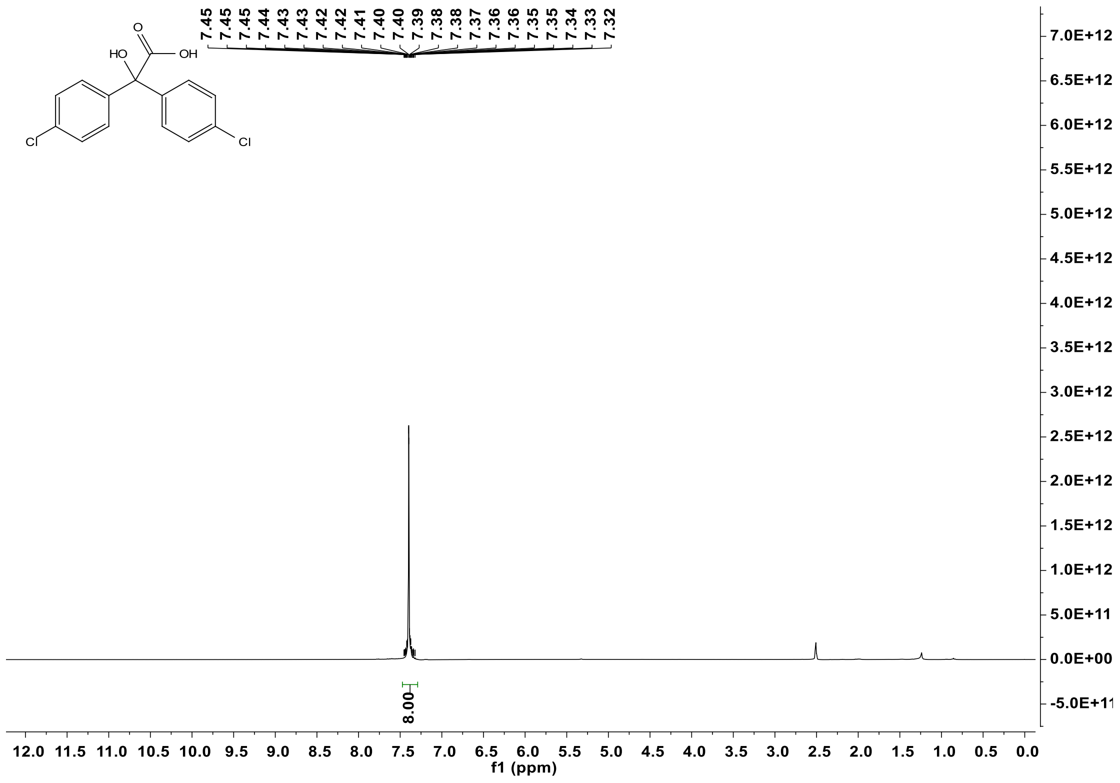

Supplementary Figure 58. ^{1}H NMR spectra of compound **4m**.

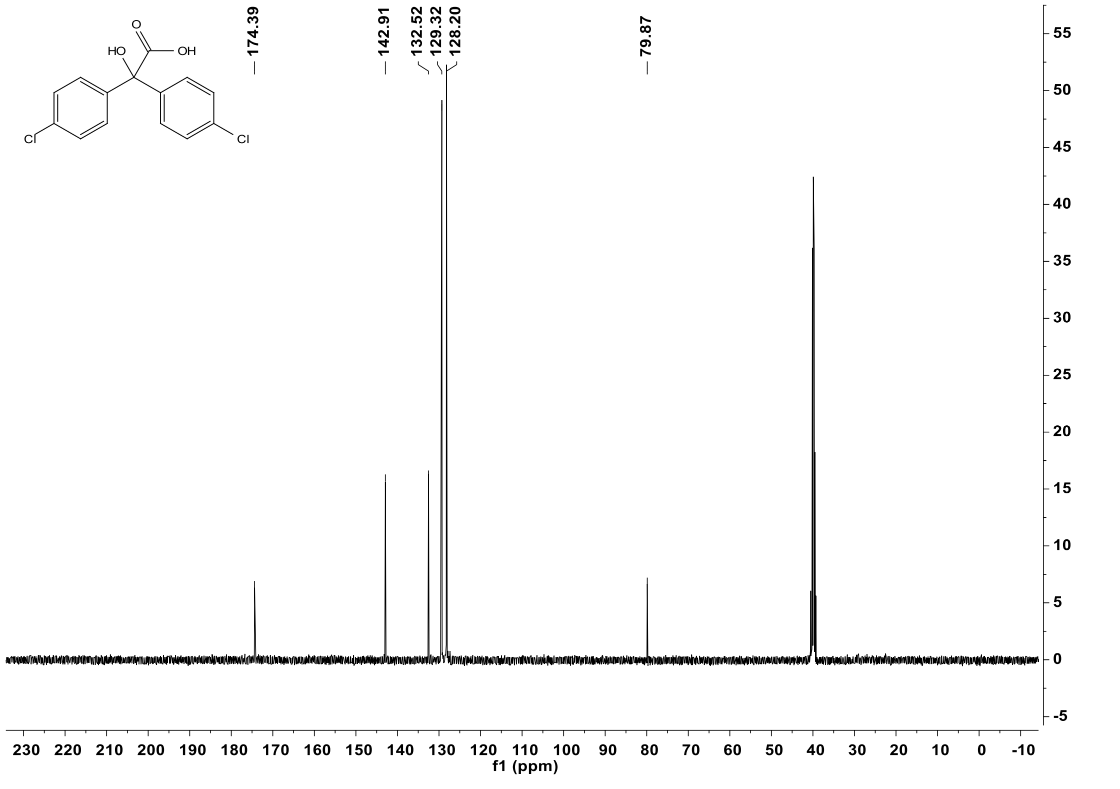

Supplementary Figure 59. ^{13}C NMR spectra of compound **4m**.

2-hydroxy-2,2-di-*p*-tolylacetic acid (4n)

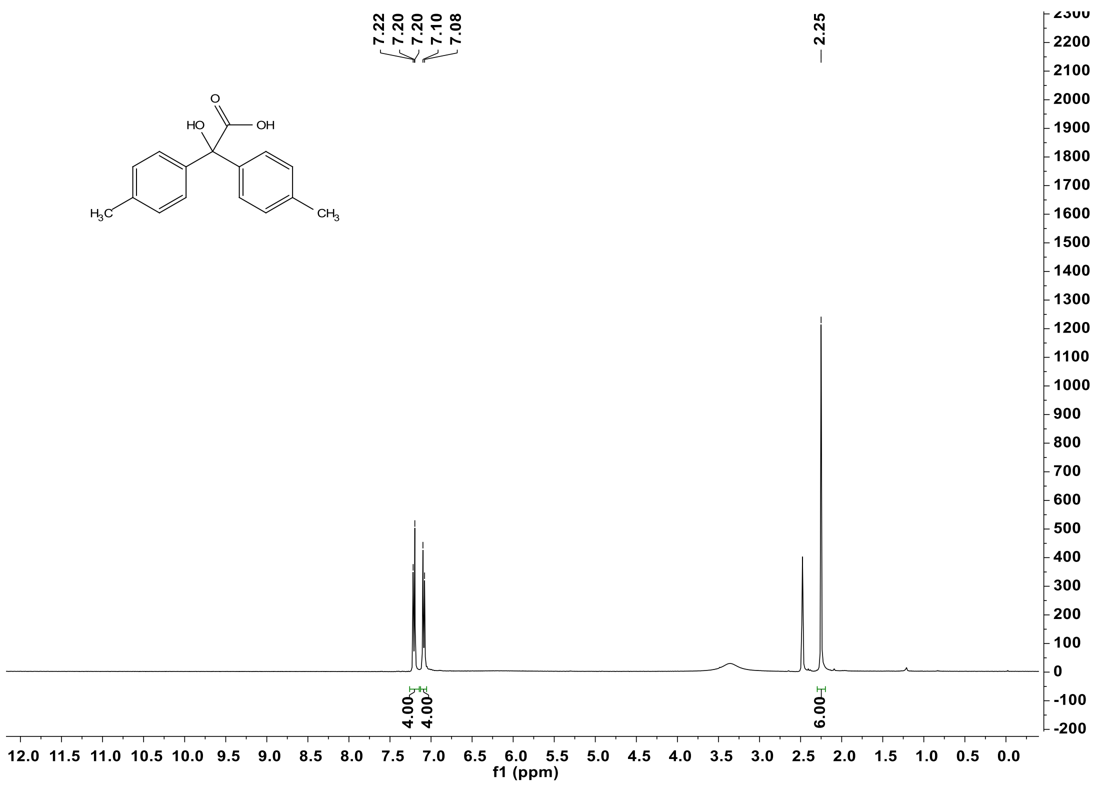

Supplementary Figure 60. ^{1}H NMR spectra of compound **4n**.

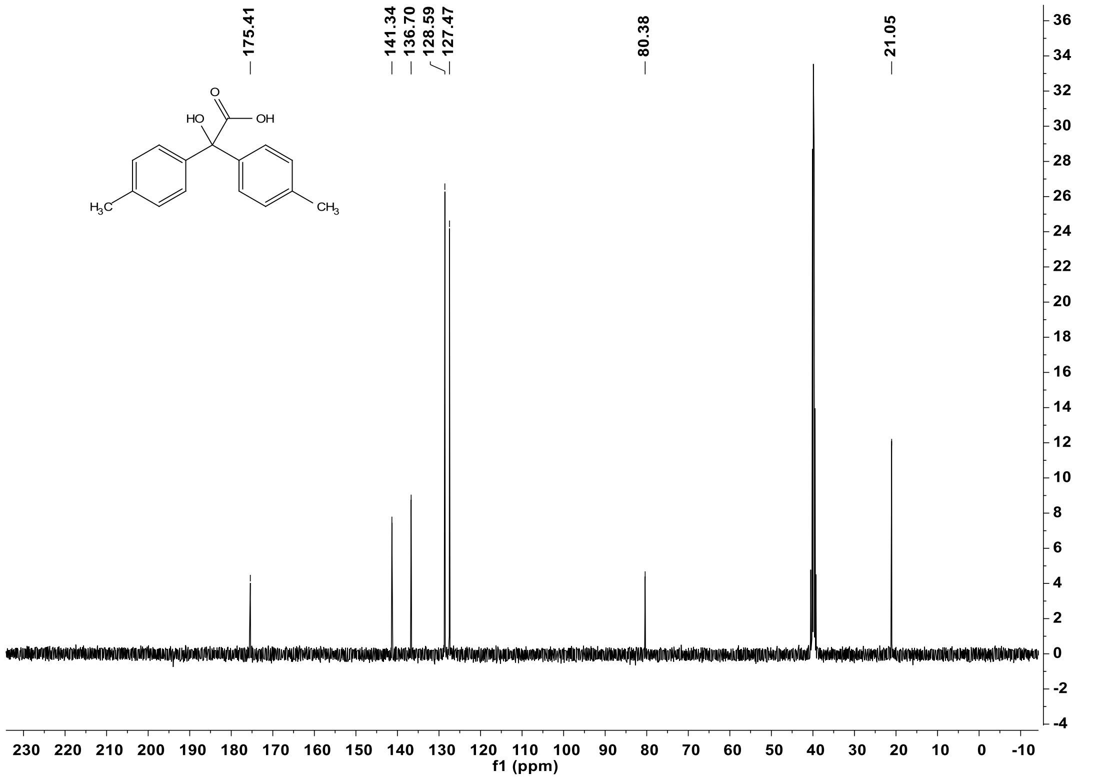

Supplementary Figure 61. ^{13}C NMR spectra of compound **4n**.

2-(4-fluorophenyl)-2-hydroxy-2-(*p*-tolyl)acetic acid (4o)

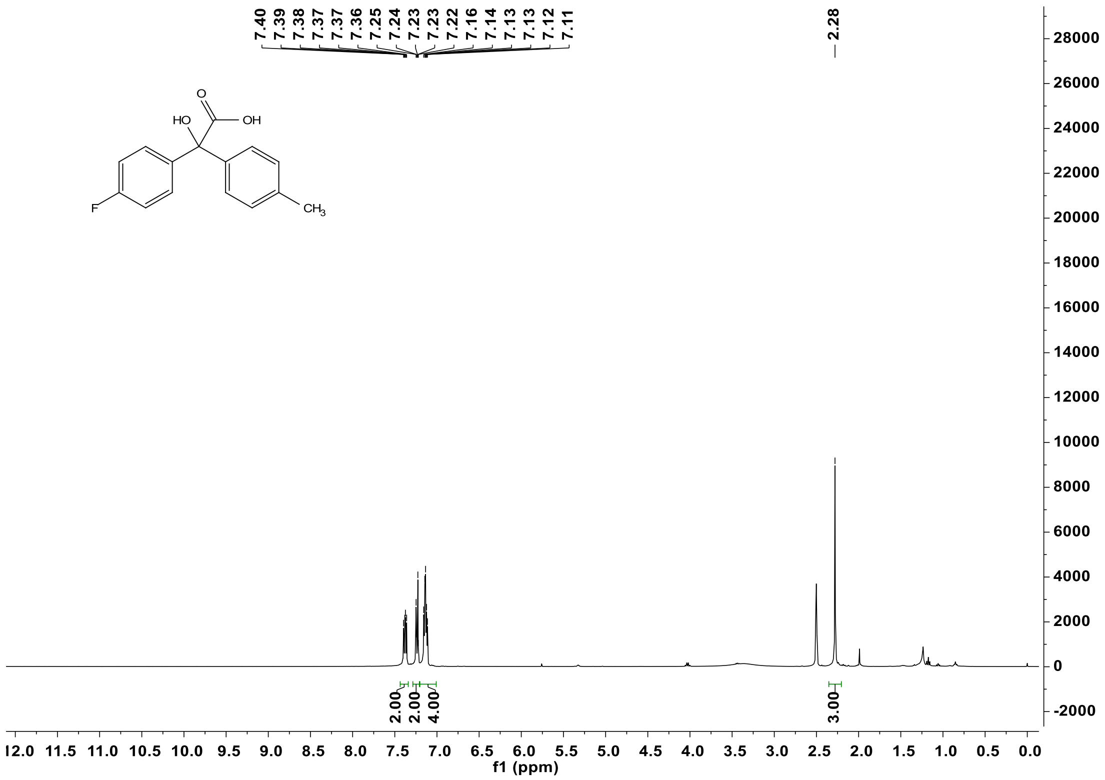

Supplementary Figure 62. ^{1}H NMR spectra of compound **4o**.

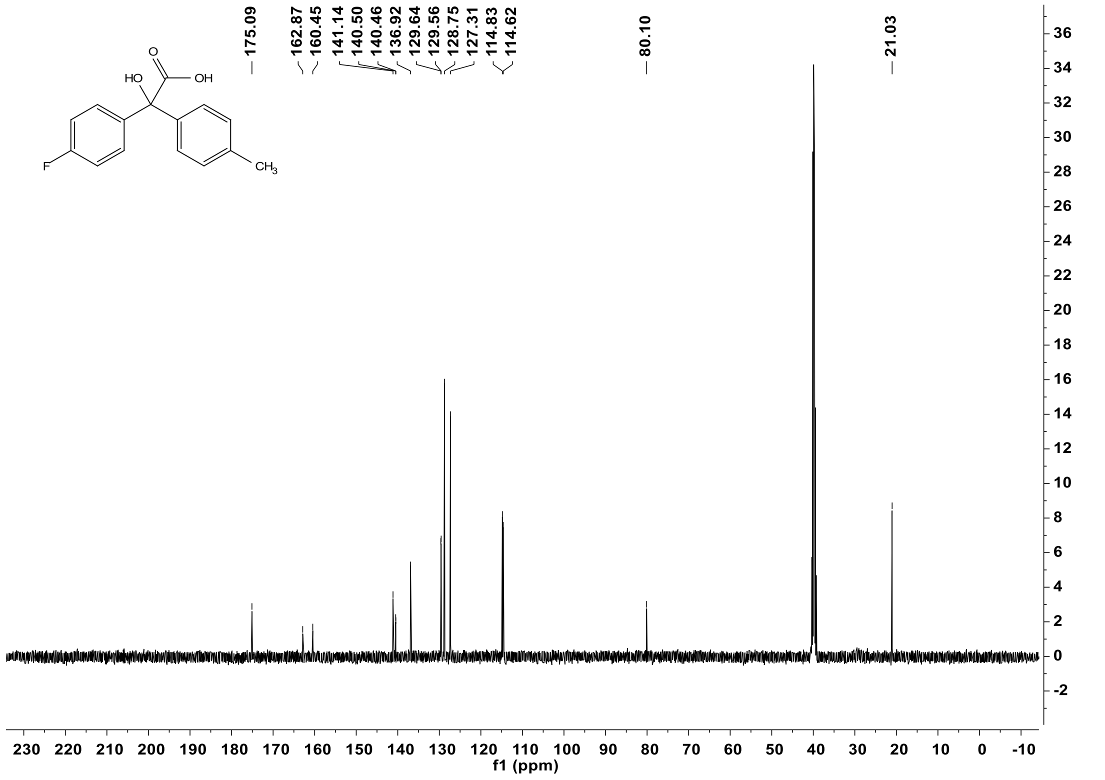

Supplementary Figure 63. ^{13}C NMR spectra of compound **4o**.

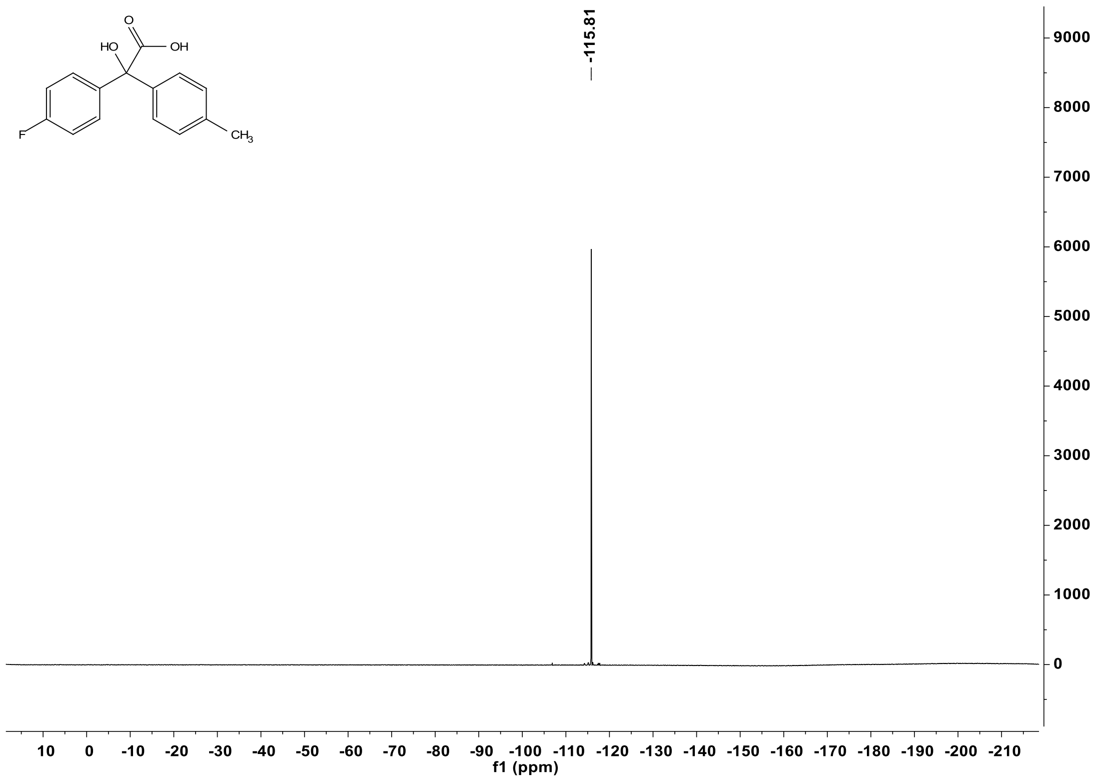

Supplementary Figure 64. ^{19}F NMR spectra of compound **4o**.

methyl 9-hydroxy-9*H*-xanthene-9-carboxylate (4p)

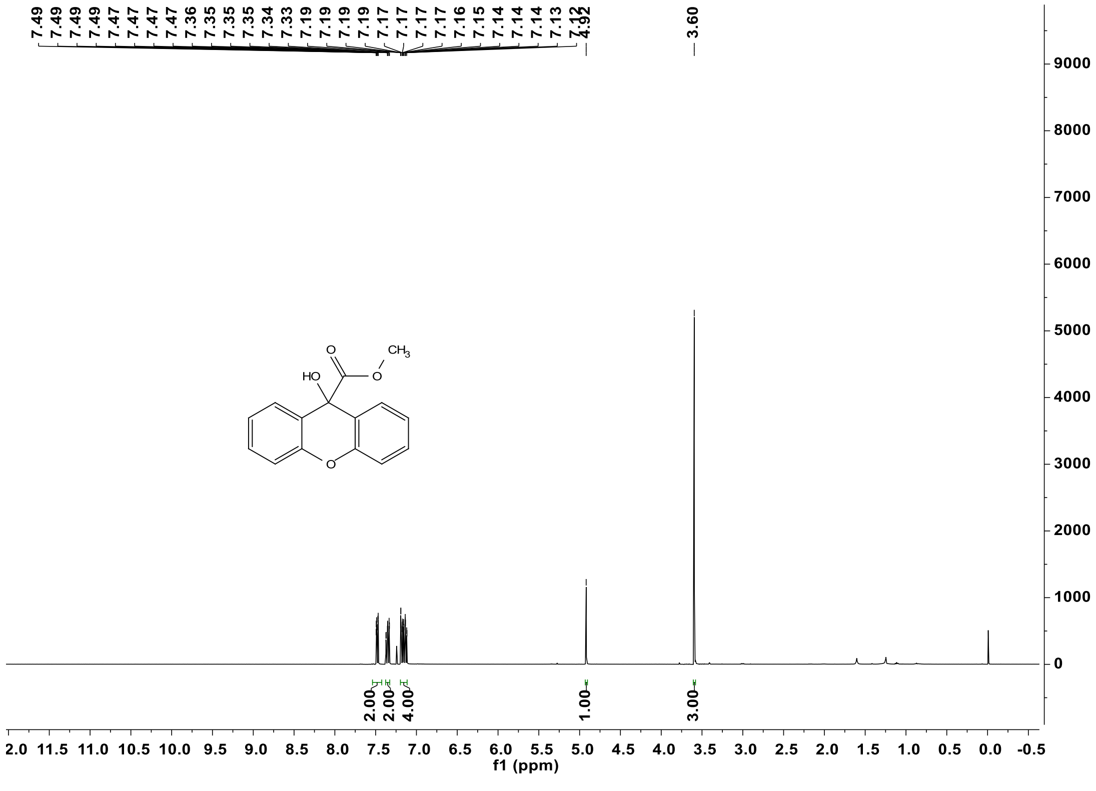

Supplementary Figure 65. ^{1}H NMR spectra of compound **4p**.

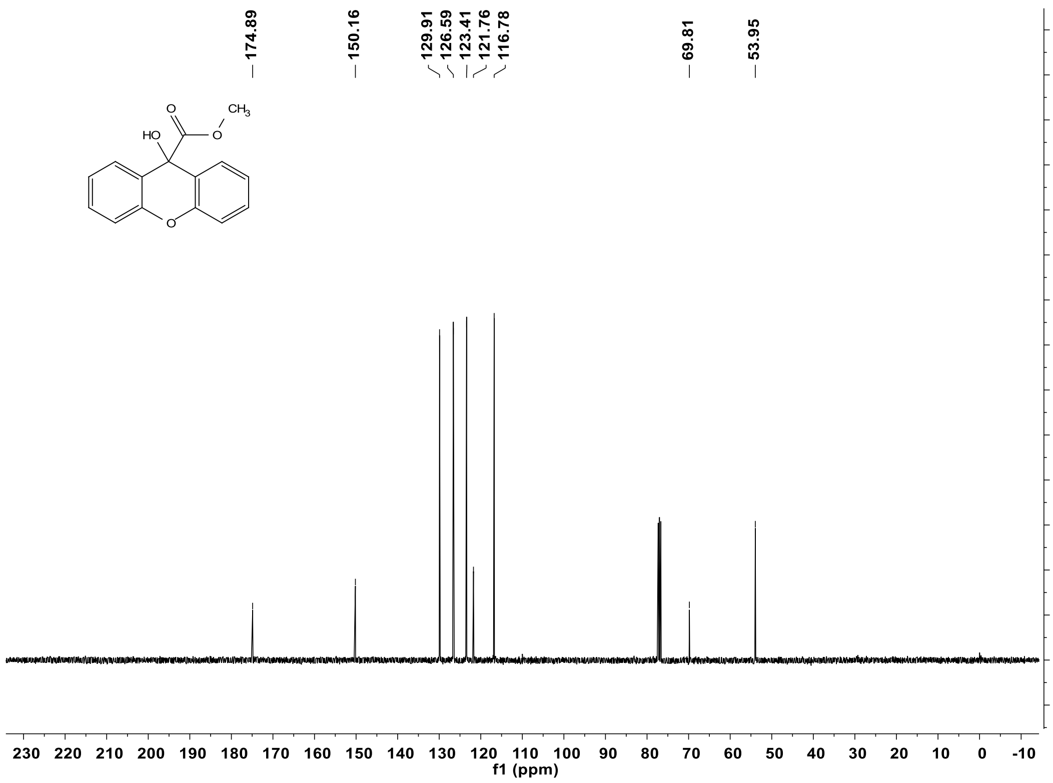

Supplementary Figure 66. ^{13}C NMR spectra of compound **4p**.

methyl 2-hydroxy-2-phenyl-2-(thiophen-2-yl)acetate (4q)

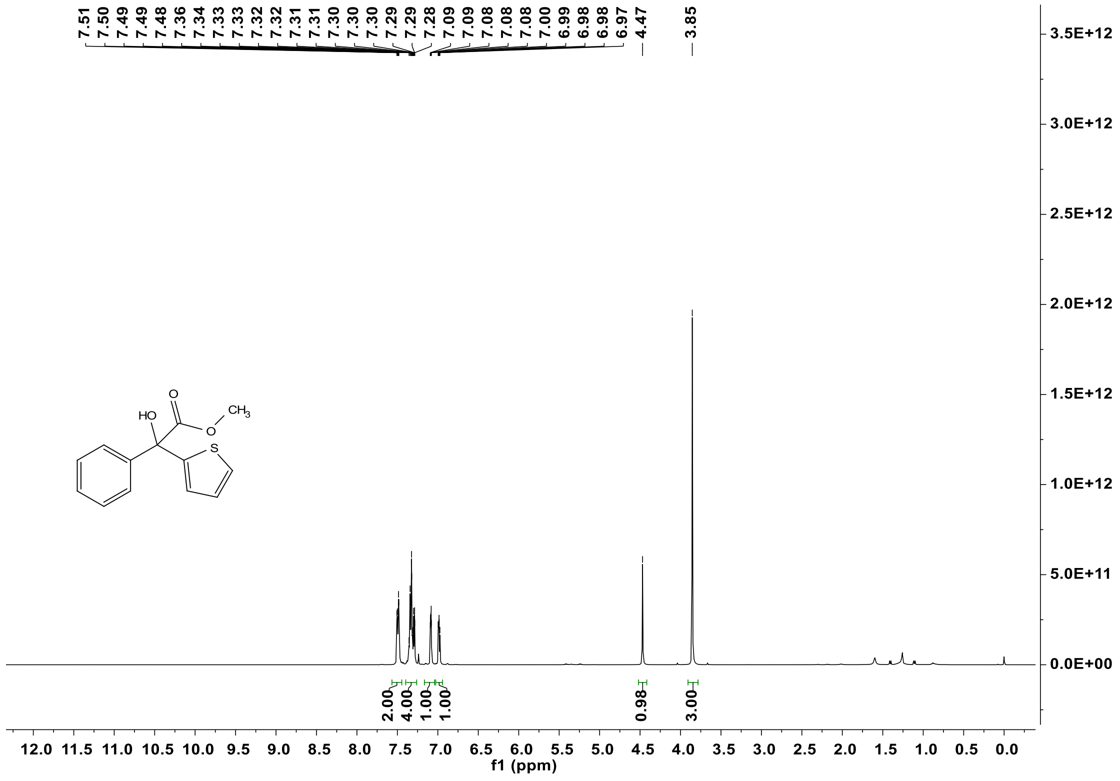

Supplementary Figure 67. ^{1}H NMR spectra of compound **4q**.

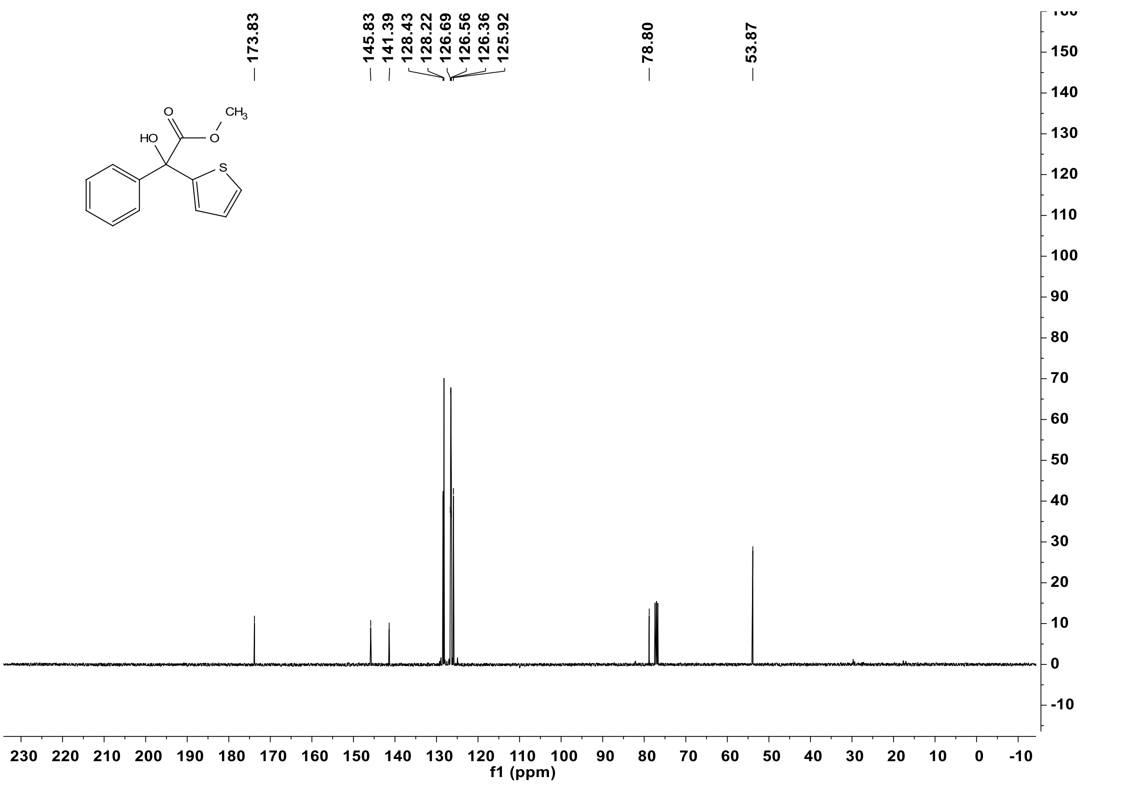

Supplementary Figure 68. ^{13}C NMR spectra of compound **4q**.

methyl 2-hydroxy-2,2-di(thiophen-2-yl)acetate (4r)

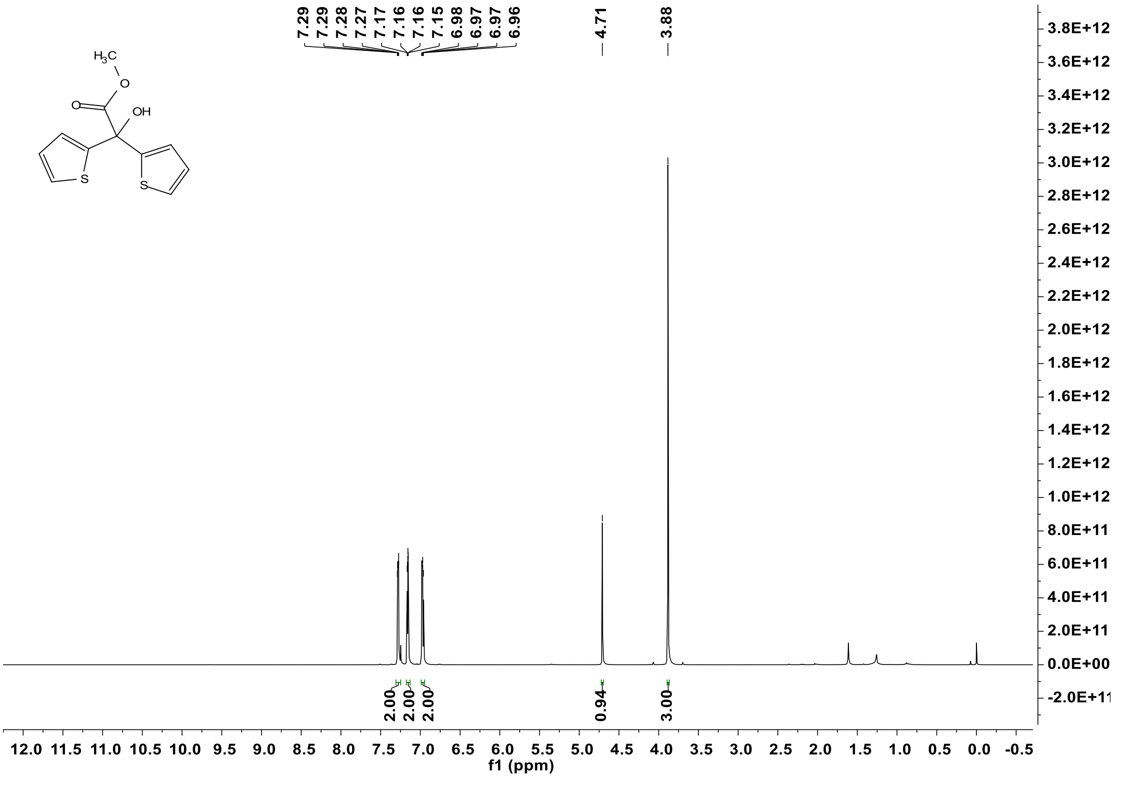

Supplementary Figure 69. ^{1}H NMR spectra of compound **4r**.

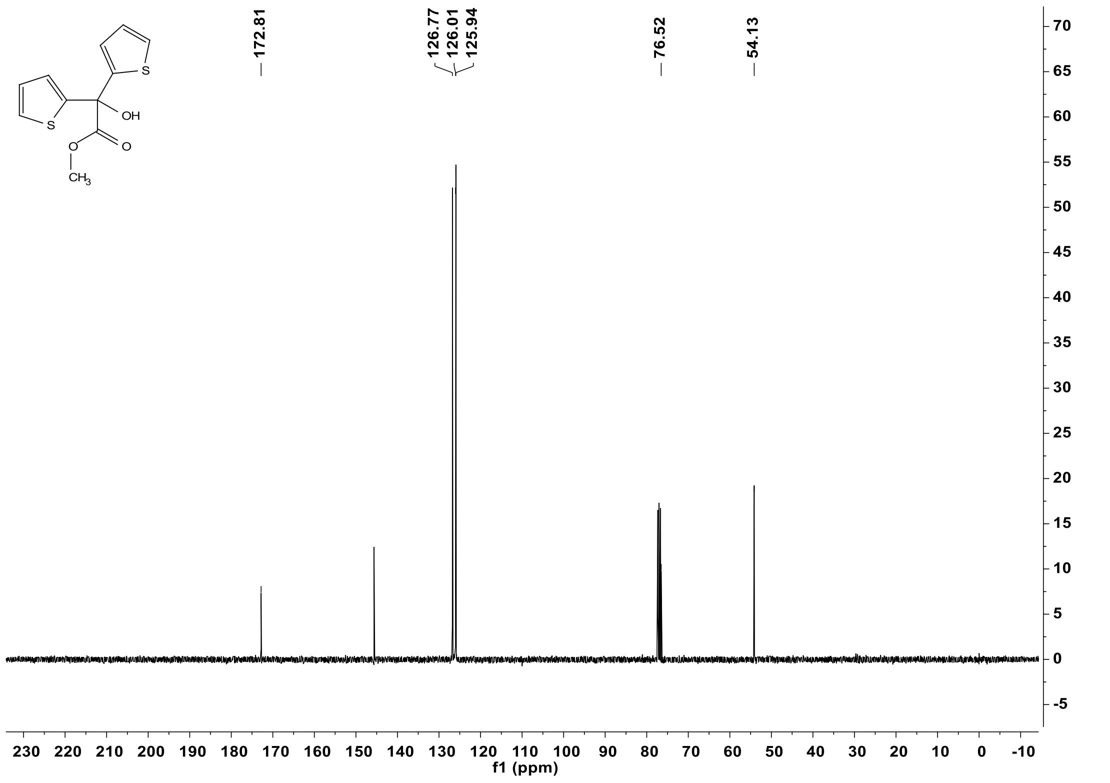

Supplementary Figure 70. ^{13}C NMR spectra of compound **4r**.

2-hydroxy-3,3-dimethyl-2-(((2-methyl-1-phenylpropan-2-yl)oxy)carbonyl)butanoic acid (6a)

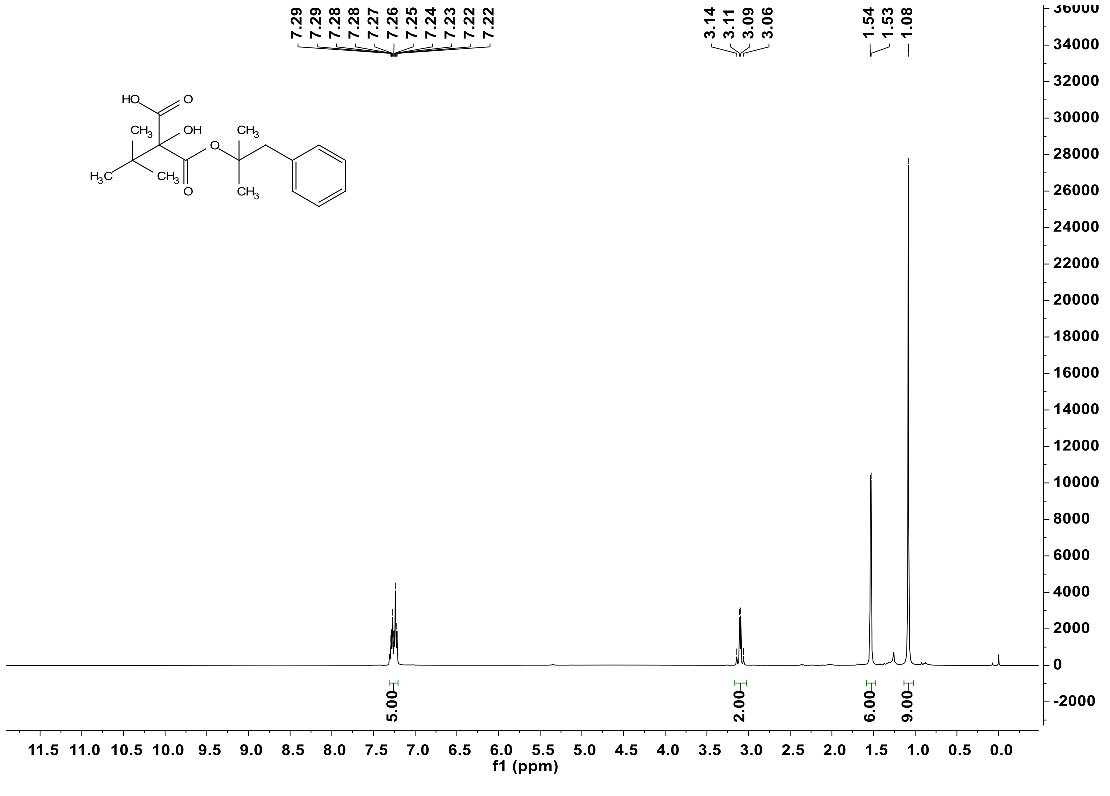

Supplementary Figure 71. ^{1}H NMR spectra of compound **6a**.

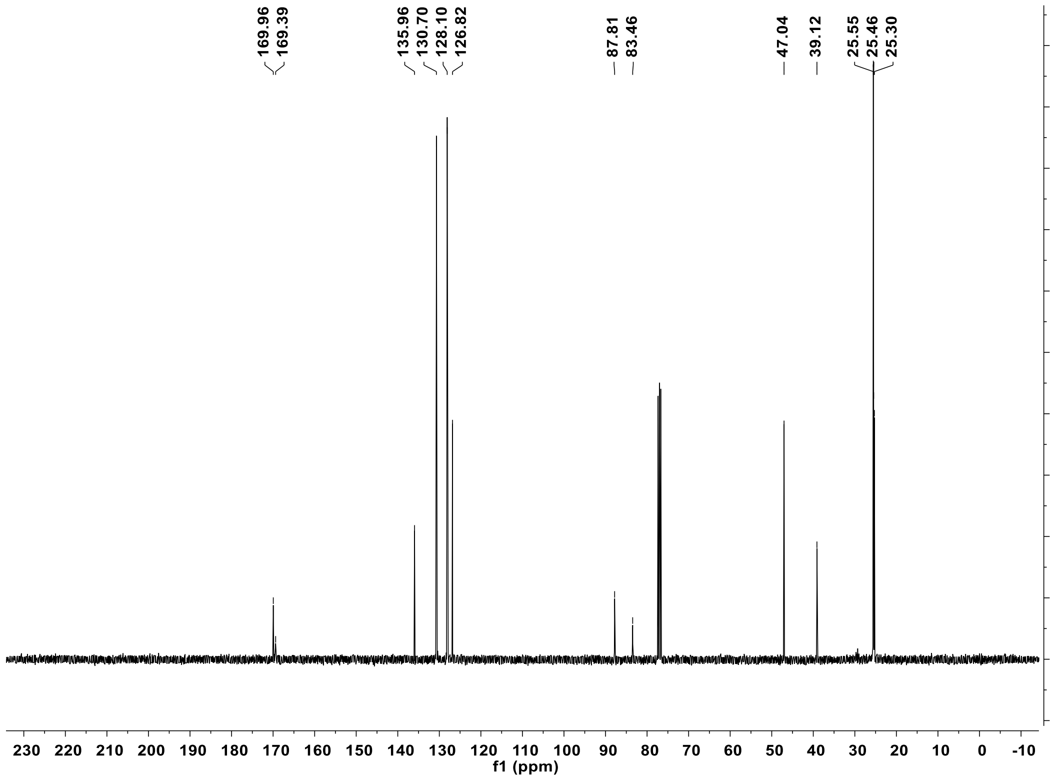

Supplementary Figure 72. ^{13}C NMR spectra of compound **6a**.

2-(*tert*-butoxycarbonyl)-2-hydroxy-3-methylhexanoic acid (6b)

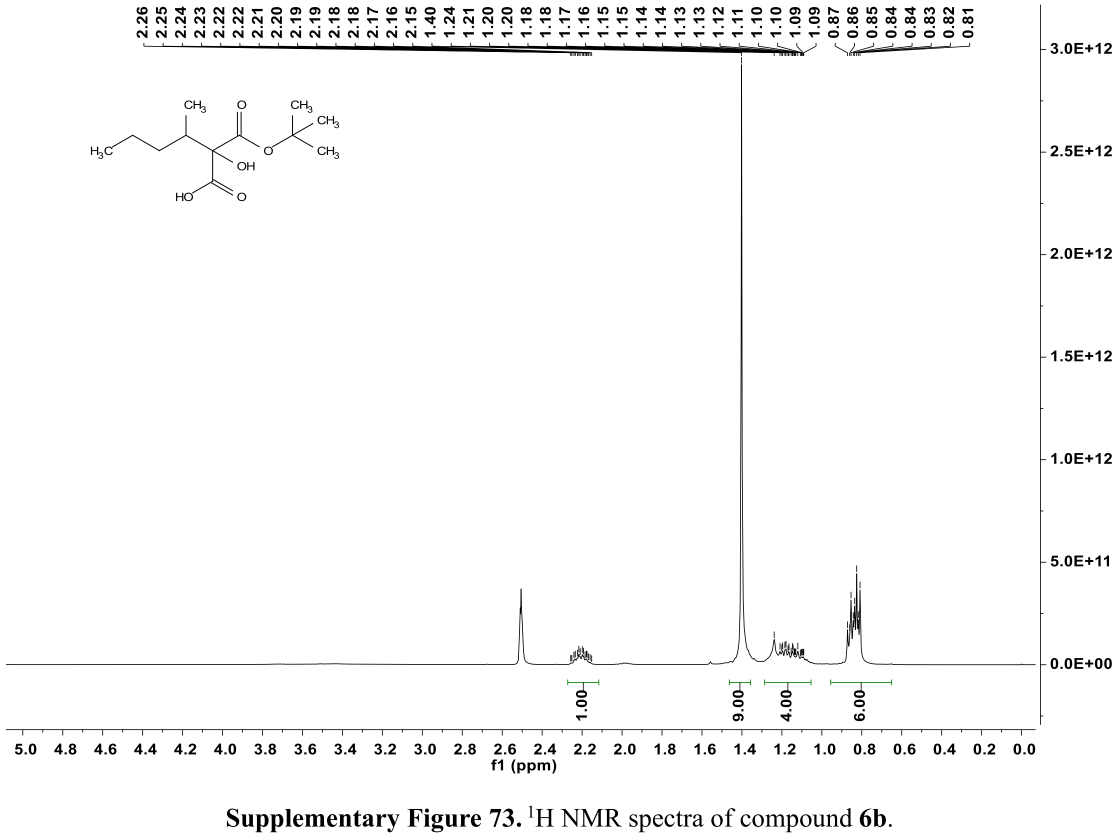

Supplementary Figure 73. ^{1}H NMR spectra of compound **6b**.

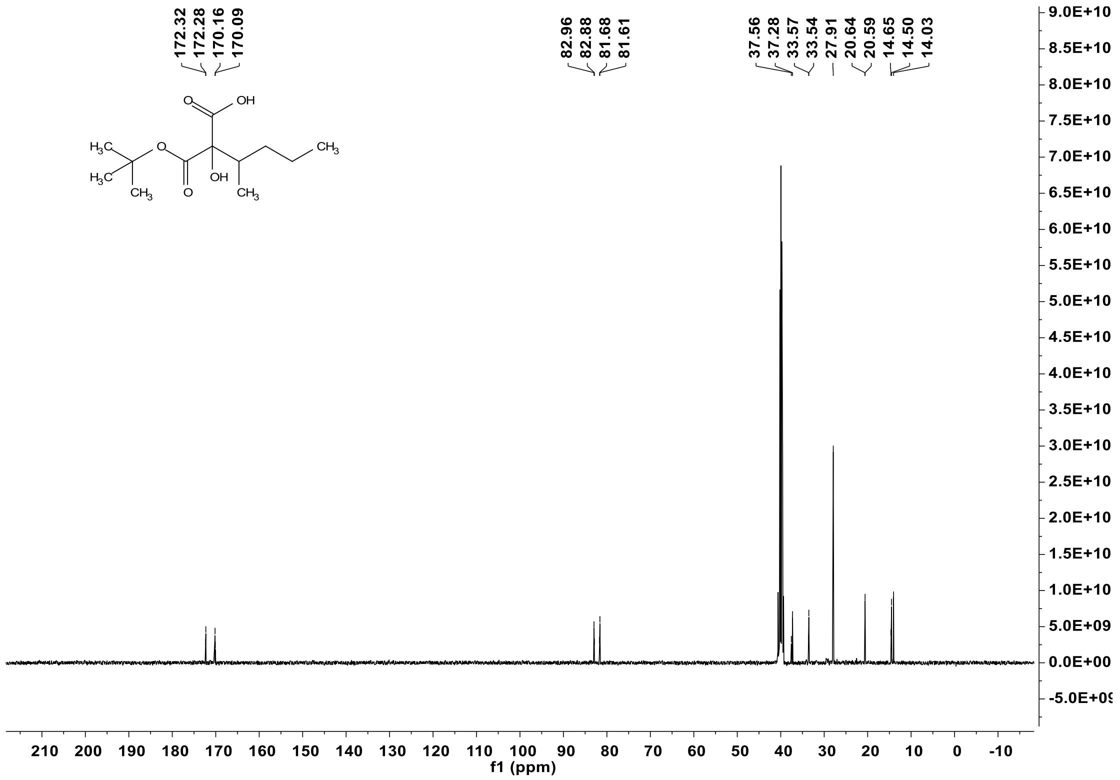

Supplementary Figure 74. ^{13}C NMR spectra of compound **6b**.

2-(*tert*-butoxycarbonyl)-2-hydroxyhexanoic acid (6c)

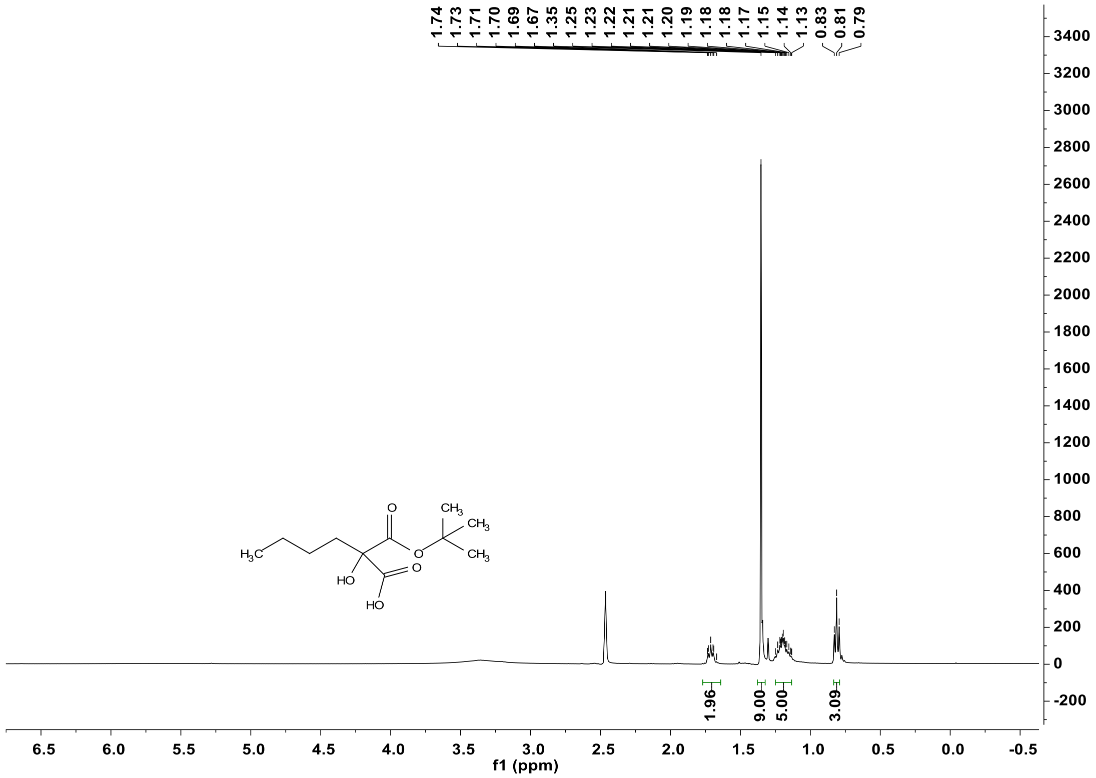

Supplementary Figure 75. ^{1}H NMR spectra of compound **6c**.

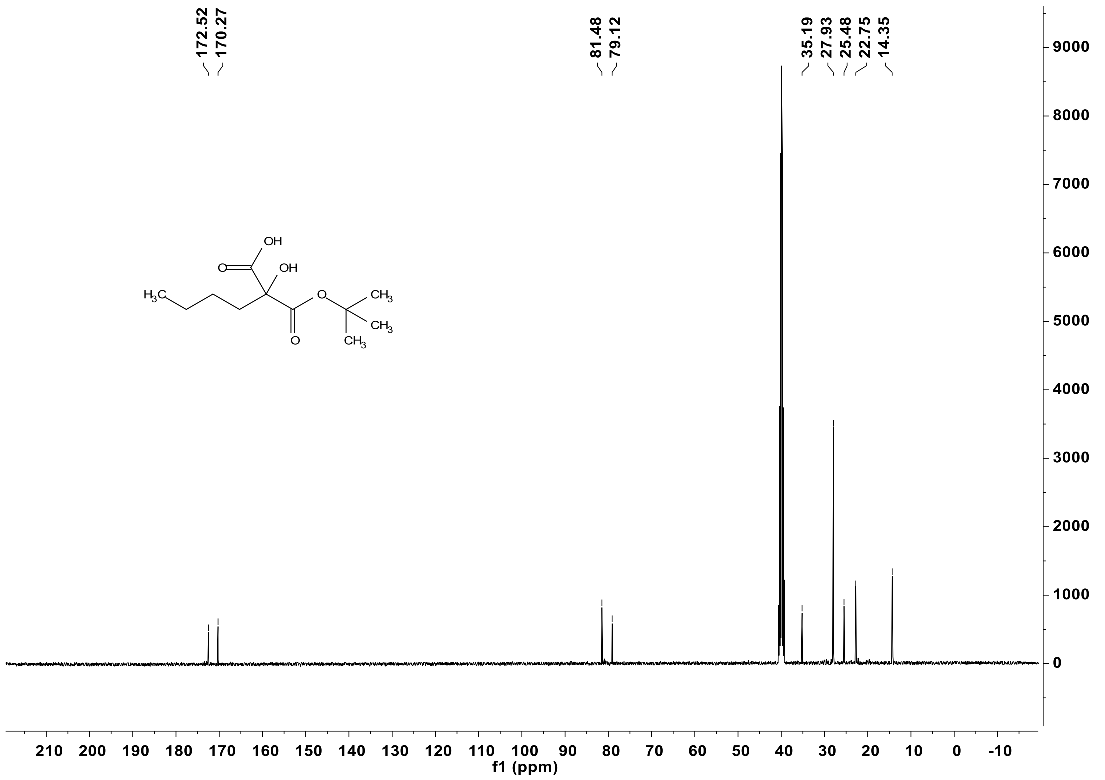

Supplementary Figure 76. ^{13}C NMR spectra of compound **6c**.

2-(*tert*-butoxycarbonyl)-2-hydroxyhex-5-enoic acid (6d)

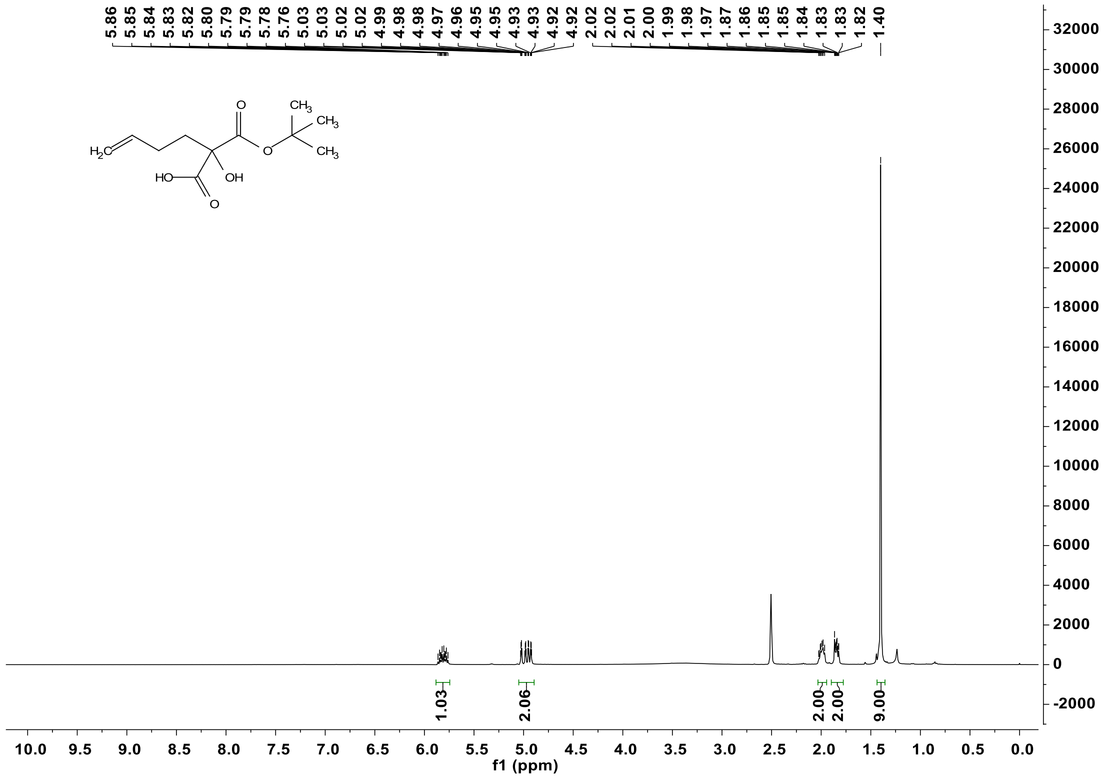

Supplementary Figure 77. ^{1}H NMR spectra of compound **6d**.

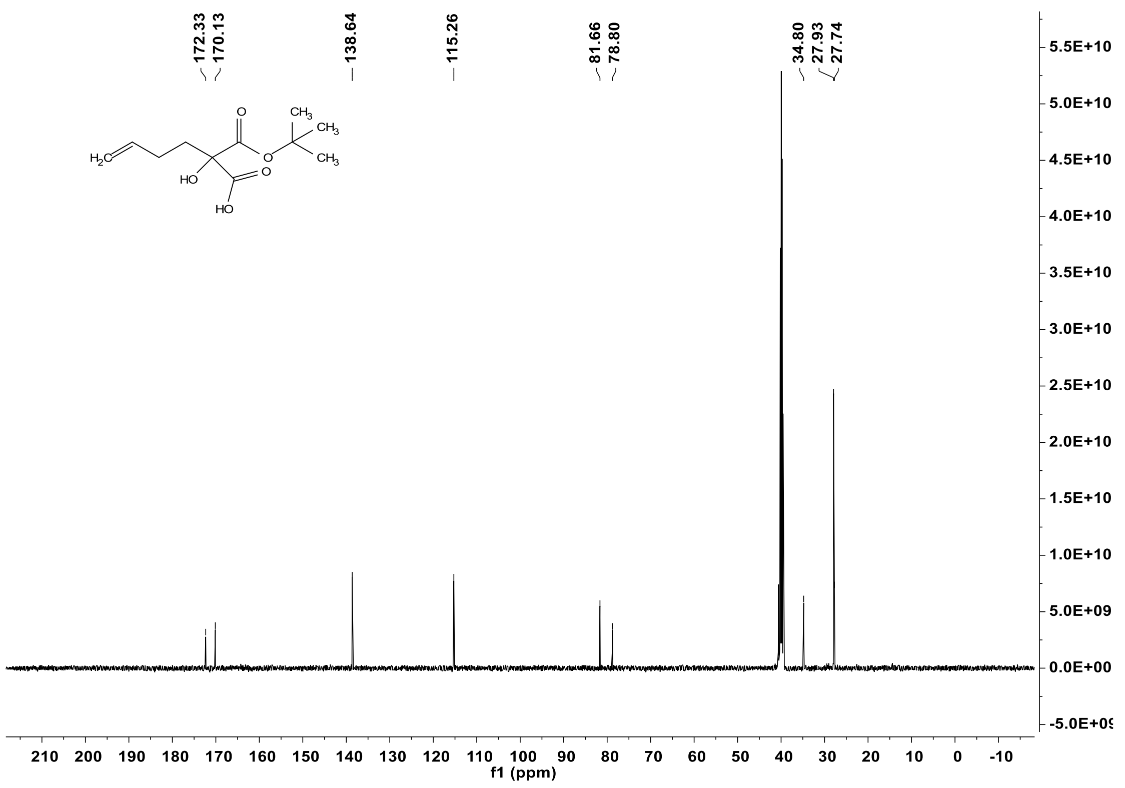

Supplementary Figure 78. ^{13}C NMR spectra of compound **6d**.

2-(*tert*-butoxycarbonyl)-2-hydroxy-5-phenylpentanoic acid (6e)

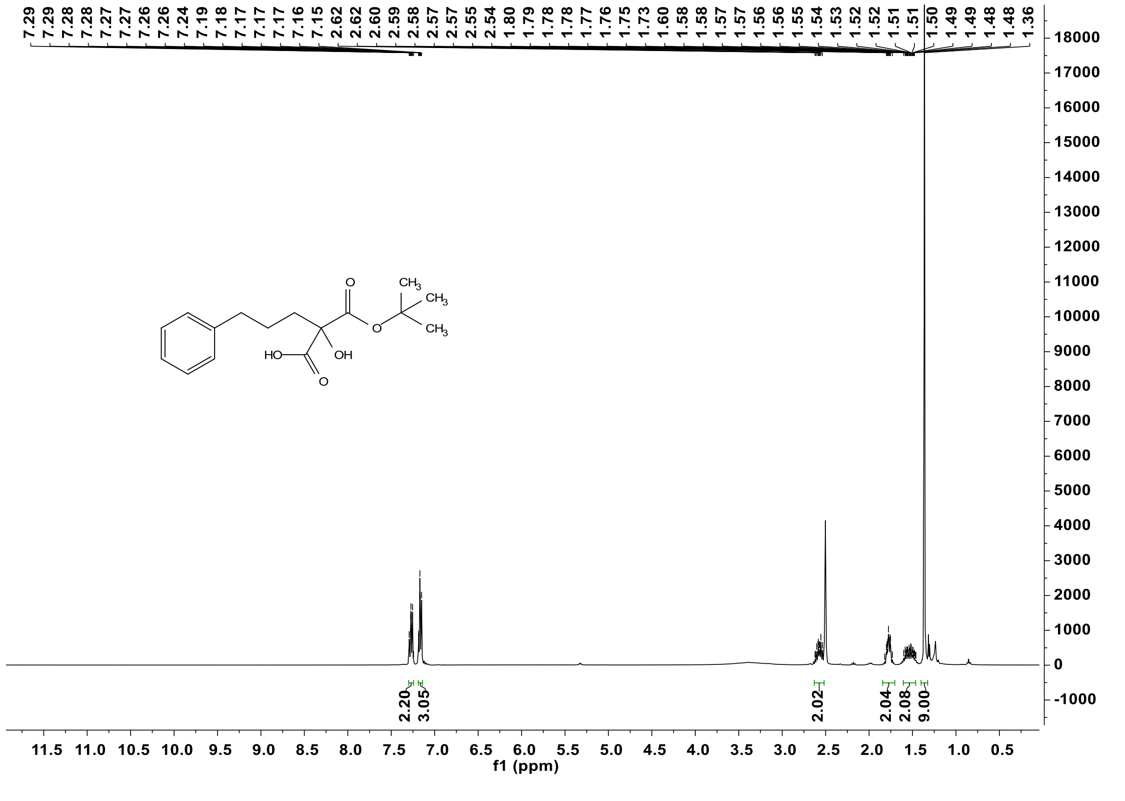

Supplementary Figure 79. ^{1}H NMR spectra of compound **6e**.

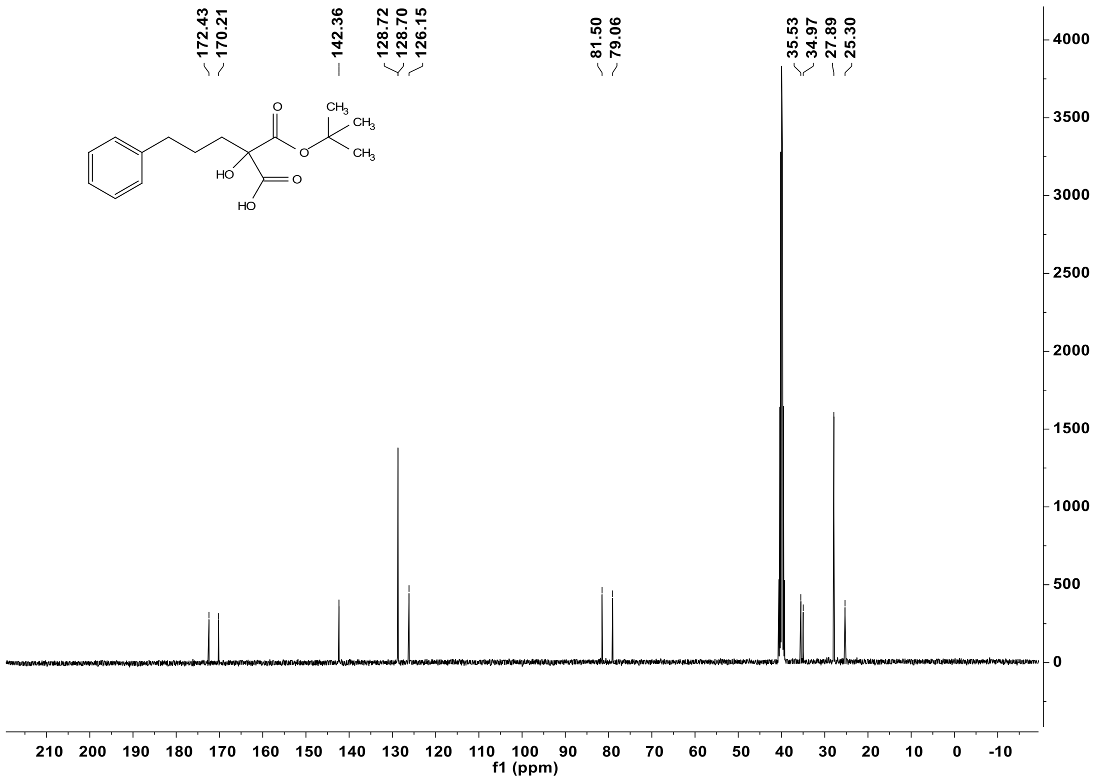

Supplementary Figure 80. ^{13}C NMR spectra of compound **6e**.

3-(((3*S*,8*S*,9*S*,10*R*,13*R*,14*S*,17*R*)-10,13-dimethyl-17-((*R*)-6-methylheptan-2-yl)-2,3,4,7,8,9,10,11,12,13,14,15,16,17-tetradecahydro-1*H*-cyclopenta[a]phenanthren-3-yl)oxy)-2-hydroxy-2-methyl-3-oxopropanoic acid (6f)

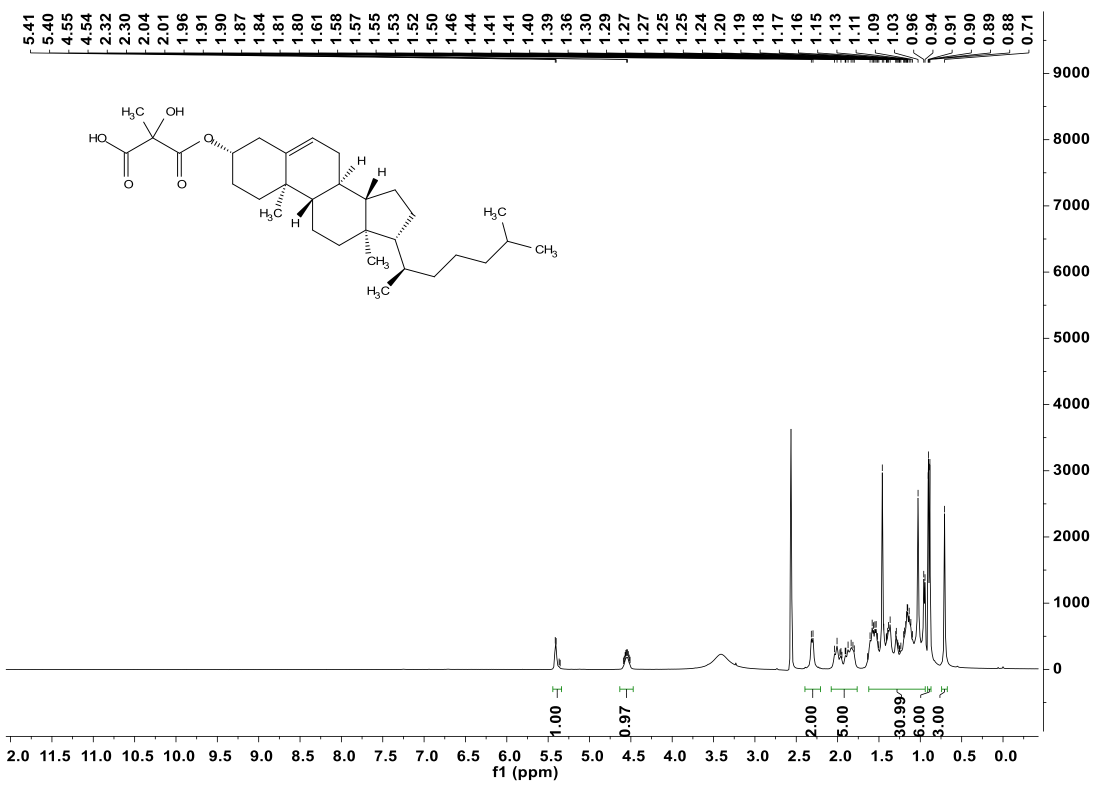

Supplementary Figure 81. ^{1}H NMR spectra of compound **6f**.

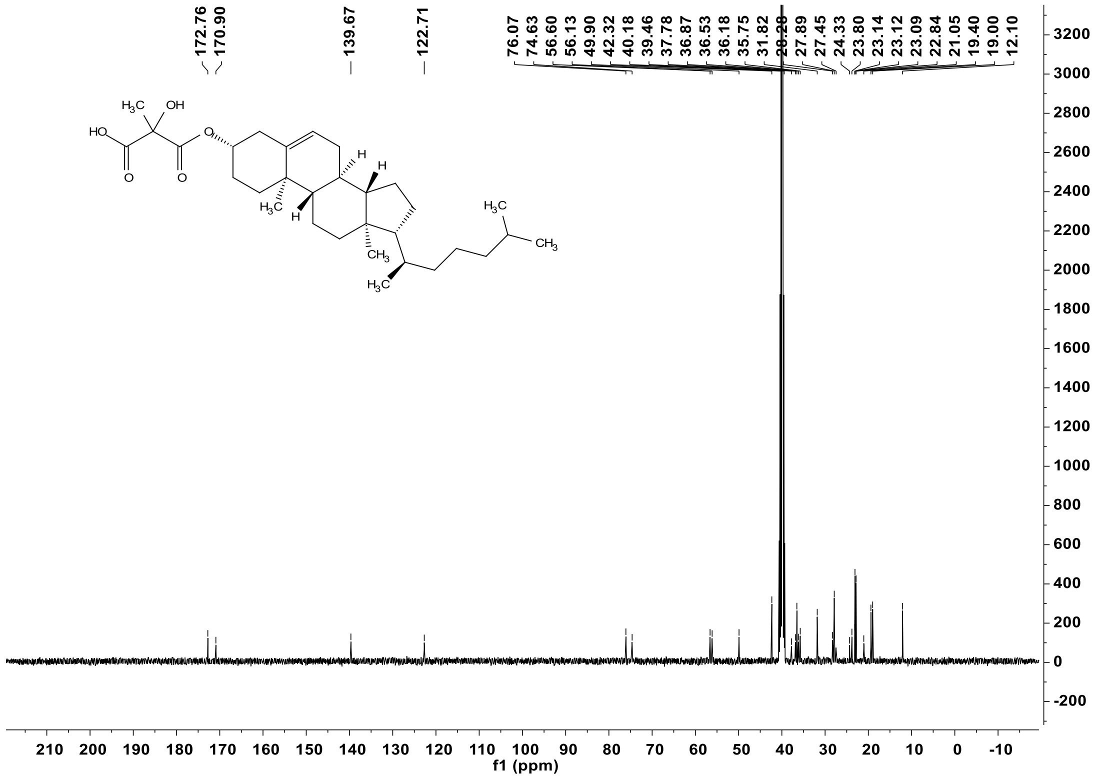

Supplementary Figure 82. ^{13}C NMR spectra of compound **6f**.

methyl 2-hydroxy-2-methyl-3-(methyl(phenyl)amino)-3-oxopropanoate (6g)

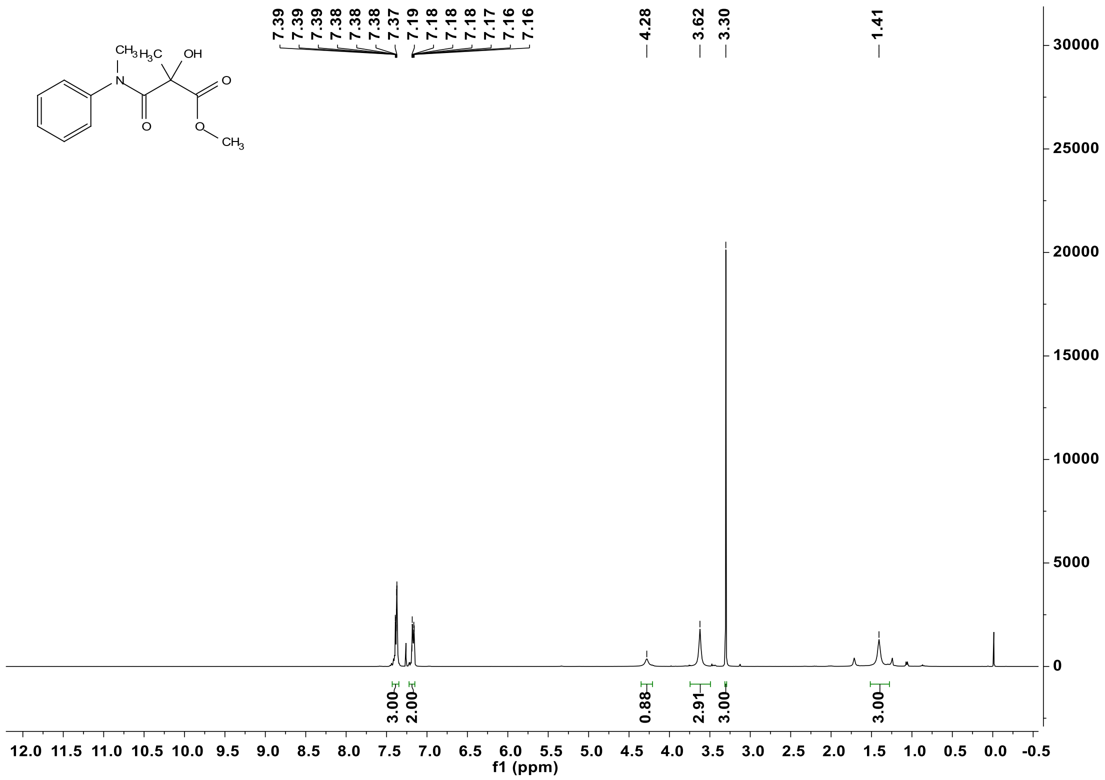

Supplementary Figure 83. ^{1}H NMR spectra of compound **6g**.

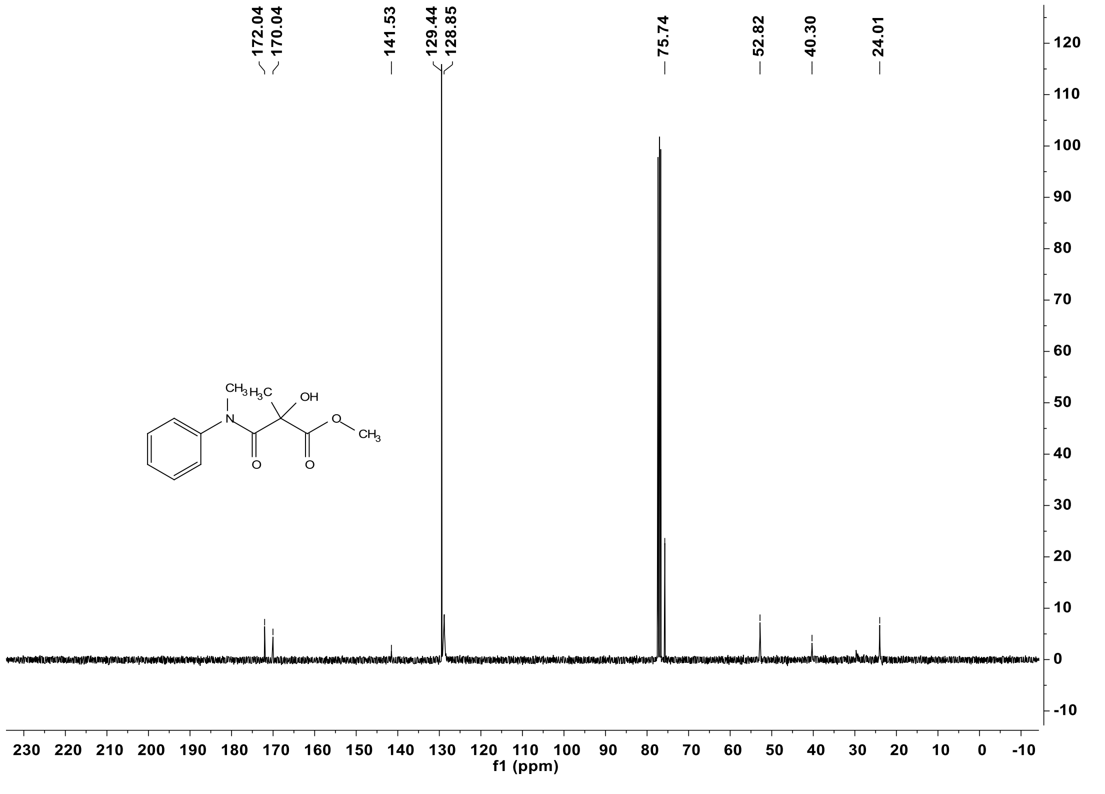

Supplementary Figure 84. ^{13}C NMR spectra of compound **6g**.

methyl 3-(ethyl(phenyl)amino)-2-hydroxy-2-methyl-3-oxopropanoate (6h)

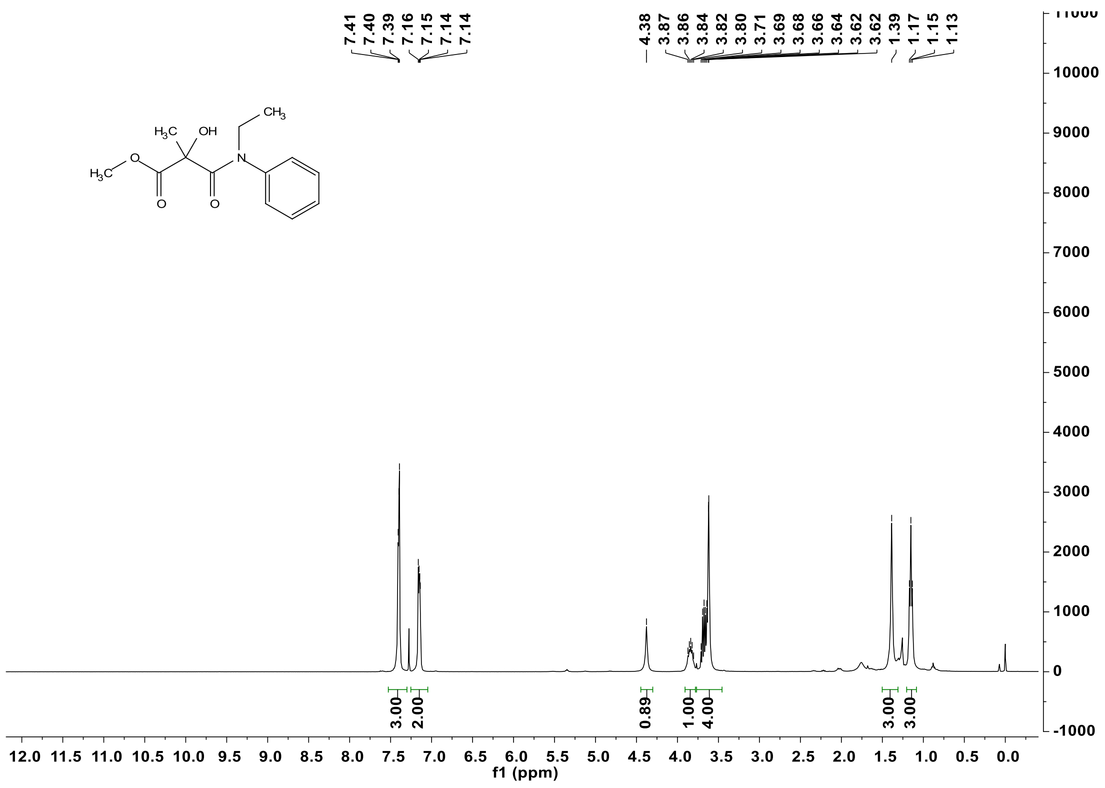

Supplementary Figure 85. ^{1}H NMR spectra of compound **6h**.

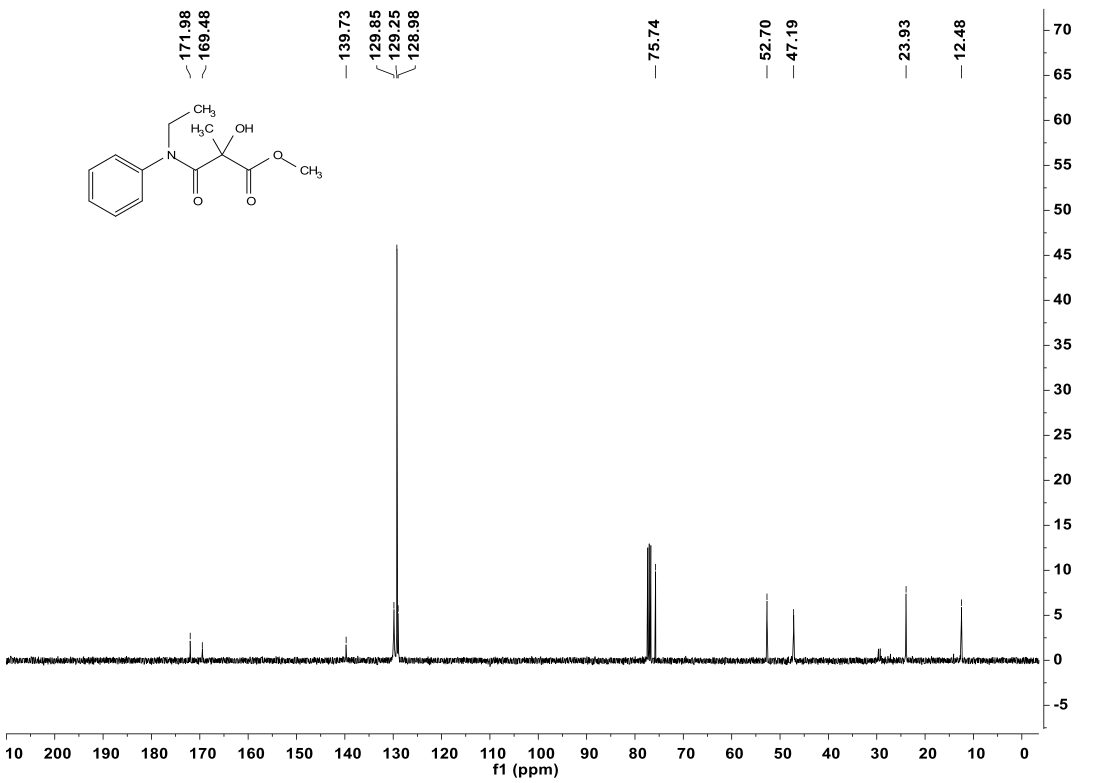

Supplementary Figure 86. ^{13}C NMR spectra of compound **6h**.

methyl 3-(butyl(phenyl)amino)-2-hydroxy-2-methyl-3-oxopropanoate (6i)

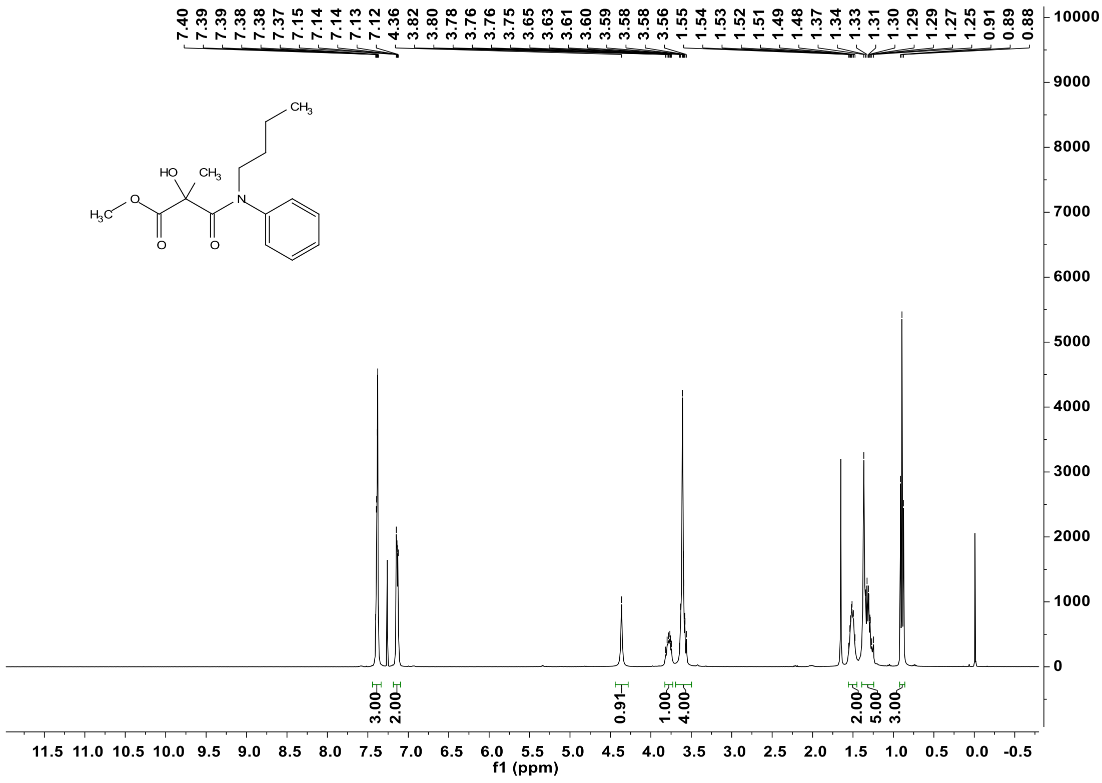

Supplementary Figure 87. ^{1}H NMR spectra of compound **6i**.

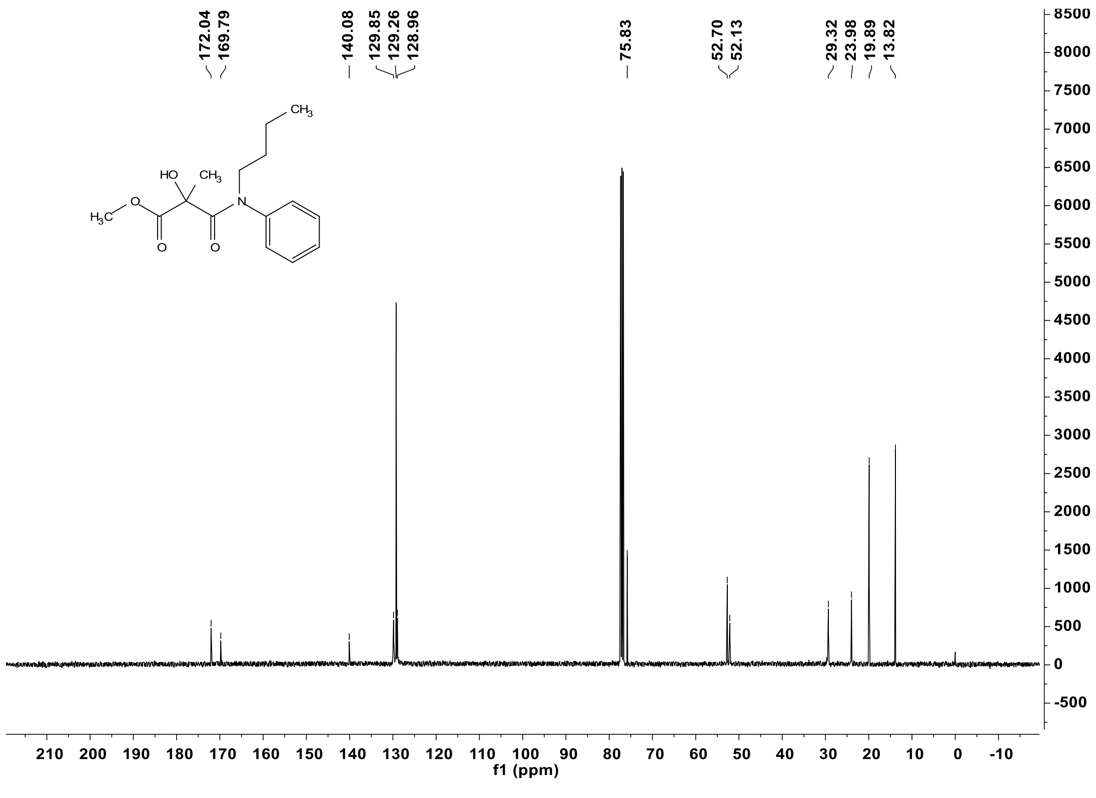

Supplementary Figure 88. ^{13}C NMR spectra of compound **6i**.

methyl 2-hydroxy-3-(isopropyl(phenyl)amino)-2-methyl-3-oxopropanoate (6j)

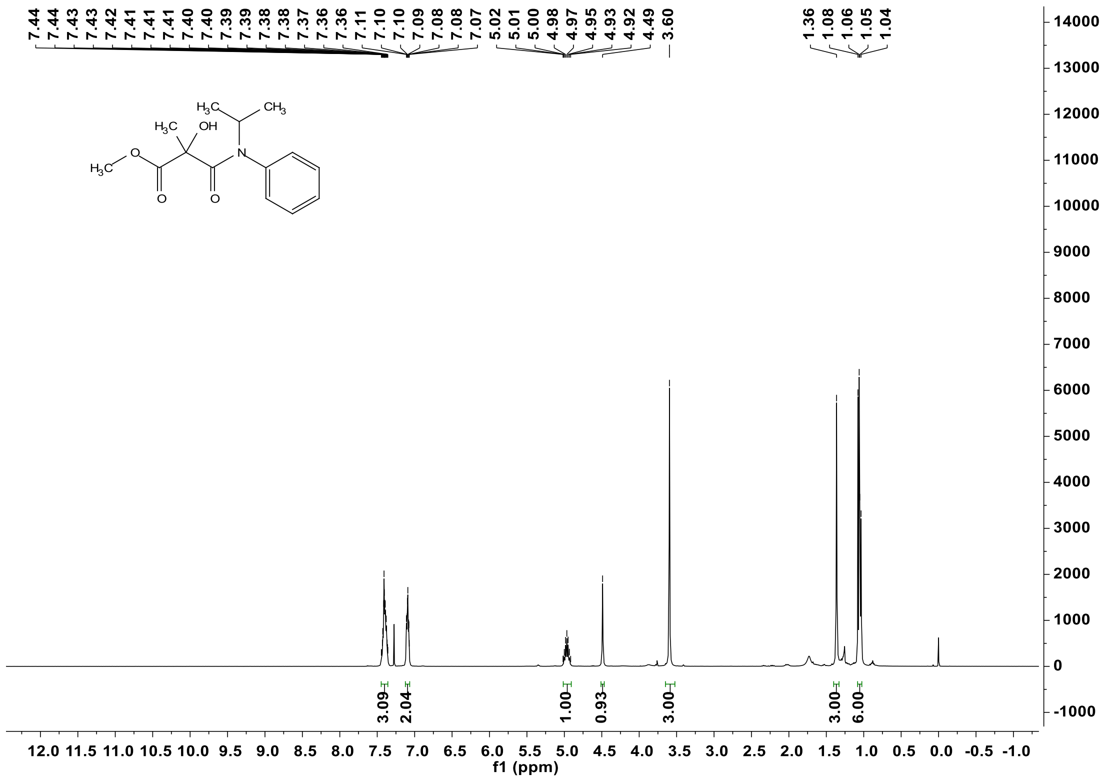

Supplementary Figure 89. ^{1}H NMR spectra of compound **6j**.

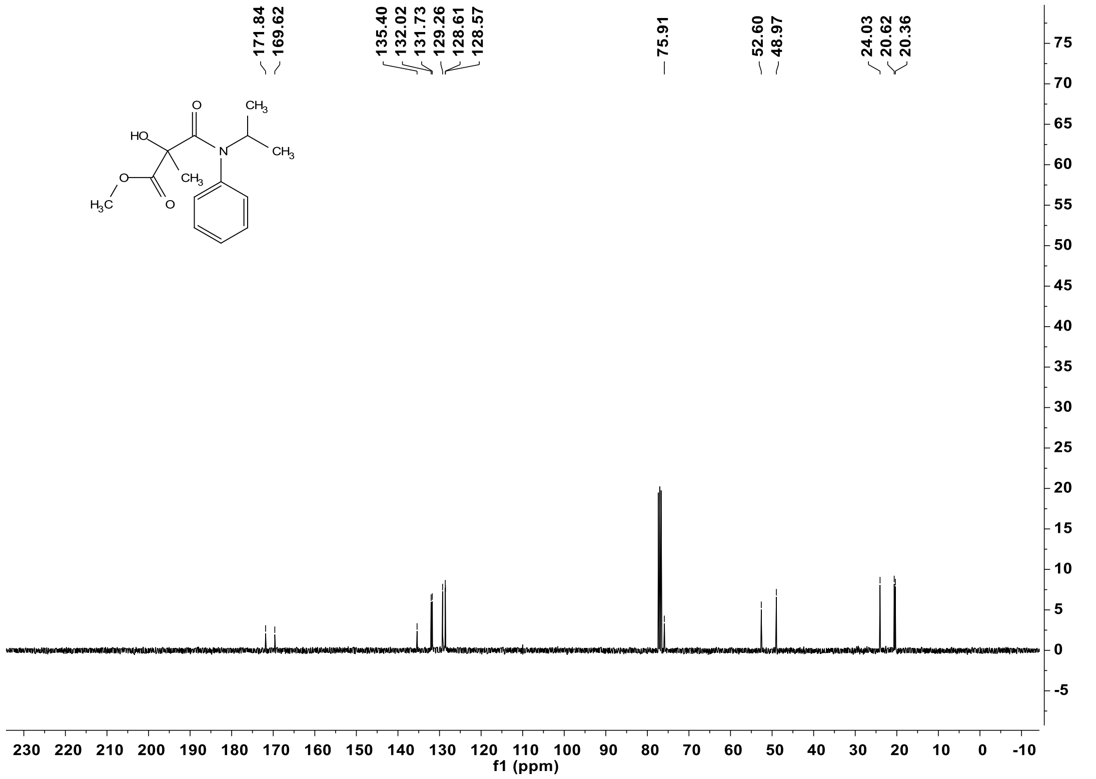

Supplementary Figure 90. ^{13}C NMR spectra of compound **6j**.

2-(benzoyloxy)-2-methyl-3-oxo-3-(piperidin-1-yl)propanoic acid (6k)

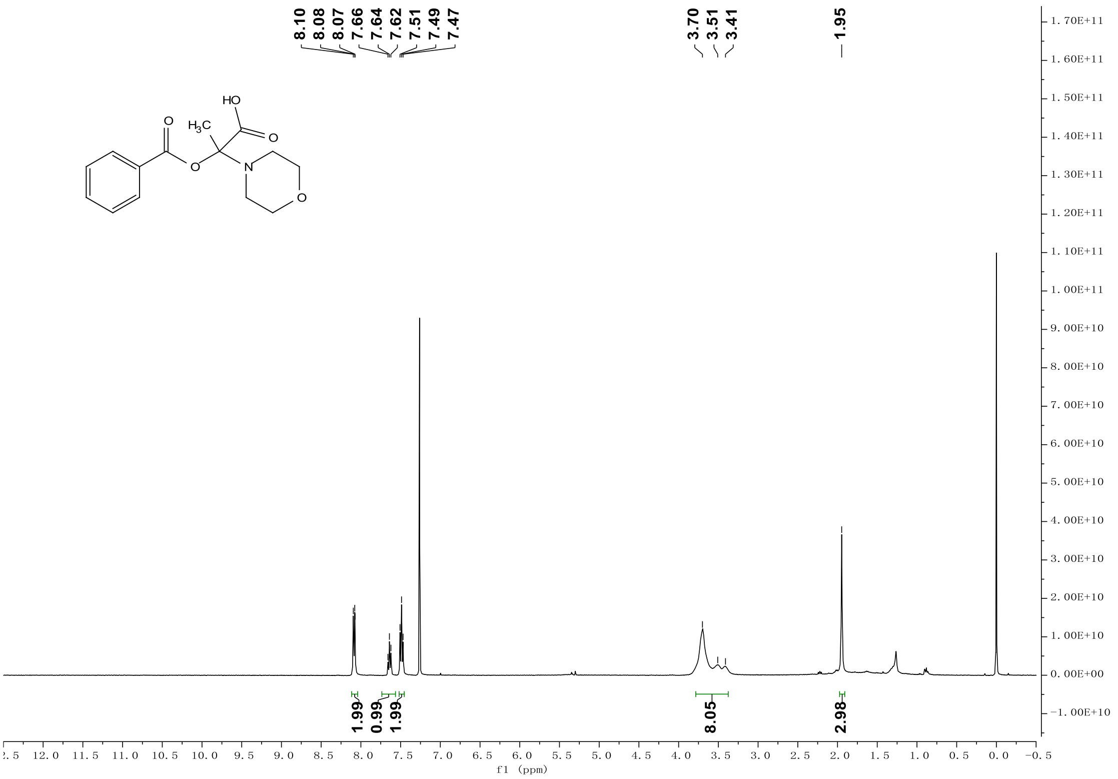

Supplementary Figure 91. ^{1}H NMR spectra of compound **6k**.

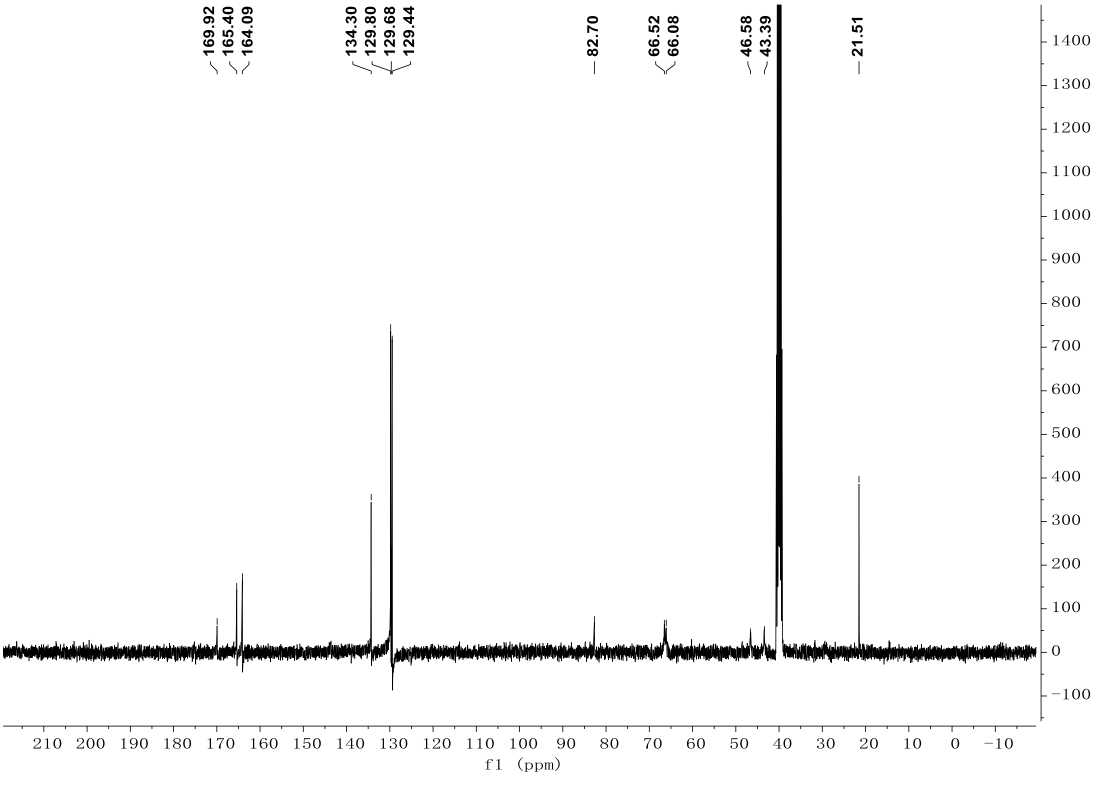

Supplementary Figure 92. ^{13}C NMR spectra of compound **6k**.

methyl 2-([1,1'-biphenyl]-4-yl)-2-hydroxyacetate (8a)

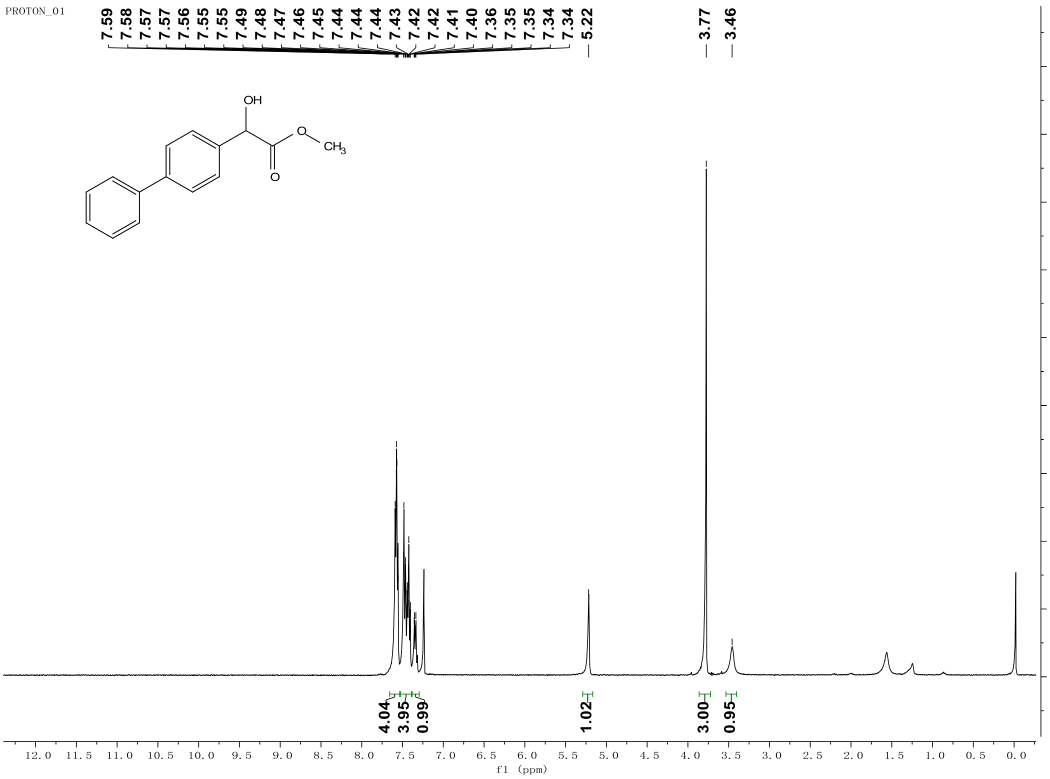

Supplementary Figure 93. ^{1}H NMR spectra of compound **8a**.

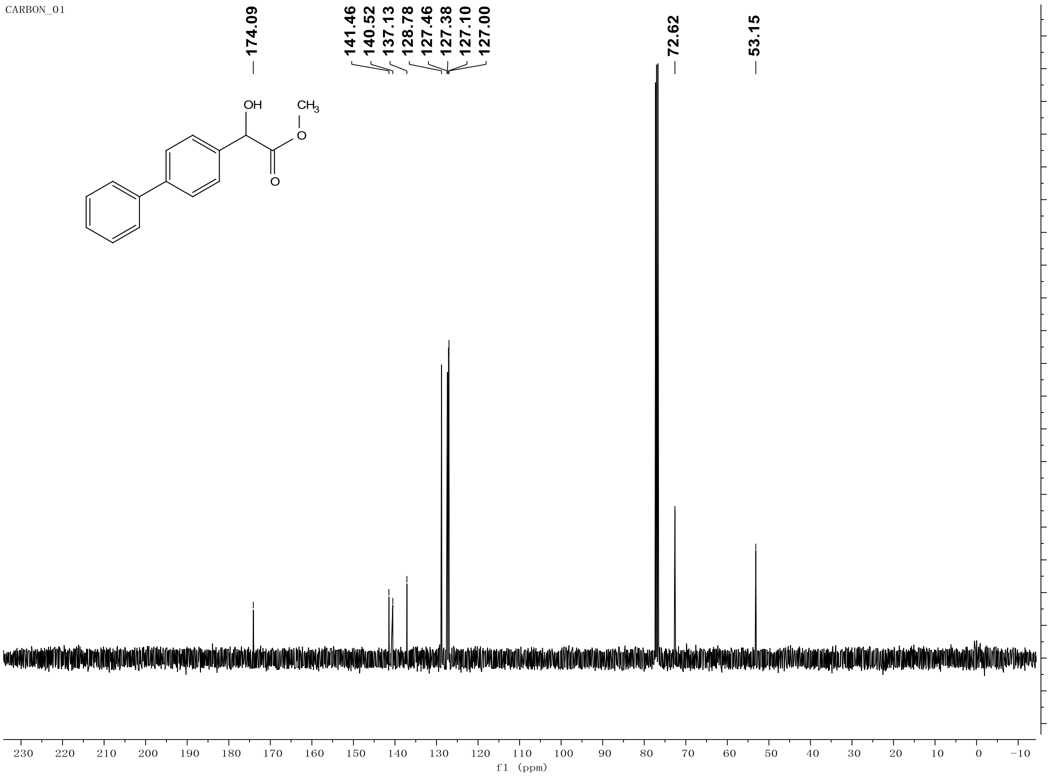

Supplementary Figure 94. ^{13}C NMR spectra of compound **8a**.

methyl 2-hydroxy-2-(4'-methyl-[1,1'-biphenyl]-4-yl)acetate (8b)

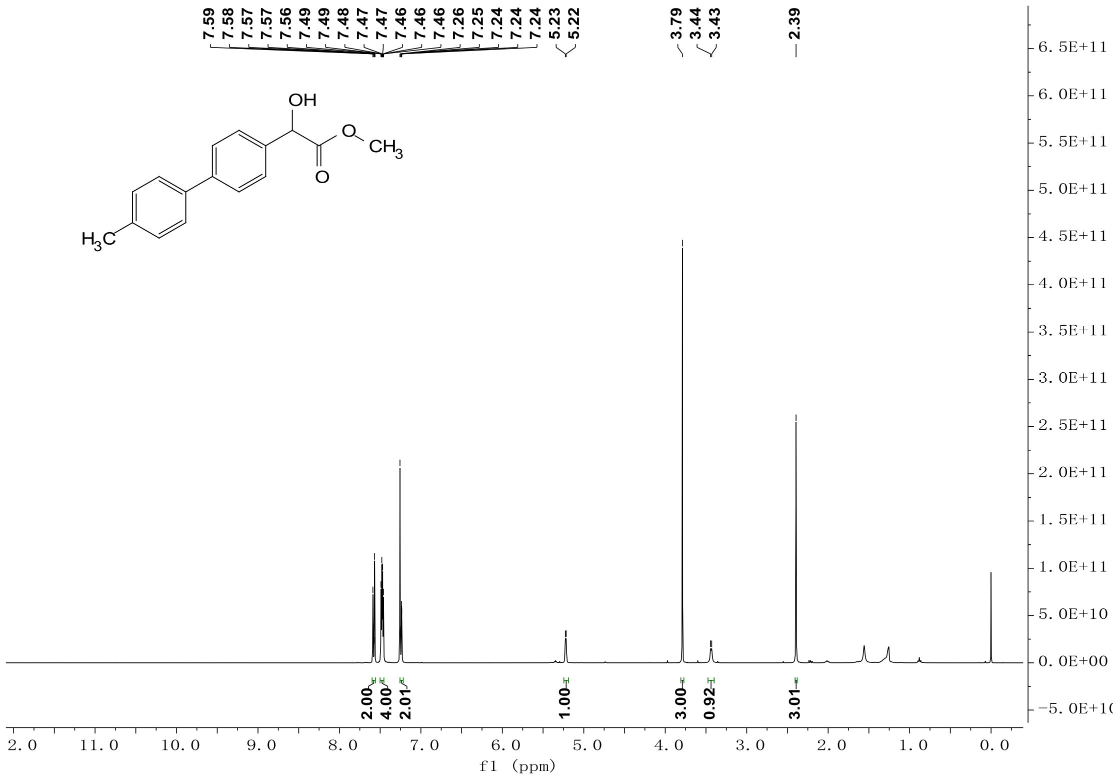

Supplementary Figure 95. ^{1}H NMR spectra of compound **8b**.

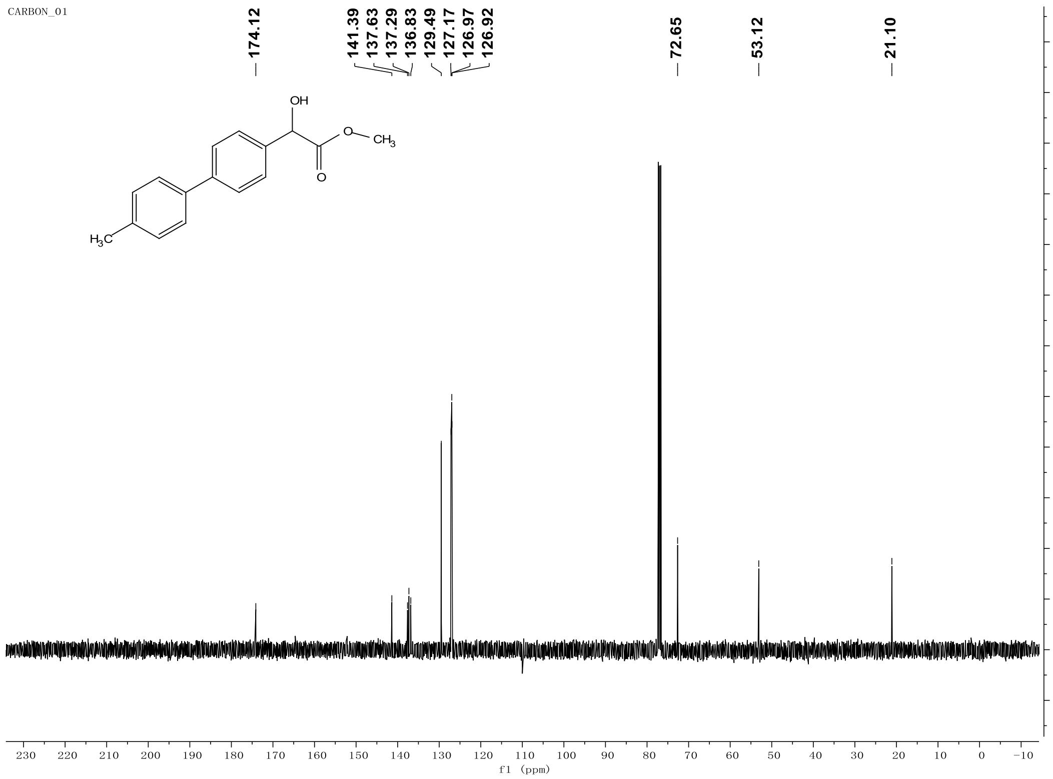

Supplementary Figure 96. ^{13}C NMR spectra of compound **8b**.

methyl 2-hydroxy-2-(4'-(methylthio)-[1,1'-biphenyl]-4-yl)acetate (8c)

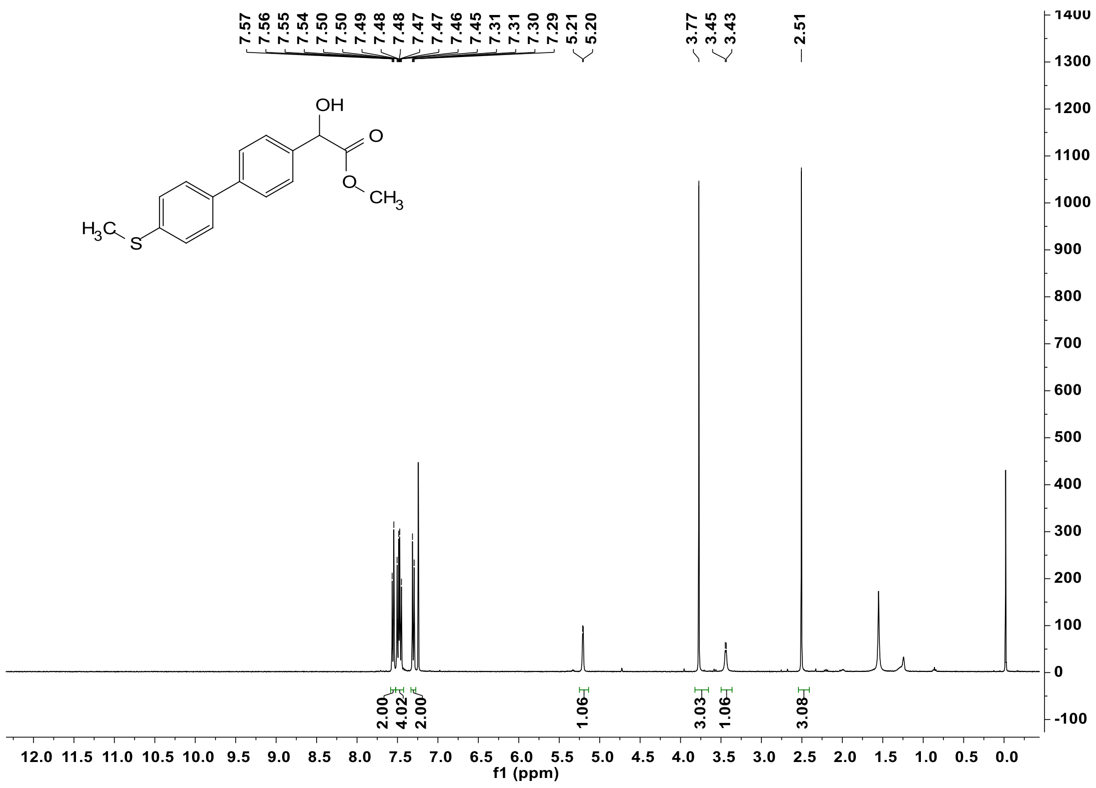

Supplementary Figure 97. ^{1}H NMR spectra of compound **8c**.

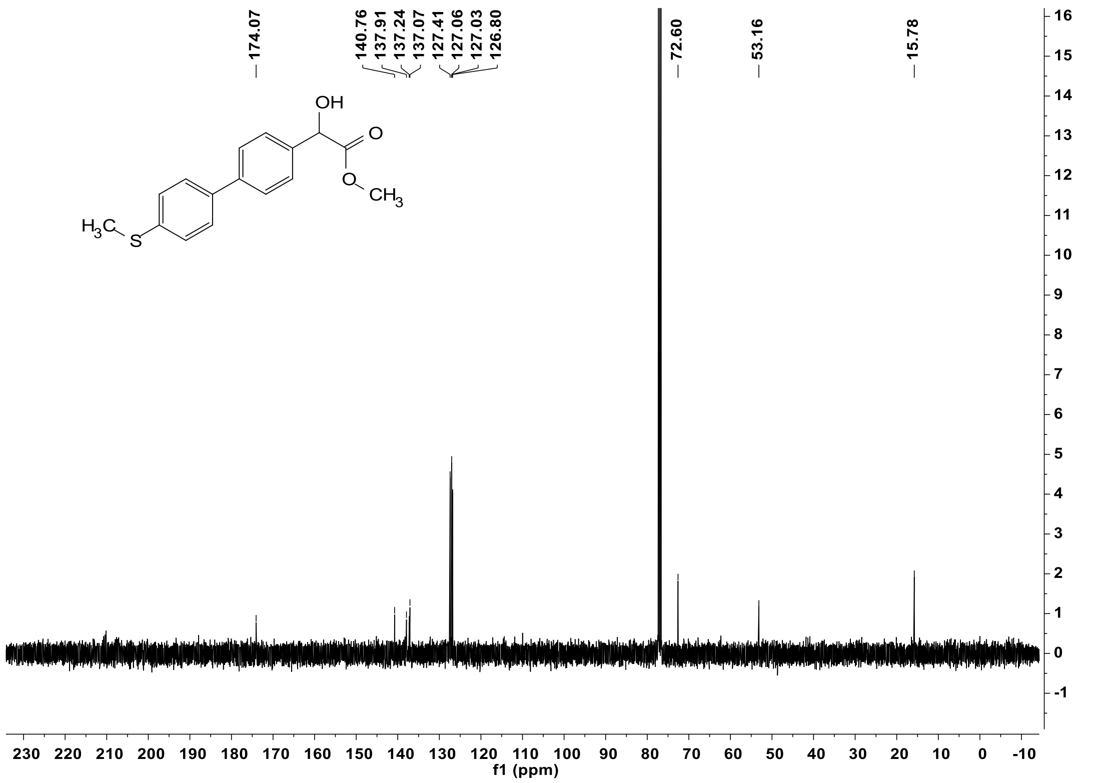

Supplementary Figure 98. ^{13}C NMR spectra of compound **8c**.

methyl 2-hydroxy-2-(4-(thiophen-3-yl)phenyl)acetate (8d)

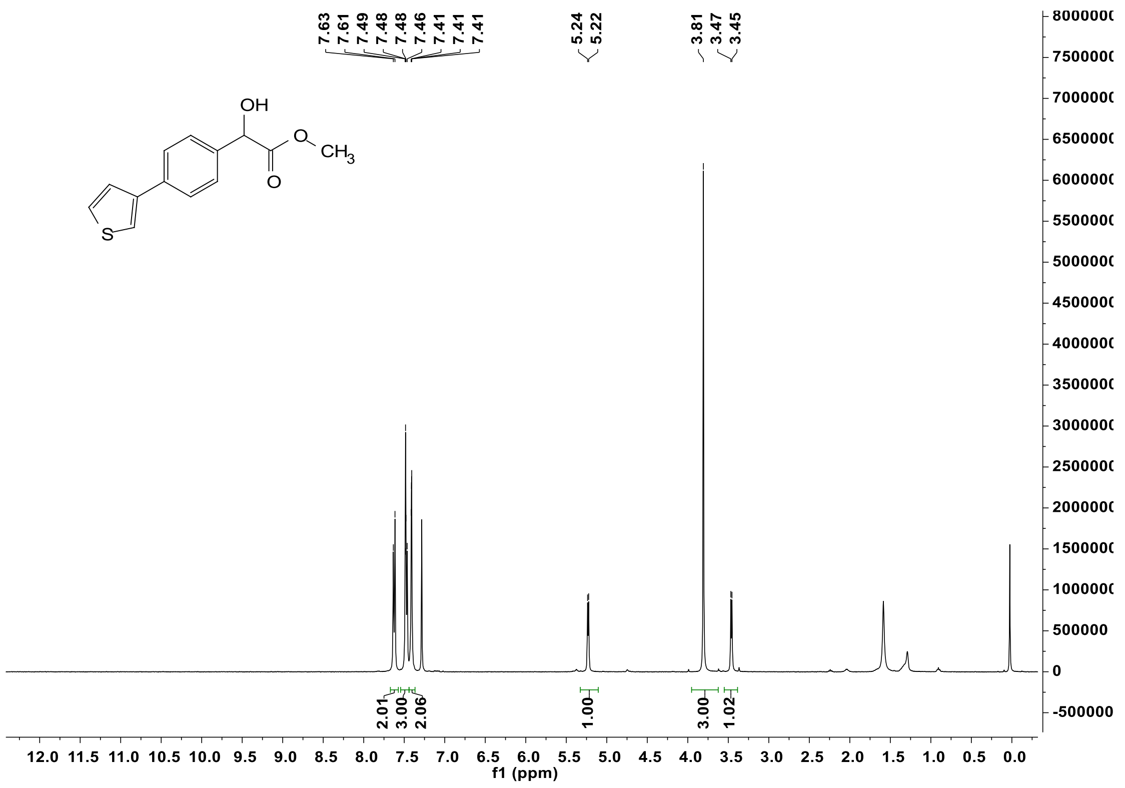

Supplementary Figure 99. ^{1}H NMR spectra of compound **8d**.

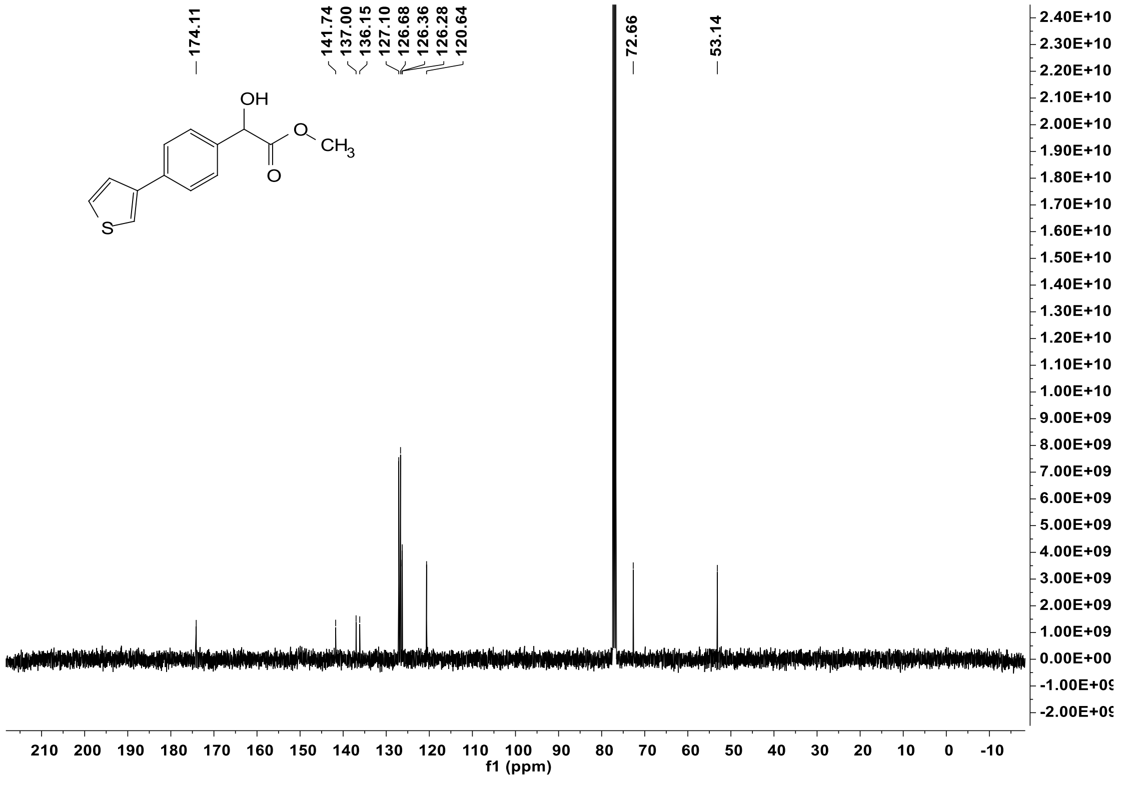

Supplementary Figure 100. ^{13}C NMR spectra of compound **8d**.

methyl 2-hydroxy-2-(naphthalen-2-yl)acetate (8e)

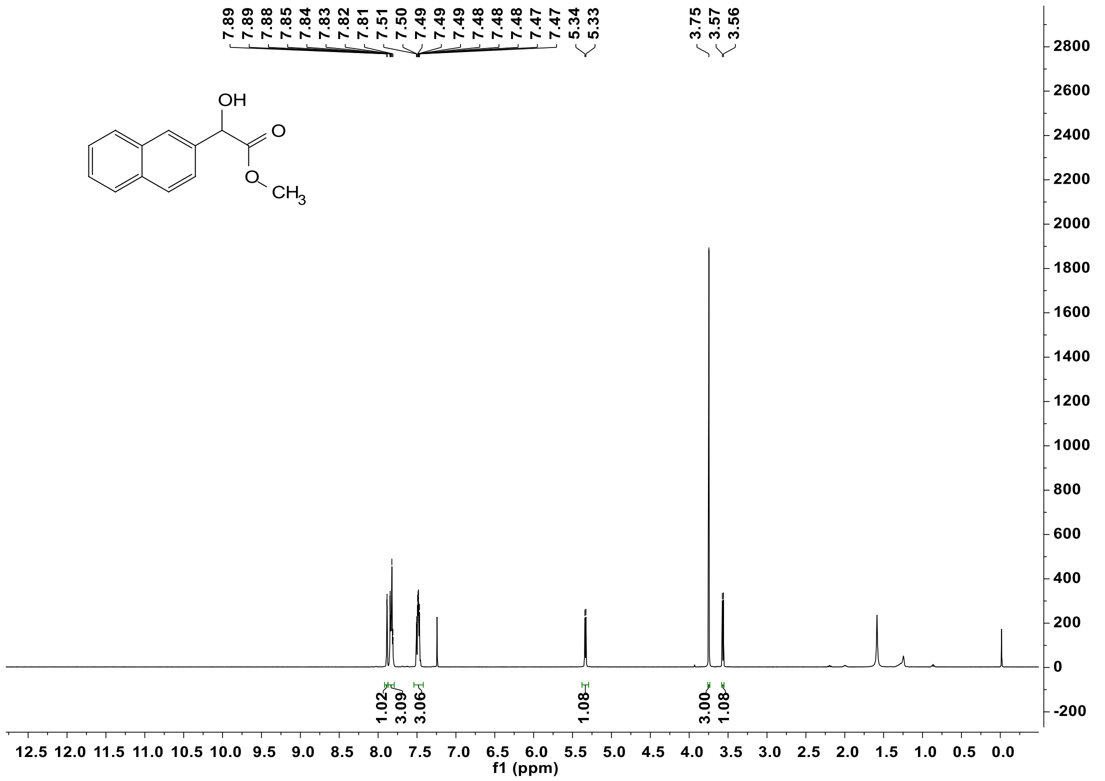

Supplementary Figure 101. ^{1}H NMR spectra of compound **8e**.

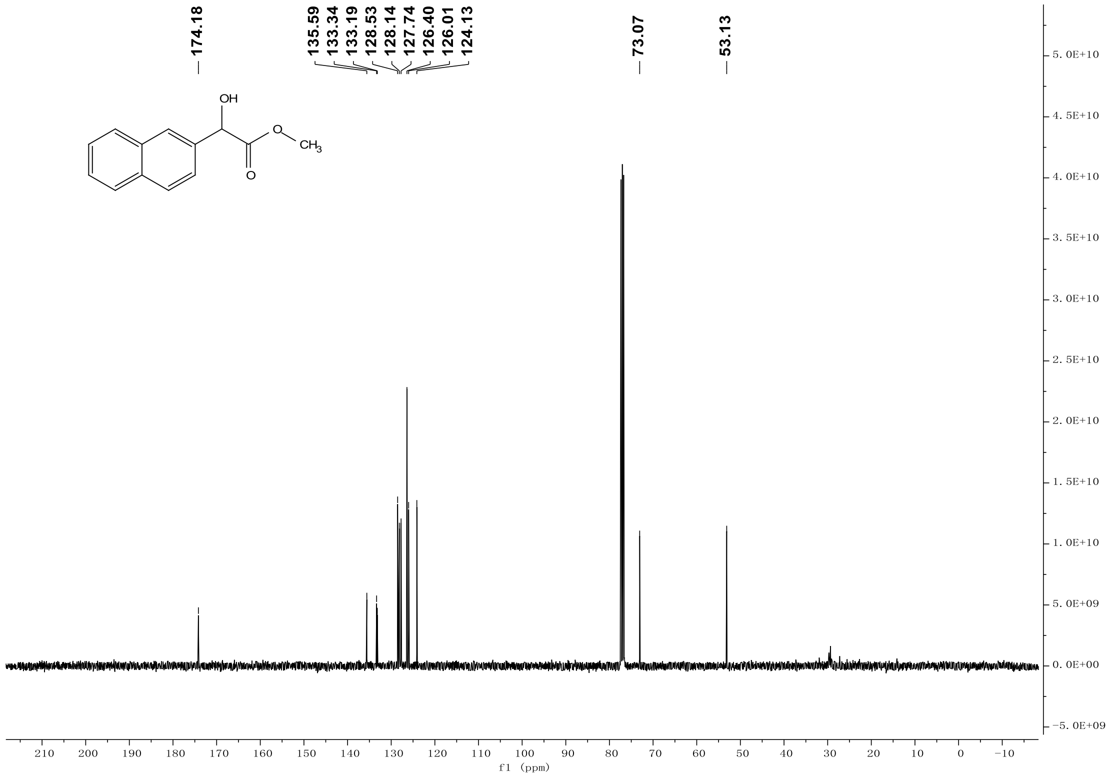

Supplementary Figure 102. ^{13}C NMR spectra of compound **8e**.

methyl 2-hydroxy-2-(phenanthren-9-yl)acetate (8f)

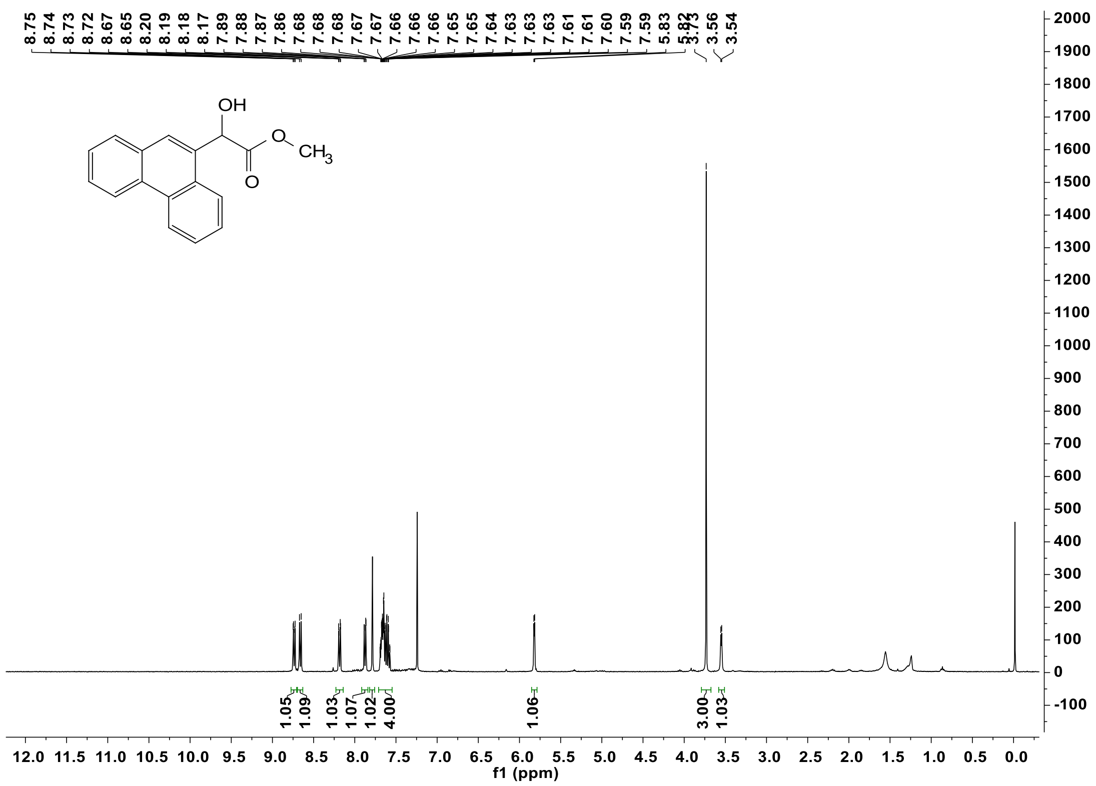

Supplementary Figure 103. ^{1}H NMR spectra of compound **8f**.

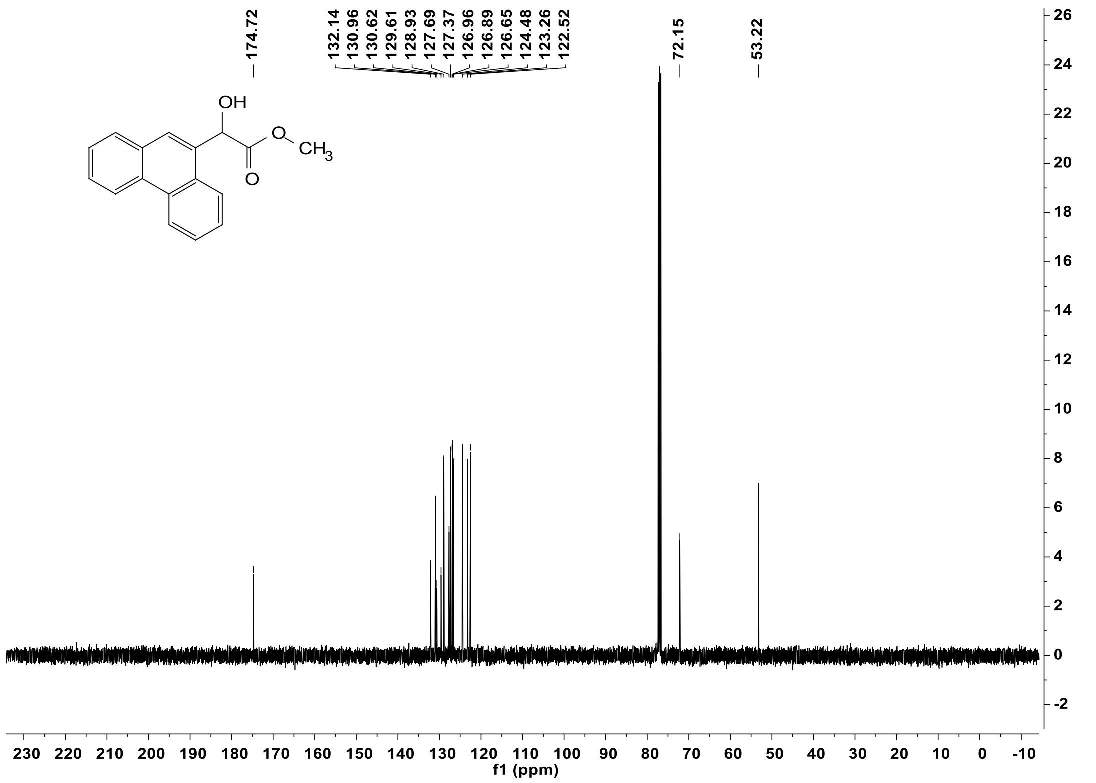

Supplementary Figure 104. ^{13}C NMR spectra of compound **8f**.

methyl 2-([1,1'-biphenyl]-4-yl)-2-hydroxypropanoate (2a-Me)

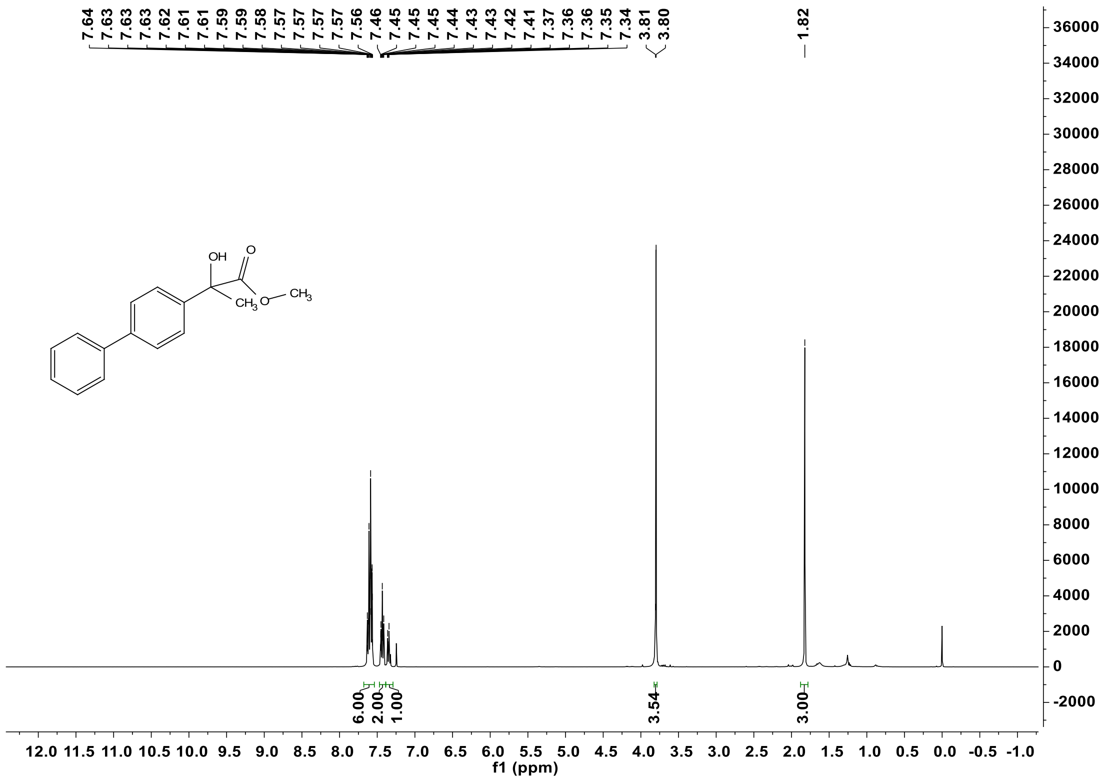

Supplementary Figure 105. ^{13}C NMR spectra of compound **2a-Me**.

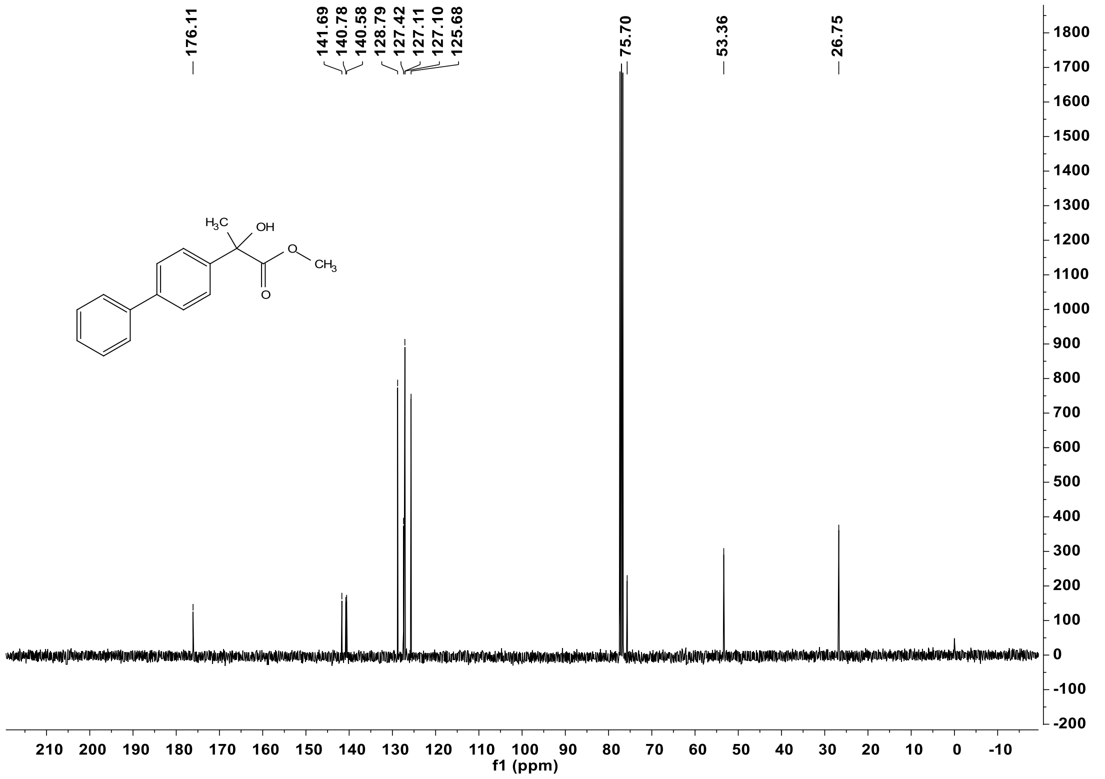

Supplementary Figure 106. ^{13}C NMR spectra of compound **2a-Me**.

dimethyl 2-([1,1'-biphenyl]-4-yl)-2-hydroxysuccinate (9)

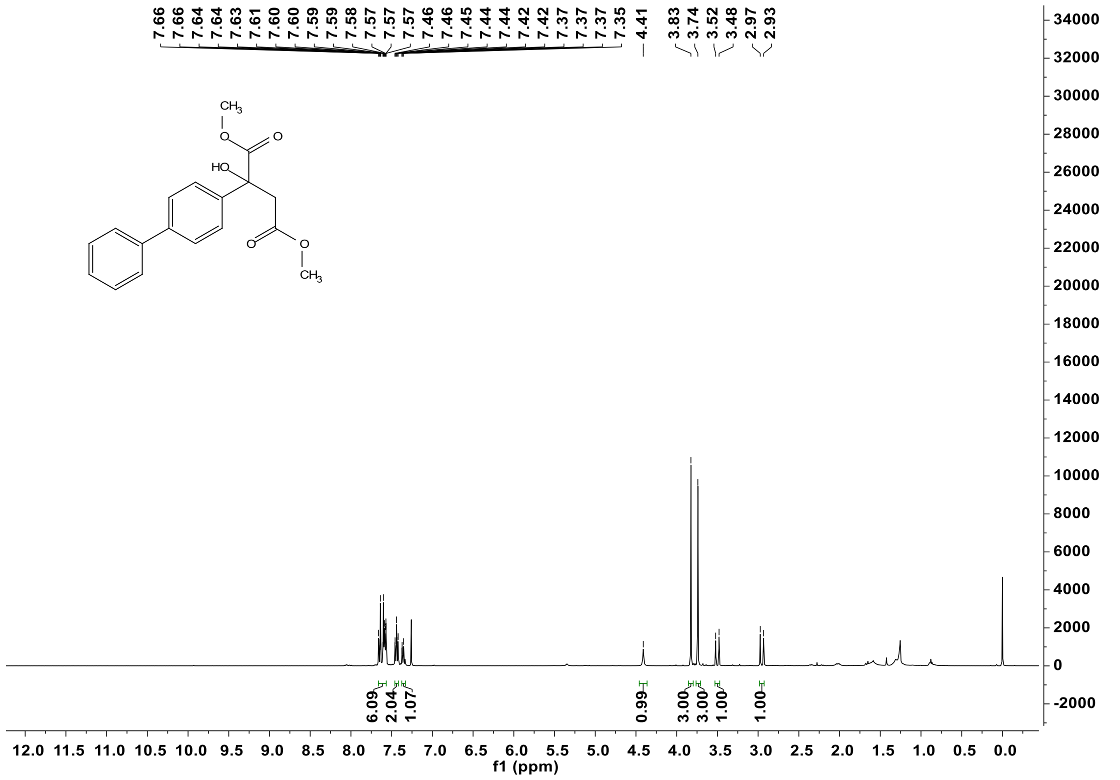

Supplementary Figure 107. ^{1}H NMR spectra of compound **9**.

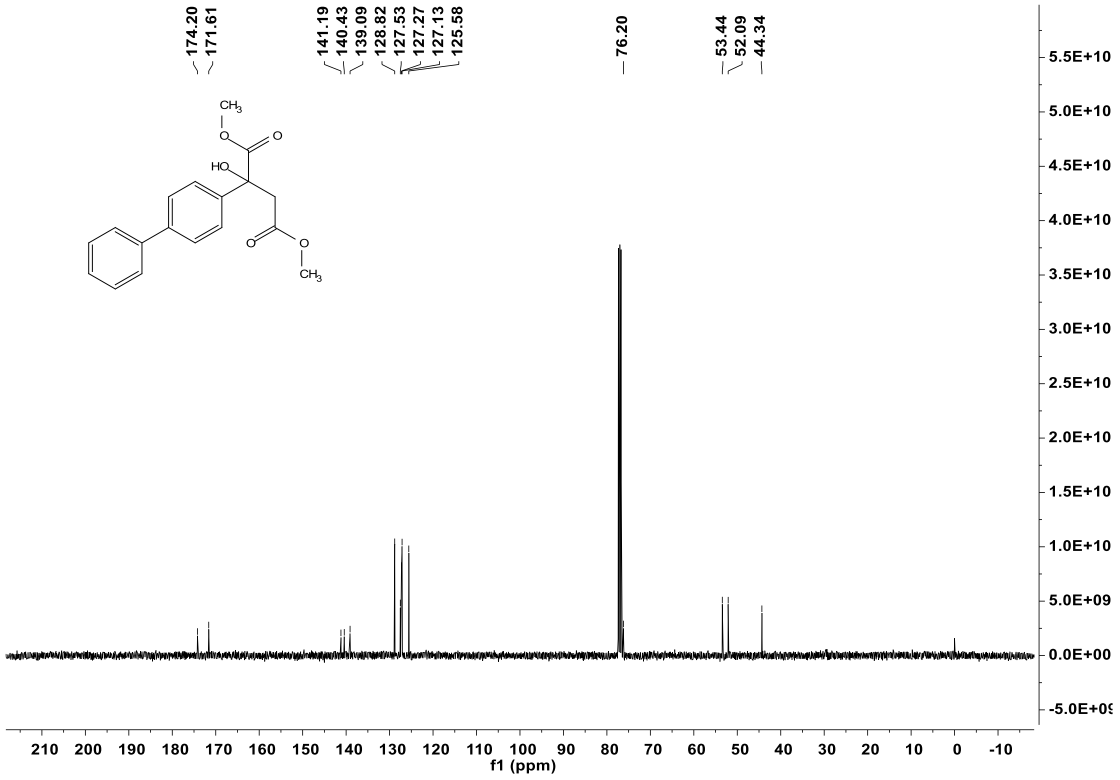

Supplementary Figure 108. ^{13}C NMR spectra of compound **9**.

1-cyclohexyl-1-phenylethane-1,2-diol (10)

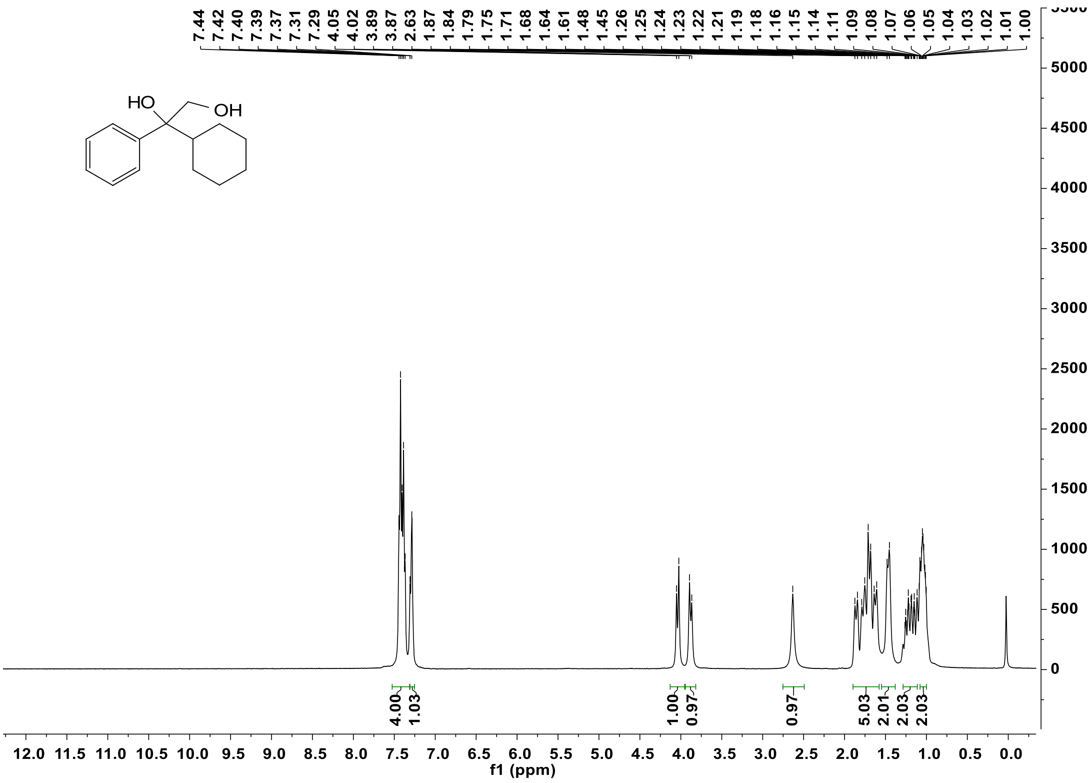

Supplementary Figure 109. ^{1}H NMR spectra of compound **10**.

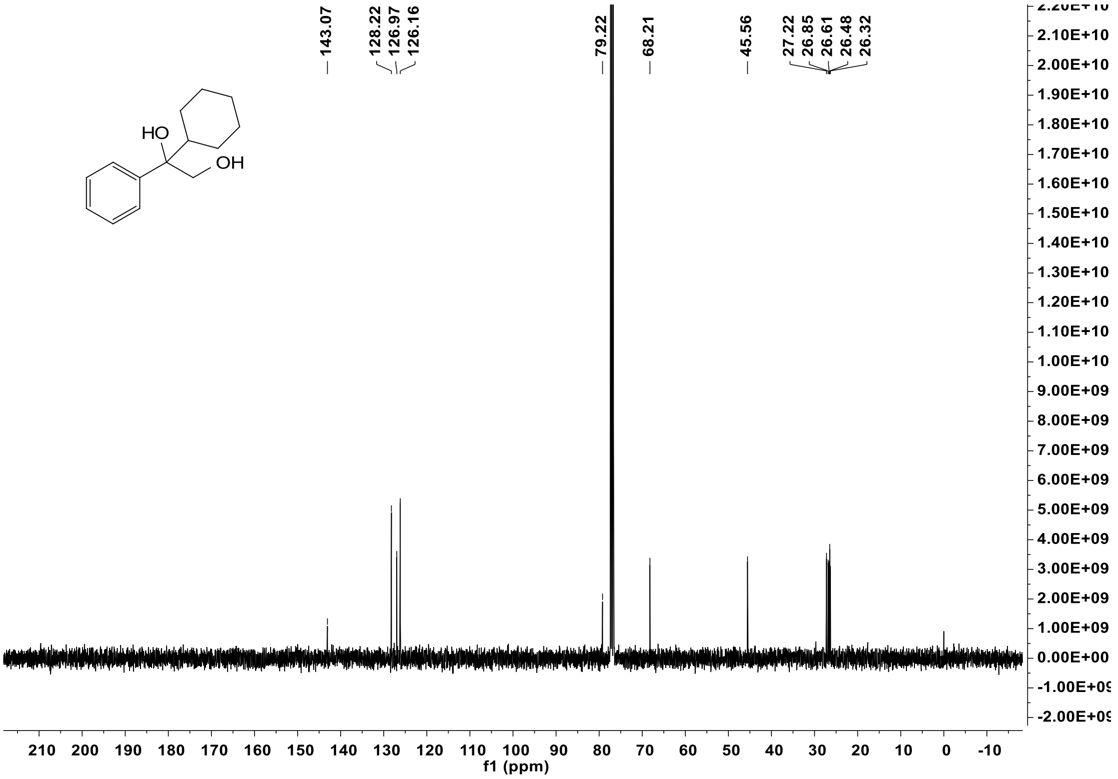

Supplementary Figure 110. ^{13}C NMR spectra of compound **10**.

4-cyclohexyl-4-phenyl-1,3-dioxolane-2-thione (11)

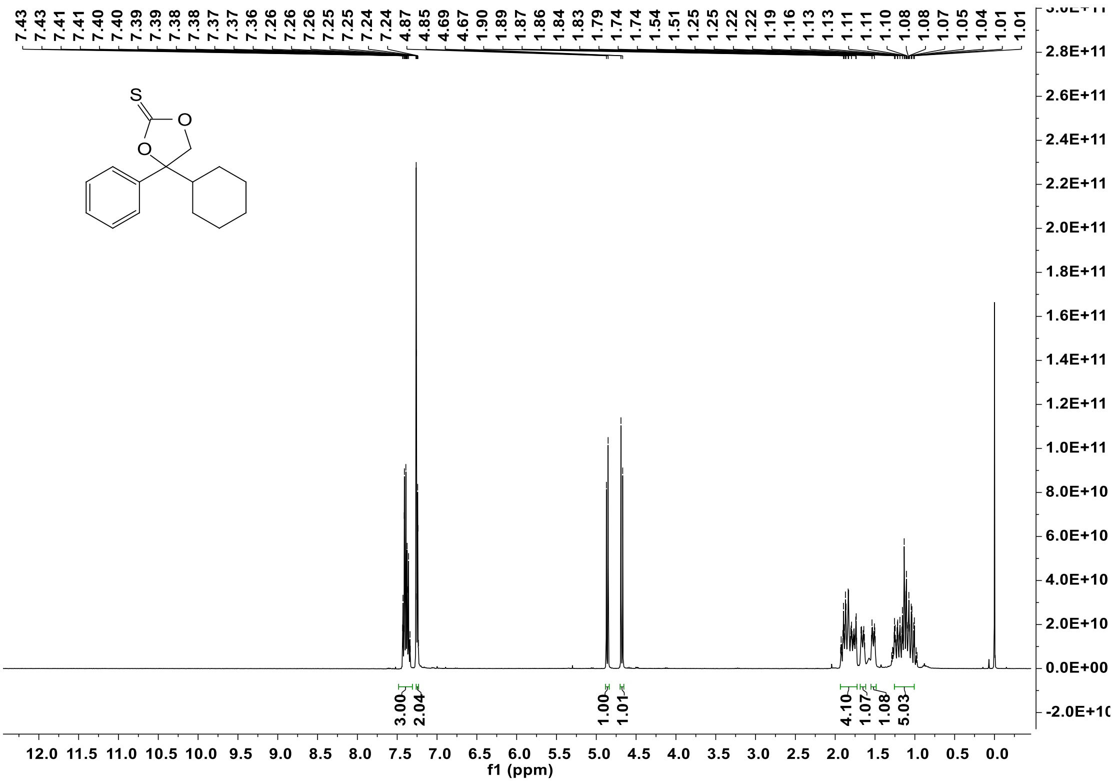

Supplementary Figure 111. ^{1}H NMR spectra of compound **11**.

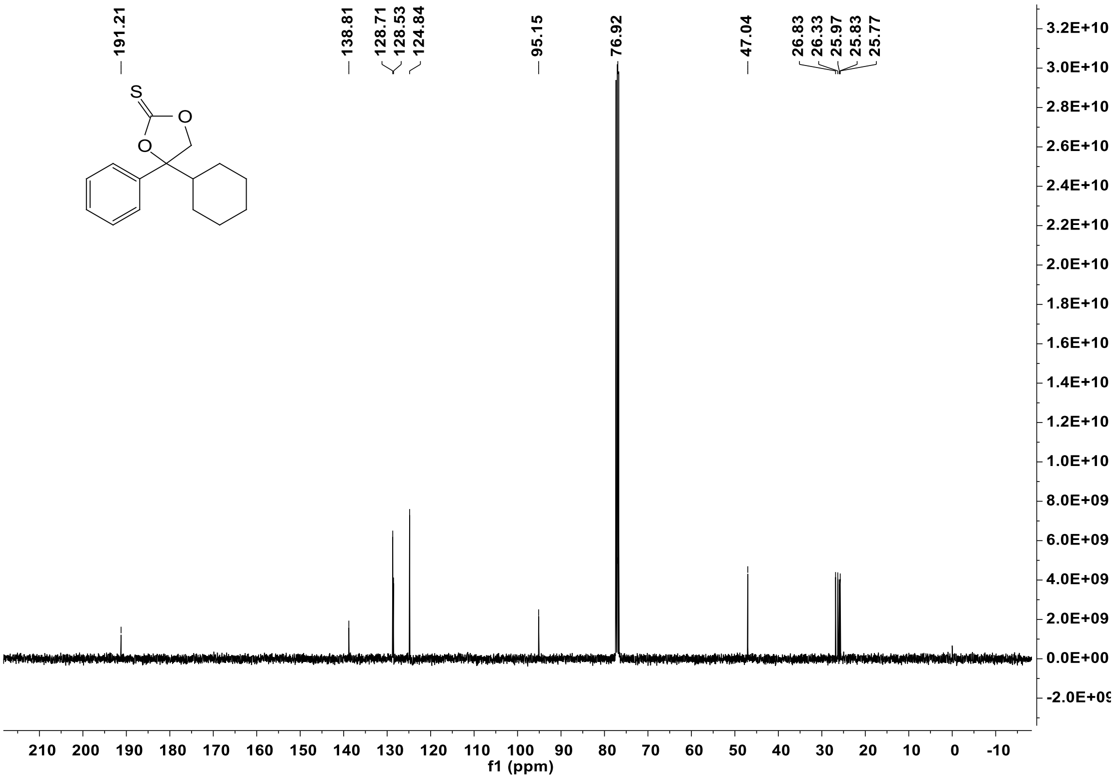

Supplementary Figure 112. ^{13}C NMR spectra of compound **11**.

methyl 2-cyclohexyl-2-fluoro-2-phenylacetate (12)

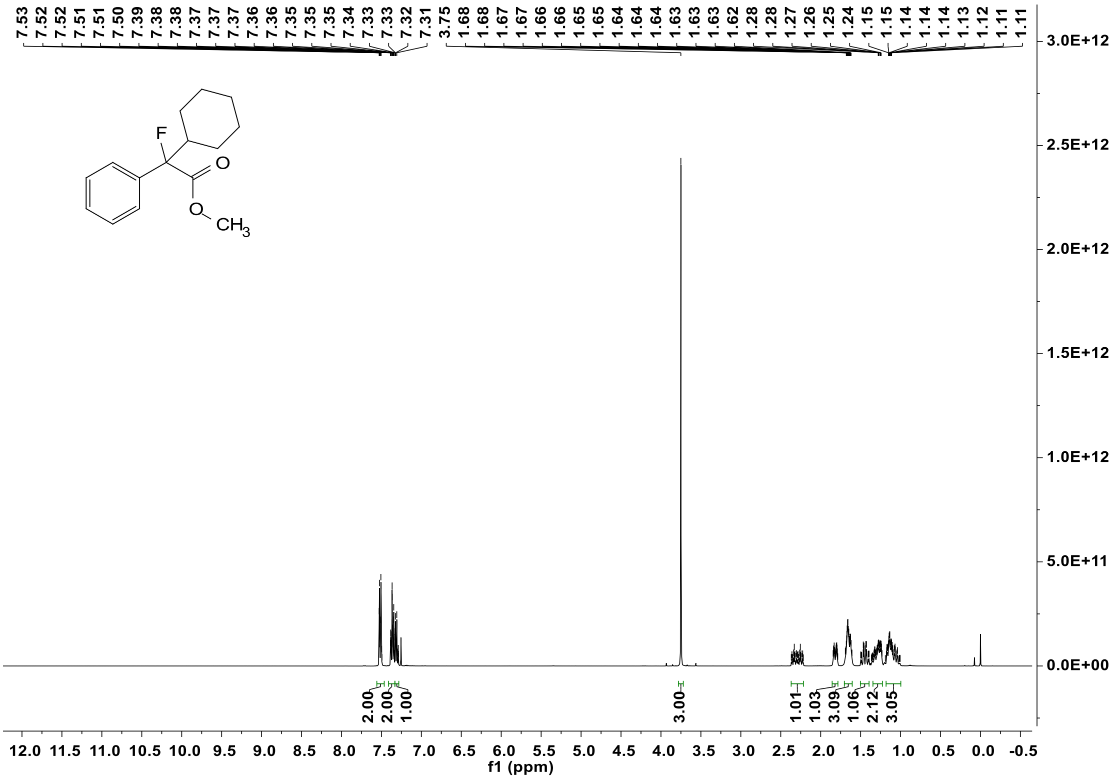

Supplementary Figure 113. ^{1}H NMR spectra of compound **12**.

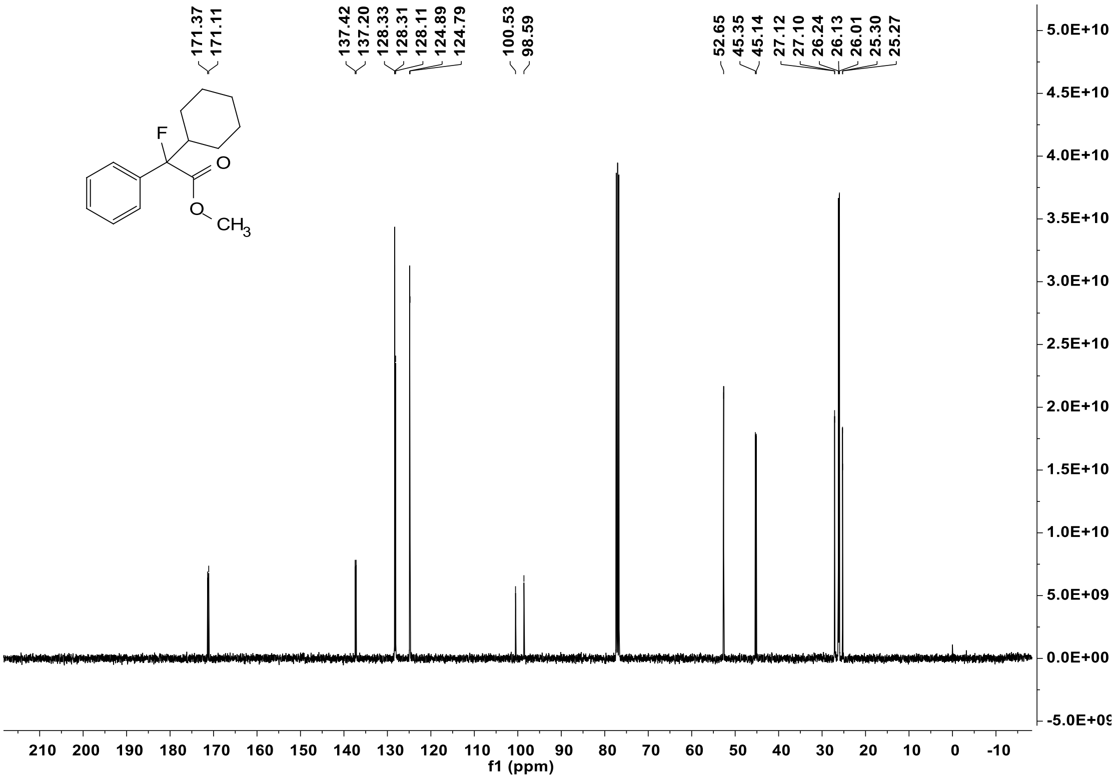

Supplementary Figure 114. ^{13}C NMR spectra of compound **12**.

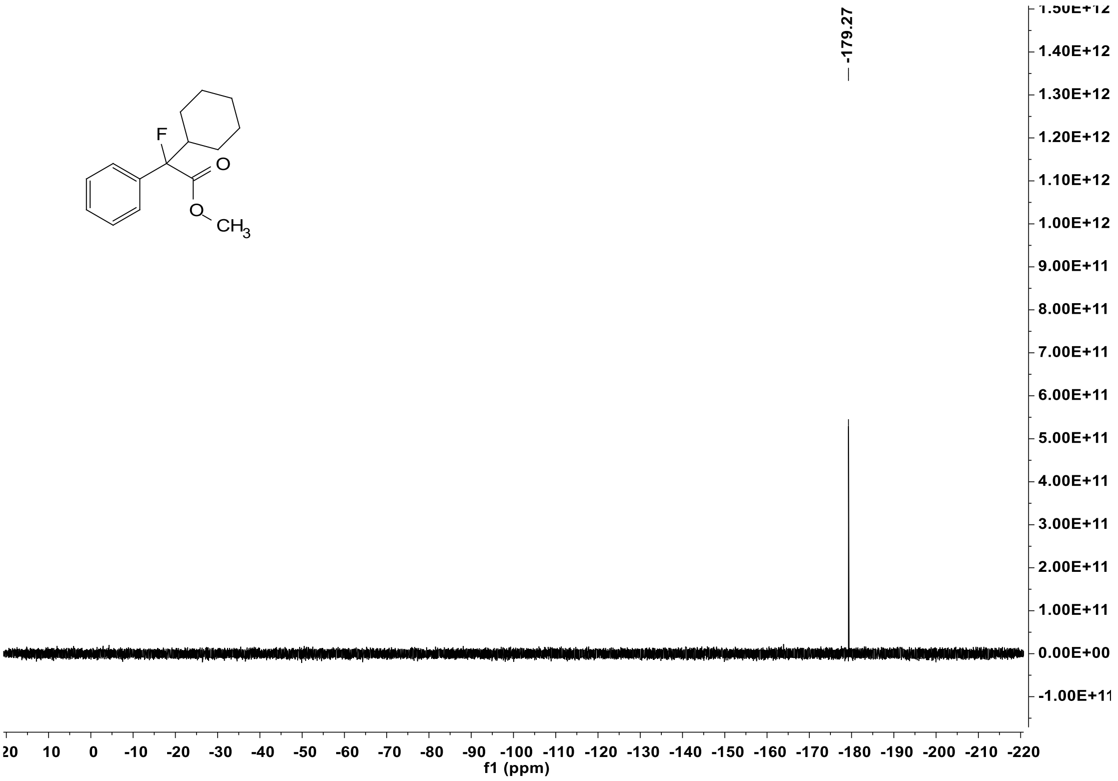

Supplementary Figure 115. ^{19}F NMR spectra of compound **12**.

2-(diethylamino)ethyl 2-cyclohexyl-2-hydroxy-2-phenylacetate (13)

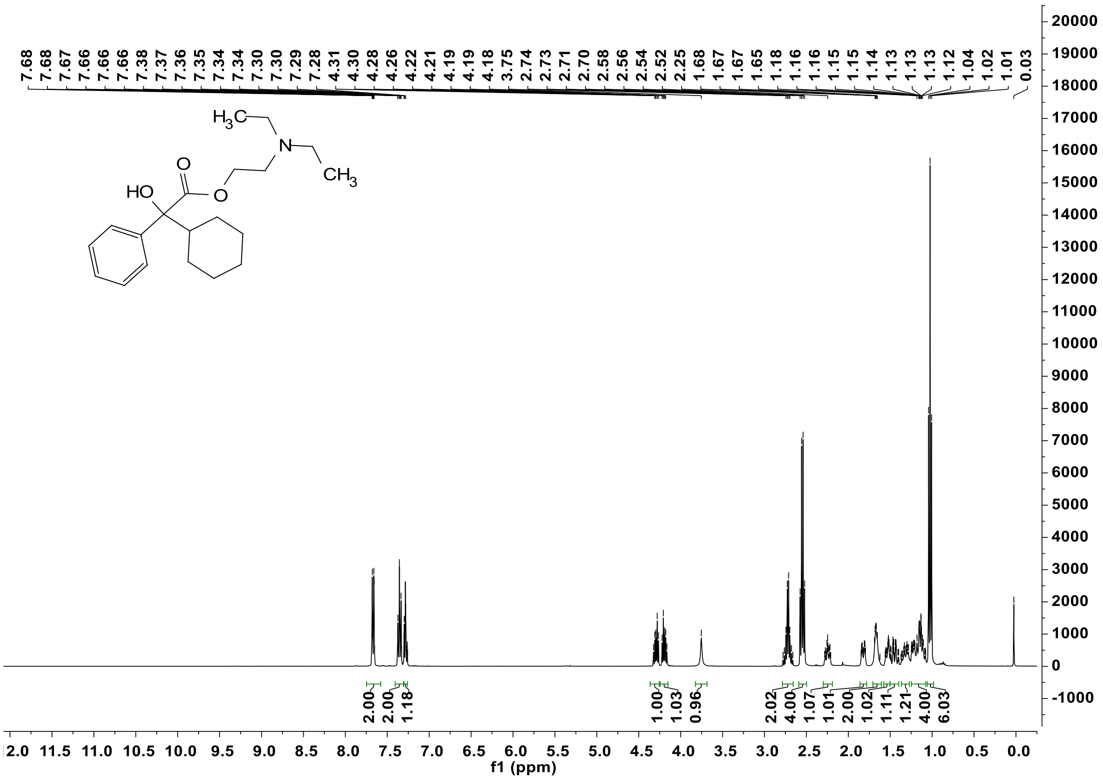

Supplementary Figure 116. ^{1}H NMR spectra of compound **13**.

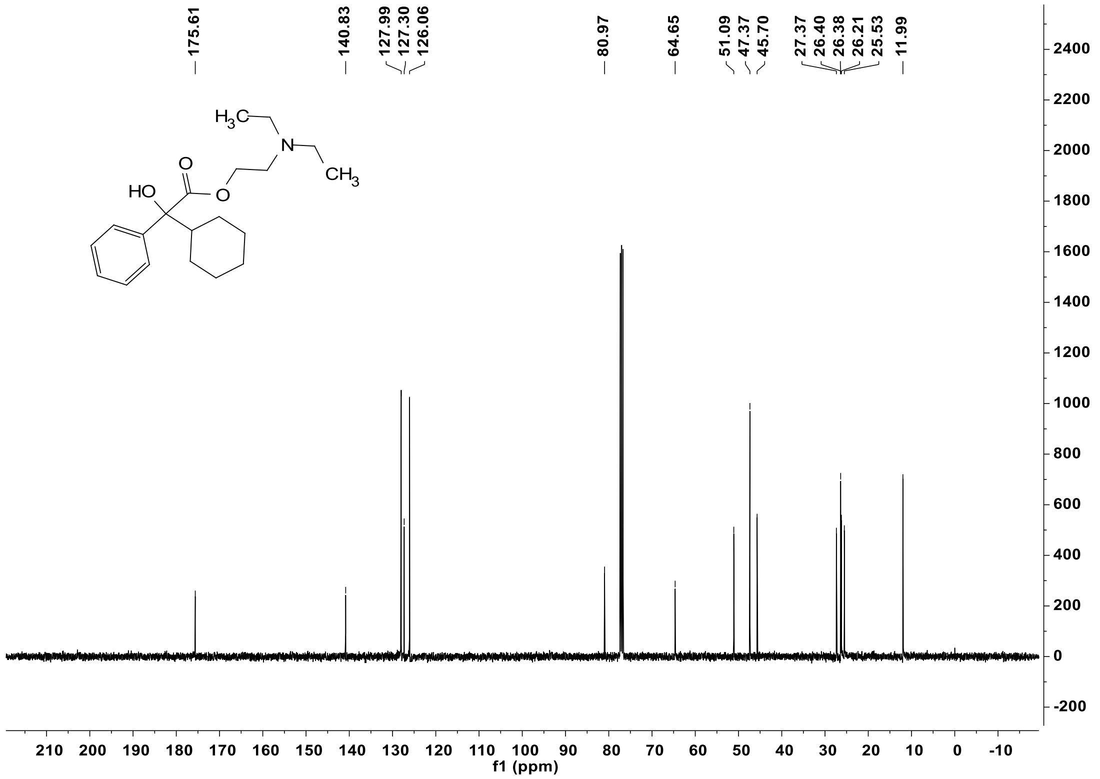

Supplementary Figure 117. ^{13}C NMR spectra of compound **13**.

2-(2-cyclohexyl-2-hydroxy-2-phenylacetoxy)-*N*,*N*-diethyl-*N*-methylethan-1-aminium iodide (14)

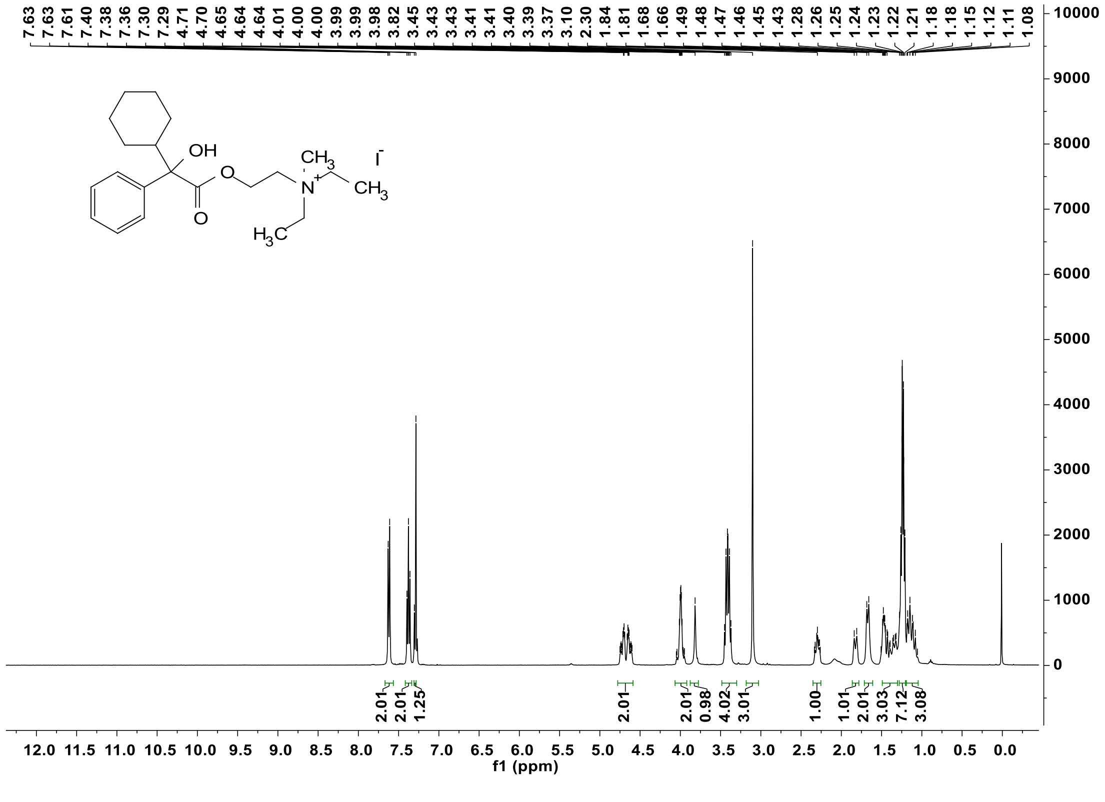

Supplementary Figure 118. ^{1}H NMR spectra of compound **14**.

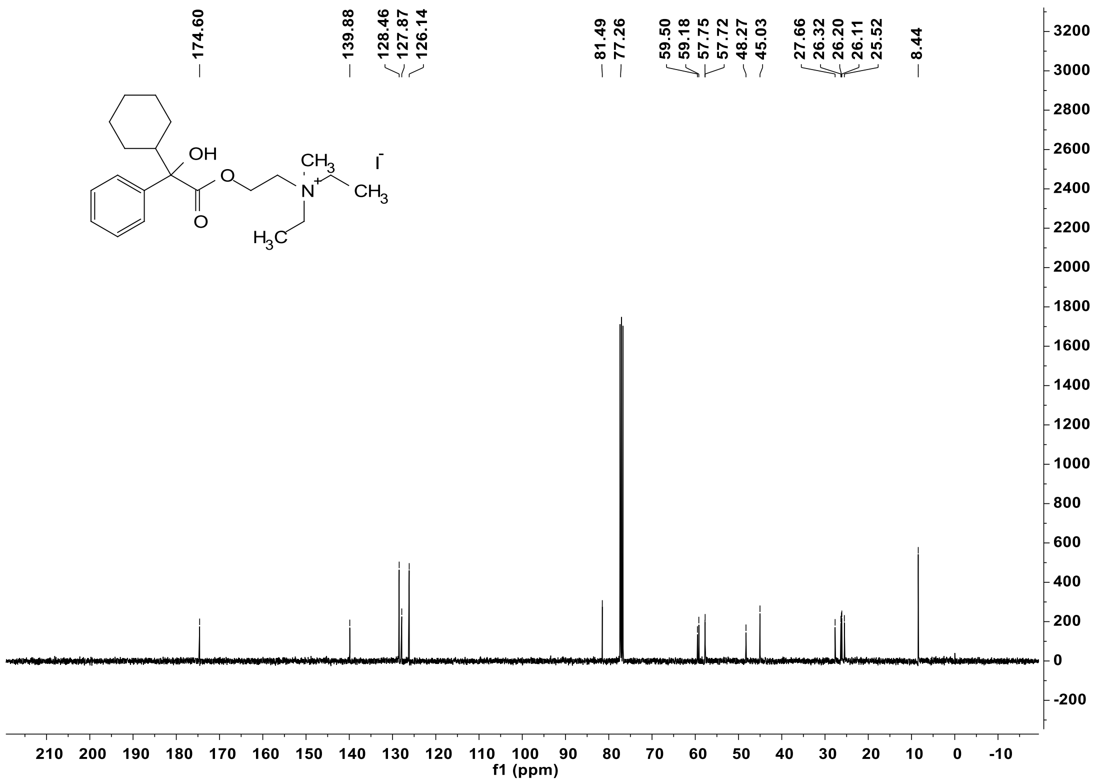

Supplementary Figure 119. ^{13}C NMR spectra of compound **14**.

5-([1,1'-biphenyl]-4-yl)-3-(*p*-tolyl)oxazolidine-2,4-dione (15)

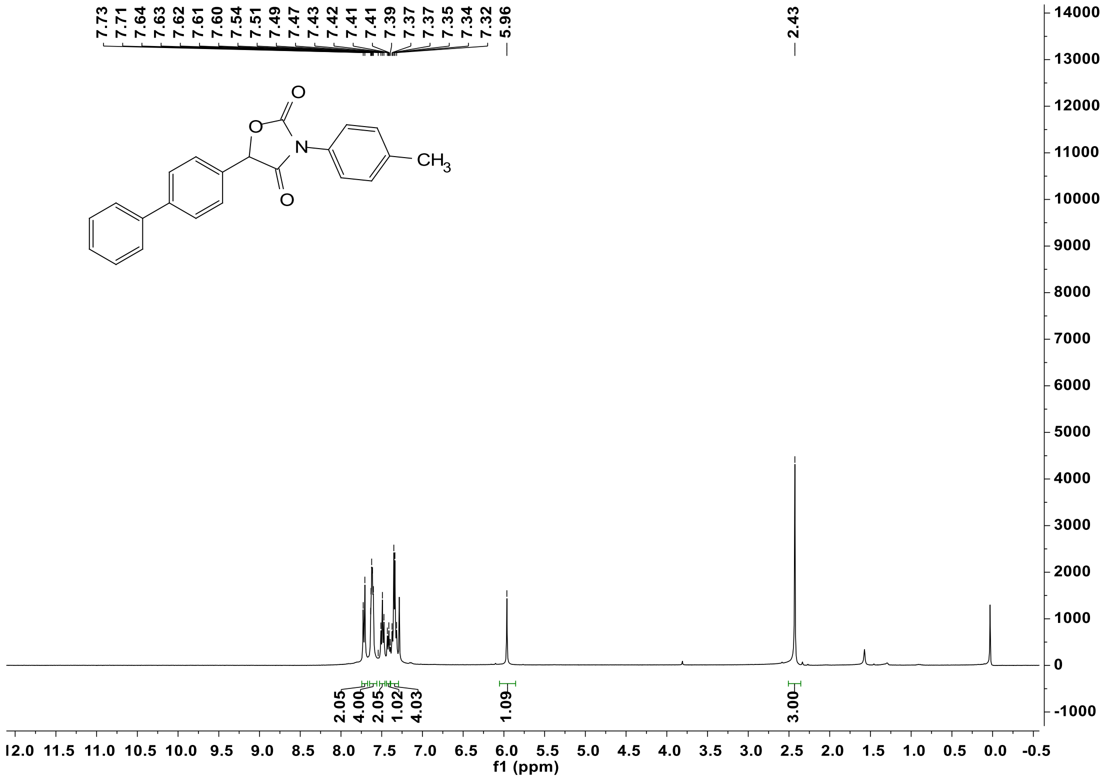

Supplementary Figure 120. ^{1}H NMR spectra of compound **15**.

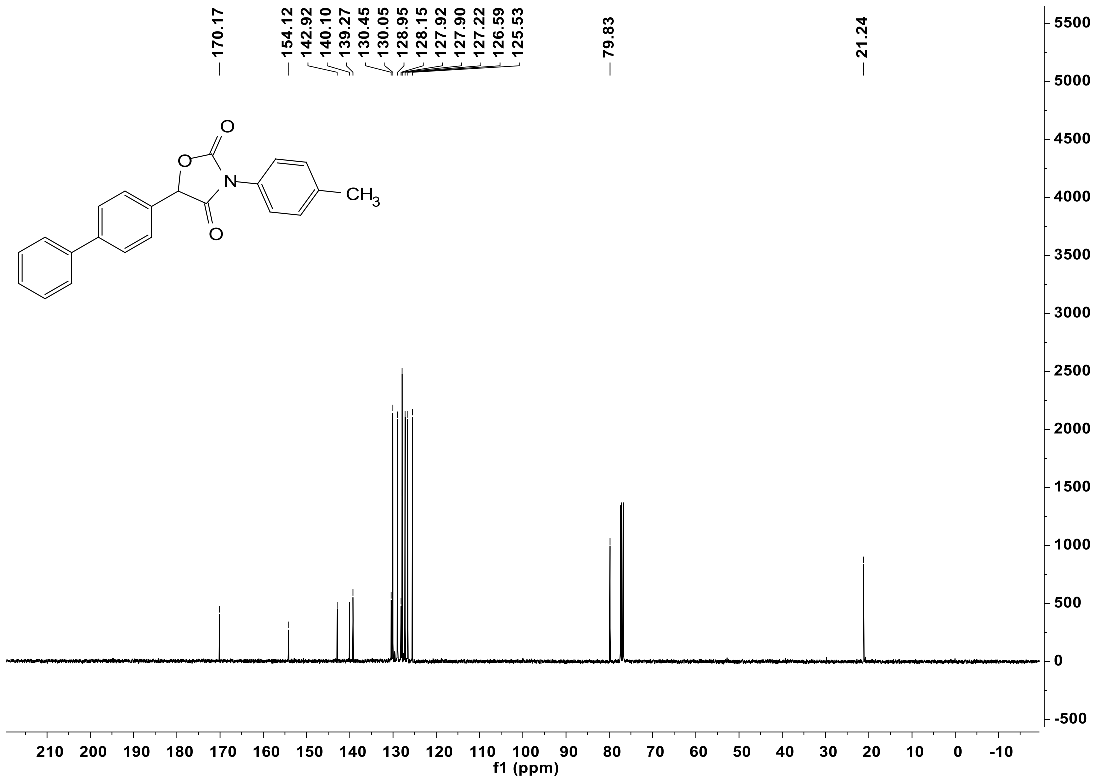

Supplementary Figure 121. ^{13}C NMR spectra of compound **15**.
